# Diastereoselective arylation of bis-lactim ethers catalyzed by *N*-coordinating ylide-functionalized phosphine (NYPhos)†

**DOI:** 10.1039/d5sc02814k

**Published:** 2025-06-30

**Authors:** Daniel Sowa Prendes, Julian Löffler, Ivan Martins Barreto, Nagesh Sankaran, Henning Remm, Ronaldo Nascimento de Oliveira, Viktoria H. Gessner, Lukas J. Gooßen

**Affiliations:** a Fakultät Chemie und Biochemie, Organische Chemie I, Ruhr-Universität Bochum Universitätsstr. 150 44801 Bochum Germany lukas.goossen@rub.de; b Fakultät Chemie und Biochemie, Anorganische Chemie II, Ruhr-Universität Bochum Universitätsstr. 150 44801 Bochum Germany viktoria.daeschlein-gessner@rub.de; c Laboratory of Synthesis of Bioactive Compounds, Department of Chemistry, Federal Rural University of Pernambuco (UFRPE) Recife 52171-900 Pernambuco Brazil

## Abstract

The palladium-catalyzed diastereoselective arylation of Schöllkopf bis-lactim ethers has recently emerged as a convenient entry to enantioenriched arylglycine derivatives. Key limitations of the prototypical protocol have now been overcome by the use of the customized, diadamantyl-substituted *N*-coordinating ylide-functionalized phosphine ligand ^Pip^adYPhos. Catalyst generated from 1-methylnaphthyl palladium bromide dimer and ^Pip^adYPhos effectively promote the coupling of aryl chlorides with a commercially available, valine-based Schöllkopf-reagent in 97% yield and a diastereoselectivity of 98 : 2. The arylglycines are accessible in high yields and enantiomeric excess by hydrolysis of the products. The correlation between diastereoselectivity and ligand properties identified the nature of the secondary metal–ligand interaction as a key factor in determining the selectivity of the arylation of bis-lactim ethers. Computational studies in combination with a quantitative structure–activity relationship analysis revealed that the rate-limiting step varies depending on the phosphine ligands used and that the secondary metal–ligand interaction determines diastereoselectivity.

## Abstract 2

A catalyst generated from 1-methylnaphthyl palladium bromide dimer and ^Pip^adYPhos enables aryl chloride coupling with a valine-based Schöllkopf reagent in up to 97% yield and >25 : 1 diastereomeric ratio, providing access to chiral arylglycines.

## Graphical abstract

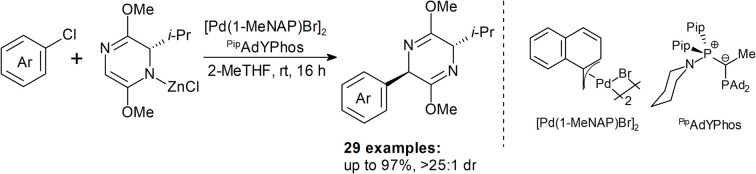

## Introduction

**Fig. 1:**
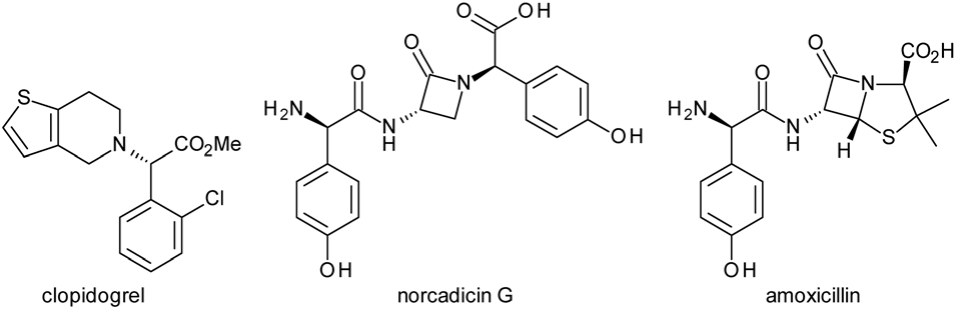
Bioactive chiral arylglycines.

Enantioenriched arylglycines are key structural components in bioactive molecules. Several top-selling drugs, such as the antiplatelet drug clopidogrel, the monocyclic β-lactam antibiotic norcadicin G or the penicillin derivative amoxicillin include this moiety (Fig. 1).

The significance of arylglycine building blocks in drug discovery has triggered the development of numerous protocols for their asymmetric synthesis.^1^ Established methods include the (dynamic) kinetic resolution of racemic esters,^2^ amides,^3^ or hydantoins^4^ as well as asymmetric variants of the Strecker^5^ and Petasis–Borono Mannich reaction.^6^ Asymmetric syntheses are possible also *via* hydrogenation of *N*-aryl α-amino esters,^7^ catalytic N–H insertions and additions,^8^ α-aminations of carboxylic acids,^9^ Knoevenagel condensation/epoxidation/esterification sequences,^10^ or Sommelet-Hauser rearrangements.^11^

**Scheme 1:**
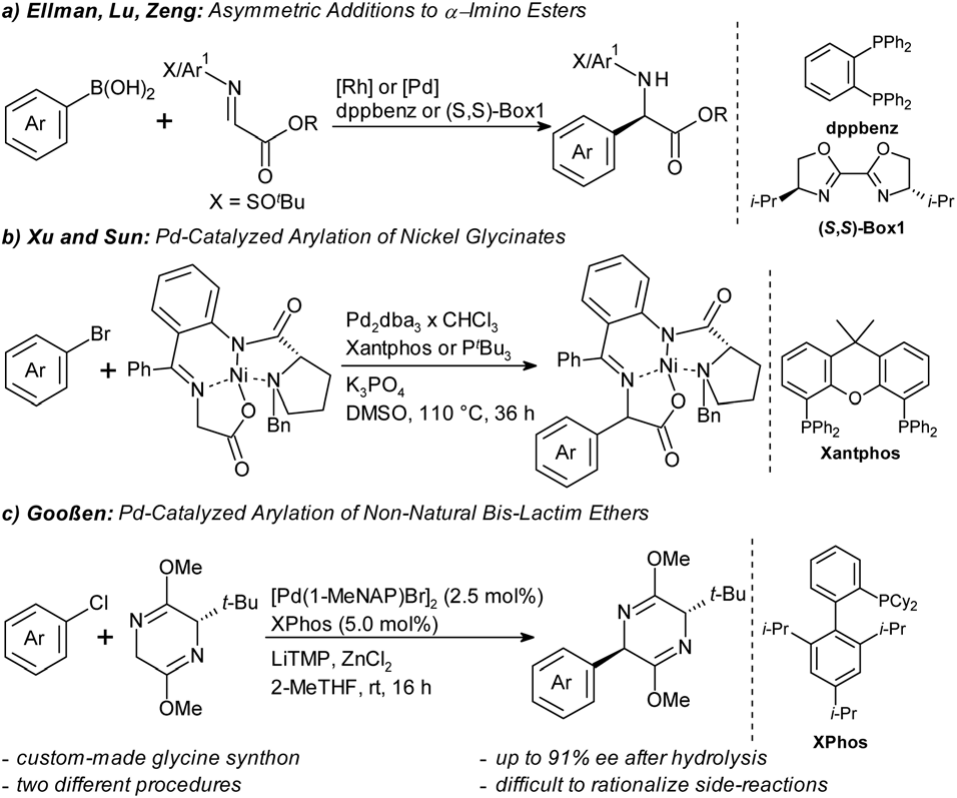
Catalytic arylations of glycine synthons.

Especially in the context of drug discovery, catalytic asymmetric arylations of glycine derivatives are advantageous over these traditional methods, since they allow the introduction of an entire amino acid moiety into functionalized arene building blocks with defined stereochemistry (Scheme 1). In most known syntheses, aryl nucleophiles are added to imine synthons. Ellman and Lu disclosed Rh- and Pd-catalyzed additions of arylboronic acids to chiral *N-tert*-butanesulfinyl imino esters (Scheme 1a).^12^ Zeng disclosed an enantioselective arylation of prochiral α-imino esters with chiral bis-oxazoline palladium complexes.^13^ Arylations of α-imino esters have also been achieved by redox chemistry^14^ or three-component reactions.^15^

Methods that utilize abundant aryl halides as electrophilic aryl sources are particularly desirable. An early example for this synthetic approach is the work by Xu and Sun, who coupled pre-formed nickel complexes of chiral glycine synthons with aryl bromides (Scheme 1b).^16^ As a straight-forward alternative, we recently introduced a palladium-catalyzed diastereoselective arylation of *tert*-leucine-derived Schöllkopf bis-lactim ethers (Scheme 1c).^17^ Its key advantages are the use of inexpensive, widely available aryl chlorides as aryl source and the predictability of the absolute stereochemistry of the products from that of the bis-lactim ether synthon. The arylglycine is liberated in high enantiomeric excess (ee) *via* hydrolytic cleavage along with the amino acid auxiliary. Arylations of bis-lactim ethers have previously only been possible *via* (non-regiospecific) Friedel–Crafts or aryne pathways or using stoichiometric arene–manganese complexes.^18^

However, our prototypical protocol, which was based on the commercially available XPhos ligand, still had several drawbacks that limited its practical applicability. In order to achieve high diastereoselectivities, a custom-made bis-lactim ether derived from the non-natural amino acid *tert*-leucin proved to be crucial. However, even with this approach, electron-deficient aryl halides gave only mediocre results showing competing side-reactions, such as redox-aromatization of the bis-lactim ether substrates/products.

We have now overcome the limitations of the initial protocol by identifying structural motifs that influence the selectivity and activity of ligands and tailored a new ligand, ^Pip^adYPhos. Its ylide group makes the palladium center highly electron-rich, enabling the conversion of aryl chlorides, while its bulky adamantyl substituents and the coordinating piperidine groups ensure that even the arylation of a simple, l-valine derived bis-lactim ether proceeds in high chemo- and diastereoselectivities. The reaction pathway was elucidated by DFT calculations, and the ligand effects on the preferred reaction pathway by statistical analyses.

## Results and discussion

### Reaction design and discovery

**Scheme 2:**
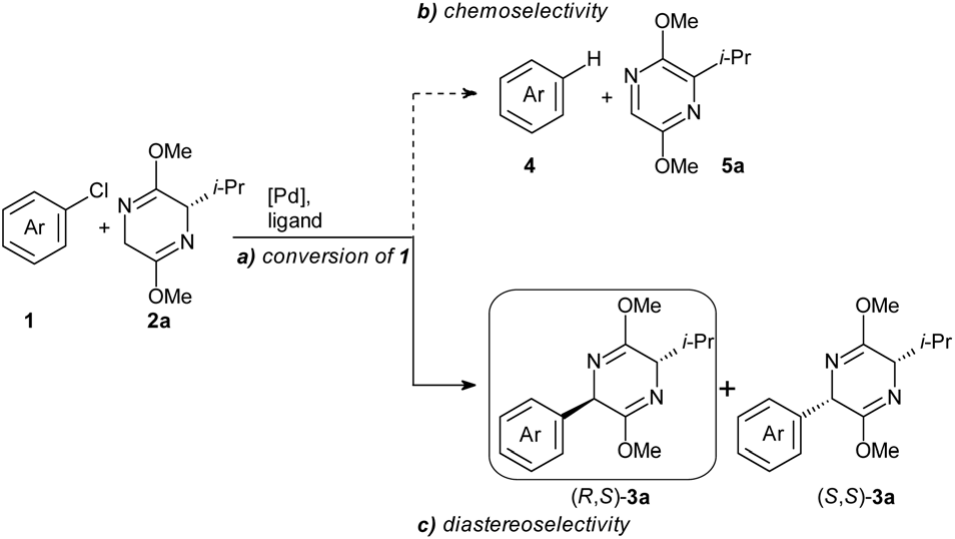
Key challenges for arylation of 2a.

In our search for an efficient ligand system for the so far elusive arylation of l-valine derived bis-lactim ether 2a, three key challenges had to be addressed (Scheme 2): (a) the ligand must donate sufficient electron density to the Pd center to facilitate the oxidative addition of aryl chloride 1. (b) The coupling must proceed chemoselectively, overcoming the competing redox-aromatization of the bis-lactim ether substrate (or the arylated product), which leads to the formation of the dehalogenated arene 4. (c) A high level of diastereoselectivity must be ensured.

In our initial protocol, we thoroughly optimized the reaction conditions for the arylation of *tert*-leucine derived bis-lactim ethers, identifying 1-methylnaphthyl palladium bromide dimer [Pd(1-MeNAP)Br]_2_ as the best palladium precursor, XPhos as the optimal ligand, lithium-2,2,6,6-tetramethylpyrrolidide (LiTMP) as the preferred base, ZnCl_2_ as the best additive, 2-MeTHF as the optimal solvent, and room temperature as the most favorable reaction temperature.^17^ While our initial protocol demonstrated that the arylation could proceed without a zinc additive, the addition of ZnCl_2_ significantly improved conversion, chemoselectivity, and functional group tolerance.^17^

**Scheme 3:**
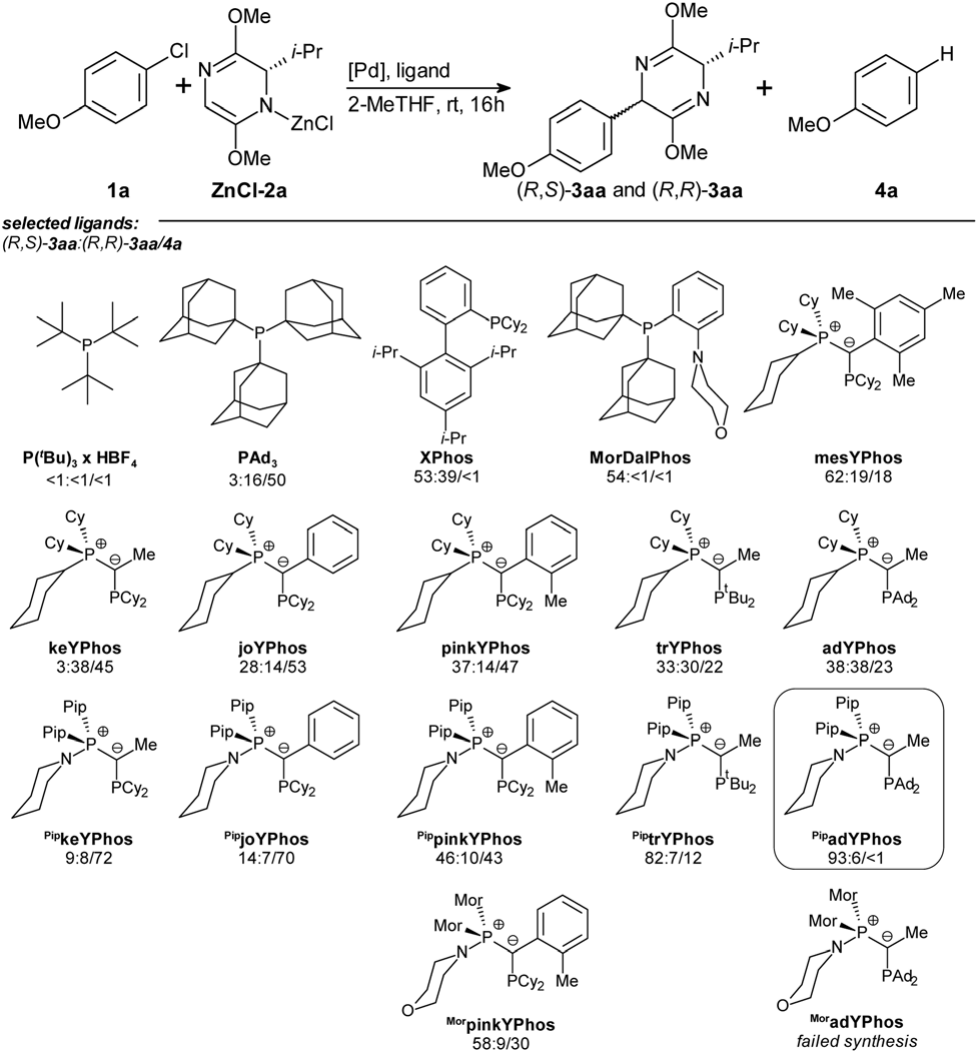
Ligand screening. Conditions: 1a (0.25 mmol), ZnCl-2a (2.0 equiv.), [Pd(1-MeNAP)Br]_2_ (2.5 mol%), ligand (5.0 mol%), 2-MeTHF (2.0 mL), rt, 16 h; yields of (*R*,*S*)-3aa: (*R*,*R*)-3aa/4a determined by GC analysis using *n*-hexadecane as internal standard. ZnCl-2a was prepared by pre-stirring 2a (2.0 equiv.), LiTMP (2.0 equiv.) and ZnCl_2_ (2.0 equiv.) at rt, for 16 h in 2-MeTHF (0.5 mL).

However, for maximal reproducibility of the yields, it now proved to be vital that the bis-lactim ether 2a is partially converted into its zinc salt ZnCl-2a before starting the catalytic reaction. Hence, we now stirred 2a, the LiTMP and ZnCl_2_ for as long as 16 hours before adding it to all other reagents. A comparison of the absolute energies of optimized geometries of metalated bis-lactim ethers identified the *N*-zincated species as the most stable, suggesting it is the predominant form involved in the catalytic reaction (Table S11†). We chose the reaction of 2 equiv. of zincated ^i^Pr-substituted bis-lactim ether ZnCl-2a with 4-chloroanisole 1a as a model system and systematically screened 40 ligands (Scheme 3 and Table S1†) under the optimized conditions.

Most of the trialkylphosphine ligands that we tested, including P^*t*^Bu_3_, PAd_2_^*n*^Bu, PAd_2_^*t*^Bu gave only low conversion. PAd_3_ provided high conversion but poor chemo- and diastereoselectivity, while XPhos achieved high conversion and chemoselectivity but failed to deliver satisfactory diastereoselectivity. Other ligands of the Buchwald-type family were less effective. The highest diastereo- and chemoselectivity was observed with MorDalPhos, albeit at an unsatisfactory conversion. This ligand features two extremely bulky adamantyl substituents and a coordinating morpholine moiety.

Ylide-functionalized phosphines (YPhos) are known to effectively promote oxidative addition of aryl chlorides, and have also proven to be the ligands of choice for challenging transmetalations.^19^ At the same time, YPhos ligands offer high modularity, where small modifications in the remote ligand backbone can significantly impact key steps in challenging catalytic cycles. Several YPhos ligands gave high conversion of 1a. However, they all steered the reaction towards the undesired redox-aromatization pathway. Systematic studies suggested that the diastereoselectivity increases with the steric bulk of the substituent at the ylide carbon: For example, with methyl substituted keYPhos, the *Z*-isomer was formed preferentially, whereas phenyl substituted joYPhos gave an *E*-diastereoselectivity of 2 : 1, and the *o*-tolyl substituted pinkYPhos of 3 : 1. Even higher chemo- and diastereoselectivity were observed with the mesityl substituted mesYPhos. Additionally, increasing the steric bulk on the phosphine substituents (keYPhos < trYPhos < adYPhos) also led to a higher yield of the desired product (*R*,*S*)-3aa.

Based on this evaluation of known ligands, we concluded that a ligand possessing (1) the electron-donating ability of a YPhos ligand with (2) bulky substituents at the ylide or the phosphine group and (3) the *N*-donor group of the MorDalPhos should be able to reach full conversion and high chemo- and stereoselectivity.

*N*-Coordinating YPhos (NYPhos) ligands have been systematically investigated in the context of ketone arylations.^20^ In the arylation of bis-lactim ethers, replacing the cyclohexyl groups in the onium moiety with cyclic amines proved to be beneficial, especially for bulky ligands, with the yield of (*R*,*S*)-3aa increasing in the order pinkYPhos < ^Pip^pinkYPhos < ^Mor^pinkYPhos. Building on these ligand trends, we synthesized two new ligands, ^Pip^trYPhos and ^Pip^adYPhos, featuring coordinating piperidine moieties and bulky *tert*-butyl and adamantyl-substituents at the phosphorous atom, respectively.

**Scheme 4:**
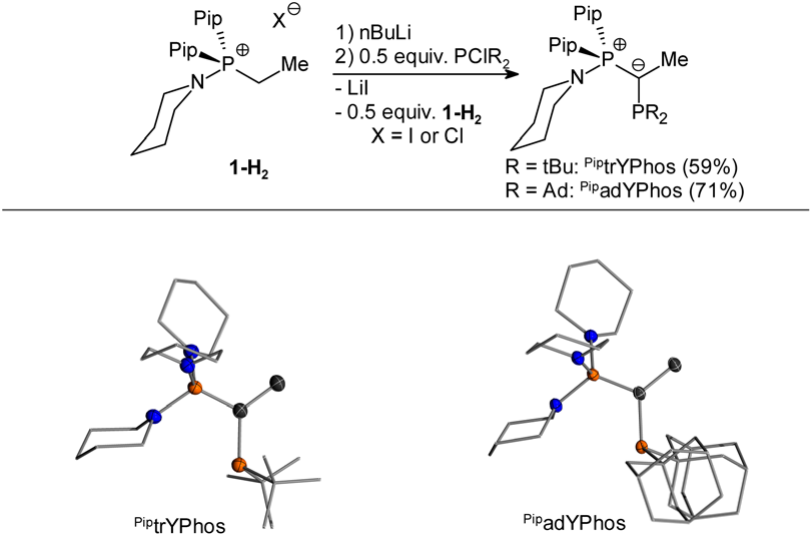
Synthesis and crystals of ^Pip^trYPhos and ^Pip^adYPhos. Molecular structures, were determined from single crystals obtained by slow evaporation from saturated C_6_D_6_ solutions. Ellipsoids drawn at the 50% probability level, and hydrogen atoms omitted for clarity.

Scheme 4 illustrates the synthesis of ^Pip^trYPhos and ^Pip^adYPhos following a “transylidation pathway” in which the intermediate formed ylide functions as a reagent and as base. Both ligands were obtained in reasonable yields of 59 and 71%, respectively. Unfortunately, this synthetic concept could not be extended to ligands bearing bulky substituents at both the phosphino group and the ylide backbone, presumably due to steric hindrance preventing the phosphorylation of the ylide precursor. Based on the findings for ^Mor^pinkYPhos, the adamantyl-substituted ^Mor^adYPhos ligand also seemed to be a worthwhile target, but this ligand could not be obtained as the respective ylide was found to be unreactive towards the chlorophosphine. Crystals of ^Pip^trYPhos and ^Pip^adYPhos were grown by solvent evaporation from saturated C_6_D_6_ solutions, allowing the determination of their molecular structures by X-ray diffraction analysis. Both ligands feature slightly smaller P–C–P angles (114.0(1) and 115.3(1)°) in comparison to the cyclohexyl-substituted YPhos ligands (116.8(1)–120.5(1)°) as a consequence of the increased steric demand of the *tert*-butyl and adamantyl substituents. This is further supported by the calculated percent buried volume of 54.6 and 55.1%, respectively (*cf.* % Vbur = 47.9% for joYPhos).^21^

With ^Pip^adYPhos, we observed a near-exclusive chemoselectivity and a high diastereomeric ratio (dr) of 16 : 1 in favor of (*R*,*S*)-3aa at full conversion of the aryl chloride 1a. Attempts to reduce the amount of reagent ZnCl-2a were unsuccessful, as comparative reactions employing lower equivalents of bis-lactim ether resulted in diminished conversion of aryl chloride 1a (Table S2†).

### Reaction scope

Given these exceptional chemo- and diastereoselectivities in our challenging test reaction, we proceeded to explore the scope of the new protocol (Table 1). In the optimized procedure, we first prepared the ZnCl-2a solution by stirring 2a, LiTMP and ZnCl_2_ in 2-MeTHF for 16 hours at room temperature. This solution was then added, without further purification, to a reaction vial containing the pre-catalyst [Pd(1-MeNAP)Br]_2_, the ligand ^Pip^adYPhos, the solvent 2-MeTHF and the desired aryl chloride substrate 1. The resulting mixture was stirred at room temperature for 16 hours.

**Table 1:** Scope with regard to aryl and alkenyl chlorides^a^

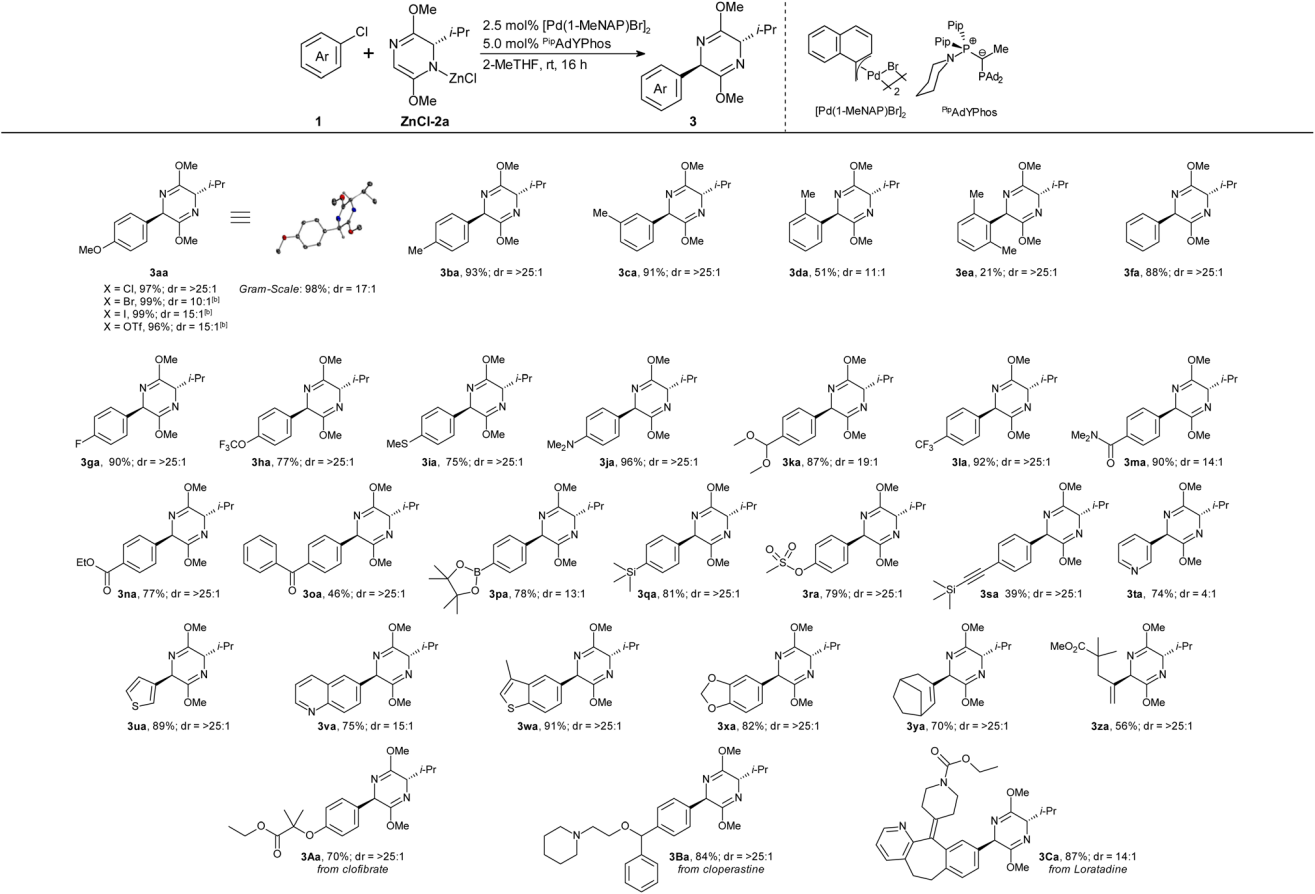

a Conditions: 1 (0.5 mmol), ZnCl-2a (2.0 equiv.), [Pd(1-MeNAP)Br]_2_ (2.5 mol%), ^Pip^adYPhos (5.0 mol%), 2-MeTHF (4.0 mL), rt, 16 h; isolated yields; dr determined by ^1^H NMR analysis.

b Yield and dr determined by GC analysis using *n*-hexadecane as internal standard. ZnCl-2a was prepared by pre-stirring 2a (2.0 equiv.), LiTMP (2.0 equiv.) and ZnCl_2_ (2.0 equiv.) at rt, for 16 h in 2-MeTHF (1.0 mL).

Under these conditions, commercially available ^i^Pr-substituted bis-lactim ether 2a was successfully coupled with a wide-range of functionalized aryl and alkenyl halides in high chemo- and diastereoselectivity. Aryl chlorides, bromides (99%, dr = 10 : 1), iodides (99%, dr = 15 : 1) and triflates (96%, dr = 15 : 1) all gave similarly high yields as shown for product 3aa. The dr was increased further by chromatographic work-up. *m*- and *p*-Substituted aryl chlorides yielded comparable results, whereas bulky substituents in *o*-position adversely affected the yield and/or dr of the arylation (3ba–3ea). Many common functional groups are tolerated, including amines, amides and esters (3fa–3oa). Even ketone-functionalized 3oa was formed in high yield despite the known tendency of ketones to undergo aldol-reactions with bis-lactim ethers.^22^ Notably, the protocol tolerates groups that can be used for follow-up cross-coupling reactions, such as silyl and boronate groups (3pa–3sa). The reaction also extends to heterocycles (3ta–3xa), even including pyridine and thiophene. It was also successfully employed in late-stage functionalizations of drug molecules (3Aa–3Ca).

Comparative experiments confirmed that the new method is vastly superior to the previous state of the art.^17^ With the ^Pip^adYPhos system, 3ja can be obtained in 96% and 3la in 92% from the commercially available, valine-based bis-lactim ether 2a. In contrast, these aryl halides gave only 66% and 32% with the XPhos system, despite employing the custom-made, *tert*-leucine derived auxiliary. The electron-withdrawing aryl chlorides 1l–1n, which had given substantial amounts of redox-aromatization byproducts with the XPhos protocol, were now smoothly arylated in high chemo- and stereoselectivities.

The scalability of the process was demonstrated in the gram-scale synthesis of 3aa in 98% yield and 17 : 1 dr. XRD analysis of 3aa confirmed a *trans*-configuration of the products. Only for the bulky 2-chloro-1,3-dimethylbenzene (2e), neither the coupling constants in 1D-NMR nor 2D-NMR investigations allowed us to pinpoint the absolute stereochemistry of the dominating stereoisomer (see chapter 10 in ESI†). It is tentatively drawn as the expected *trans*-isomer, but a *cis*-configuration cannot be excluded.

### Further application

**Scheme 5:**
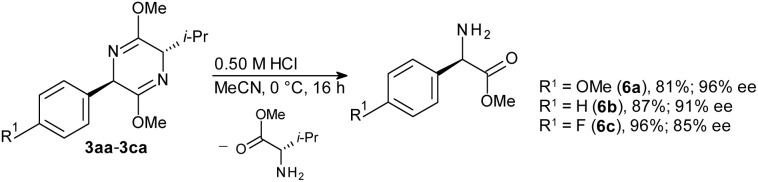
Hydrolysis of bis-lactim ethers.

Hydrolytic cleavage of the bis-lactim ethers afforded the corresponding arylglycines with high enantiomeric excess (ee) (Scheme 5). When starting from commercially available bis-lactim ether substrate 2a with an ee above 95%, products with ee values of 85–96% were obtained, demonstrating that the arylation-hydrolysis sequence proceeds with high stereoselectivity.

### Mechanistic studies

**Scheme 6:**
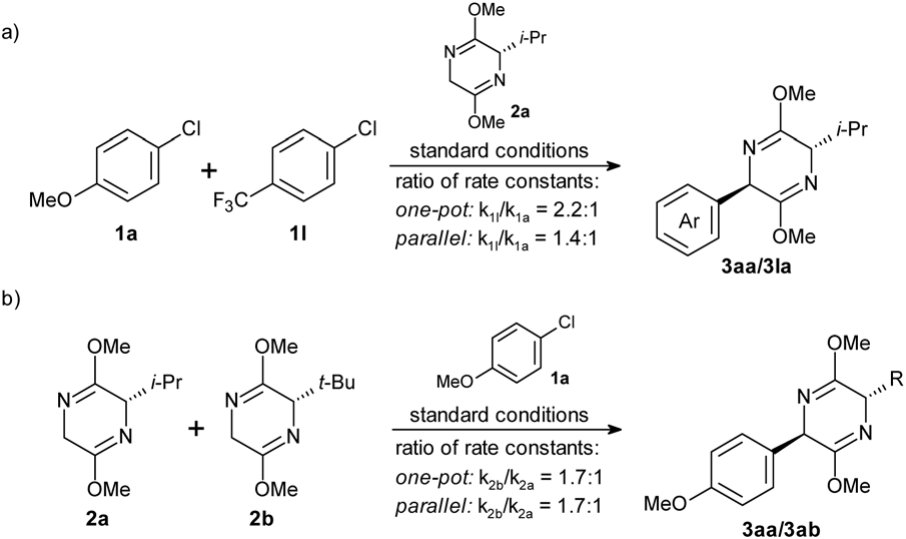
Competition experiments (conditions see Table 1 and ESI chapter 9†).

To shed light on the mechanism of the bis-lactim ether arylation, we conducted competition experiments by monitoring reactant and product concentrations over time (Scheme 6 and ESI chapter 9†). In one-pot experiments, we assumed second-order kinetics, as the conversion depended on the concentration of the bis-lactim ether (see Table S2†), and we extracted rate constant ratios directly from the starting material concentrations. In parallel experiments using a two-fold excess of bis-lactim ether, we applied pseudo-first-order kinetics and determined rate constant ratios by linear fitting of data points from the early to mid stages of the reaction.

The one-pot reaction of electron-rich 1a and electron-poor aryl chloride 1l showed a preferential conversion of 1l in a *k*_1l_ : *k*_1a_ = 2.2 : 1 ratio (Scheme 6a), confirming that the initiating oxidative addition step is facilitated by electron-deficient substituents. However, in the corresponding parallel experiments, the rate ratio decreased to *k*_1l_ : *k*_1a_ = 1.4 : 1. This suggests that while oxidative addition is accelerated by electron-withdrawing substituents, it is not the rate-determining step of the catalytic cycle. If oxidative addition were rate-limiting, similar rate ratios would be expected in both one-pot and parallel experiments. The fact that the higher reactivity of 1l in the oxidative addition did not translate into a higher overall rate means that oxidative addition and reductive elimination are so fast that electronic factors influencing these steps are without consequence on the overall rate, or that oxidative addition and reductive elimination are similarly fast so that electronic factors influencing both steps in opposite directions partially offset each other.

We also explored the influence of the bis-lactim ether substituent on the reaction rate (Scheme 6b). The one-pot competition experiment of ^i^Pr- (2a) and ^*t*^Bu-substituted bis-lactim ethers (2b) showed preferential coupling of the bulkier and slightly more electron-rich reagent 2b (*k*_2b_ : *k*_2a_ = 1.7 : 1). The parallel experiment gave a similar ratio of rates (*k*_2b_ : *k*_2a_ = 1.7 : 1), suggesting that the reaction step that is so strongly influenced by this structural variation of the nucleophile is overall rate-determining. Both the reductive elimination and the transmetalation would be accelerated by electron-rich substituents. However, while reductive elimination is usually accelerated by increased steric bulk, transmetalation is likely to be retarded by it. Therefore, we assume that the reductive elimination step is rather slow. However, if it were the only rate-limiting step, the electron-rich aryl chloride 1a should have given a higher conversion than 1l in the parallel experiment shown in Scheme 6a, since reductive elimination typically proceeds faster for electron-rich substrates. The most likely explanation for these findings is that oxidative addition and reductive elimination proceed at comparable rates and are slower than the transmetalation.

To further support this hypothesis, we evaluated various potential reaction pathways using density functional theory (DFT) calculations on the PBE0-D3/def2TZVP + LANL2TZ(f)/SMD(THF)//PBE0-D3/def2-SVP/PCM(THF) level of theory using a continuous solvent model for THF. We chose chlorobenzene 1f and the zincated valine-derived bis-lactim ether ZnCl-2a with one explicit THF solvent molecule as substrates. The pathways were calculated for both ^Pip^adYPhos and keYPhos to disentangle ligand effects. We identified three competing pathways leading to the *trans*- and *cis*-arylation product as well as to redox-aromatization. While we had previously attributed the formation of hydrodehalogenated arene 4 and aromatized bis-lactim ether reagent 5a (or arylated product) to competing hydride elimination and reductive elimination pathways, our calculations now reveal that the pathways leading to these potential products diverge as early as the transmetalation step. The zincated bis-lactim ether can coordinate to the Pd center either *via* its prochiral carbon atom – *cis*- or anti to the iso-propyl substituent – or *via* the nitrogen atom adjacent to the iso-propyl group. The first two cases lead to diasteroselective arylations, whereas in the latter case, the reaction follows the undesired redox-aromatization pathway.

**Fig. 2:**
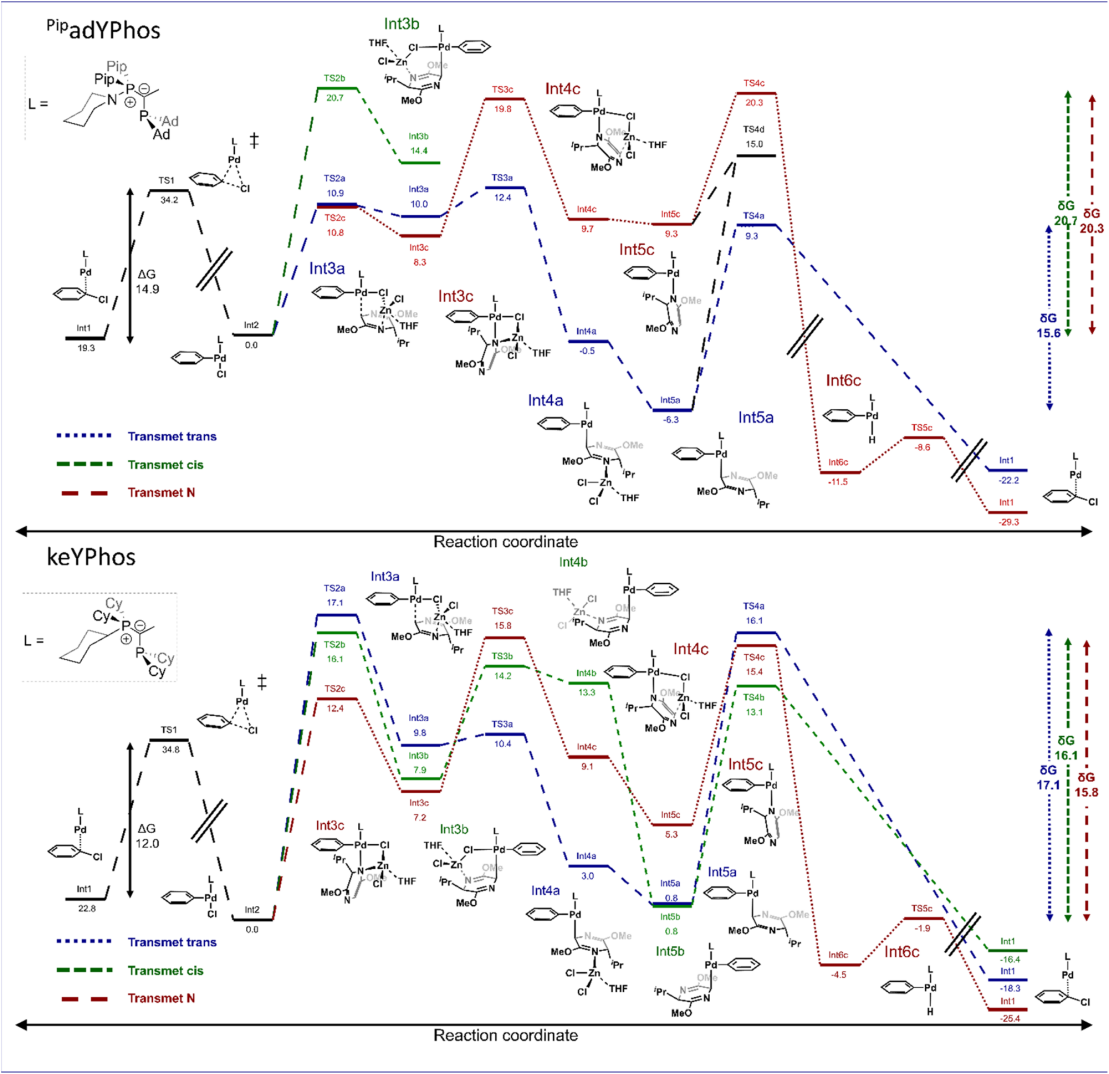
Comparative energy profile (in kcal mol^−1^) in the arylation of valine-derived bis-lactim ether 2a and chlorobenzene 1f using ^Pip^adYPhos (top) and keYPhos (bottom). Energies (PBE0-D3/def2TZVP + LANL2TZ(f)) are given relative to the oxidative addition complex and the zincated bis-lactim ether with two coordinating THF molecules (see the ESI for details†).

All three pathways were calculated and their relative efficiencies were assessed based on the energy span model by Kozuch and Shaik.^23^Fig. 2, shows the energy profiles for ^Pip^adYPhos and keYPhos.

For ^Pip^adYPhos, the oxidative addition of chlorobenzene 1f is predicted to proceed with a barrier of 14.9 kcal mol^−1^, which is in agreement with the experimental findings that this step proceeds smoothly at room temperature. The formation of the desired *trans*-arylation product (Fig. 2 top, blue pathway) starts with the transmetalation of the zincated bis-lactim ether with formation of a Pd–carbon bond anti to the iso-propyl group. Following two rather low activation barriers for the Pd–carbon bond formation (10.9 kcal mol^−1^, TS2a) and the cleavage of the Pd–Cl bond (2.4 kcal mol^−1^, TS3a), intermediate Int4a is formed. The elimination of the zinc chloride from the nitrogen atom of the bis-lactim ether leading to Int5a is exergonic. The reductive elimination of the *trans*-product (*R*,*S*)-3fa*via*TS4a has a substantial activation barrier of 15.6 kcal mol^−1^. This barrier represents the highest energy span of the pathway yielding (*R*,*S*)-3fa.

This profile is in excellent agreement with the kinetic studies. With its energy span of 14.9 kcal mol^−1^, the oxidative addition is expected to proceed at a similar rate as the reductive elimination with its span of 15.6 kcal mol^−1^. In contrast, the transmetalation steps with a barrier of 12.4 kcal mol^−1^ (between Int2 and TS3a) should have minimal influence on the overall rate. This explains why the electronic effects of the aryl chloride, which are opposite for oxidative addition and reductive elimination, offset each other. It also explains why bulky substituents at the bis-lactim ether, which facilitate the reductive elimination reaction, increase the overall reaction rate (compare Scheme 6).

The pathway leading to the undesired regioisomer *via cis*-arylation starts with a high activation barrier of 20.7 kcal mol^−1^ for the transmetalation step towards Int3b. In this intermediate, the Pd coordinates to the prochiral carbon atom syn to the iso-propyl group (Fig. 2 up, green pathway). Since this barrier is already 5.1 kcal mol^−1^ higher than the total energy span of the *trans*-arylation pathway, the pathway leading to the (*S*,*S*)-3fa product is strongly disfavored. This aligns well with the experimentally observed high diastereoselectivity of the ^Pip^adYPhos ligand. The pathway for the redox-aromatization of the bis-lactim ether starts with the coordination of the Pd center by the nitrogen rather than the prochiral carbon atom of the bis-lactim ether *via* a low activation barrier of only 10.8 kcal mol^−1^ (Fig. 2 up, red pathway). The release of the zinc chloride with formation of Int5c also proceeds *via* low activation barriers. The energy span of this pathway (20.3 kcal mol^−1^) is determined by the energy difference of the low-lying oxidative addition complex Int2 and the transition state TS4c of the β-hydride elimination which liberates the aromatized bis-lactim ether 5a. The reductive elimination of the arene 4a is exergonic and has a negligible barrier of 2.9 kcal mol^−1^. The fact that the energy span of this pathway is 5.3 kcal mol^−1^ larger than that of the desired cross-coupling to (*R*,*S*)-3fa agrees well with the experimentally observed high chemoselectivity obtained with the ^Pip^adYPhos ligand.

There are interconnections between the pathways, for example the transition between Int5c and Int5a with a barrier of 15.0 kcal mol^−1^. However, these interconversions are less favorable than the direct pathway to the *trans*-product. Thus, these additional pathways are omitted in Fig. 2 for clarity, as they do not impact the key conclusions of the calculations.

For comparison, we also calculated the energy profile of the catalysis using keYPhos as ligand (Fig. 2, bottom). Experimentally, we observed a preference for the *cis*-product (*S*,*S*)-3aa and a significant amount of redox-aromatization in the reaction of 1a with ZnCl-2a (compare Scheme 3). The oxidative addition of chlorobenzene 1f, with an activation energy of 12.0 kcal mol^−1^, is comparable to that observed for ^Pip^adYPhos as ligand. The overall energy span for the desired *trans*-arylation pathway is determined by the energetic difference of the oxidative addition complex Int2 and the first TS of the transmetalation sequence (TS2a) as 17.1 kcal mol^−1^. The analogous transmetalation step (TS2b) leading to the *cis*-product has a slightly lower barrier of 16.1 kcal mol^−1^, accounting for the preferential formation of the (*S*,*S*)-diastereomer. However, the redox-aromatization pathway exhibits the lowest energy span of 15.8 kcal mol^−1^ (between Int2 and TS4c of β-hydride elimination). These findings are in excellent agreement with the experimental finding, which showed that with the keYPhos ligand and aryl chloride 1a, arene 4a is formed in 45%, along with 38% of the *cis*-arylation product and only 3% yield of the *trans*-product (*R*,*S*)-3aa.

To gain a deeper understanding of how ligand properties influence the catalytic outcome we attempted a correlation of the experimentally observed chemo- and diastereoselectivites with DFT-derived, physio-chemical descriptors of the phosphine ligands. We calculated the descriptors based on the oxidative addition complexes, as they appeared to be a better model system compared to the free ligands, since the selectivity determining steps originate from these complexes (see Int2 in Fig. 2). Models were developed through stepwise forward regression for feature selection of normalized descriptors. Ligands that demonstrated less than 10% conversion or only showed one of the compared two reactions were excluded. The remaining ligands were split into a training and a test set with a ratio of 25% according to the Kennard-Stone algorithm to obtain a uniform distribution over the descriptor space. The final model was validated with an additional experimental observation using RockPhos as ligand.

**Fig. 3:**
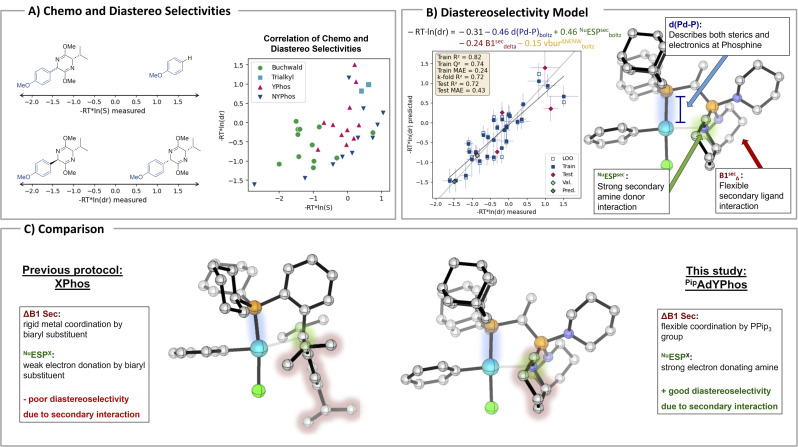
Multivariate descriptor-based modelling for determining the key ligand properties for selective bis-lactim ether arylation. Val. refers to the external validation using RockPhos as ligand. Pred. refers to the predicted results with ^Mor^adYPhos as ligand.

An initial analysis of the observed selectivities for the arylation of bis-lactim ethers revealed a weak correlation between chemoselectivity and diastereoselectivity (Fig. 3A). Ligands that exhibited high chemoselectivity tend to displayed similarly high diastereoselectivity, suggesting that the underlying factors governing these selectivities are interrelated. A plot of these selectivities illustrates the trends across different ligand classes. Notably, Buchwald-type ligands generally outperformed YPhos ligands in both metrics. However, the NYPhos ligand class demonstrated significant variability in performance, with certain ligands achieving exceptional selectivities.

Unfortunately, attempts to develop a robust model for the prediction of the chemoselectivity failed (see the ESI†), indicating that either the descriptors are inadequate for capturing the underlying relationship or that the data is insufficient to establish these connections. In contrast, a reliable and robust model could be established for the diastereoselectivity. Given the correlation between chemo- and diastereoselectivity it can be assumed that similar ligand properties are also decisive for the chemoselectivity.

In the obtained model for diastereoselectivity (Fig. 3B), the Pd-phosphine distance (d(Pd–P)) emerged as a critical descriptor, with larger Pd–P distances resulting in higher dr in favor of the *trans*-diastereomer. This descriptor is quite ambiguous as it is influenced by both steric and electronic effects. Additionally, the range of the sterimol parameter B1 for the secondary metal ligand interaction within the examined conformer ensemble, which represents a measure for the flexibility of that interaction, and the nucleophilic electrostatic potential (ESP) of the secondary donor atom revealed to be significant predictors. The negative coefficient for B1^sec^delta and the positive one for ^Nu^ESP^sec^ indicate that highly flexible, yet strong donors are advantageous for achieving good diastereoselectivity. These findings suggest that the secondary interaction plays a crucial role in stabilizing the catalytic intermediate and directing the diastereoselective pathway (see also the ESI,† page 63).

A comparison of the key ligand properties of the best ligand of our first bis-lactim ether arylation protocol, *i.e.* XPhos, with those of ^Pip^adYPhos reveals that the secondary interaction may also be the reason why the new ligand shows such a significantly improved diastereoselectivity in the arylation of valine-derived bis-lactim ethers (Fig. 3C). XPhos exhibits a very rigid secondary interaction with its biaryl substituent, while a more flexible and maybe stronger interaction would be necessary for achieving high diastereoselectivity. Using our model, we predicted the diastereoselectivity for the morpholyl-substituted ^Mor^adYPhos ligand, which we were unable to synthesize (see above). Compared to the 14 : 1 predicted selectivity for ^Pip^adYPhos (exp. 15 : 1) the ^Mor^adYPhos ligand is expected to achieve an improved 17 : 1 selectivity. Therefore, developing an alternative synthetic route for this ligand would be highly desirable in the future.

## Conclusions

In conclusion, we found that *N*-coordinating ylide functionalized phosphines (NYPhos) exhibit unique selectivity in the diastereoselective arylation of commercially available valine-derived Schöllkopf bis-lactim ethers with aryl chlorides. An optimized NYPhos ligand enabled high chemo- and diastereoselectivities in the coupling of aryl and alkenyl chlorides, as well as in the late-stage functionalization of common drug molecules, obtaining the desired arylated bis-lactim ethers in up to 97% yield and with a >25 : 1 dr. Aqueous hydrolytic cleavage afforded the desired enantiopure arylglycines in up to 96% ee, which are common structural motifs present in many pharmaceutically relevant compounds. Computational studies elucidated the precise mechanism of the transformation, showing that the rate-limiting step varies depending on the phosphine ligands used. The correlation between diastereoselectivity and ligand properties identified the nature of the secondary metal–ligand interaction as a key factor in determining the selectivity of the arylation of bis-lactim ethers.

## Notes

Pd-methylnaphthyl complexes are part of a patent filed by Umicore AG & Co. KG in 2021. The authors have filed patent WO2019030304 in collaboration with UMICORE AG & Co. KG, covering YPhos ligands and complexes. The authors have filed patent EP 23158954.0 in collaboration with UMICORE AG & Co. KG, covering the NYPhos ligands, their complexes and application.

## Author contributions

D. S. P.: methodology, investigation, project administration, writing – original draft, writing – review and editing; J. L.: computational studies, investigation, writing – original draft, I. M. B.: investigation, validation; N. S.: methodology, investigation; H. R.: computational studies; R. N. de O.: supervision; V. H. G.: supervision, resources, writing – review and editing; L. J. G.: supervision, resources, writing – review and editing.

## Conflicts of interest

There are no conflicts to declare.

## Supplementary Material

SC-OLF-D5SC02814K-s001

SC-OLF-D5SC02814K-s002

SC-OLF-D5SC02814K-s003

SC-OLF-D5SC02814K-s004

SC-OLF-D5SC02814K-s005

SC-OLF-D5SC02814K-s006

## Data Availability

The data supporting this article have been included as part of the ESI.† Experimental data for this article, including analytical spectra are available at sciflection at https://identifiers.org/sciflection:75802b9b-8785-46cb-b6e2-3a9080df3cd7.
