# Supplementary material for: Diastereoselective arylation of bis-lactim ethers catalyzed by N-coordinating ylide-functionalized phosphine (NYPhos)
Source: Chem Sci. 2025 Jun 30;16(31):14152–60. doi: 10.1039/d5sc02814k (PMC12230854; doi:10.1039/d5sc02814k)
Supplement: SC-016-D5SC02814K-s002 [file SC-016-D5SC02814K-s002.pdf]

This journal is © The Royal Society of Chemistry 2025

In [ ]:
```
import numpy as np
import matplotlib.pyplot as plt
import seaborn as sns
import pandas as pd

from sklearn import metrics
from sklearn.preprocessing import StandardScaler
from sklearn.linear_model import LinearRegression
from sklearn.model_selection import RepeatedKFold,LeaveOneOut

import statsmodels.api as sm

import warnings
warnings.filterwarnings("ignore")

randomstate = 42
temp = 298.15
R = 0.00198588

colors = {"CP_darkblue": '#0c4383',
"CP_lightblue": "#22a0b6",
"CP_türkis": "#46E0A6",
"CP_darkred": '#7b1346',
"CP_lightred": '#cb0c59',
"CP_orange": "#ed8554",
"CP_green":"#029645",
"CP_yellow":"#c8b029"}
```

In [ ]:
```
# Metric functions

def r2_val(y_test,y_pred_test,y_train):
    """Calculates the external R2 pred as described:
    https://
pdfs.semanticscholar.org/4eb2/5ff5a87f2fd6789c5b9954eddddfd1c59da
    y_resid = y_pred_test - y_test
    SS_resid = np.sum(y_resid**2)
    y_var = y_test - np.mean(y_train)
    SS_total = np.sum(y_var**2)
    r2_validation = 1-SS_resid/SS_total
    return(r2_validation)

### LeaveOneOut



def get_q2(X,y,model=LinearRegression()):

    loo = LeaveOneOut()
    ytests = []
    ypreds = []
    for train_idx, test_idx in loo.split(X):
        X_train, X_test = X[train_idx], X[test_idx]
        y_train, y_test = y[train_idx], y[test_idx]

        model.fit(X_train,y_train)
        y_pred = model.predict(X_test)

        ytests += list(y_test)
        ypreds += list(y_pred)

    rr = metrics.r2_score(ytests, ypreds)
    return(rr,ypreds)



def repeated_k_fold(X_train,y_train,reg = LinearRegression(),
k=5, n=100):
    """Reapeated k-fold cross-validation.
    For each of n repeats, the (training)data is split into k
```

```
[illegible]
folds.
    For each fold, this part of the data is predicted using the
rest.
    Once this is done for all k folds, the coefficient of
determination (R^2) of the predictions of all folds combined (=
the complete data set) is evaluated
    This is repeated n times and all n R^2 are returned for
averaging/further analysis
    """

    rkf = RepeatedKFold(n_splits=k, n_repeats=n)
    r2_scores = []
    y_validations,y_predictions = np.zeros((np.shape(X_train)
[0],n)),np.zeros((np.shape(X_train)[0],n))
    foldcount = 0
    for i,foldsplit in enumerate(rkf.split(X_train)):
        fold, rep = i%k, int(i/k) # Which of k folds. Which of n
repeats
        model =
reg.fit(X_train[foldsplit[0]],y_train[foldsplit[0]]) #
foldsplit[0]: k-1 training folds
        y_validations[foldcount:foldcount+len(foldsplit[1]),rep]
= y_train[foldsplit[1]] # foldsplit[1]: validation fold
        y_predictions[foldcount:foldcount+len(foldsplit[1]),rep]
= model.predict(X_train[foldsplit[1]])
        foldcount += len(foldsplit[1])
        if fold+1==k:
            foldcount = 0
    r2_scores =
np.asarray([metrics.r2_score(y_validations[:,rep],y_predictions[:
for rep in range(n)])
    return(r2_scores)



def kennardstonealgorithm( X, k ):
    """
    @author: Hiromasa Kaneko
    https://github.com/hkaneko1985/kennardstonealgorithm
    --- input ---
    X : dataset of X-variables (samples x variables)
```

```
    k : number of samples to be selected

    --- output ---
    selectedsamplenumbers : selected sample numbers (training data)
    remainingsamplenumbers : remaining sample numbers (test data)
    """
    X = np.array( X )
    originalX = X
    distancetoaverage = ( (X - np.tile(X.mean(axis=0), (X.shape[0], 1) ) )**2 ).sum(axis=1)
    maxdistancesamplenumber = np.where( distancetoaverage == np.max(distancetoaverage) )
    maxdistancesamplenumber = maxdistancesamplenumber[0][0]
    selectedsamplenumbers = list()
    selectedsamplenumbers.append(maxdistancesamplenumber)
    remainingsamplenumbers = np.arange( 0, X.shape[0], 1)
    X = np.delete( X, selectedsamplenumbers, 0)
    remainingsamplenumbers = np.delete( remainingsamplenumbers, selectedsamplenumbers, 0)
    for iteration in range(1, k):
        selectedsamples = originalX[selectedsamplenumbers,:]
        mindistancetoselectedsamples = list()
        for mindistancecalculationnumber in range( 0, X.shape[0]):
            distancetoselectedsamples = ( (selectedsamples - np.tile(X[mindistancecalculationnumber,:], (selectedsamples.shape[0], 1)) )**2 ).sum(axis=1)
            mindistancetoselectedsamples.append( np.min(distancetoselectedsamples) )
        maxdistancesamplenumber = np.where( mindistancetoselectedsamples == np.max(mindistancetoselectedsamples) )
        maxdistancesamplenumber = maxdistancesamplenumber[0][0]
        
selectedsamplenumbers.append(remainingsamplenumbers[maxdistancesa
        X = np.delete( X, maxdistancesamplenumber, 0)
        remainingsamplenumbers = np.delete( remainingsamplenumbers, maxdistancesamplenumber, 0)
```

```
    return(selectedsamplenumbers, remainingsamplenumbers)
```

Selectivity and Error Propagation Formulas

The **ChemoSelectivity** is calculated as:

$$\text{ChemoSelectivity} = \frac{\text{anti} + \text{syn}}{\text{conversion} - \text{anti} - \text{syn}}$$

The error in **ChemoSelectivity** is calculated as:

$$\sigma_{\text{ChemoSelectivity}} = \text{ChemoSelectivity} \cdot \sqrt{\left(\frac{\sigma_{\text{numerator}}}{\text{numerator}}\right)^2 + \left(\frac{\sigma_{\text{denominator}}}{\text{denominator}}\right)^2}$$

where:

$$\sigma_{\text{numerator}} = \sqrt{\sigma_{\text{anti}}^2 + \sigma_{\text{syn}}^2}, \quad \sigma_{\text{denominator}} = \sqrt{\sigma_{\text{conversion}}^2 + \sigma_{\text{anti}}^2 + \sigma_{\text{syn}}^2}$$

The **DiastereoSelectivity** is given by:

$$\text{DiastereoSelectivity} = \frac{\text{anti}}{\text{syn}}$$

The error propagation for **DiastereoSelectivity** is:

$$\sigma_{\text{DiastereoSelectivity}} = \text{DiastereoSelectivity} \cdot \sqrt{\left(\frac{\sigma_{\text{anti}}}{\text{anti}}\right)^2 + \left(\frac{\sigma_{\text{syn}}}{\text{syn}}\right)^2}$$

For the ($\Delta \, \Delta$ G), we use:

$$\Delta \, \Delta \text{ G} = -RT \cdot \ln(\text{Selectivity})$$

The propagated error in ($\Delta \, \Delta$ G) is:

$$\sigma_{\Delta \, \Delta \text{ G}} = RT \cdot \frac{\sigma_{\text{Selectivity}}}{\text{Selectivity}}$$

In [3]:
```
# Load Datasets
df_X1 =
pd.read_csv("bi_gen_red_filt.csv").set_index("Lig_ID",drop=True)
df_X2 =
pd.read_csv("bi_bi_red_filt.csv").set_index("Lig_ID",drop=True)
df_X = pd.merge(df_X1, df_X2,how='inner',
on='Lig_ID').drop(['V_min_boltz','V_min_r_boltz'],axis = 1)
df_ext = df_X.loc["LYn50"] # Not synthesizeable MoradYPhos
Ligand
df_val = df_X.loc["LBn04"] # External Validation (RockPhos)
df_X = df_X.drop(["LYn50","LBn04"],axis = 0)
X = np.asarray(df_X)

df_y = pd.read_csv("targets.csv").set_index("Lig_ID",drop=True)
df_y = df_y[df_y.index.notnull()]
df_y["conversion"] = 100 - df_y["arcl"]

# Assuming all GC errors in raw values are +- 2
error_value = 2

sigma_num_chemo = np.sqrt(error_value**2 + error_value**2)  #
error in (product_anti + product_syn)
sigma_den_chemo = np.sqrt(error_value**2 + error_value**2 +
error_value**2)  # error in (conversion - product_anti -
product_syn)
num_chemo = df_y["anti"] + df_y["syn"]
den_chemo = df_y["conversion"] - df_y["anti"] - df_y["syn"]

df_y["ChemoSelectivity"] = (df_y["anti"] + df_y["syn"]) /
(df_y["conversion"] - df_y["anti"] - df_y["syn"])
df_y["ChemoSelectivity_error"] = df_y["ChemoSelectivity"] *
np.sqrt((sigma_num_chemo / num_chemo)**2 + (sigma_den_chemo /
den_chemo)**2)

df_y["DiastereoSelectivity"] = df_y["anti"] / df_y["syn"]
df_y["DiastereoSelectivity_error"] =
df_y["DiastereoSelectivity"] * np.sqrt((error_value /
df_y["anti"])**2 + (error_value / df_y["syn"])**2)

df_y["ddG_chemo"] = -1 * temp * R *
np.log(df_y["ChemoSelectivity"])
```

```
[illegible]
df_y["ddG_chemo_error"] = R * temp *
(df_y["ChemoSelectivity_error"] / df_y["ChemoSelectivity"])

df_y["ddG_dr"] = -1 * temp * R *
np.log(df_y["DiastereoSelectivity"])
df_y["ddG_dr_error"] = R * temp *
(df_y["DiastereoSelectivity_error"] /
df_y["DiastereoSelectivity"])
```

In [4]:

```
hues = {
    "NYPhos":colors["CP_darkblue"],
    "YPhos":colors["CP_lightred"],
    "Buchwald":colors["CP_green"],
    "Trialkyl":colors["CP_lightblue"]}

markers = ['o', 's', '^', 'v']

# Plor dr vs chemoselectivity
fig,ax = plt.subplots(figsize=(4, 3.6))
sns.scatterplot(data=df_y.drop(["LR12"],axis=0), x="ddG_chemo",
y="ddG_dr", s=70, style="Type", hue="Type",
                palette=hues, markers=markers).set(xlabel = "-
RT*ln(S)",ylabel = "-RT*ln(dr)")

plt.legend()
plt.tight_layout()
plt.show()
```

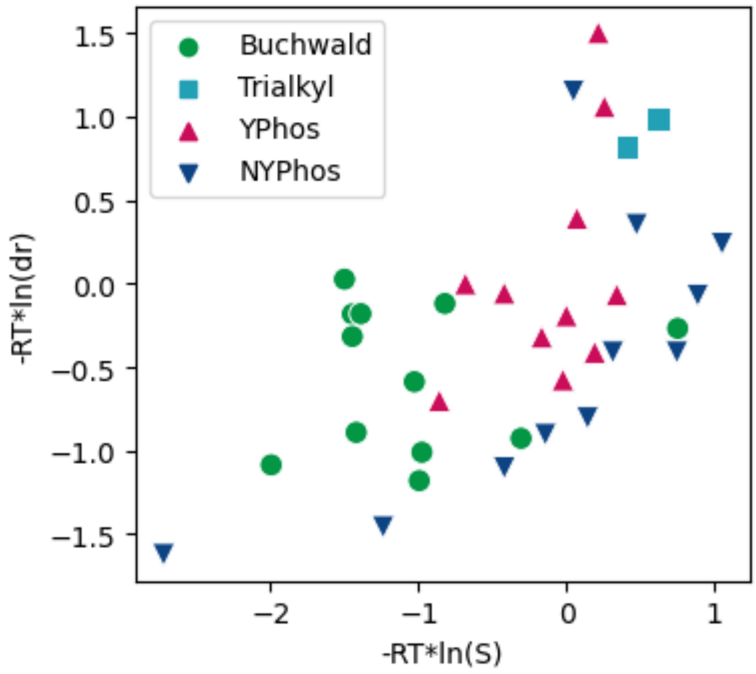

In [5]:
```
df_y.head()
```

Out[5]:

	Ligand	Type	anti	syn	dehalo	arom	arcl	conversion	ChemoSelectivity	Ch
**Lig_ID**										
**LB04**	XPhos	Buchwald	53	39	0	4	0	100	11.500000	
**LB14**	BrettPhos	Buchwald	58	34	0	3	0	100	11.500000	
**LB12**	tBu-BrettPhos	Buchwald	44	8	9	0	38	62	5.200000	
**LB11**	tBu-XPhos	Buchwald	62	23	4	0	0	100	5.666667	
**LB06**	RuPhos	Buchwald	43	45	6	0	5	95	12.571429	

Diastereoselectivity

In [6]:
```
# Filter
df_y_dr = df_y[~df_y['ddG_dr'].isin([np.nan, np.inf, -np.inf])]

print(f'ddG ranges from {df_y_dr["ddG_dr"].min():.3} to
{df_y_dr["ddG_dr"].max():.3} kcal/mol')
print(f'Range =
{(abs(df_y_dr["ddG_dr"].min())+abs(df_y_dr["ddG_dr"].max())):.3}
kcal/mol')
print(f'ddG Errors range from {df_y_dr["ddG_dr_error"].min():.3}
to {df_y_dr["ddG_dr_error"].max():.3} kcal/mol')
print(f'Average Error = {df_y_dr["ddG_dr_error"].mean():.3}
kcal/mol')
```

```
ddG ranges from -1.62 to 1.5 kcal/mol
Range = 3.13 kcal/mol
ddG Errors range from 0.0377 to 0.402 kcal/mol
Average Error = 0.143 kcal/mol
```

In [7]:

```
# Plotting the error distribution
plt.figure(figsize=(4, 4))
plt.hist(df_y_dr['ddG_dr_error'],
bins=15,color=colors["CP_darkblue"], edgecolor='black')
plt.xlabel('Error')
plt.ylabel('Frequency')
plt.title('Distribution of Errors')
plt.show()
```

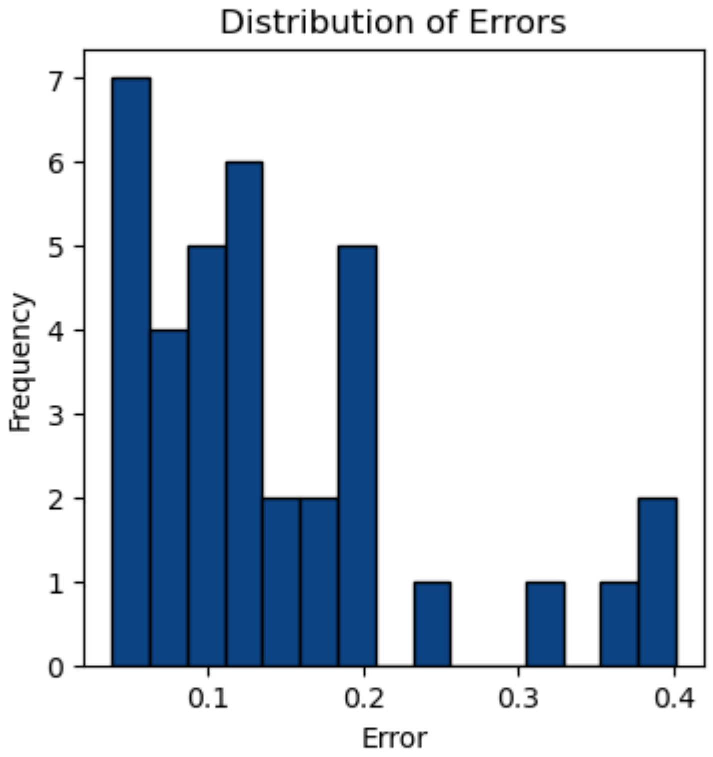

In [8]:
```
y_target = "ddG_dr"
y_target_errors = "ddG_dr_error"

# prepare targets
y_exp = pd.to_numeric(df_y_dr[y_target].dropna(),
errors='coerce')
y_errors = pd.to_numeric(df_y_dr[y_target_errors].dropna(),
errors='coerce')

y = np.asarray([float(i) for i in
np.asarray(y_exp).reshape(-1)])
y_err = np.asarray([float(i) for i in
np.asarray(y_errors).reshape(-1)])
y_val = df_y_dr[y_target].loc["LBn04"]

df_y_dr.drop(["LBn04"],axis=0,inplace=True)

df_X = df_X.loc[df_y_dr[y_target].dropna().index,:]
df_X = df_X.drop([i for i in df_X.columns if
(len(df_X[i].unique()) == 1 or len(df_X[i].unique()) == 0)],
axis = "columns")

X = np.asarray(df_X)
X_ext = np.asarray(df_ext).reshape(1, -1)
X_val = np.asarray(df_val).reshape(1, -1)

df_X_y = pd.concat([df_X,df_y_dr],axis=1)

fig,ax = plt.subplots(figsize=(4,4))
plt.hist(y_exp, bins=10, color=colors["CP_darkblue"],
edgecolor='black')
plt.xlabel(y_target)
plt.ylabel("N samples")
plt.title("Target Distribution")
plt.show()
```

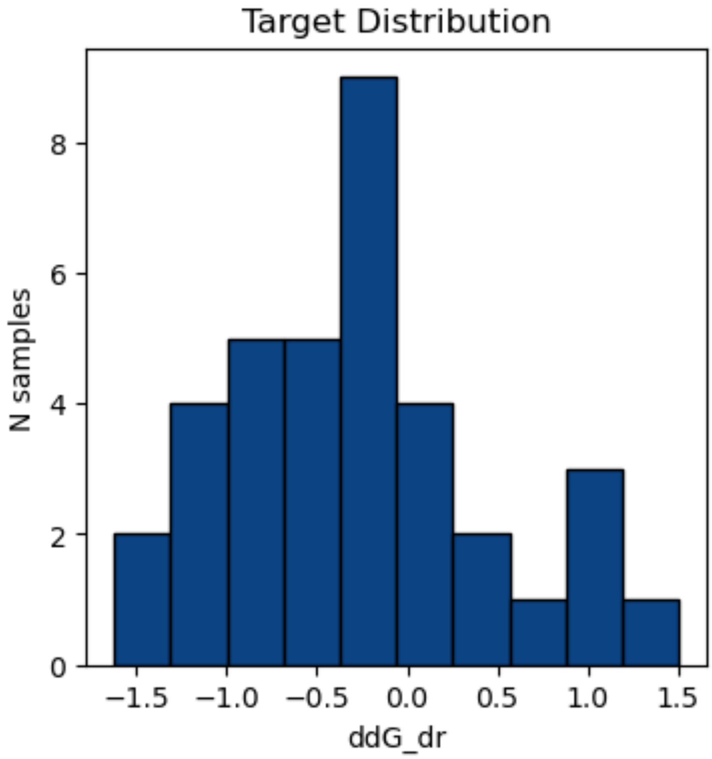
Target Distribution
N samples
ddG_dr
0
2
4
6
8
−1.5
−1.0
−0.5
0.0
0.5
1.0
1.5

In [ ]:
```
# Split Data according to KS Algorithm
X_sel,y_sel,labels_sel = X.astype(float),y,df_y_dr.index
rs = 42
test_ratio = 0.8

VS,TS = kennardstonealgorithm(X_sel,int((1-
test_ratio)*np.shape(X_sel)[0]))

X_train, y_train, y_train_err = X[TS], y[TS], y_err[TS]
X_test, y_test, y_test_err = X[VS], y[VS], y_err[VS]

print("TS: {}".format(([df_X.index[i] for i in TS])))
print("VS: {}".format(([df_X.index[i] for i in VS])))
print("y_mean TS: {:.3f}".format(np.mean(y_train)))
print("y_mean VS: {:.3f}".format(np.mean(y_test)))
print("Shape X_train: {}".format(X_train.shape))
print("Shape X_test:  {}".format(X_test.shape))
fig,ax = plt.subplots(figsize=(4, 4))
hist,bins = np.histogram(y_sel,bins=10)
plt.hist(y_train, bins, alpha=1,
label='y_train',color=colors["CP_darkblue"], edgecolor='black')
plt.hist(y_test, bins, alpha=1, label='y_test',
color=colors["CP_lightred"], edgecolor='black')
plt.legend(loc='best')
plt.xlabel("target")
plt.ylabel("N samples")
plt.show()

# Scale Data
scaler = StandardScaler()
X_train_sc = scaler.fit_transform(X_train)
X_test_sc = scaler.transform(X_test)
X_ext_sc = scaler.transform(X_ext)
X_val_sc = scaler.transform(X_val)
```

```
TS: ['LB04', 'LB14', 'LB12', 'LB11', 'LB06', 'LY02', 'LY01', 'LY03', 'LY0
5', 'LY12', 'LY18', 'LY13', 'LY14', 'LY08', 'LYn34', 'LYn37', 'LYn30', 'L
Y21', 'LYn43', 'LYn29', 'LYn28', 'LYn32', 'LY22', 'LB08', 'LY04', 'LA11',
'LYn33', 'LB03', 'LBn02']
VS: ['LB07', 'LYn44', 'LB05', 'LY20', 'LA01']
y_mean TS: -0.306
y_mean VS: 0.151
Shape X_train: (29, 194)
Shape X_test:  (5, 194)
```

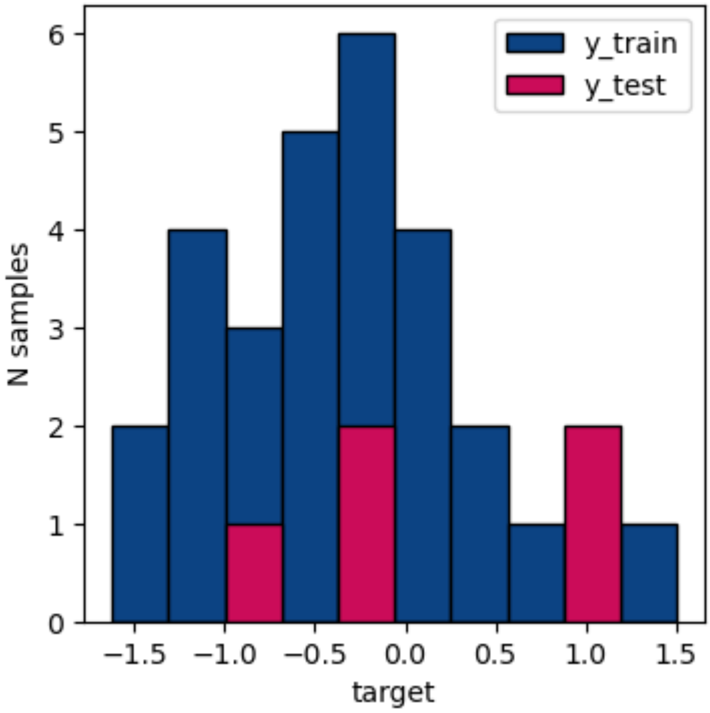

Model

In [10]:
```
features_names =
["d_Pd_P_boltz","nuesp_X_boltz","qvbur_full_delta_NENW_boltz","st
features_py = sorted([list(df_X.columns).index(i) for i in
features_names])
features_py = sorted(features_py)
X_train_sel = X_train_sc[:,features_py]
X_test_sel = X_test_sc[:,features_py]
X_ext_sel = X_ext_sc[:,features_py]
X_val_sel = X_val_sc[:,features_py]

lr = LinearRegression().fit(X_train_sel, y_train)

y_pred_train = lr.predict(X_train_sel)
y_pred_test =  lr.predict(X_test_sel)
y_pred_ext = lr.predict(X_ext_sel)
y_pred_val = lr.predict(X_val_sel)
q2,loo_train = get_q2(X_train_sel,y_train)
kfoldscores = repeated_k_fold(X_train_sel,y_train,k=5,n=200)

print("\nParameters:\n{:10.4f} + \n".format(lr.intercept_) +
"\n".join(["{:10.4f} *
{}".format(lr.coef_[i],df_X.columns[sorted(features_py)[i]]) for
i in range(len(features_py))]))
print(f"\nTraining R2  = {lr.score(X_train_sel,
y_train):.3f}\nTraining Q2  = {q2:.3f}")
print(f"Training MAE =
{metrics.mean_absolute_error(y_train,y_pred_train):.3f}")
print("Training K-fold R2 = {:.3f} (+/-
{:.3f})".format(kfoldscores.mean(), kfoldscores.std() ** 2))
print(f"\nTest R2      =
{r2_val(y_test,y_pred_test,y_train):.3f}\nTest MAE     =
{metrics.mean_absolute_error(y_test,y_pred_test):.3f}")
print(f"\nTraining/Test: {y_train.shape[0]}/{y_test.shape[0]}")

model = sm.OLS(y_train,
sm.add_constant(pd.DataFrame(X_train_sel))).fit()
print(model.summary())
```

```
Parameters:
   -0.3056 +
   -0.4621 * d_Pd_P_boltz
   -0.1486 * qvbur_full_delta_NENW_boltz
    0.4598 * nuesp_X_boltz
   -0.2413 * sterimol_B1_X_delta

Training R2  = 0.817
Training Q2  = 0.739
Training MAE = 0.240
Training K-fold R2 = 0.723 (+/- 0.001)

Test R2      = 0.720
Test MAE     = 0.431

Training/Test: 29/5
                            OLS Regression Results
==========================================================================
Dep. Variable:                      y   R-squared:
Model:                            OLS   Adj. R-squared:
Method:                 Least Squares   F-statistic:
Date:                Tue, 10 Jun 2025   Prob (F-statistic):           1.5
Time:                        17:34:26   Log-Likelihood:                -6
No. Observations:                  29   AIC:
Df Residuals:                      24   BIC:
Df Model:                           4
Covariance Type:            nonrobust
==========================================================================
                 coef    std err          t      P>|t|      [0.025      0
--------------------------------------------------------------------------
const         -0.3056      0.062     -4.931      0.000      -0.433      -
0             -0.4621      0.063     -7.388      0.000      -0.591      -
1             -0.1486      0.070     -2.129      0.044      -0.293      -
2              0.4598      0.074      6.175      0.000       0.306
3             -0.2413      0.068     -3.551      0.002      -0.382      -
==========================================================================
Omnibus:                        2.484   Durbin-Watson:
Prob(Omnibus):                  0.289   Jarque-Bera (JB):
Skew:                           0.517   Prob(JB):
Kurtosis:                       3.308   Cond. No.
==========================================================================

Notes:
[1] Standard Errors assume that the covariance matrix of the errors is co
ly specified.
```

Model exploration

In [11]:
```
import scipy.stats as stats
import matplotlib.pyplot as plt

# Normal distribution of residuals
residuals = y_train - y_pred_train
fig,ax = plt.subplots(figsize=(4, 4))
stats.probplot(residuals, dist="norm", plot=ax)
plt.title("Q-Q Plot of Residuals")
plt.show()
```

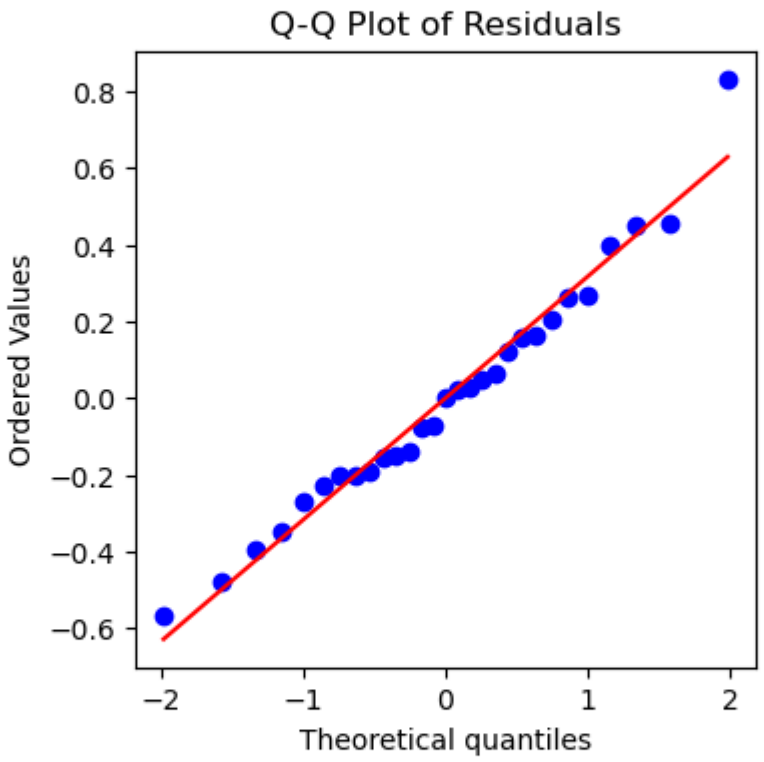

In [12]:
```
# Shapiro-Wilk Test
stat, p = stats.shapiro(residuals)
print(f"Shapiro-Wilk Test Statistic: {stat:.3f}, p-value:
{p:.3f}")
```

```
Shapiro-Wilk Test Statistic: 0.978, p-value: 0.772
```

In [13]:

```
import statsmodels.stats.diagnostic as smd

# Residuals vs. Fitted Values Plot
fig,ax = plt.subplots(figsize=(4, 4))
plt.scatter(y_pred_train, residuals, alpha=0.6)
plt.axhline(0, color="red", linestyle="--")
plt.xlabel("Fitted Values")
plt.ylabel("Residuals")
plt.title("Residuals vs. Fitted Values")
plt.show()
```

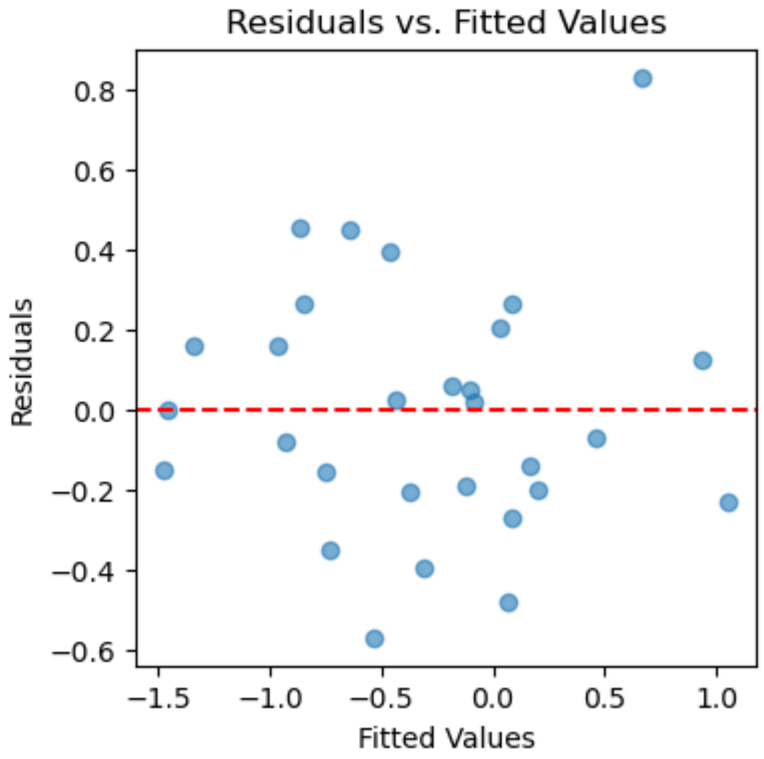

In [14]:

```
# Breusch-Pagan Test
bp_test = smd.het_breuschpagan(residuals,
sm.add_constant(X_train_sel))
print(f"Breusch-Pagan Test Statistic: {bp_test[0]:.3f}, p-value:
{bp_test[1]:.3f}")
```

```
Breusch-Pagan Test Statistic: 2.839, p-value: 0.585
```

In [15]:

```
# correlation matrix of features
new_df = pd.DataFrame()
for i in features_py:
    feature = df_X.columns[i]
    new_df[f"{feature}"] = X_sel[:,i]
new_df.columns = ["x1","x2","x3","x4"]
plt.figure(figsize=(5,3))
sns.heatmap(new_df.corr()**2, annot=True, cmap = "Blues", vmin =
0, vmax = 1)
plt.xticks(rotation=45)
plt.title("R²")
plt.show()
```

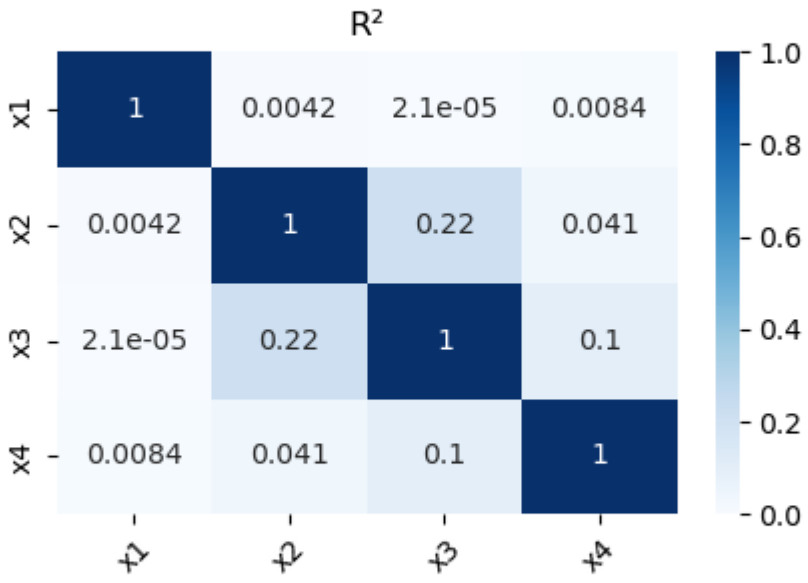

In [16]:

```
plt.figure(figsize=(1,1))
sns.pairplot(data = df_X_y[features_names+[y_target]], size=2)
plt.show()
```

```
<Figure size 100x100 with 0 Axes>
```

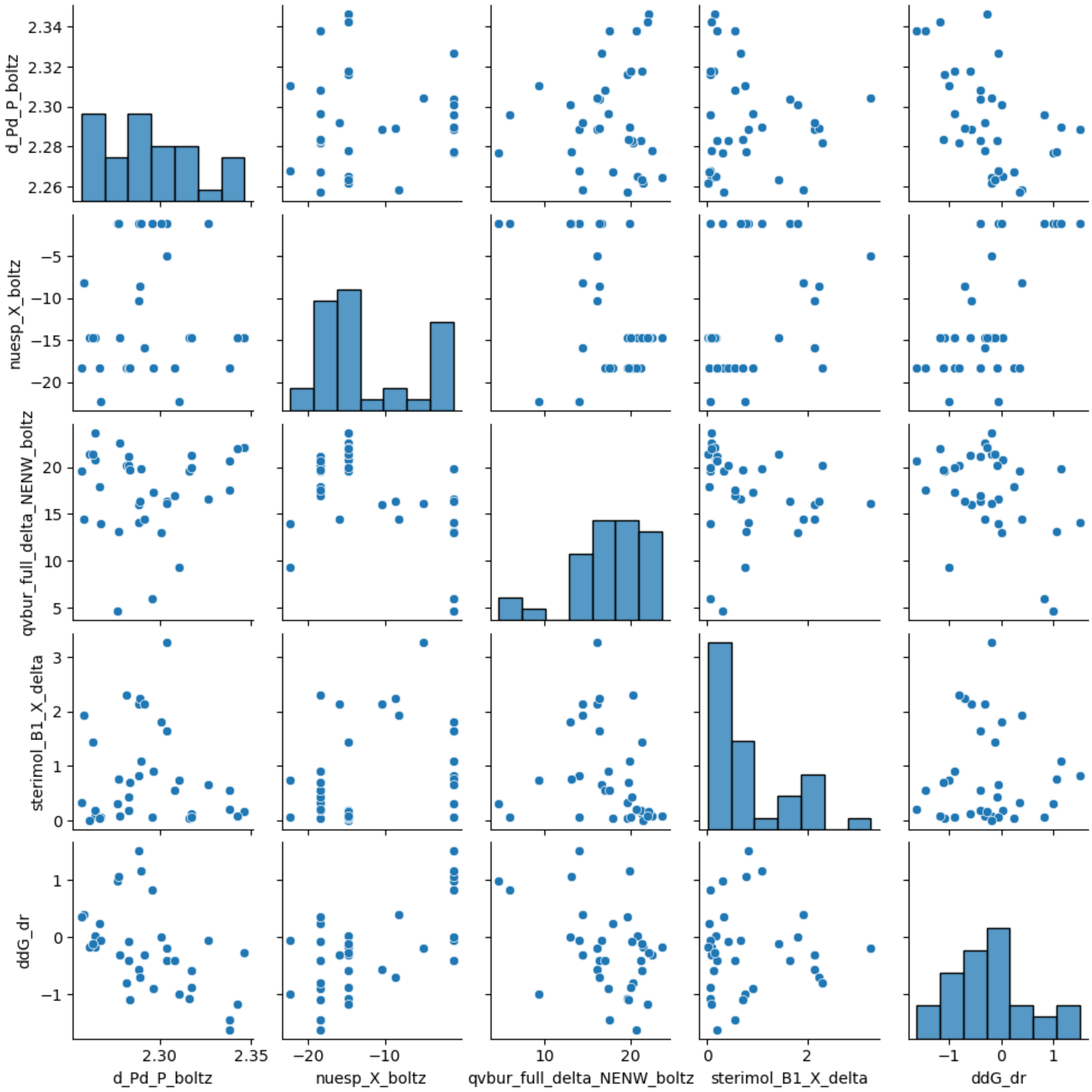

In [17]:

```
# prediction error
# half-width of confidence intervals of predictions using sm
prediction_summary =
model.get_prediction(sm.add_constant(X_test_sel))
confidence_intervals_test = prediction_summary.conf_int()
vertical_errors_test = (confidence_intervals_test[:, 1] -
confidence_intervals_test[:, 0]) / 2

prediction_summary =
model.get_prediction(sm.add_constant(X_train_sel))
confidence_intervals_train = prediction_summary.conf_int()
vertical_errors_train = (confidence_intervals_train[:, 1] -
confidence_intervals_train[:, 0]) / 2
```

In [18]:

```
# final plot
y_orig_min = np.min(np.hstack((y_train,y_test)))
y_pred_min = np.min(np.hstack((y_pred_train,y_pred_test)))
y_orig_max = np.max(np.hstack((y_train,y_test)))
y_pred_max = np.max(np.hstack((y_pred_train,y_pred_test)))
delta_x = 0.15 * (y_orig_max-y_orig_min)
delta_y = 0.15 * (y_pred_max-y_pred_min)

yy_fit = np.polyfit(y_train,y_pred_train,deg=1)
yy_fit_line = yy_fit[1]+yy_fit[0]*y_train

plt.figure(figsize=(5,5))
plt.plot(np.linspace(y_orig_min-delta_x,y_orig_max+delta_x),
         np.linspace(y_orig_min-
delta_x,y_orig_max+delta_x),color="grey",alpha=0.5)
plt.plot(sorted(y_train),sorted(yy_fit_line),color="black",alpha=

plt.xlim([y_orig_min-delta_x,y_orig_max+delta_x])
plt.ylim([y_pred_min-delta_y,y_pred_max+delta_y])

plt.errorbar(y_train, y_pred_train, xerr= y_train_err,
fmt="none",color=colors["CP_darkblue"], capsize=0,
linestyle="none", alpha = 0.25)
plt.errorbar(y_test, y_pred_test, xerr= y_test_err,
fmt="none",color=colors["CP_lightred"], capsize=0,
linestyle="none", alpha = 0.25)

plt.errorbar(y_test, y_pred_test, yerr=vertical_errors_test,
fmt="none", ecolor=colors["CP_lightred"], alpha=0.25, capsize=0)
plt.errorbar(y_train, y_pred_train, yerr=vertical_errors_train,
fmt="none", ecolor=colors["CP_darkblue"], alpha=0.25, capsize=0)


plt.scatter(y_train,loo_train,label="LOO",color=colors["CP_darkbl
plt.scatter(y_train,y_pred_train,label="Train",color=colors["CP_d
plt.scatter(y_test,y_pred_test,label="Test",color=colors["CP_ligh
= colors["CP_darkred"],marker="D",s=30)

plt.scatter(y_val,
y_pred_val,label="Val.",color=colors["CP_türkis"],edgecolors="bla
marker="D", s =35)
```

```
plt.scatter(y_pred_ext,y_pred_ext,label="Pred.", color=
colors["CP_green"],edgecolors="black", marker="D", s =35)


textstr = '\n'.join((
    f"Train R\u00b2 = {lr.score(X_train_sel, y_train):.2f}",
    f"Train Q\u00b2  = {q2:.2f}",
    f"Train MAE =
{metrics.mean_absolute_error(y_train,y_pred_train):.2f}",
    f"k-fold R\u00b2 = {round(kfoldscores.mean(),2)}",#\u00B1
{round(kfold_all.std()**2,3)}"
    f"Test R\u00b2 = {r2_val(y_test,y_pred_test,y_train):.2f}",
    f"Test MAE =
{metrics.mean_absolute_error(y_test,y_pred_test):.2f}"
    ))

props = dict(boxstyle='round', facecolor='wheat', alpha=0.5)
plt.text(0.12, 1.25, textstr, transform=ax.transAxes,
fontsize=11,
        verticalalignment='top', bbox=props)

label = "-RT*ln(dr)"
plt.legend(loc='lower right')
plt.xlabel(label+" measured",fontsize=11)
plt.ylabel(label+" predicted",fontsize=11)
plt.show()
```

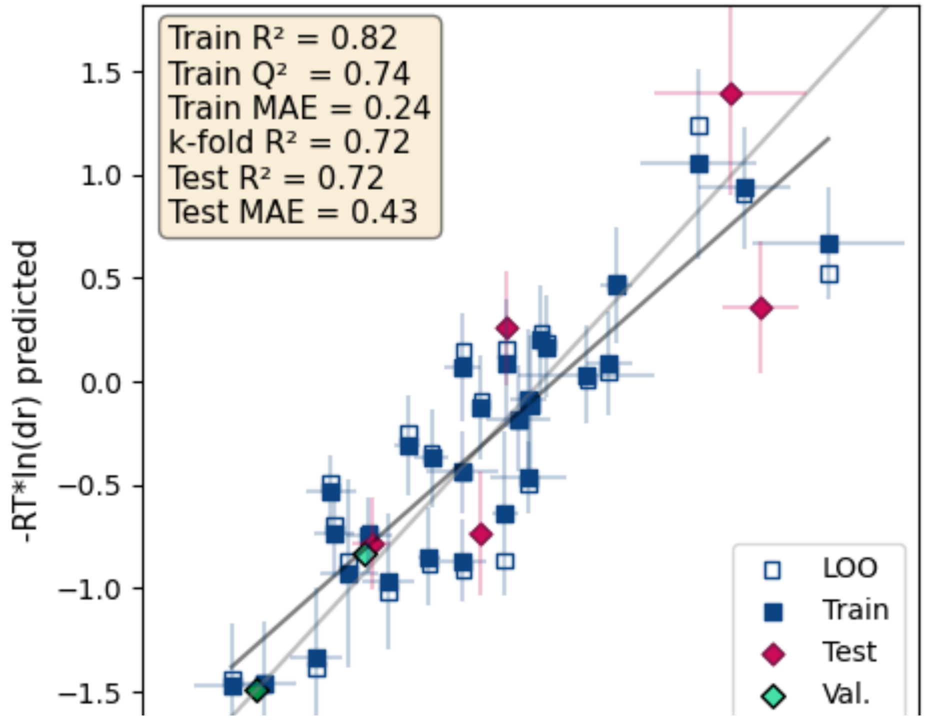

In [19]:
```
# dr Prediction of MoradYPhos Ligand
pred_dr = np.exp(-1*y_pred_ext[0]/(temp*R))
print(pred_dr)
```

```
12.402109017926
```
