# Supplementary material for: Diastereoselective arylation of bis-lactim ethers catalyzed by N-coordinating ylide-functionalized phosphine (NYPhos)
Source: Chem Sci. 2025 Jun 30;16(31):14152–60. doi: 10.1039/d5sc02814k (PMC12230854; doi:10.1039/d5sc02814k)
Supplement: SC-016-D5SC02814K-s003 [file SC-016-D5SC02814K-s003.pdf]

Supplementary Information (SI) for Chemical Science.
This journal is © The Royal Society of Chemistry 2025

Supporting Information

Diastereoselective Arylation of Bis-Lactim Ethers Catalyzed by *N*-coordinating Ylide-Functionalized Phosphine (NYPhos)

Daniel Sowa Prendes[a], Julian Löffler[‡b], Ivan Martins Barreto[‡a,c], Nagesh Sankaran[a], Ronaldo Nascimento de Oliveira[c], Viktoria H. Gessner*[b], Lukas J. Gooßen*[a]

[a]*Fakultät Chemie und Biochemie, Organische Chemie I, Ruhr-Universität Bochum, Universitätsstr. 150, 44801 Bochum, Germany*

[b]*Fakultät Chemie und Biochemie, Anorganische Chemie II, Ruhr-Universität Bochum, Universitätsstr. 150, 44801 Bochum, Germany*

[c]*Laboratory of Synthesis of Bioactive Compounds, Department of Chemistry, Federal Rural University of Pernambuco (UFRPE), Recife 52171-900, Pernambuco, Brazil*

[‡]*These authors contributed equally*

*Email: viktoria.gessner@rub.de; lukas.goossen@rub.de

Index

1. General Methods ... 2
2. General Procedures ... 2
3. Additional Screening Results ... 3
4. Synthesis and Characterization of novel NYPhos Ligands ... 4
5. Synthesis and Characterization of Products ... 5
6. Crystal Structure Determination of (*R,S*)-3aa ... 14
7. Crystal Structure Determination of ^{Pip}trYPhos ... 16
8. Crystal Structure Determination of ^{Pip}AdYPhos ... 19
9. Competition Experiments ... 24
10. Stereochemistry for Compound 3ea ... 31
11. NMR-Spectra of novel NYPhos Ligands ... 33
12. NMR-Spectra of Products ... 36
13. Computational and statistical studies ... 72
14. References ... 119

1. General Methods

All reactions were performed in oven-dried glassware containing a Teflon-coated stirring bar and dry septum under argon atmosphere. Optimization reactions were monitored by GC analysis using *n*-hexadecane as internal standard. ^{1}H and $^{13}C\{^{1}H\}$ NMR spectra were recorded on an Avance-III-300 or Avance-Neo-400 spectrometer at 25 °C if not stated otherwise. ^{19}F NMR spectra were recorded on Spinsolve Benchtop NMR (MAGRITEK) spectrometers at 25 °C. All values of the chemical shift are in ppm regarding the ***δ***-scale. To display multiplicities and signal forms correctly the following abbreviations were used: s = singlet, d = doublet, t = triplet, q = quartet, quin = quintet, m = multiplet, dd = doublet of doublets, sptd = septet of doublets, br = broad signal. GC analyses were carried out using an HP-5 capillary column (Phenyl methyl siloxane, 30 m × 320 × 0.25, 100/2.3-30-300/3) using the following conditions: Carrier gas: N_2; Initial Temperature: 60 °C; Final Temperature: 300 °C; Rate: 30 $°C·min^{-1}$; Hold Time: 8 min; Column Flow: 1.0 mL min^{-1}; Detector: FID, Temperature FID: 330 °C. Column chromatography was performed on a Pure C-815 Flash (Büchi) using Reveleris packed columns (12 g or 40 g). Mass spectrometric data were acquired on a GC-MS Agilent 5977B MSD. HRMS analyses were acquired using a GC-MS system consisting of an Agilent 7250 GC/Q-TOF, in which ionization was achieved by EI, or an HPLC-MS system consisting of an UltiMate 3000 HPLC (ThermoFisher) with an EC 4/2 Universal RP pre-column (Macherey-Nagel), eluting at 40 °C with acetonitrile/water/formic acid (3:1:0.01) and a flow rate of 0.3 mL/min, coupled with a maXisCompact high-resolution mass spectrometer (Bruker Daltonik GmbH). The MS ionization was achieved by ESI. Chiral HPLC was performed on a Shimadzu LC-20AD using a chiral column (Chiralpak® IA, Daicel™, 25 cm × 4.6 mm, 5 µm, *n*-hexane: isopropanol 95:5 or 97.5:2.5, 0.5 mL/min). Infrared spectra were recorded on Bruker Vertex 70 Spectrometer with Universal ATR Sampling Accessory. Melting points were measured on a Mettler Toledo MP70. Commercial substrates, among others (*S*)-2,5-Dihydro-3,6-dimethoxy-2-isopropylpyrazine, were used as received unless otherwise stated. Solvents were purchased (puriss p.A.) from commercial suppliers and dried by standard procedures.[1] 2-MeTHF was dried over 3 Å activated molecular sieves. The amount of water in the solvent was regularly checked by Karl-Fischer titration. All solvents and liquid reactants were degassed by Argon purge prior to use. $[Pd(1\text{-}MeNAP)Br]_2$ as well as other Pd sources were donated by Umicore. Phosphonium salts (including **1-H₂**), YPhos and NYPhos ligands were synthesized by the Gessner group according to literature procedures.[2–4]

2. General Procedures

<u>General procedure for the arylation of bis-lactim ethers (Procedure A)</u>

An oven-dried vial (20 mL) was charged with $[Pd(1\text{-}MeNAP)Br]_2$ (8.19 mg, 0.0125 mmol, 2.5 mol%) and ^{Pip}AdYPhos (15.3 mg, 0.025 mmol, 5 mol%) in a nitrogen filled glovebox. To this reaction vial, 2-MeTHF (4.0 mL) and the aryl chloride (0.50 mmol, 1.00 equiv.) were added. To ensure full deprotonation and zincation of the bis-lactim ether, LiTMP (147 mg, 1.00 mmol, 2.00 equiv.), $ZnCl_2$ (136 mg, 1.00 mmol, 2.00 equiv.) and (*S*)-2,5-Dihydro-3,6-dimethoxy-2-isopropylpyrazine (184 mg, 1.00 mmol, 2.00 equiv.) were prestirred for 16 hours at room temperature using 2-MeTHF (1.0 mL) as solvent. This mixture was added to the vial containing catalyst, solvent, and aryl chloride, which was subsequently stirred for 16 hours at room temperature. Afterwards, the reaction was quenched with MeOH (1 mL), diluted with EtOAc (30 mL), and extracted with $NaHCO_3$ (30 mL), water (30 mL) and brine (30 mL). The combined organic phase was dried over $MgSO_4$ and purified by flash column chromatography (SiO_2, Cy/EtOAc 9.5:0.5 to 9:1 or DCM/MeOH 9.5:0.5 to 9:1) to yield the arylated bis-lactim ether.
Optimization reactions and mechanistic studies were monitored via GC analysis adding *n*-hexadecane (20 µL) as internal standard to a freshly prepared stock solution of 2-MeTHF and the corresponding aryl chloride.

<u>General Procedure for the cleavage of bis-lactim ethers (Procedure B)</u>

0.5 M HCl (1.10 mL, 0.55 mmol, 2.20 equiv.) was added to the arylated bis-lactim ether (0.25 mmol, 1.00 equiv.) in MeCN (1.20 mL), and the mixture was stirred at 0 ºC overnight. Saturated aqueous Na_2CO_3 was added dropwise to pH 8-9 and the mixture was extracted with EtOAc (3 × 5 mL). The combined organic layers were washed with brine (10 mL), dried over $MgSO_4$, filtered, and concentrated in vacuo. The crude material was dried under high vacuum for several hours, and purified via column chromatography (SiO_2, DCM/MeOH/(NH_3)) to yield the desired arylglycine methyl ester.

3. Additional Screening Results

Table S1. Ligand screening.[a]

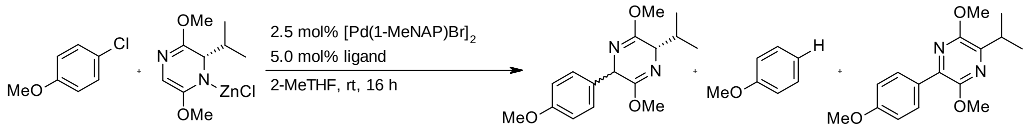

1a **ZnCl-2a** **(*R*,*S*)-3aa** and **(*S*,*S*)-3aa** **4a** **7a**

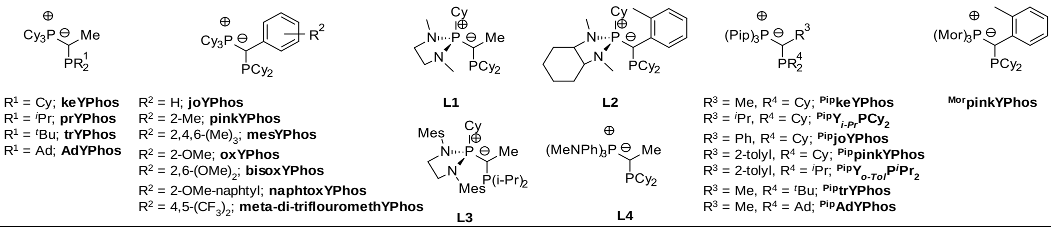

Entry	**Ligand**	**(*R*,*S*)-3aa [%]**	**(*S*,*S*)-3aa [%]**	**4a [%]**	**7a [%]**	**1a [%]**
1	XPhos	53	39	<1	4	<1
2	ᵗBu-XPhos	62	23	4	<1	<1
3	CyJohnPhos	<1	<1	<1	1	98
4	JohnPhos	54	12	5	6[b]	28
5	ᵗBuMePhos	75	12	<1	<1	10
6	BrettPhos	58	34	<1	3	<1
7	ᵗBu-BrettPhos	44	8	9	<1	38
8	AdBrettPhos	66	9	7	<1	11
9	RockPhos	43	10	8	<1	17
10	DavePhos	11	9	<1	2	75
11	SPhos	42	31	9	2	20
12	RuPhos	43	45	6	<1	5
13	AlPhos	11	7	28	<1	18
14	P^tBu_3 x HBF_4	<1	<1	<1	<1	98
15	PAd_3	3	16	50	<1	27
16	$PAd_2{}^nBu$	<1	4	10	<1	83
17	$PAd_2{}^tBu$	4	16	40	<1	40
18	MorDalPhos	54	<1	<1	<1	45
19	keYPhos	3	38	45	3	<1
20	prYPhos	5	30	40	4	11
21	trYPhos	33	30	22	2	6
22	AdYPhos	38	38	23	<1	<1
23	joYPhos	28	14	53	4	<1
24	pinkYPhos	37	14	47	4[b]	<1
25	mesYPhos	62	19	18	3[b]	<1
26	oxYPhos	19	17	61	4	<1
27	bisoxYPhos	16	31	48	3	<1
28	naphtoxYPhos	36	21	39	5[b]	<1
29	meta-di-trifluoromethYPhos	29	21	46	5[b]	<1
30	L1	4	6	55	4	31
31	L2	49	29	18	4	<1
32	L3	6	42	41	3	<1
33	L4	11	20	66	4[b]	<1
34	^{Pip}keYPhos	9	8	72	3	7
35	$^{Pip}Y_{i\text{-}Pr}PCy_2$	22	11	54	3	11
36	^{Pip}joYPhos	14	7	70	3	5
37	^{Pip}pinkYPhos	46	10	43	3	<1
38	$^{Pip}Y_{o\text{-}Tol}P^iPr_2$	35	9	55	3	<1
39	^{Pip}trYPhos	82	7	12	<1	<1
40	^{Pip}adYPhos	93	6	<1	<1	<1
41	^{Mor}pinkYPhos	58	9	30	<1	<1

[a] Conditions: **1a** (0.25 mmol), **ZnCl-2a** (2.0 equiv.), [Pd(1-MeNAP)Br]$_2$ (2.5 mol%), ligand (5.0 mol%), 2-MeTHF (2.0 mL), rt, 16 h; yields determined by GC analysis using *n*-hexadecane as internal standard. [b] signal overlaps with other impurities. **ZnCl-2a** was prepared by pre-stirring bis-lactim ether **2a** (2.0 equiv.), LiTMP (2.0 equiv.) and $ZnCl_2$ (2.0 equiv.) at rt, for 16 h in 2-MeTHF (1.0 mL).

Table S2. Additional screening experiments.[a]

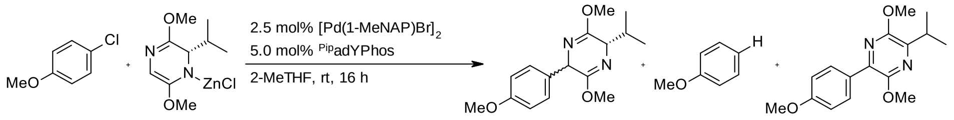

1a ZnCl-2a (R,S)-3aa and (S,S)-3aa 4a 7a

Entry	**Amount of ZnCl-2a**	**(*R*,*S*)-3aa [%]**	**(*S*,*S*)-3aa [%]**	**4a [%]**	**7a [%]**	**1a [%]**
1	2.0 equiv.	93	6	<1	<1	<1
2	1.5 equiv.	58	4	<1	<1	36
3	1.2 equiv.	46	3	<1	<1	50
4	1.0 equiv.	35	3	<1	<1	60

[a] Conditions: **1a** (0.25 mmol), **ZnCl-2a** (2.0 equiv.), $[Pd(1\text{-}MeNAP)Br]_2$ (2.5 mol%), ligand (5.0 mol%), 2-MeTHF (2.0 mL), rt, 16 h; yields determined by GC analysis using *n*-hexadecane as internal standard. **ZnCl-2a** was prepared by pre-stirring bis-lactim ether **2a** (2.0 equiv.), LiTMP (2.0 equiv.) and $ZnCl_2$ (2.0 equiv.) at rt, for 16 h in 2-MeTHF (1.0 mL).

4. Synthesis and Characterization of novel NYPhos Ligands

^{Pip}trYPhos

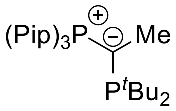

1.92 g (4.36 mmol, 1 eq.) ethyltri(piperidin-1-yl)phosphonium iodide (**1·H₂**) was suspended in THF and deprotonated by slow addition of 2.81 mL of a *n*-BuLi solution (1.55 M in hexane, 4.36 mmol, 1 eq.) until a clear, slightly yellow solution was formed. The solution was stirred for 45 min and then 0.43 mL ditertbutylchlorophosphine (2.18 mmol, 0.5 eq.) was added at room temperature. The solution was stirred for 16 h at reflux, during which time reformed phosphonium salt precipitated as a white solid. The solid was filtered off and the solvent of the solution was removed *in vacuo*. The residue was suspended in 20 mL acetonitrile and stirred overnight, forming a colorless solid. The solid was filtered off, washed with acetonitrile 2 × 15 mL and dried *in vacuo* thus giving **^{Pip}trYPhos** (587 mg, 1.29 mmol, 59%) as a colorless solid.

^{1}H NMR (400 MHz, C_6D_6): δ = 3.04 (q, J_{HH} = 5.1 Hz, 12H, Pip Pos. 1 CH_2), 1.98 (dd, $^3J_{HP}$ = 15.4, 3.0 Hz, 3H, P-C$^-$-CH_3), 1.53 (d, $^3J_{HP}$ = 10.8 Hz, 18H, ^{t}Bu CH_3), 1.30 - 1.45 (m, 18H, Pip Pos. 2 + Pos. 3 CH_2).
^{13}C NMR (101 MHz, C_6D_6): δ = 47.9 (d, $^2J_{CP}$ = 4.8 Hz, Pip Pos. 1 CH_2), 36.0 (dd, $^1J_{CP}$ = 23.4 Hz, $^3J_{CP}$ = 10.7 Hz, ^{t}Bu $C(CH_3)_3$), 33.0 (d, $^2J_{CP}$ = 15.2 Hz, ^{t}Bu $C(CH_3)_3$), 27.1 (d, $^3J_{CP}$ = 5.0 Hz, Pip Pos. 2 CH_2), 25.5 (d, $^4J_{CP}$ = 1.2 Hz, Pip Pos. 3 CH_2), 17.5 (d, $^2J_{CP}$ = 9.9 Hz, P-C$^-$-CH_3), 16.9 (dd, $^1J_{CP}$ = 191.5, 25.3 Hz, P-C^--P).
^{31}P NMR (162 MHz, C_6D_6): δ = 60.8 (d, $^2J_{PP}$ = 208.5 Hz, $P(Pip)_3$), 25.3 (d, $^2J_{PP}$ = 208.9 Hz, P^tBu_2).
IR (ATR) [cm^{-1}]: 2924 (m), 2848 (w), 1636 (w), 1439 (w), 1356 (w), 1322 (w), 1258 (w), 1213 (m), 1153 (s), 1119 (w), 1052 (vs), 939 (vs), 888 (s), 854 (m), 808 (m), 696 (m), 587 (m), 545 (m).
Melting point: 147.8 °C.
HRMS (ESI): m/z $C_{25}H_{52}N_3P_2$ $[M+H]^+$ Calculated: 456.3631 Found: 456.3625.

^{Pip}adYPhos

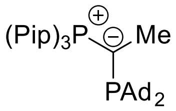

3.8 g (8.64 mmol, 1 eq.) ethyltri(piperidin-1-yl)phosphonium iodide (**1·H₂**) was suspended in 15 mL THF and deprotonated by slow addition of 5.72 mL of a *n*-BuLi solution (1.51 M in hexane, 8.64 mmol, 1 eq.) until a clear, slightly yellow solution was formed. The solution was stirred for 45 min and then a solution of 1.5 g diadamantylchlorophosphine (4.32 mmol, 0.5 eq.) in 10 mL THF was added at room temperature. The solution was stirred for 24 h at reflux, during which time reformed phosphonium salt precipitated as a white solid. The solid was filtered off and the solvent of the solution was removed *in vacuo*. The residue was suspended in 25 mL acetonitrile and stirred overnight, forming a colorless solid. The solid was filtered off, washed with acetonitrile 4× 10 mL and dried *in vacuo* thus giving **^{Pip}AdYPhos** (1.87 g, 3.06 mmol, 71%) as a colorless solid.

^{1}H NMR (400 MHz, C_6D_6): δ = 3.12 (q, J_{HH} = 4.93 Hz, 12H, CH_2 Pip Pos. 1), 2.46 – 2.30 (m, 12H, CH_2 Ad), 2.17 – 2.07 (m, 9H, CH Ad + CH_3), 1.95 (d, J_{HH} = 11.35 Hz, 6H, CH_2 Ad), 1.84 (d, J_{HH} = 12.19 Hz, 6H, CH_2 Ad), 1.48 – 1.33 (m, 18H, CH_2 Pip Pos. 2 +3).
^{13}C NMR (101 MHz, C_6D_6): δ = 48.0 (d, $^2J_{CP}$ = 4.70 Hz, CH_2 Pip Pos. 1), 43.9 (d, $^2J_{CP}$ = 12.85 Hz, CH_2 Ad), 40.9 (dd, $^1J_{CP}$ = 23.25, $^3J_{CP}$ = 10.68 Hz, C Ad), 38.1 (CH_2 Ad), 30.1 (d, $^3J_{CP}$ = 8.35 Hz, CH Ad), 27.1 (d, $^3J_{CP}$ = 5.14 Hz, CH_2 Pip Pos. 2), 25.5 (CH_2 Pip Pos. 3), 18.9 (dd, $^2J_{CP}$ = 10.30, 1.75 Hz, CH_3), 14.7 (dd, $^1J_{CP}$ = 191.09, 25.37 Hz, PC^-P).
^{31}P NMR (162 MHz, C_6D_6): δ = 61.3 (d, $^2J_{PP}$ = 208.13 Hz, $P(Pip)_3$), 30.1 (d, $^2J_{PP}$ = 207.55 Hz, PAd_2).
IR (ATR) [cm^{-1}]: 2898 (m), 2844 (w), 2673 (w), 1447 (w), 1368 (w), 1325 (w), 1258 (w), 1213 (w), 1154 (w), 1118 (w), 1058 (m), 1024 (w), 933 (vs), 884 (m), 809 (w), 693 (m), 667 (w), 558 (w), 466 (m), 423 (m).
Melting point: 179.9 °C (decomposition).
HRMS (ESI): m/z $C_{37}H_{64}N_3P_2$ $[M+H]^+$ Calculated: 612.4575 Found: 612.4574.

5. Synthesis and Characterization of Products

(2*S*,5*R*)-2-isopropyl-3,6-dimethoxy-5-(4-methoxyphenyl)-2,5-dihydropyrazine (**3aa**) [CAS: 109012-91-1]

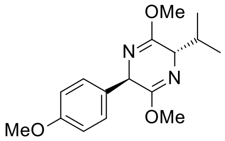

Following general procedure **A** and starting from 4-chloroanisole (71.3 mg, 0.5 mmol), the title compound was obtained as a yellow solid (141 mg, 0.486 mmol, 97%, dr = >25:1).
Before isolation GC-analysis of the crude reaction mixture showed a dr = 15:1.

m.p.: 55-57 °C.
^{1}H NMR (300 MHz, $CDCl_3$): δ = 7.19 - 7.14 (m, 2H), 6.90 - 6.85 (m, 2H), 5.04 (d, *J* = 3.6 Hz, 1 H), 4.10 (dd, *J* = 3.4, 3.4 Hz, 1 H), 3.80 (s, 3 H), 3.72 (s, 3 H), 3.64 (s, 3 H), 2.38 (sptd, *J* = 6.8, 3.4 Hz, 1 H), 1.12 (d, *J* = 6.9 Hz, 3 H), 0.77 (d, *J* = 6.8 Hz, 3 H) ppm.
^{13}C NMR (75 MHz, $CDCl_3$): δ = 164.1, 162.7, 158.9, 132.8, 128.7, 113.7, 60.6, 59.4, 55.2, 52.7, 52.6, 31.5, 19.1, 16.5 ppm.
IR (ATR): $\tilde{\nu}$ = 2943, 2870, 1699, 1611, 1585, 1513, 1457, 1432, 1230, 1030 cm^{-1}.
HRMS (EI-TOF) $[M]^+$ calcd. for $C_{16}H_{22}N_2O_3$: 290.1630; found: 290.1630.

(2*S*,5*R*)-2-isopropyl-3,6-dimethoxy-5-(4-tolyl)-2,5-dihydropyrazine (**3ba**)

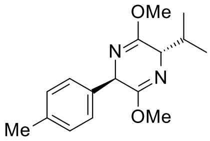

Following general procedure **A** and starting from 4-chlorotoluene (63.3 mg, 0.5 mmol), the title compound was obtained as a colorless oil (127 mg, 0.463 mmol, 93%, dr = >25:1).
Before isolation GC-analysis of the crude reaction mixture showed a dr = 16:1.

^{1}H NMR (300 MHz, $CDCl_3$): δ = 7.17 - 7.11 (m, 4 H), 5.06 (d, *J* = 3.6 Hz, 1 H), 4.11 (dd, *J* = 3.6, 3.4 Hz, 1 H), 3.71 (s, 3 H), 3.64 (s, 3 H), 2.45 - 2.36 (m, 1 H), 2.34 (s, 3 H), 1.12 (d, *J* = 6.9 Hz, 3 H), 0.77 (d, *J* = 6.8 Hz, 3 H) ppm.
^{13}C NMR (75 MHz, $CDCl_3$): δ = 164.0, 162.6, 137.6, 137.0, 129.1, 127.5, 60.6, 59.8, 52.7, 52.6, 31.5, 21.1, 19.1, 16.5 ppm.
IR (ATR): $\tilde{\nu}$ = 2958, 2944, 1690, 1513, 1460, 1381, 1234, 1193, 1008, 770 cm^{-1}.
HRMS (EI-TOF) $[M]^+$ calcd. for $C_{16}H_{22}N_2O_2$: 274.1681; found: 274.1685.

(2*S*,5*R*)-2-isopropyl-3,6-dimethoxy-5-(3-tolyl)-2,5-dihydropyrazine (**3ca**)

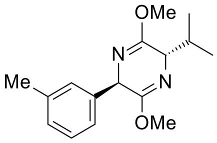

Following general procedure **A** and starting from 3-chlorotoluene (64.6 mg, 0.5 mmol), the title compound was obtained as a colorless oil (125 mg, 0.456 mmol, 91%, dr = >25:1).
Before isolation GC-analysis of the crude reaction mixture showed a dr = 14:1.

^{1}H NMR (300 MHz, $CDCl_3$): δ = 7.26 - 7.21 (m, 1 H), 7.11 - 7.09 (m, 1 H), 7.06 - 7.03 (m, 2 H), 5.06 (d, *J* = 3.6 Hz, 1 H), 4.14 (dd, *J* = 3.6, 3.4 Hz, 1 H), 3.73 (s, 3 H), 3.66 (s, 3 H), 2.50 - 2.38 (m, 1 H), 2.36 (s, 3 H), 1.14 (d, *J* = 6.9 Hz, 3 H), 0.78 (d, *J* = 6.8 Hz, 3 H) ppm.
^{13}C NMR (75 MHz, $CDCl_3$): δ = 164.0, 162.5, 140.5, 138.0, 128.4, 128.3, 128.2, 124.8, 60.6, 60.2, 52.7, 52.7, 31.5, 21.5, 19.1, 16.5 ppm.
IR (ATR): $\tilde{\nu}$ = 2958, 2944, 1690, 1608, 1488, 1460, 1365, 1233, 1193, 1009 cm^{-1}.
HRMS (EI-TOF) $[M]^+$ calcd. for $C_{16}H_{22}N_2O_2$: 274.1681; found: 274.1684.

(2*S*,5*R*)-2-isopropyl-3,6-dimethoxy-5-(2-tolyl)-2,5-dihydropyrazine (**3da**)

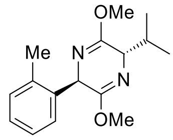

Following general procedure **A** and starting from 2-chlorotoluene (64.6 mg, 0.5 mmol), the title compound was obtained as a colorless oil (70 mg, 0.255 mmol, 51%, dr = 11:1).
Before isolation GC-analysis of the crude reaction mixture showed a dr = 11:1.

^{1}H NMR (300 MHz, $CDCl_3$): δ = 7.20 - 7.16 (m, 3 H), 7.04 - 6.98 (m, 1 H), 5.29 (d, *J* = 3.8 Hz, 1 H), 4.14 (dd, *J* = 3.8, 3.6 Hz, 1 H), 3.70 (s, 3 H), 3.65 (s, 3 H), 2.44 (s, 3 H), 2.42 - 2.32 (m, 1 H), 1.14 (d, *J* = 6.9 Hz, 3 H), 0.81 (d, *J* = 6.8 Hz, 3 H) ppm.
^{13}C NMR (75 MHz, $CDCl_3$): δ = 163.9, 162.9, 138.9, 136.5, 130.6, 127.6, 127.3, 126.1, 60.9, 57.4, 52.7, 52.6, 31.8, 19.4, 19.1, 16.7 ppm.
IR (ATR): $\tilde{\nu}$ = 2958, 2944, 1690, 1461, 1435, 1365, 1332, 1235, 1193, 1009 cm^{-1}.
HRMS (EI-TOF) $[M]^+$ calcd. for $C_{16}H_{22}N_2O_2$: 274.1681; found: 274.1687.

(2*S*,5*S*)-2-(2,6-dimethylphenyl)-5-isopropyl-3,6-dimethoxy-2,5-dihydropyrazine (**3ea**)

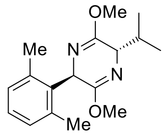

Following general procedure **A** and starting from 2,6-dimethylbenzene (70.6 mg, 0.5 mmol), the title compound was obtained as a yellow oil (30 mg, 0.104 mmol, 21%, dr = >25:1).
Before isolation GC-analysis of the crude reaction mixture showed a dr = >25:1.

^{1}H NMR (300 MHz, $CDCl_3$): δ = 7.12 – 7.00 (m, 3 H), 5.40 (d, *J* = 7.7 Hz, 1 H), 3.91 (dd, *J* = 7.8, 3.0 Hz, 1 H), 3.70 (s, 3 H), 3.65 (s, 3 H), 2.63 - 2.49 (m, 1 H), 2.35 (br. s., 3 H), 2.14 (br. s., 3 H), 1.19 (d, *J* = 6.8 Hz, 3 H), 0.82 (d, *J* = 6.7 Hz, 3 H) ppm.

Coupling constant of 7.7 Hz for the doublet at 5.40 ppm, suggests a cis-configuration of the bis-lactim ether, which is in accordance with spectroscopic literature data of other syn-arylated bis-lactim ethers.[5] However, comparative NOESY experiments suggest an trans-configuration of the product (chapter 10). Neither 1D-NMR nor 2D-NMR investigations allowed us to pinpoint the absolute stereochemistry of the dominating stereoisomer. It is tentatively drawn as the expected trans-isomer, but a cis-configuration cannot be excluded.
^{13}C NMR (75 MHz, $CDCl_3$): *δ* = 163.6, 163.1, 136.9, 136.3, 127.4, 127.3, 60.5, 55.7, 53.1, 53.0, 30.2, 20.7, 20.5, 19.8, 17.2 ppm.
IR (ATR): $\tilde{\nu}$ = 2961, 1682, 1459, 1435, 1339, 1297, 1228, 1196, 1011, 791 cm^{-1}.
HRMS (ESI-TOF) $[M + H]^+$ calcd. for $C_{17}H_{25}N_2O_2$: 289.1911; found: 289.1911.

(2*S*,5*R*)-2-isopropyl-3,6-dimethoxy-5-phenyl-2,5-dihydropyrazine (**3fa**) [CAS: 126204-55-5]

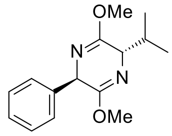

Following general procedure **A** and starting from chlorobenzene (56.6 mg, 0.5 mmol), the title compound was obtained as a colorless solid (115 mg, 0.442 mmol, 88%, dr = >25:1).
Before isolation GC-analysis of the crude reaction mixture showed a dr = 15:1.

m.p.: 35-37 °C.
^{1}H NMR (300 MHz, $CDCl_3$): *δ* = 7.38 - 7.24 (m, 5 H), 5.11 (d, *J* = 3.7 Hz, 1 H), 4.12 (dd, *J* = 3.7, 3.4 Hz, 1 H), 3.74 (s, 3 H), 3.65 (s, 3 H), 2.40 (sptd, *J* = 6.8, 3.4 Hz, 1 H), 1.13 (d, *J* = 6.9 Hz, 3 H), 0.79 (d, *J* = 6.8 Hz, 3 H) ppm.
^{13}C NMR (75 MHz, $CDCl_3$): *δ* = 164.3, 162.4, 140.5, 128.3, 127.7, 127.4, 60.6, 60.1, 52.7, 52.7, 31.6, 19.2, 16.5 ppm.
IR (ATR): $\tilde{\nu}$ = 2957, 1690, 1455, 1434, 1307, 1230, 1193, 1006, 787, 756 cm^{-1}.
HRMS (EI-TOF) $[M]^+$ calcd. for $C_{15}H_{20}N_2O_2$: 260.1525; found: 260.1528.

(2*R*,5*S*)-2-(4-fluorophenyl)-5-isopropyl-3,6-dimethoxy-2,5-dihydropyrazine (**3ga**)

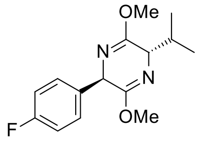

Following general procedure **A** and starting from 1-chloro-4-fluorobenzene (65.3 mg, 0.5 mmol), the title compound was obtained as a yellow oil (125 mg, 0.449 mmol, 90%, dr = >25:1).
Before isolation GC-analysis of the crude reaction mixture showed a dr = >25:1.

^{1}H NMR (300 MHz, $CDCl_3$): *δ* = 7.25 - 7.20 (m, 2 H), 7.05 - 6.98 (m, 2 H), 5.08 (d, *J* = 3.6 Hz, 1 H), 4.11 (dd, *J* = 3.6, 3.5 Hz, 1 H), 3.73 (s, 3 H), 3.64 (s, 3 H), 2.39 (sptd, *J* = 6.8, 3.5 Hz, 1 H), 1.12 (d, *J* = 7.0 Hz, 3 H), 0.77 (d, *J* = 6.8 Hz, 3 H) ppm.
^{13}C NMR (75 MHz, $CDCl_3$): *δ* = 164.5, 162.2, 162.1 (d, *J* = 245.2 Hz), 136.3 (d, *J* = 3.3 Hz), 129.3 (d, *J* = 8.3 Hz), 115.1 (d, *J* = 21.5 Hz), 60.7, 59.2, 52.7, 52.7, 31.6, 19.1 16.5 ppm.
^{19}F NMR (41 MHz, CDCl3): δ = –113.1 - –113.7 (m) ppm.
IR (ATR): $\tilde{\nu}$ = 2959, 1694, 1603, 1508, 1461, 1380, 1308, 1235, 1221, 1005 cm^{-1}.
HRMS (EI-TOF) $[M]^+$ calcd. for $C_{15}H_{19}FN_2O_2$: 278.1431; found: 278.1433.

(2*S*,5*R*)-2-isopropyl-3,6-dimethoxy-5-[4-(trifluoromethoxy)phenyl]-2,5-dihydropyrazine (**3ha**)

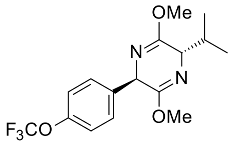

Following general procedure **A** and starting from 4-(trifluoromethoxy)chlorobenzene (98.3 mg, 0.5 mmol), the title compound was obtained as a colorless solid (132 mg, 0.383 mmol, 77%, dr = >25:1).
Before isolation GC-analysis of the crude reaction mixture showed a dr = 10:1.

m.p.: 36-39 °C.
^{1}H NMR (300 MHz, $CDCl_3$): *δ* = 7.31 - 7.26 (m, 2 H), 7.20 - 7.17 (m, 2 H), 5.11 (d, *J* = 3.6 Hz, 1 H), 4.11 (dd, *J* = 3.6, 3.4 Hz, 1 H), 3.74 (s, 3 H), 3.65 (s, 3 H), 2.40 (sptd, *J* = 6.8, 3.4 Hz, 1 H), 1.12 (d, *J* = 6.9 Hz, 3 H), 0.78 (d, *J* = 6.8 Hz, 3 H) ppm.
^{13}C NMR (75 MHz, $CDCl_3$): *δ* = 164.7, 161.8, 148.4 (q, *J* = 1.7 Hz), 139.1, 129.1, 120.8, 120.5 (q, *J* = 257.2 Hz), 60.7, 59.2, 52.8, 52.2, 31.7, 19.1, 16.5 ppm.
^{19}F NMR (41 MHz, $CDCl_3$): δ = –55.8 ppm.
IR (ATR): $\tilde{\nu}$ = 2967, 2946, 1695, 1673, 1507, 1462, 1437, 1310, 1254, 1191 cm^{-1}.
HRMS (EI-TOF) $[M]^+$ calcd. for $C_{16}H_{19}F_3N_2O_3$: 344.1348; found: 344.1352.

(2*S*,5*R*)-2-isopropyl-3,6-dimethoxy-5-(4-thioanisole)-2,5-dihydropyrazine (**3ia**)

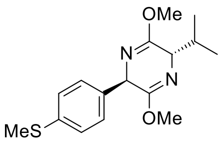

Following general procedure **A** and starting from 4-chlorothioanisole (80.5 mg, 0.5 mmol), the title compound was obtained as a white solid (115 mg, 0.375 mmol, 75%, dr = >25:1).
Before isolation GC-analysis of the crude reaction mixture showed a dr = >25:1.

m.p.: 62-66 °C.
^{1}H NMR (300 MHz, $CDCl_3$): *δ* = 7.26 - 7.20 (m, 2 H), 7.20 - 7.14 (m, 2 H), 5.05 (d, *J* = 3.6 Hz, 1 H), 4.10 (dd, *J* = 3.6, 3.4 Hz, 1 H), 3.72 (s, 3 H), 3.64 (s, 3 H), 2.48 (s, 3 H), 2.45 - 2.31 (m, 1 H), 1.12 (d, *J* = 6.9 Hz, 3 H), 0.77 (d, *J* = 6.8 Hz, 3 H) ppm.
^{13}C NMR (75 MHz, $CDCl_3$): *δ* = 164.3, 162.3, 137.5, 137.4, 128.1, 126.6, 60.7, 59.6, 52.7, 52.7, 31.6, 19.1, 16.5, 15.9 ppm.
IR (ATR): $\tilde{\nu}$ = 2959, 2943, 1687, 1460, 1434, 1381, 1333, 1301, 1230, 1009 cm^{-1}.

HRMS (EI-TOF) $[M]^+$ calcd. for $C_{16}H_{22}N_2O_2S$: 306.1402; found: 306.1402.

4-[(2*R*,5*S*)-5-isopropyl-3,6-dimethoxy-2,5-dihydropyrazin-2-yl]-*N*,*N*-dimethyl-aniline (**3ja**)

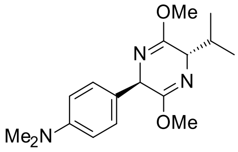

Following general procedure **A** and starting from 4-chloro-*N*,*N*-dimethylaniline (80.2 mg, 0.5 mmol), the title compound was obtained as a yellow solid (145 mg, 0.478 mmol, 96%, dr = >25:1).

m.p.: 81-84 °C.
^{1}H NMR (300 MHz, $CDCl_3$): δ = 7.14 - 7.09 (m, 2 H), 6.73 - 6.69 (m, 2 H), 5.02 (d, *J* = 3.6 Hz, 1 H), 4.11 (dd, *J* = 3.6, 3.4 Hz, 1 H), 3.72 (s, 3 H), 3.66 (s, 3 H), 2.95 (s, 6 H), 2.40 (sptd, *J* = 6.8, 3.4 Hz, 1 H), 1.13 (d, *J* = 6.9 Hz, 3 H), 0.77 (d, *J* = 6.8 Hz, 3 H) ppm.
^{13}C NMR (75 MHz, $CDCl_3$): δ = 163.7, 163.1, 149.9, 128.6, 128.2, 112.4, 60.5, 59.5, 52.6, 52.6, 40.6, 31.5, 19.2, 16.4 ppm.
IR (ATR): $\tilde{\nu}$ = 2956, 2944, 1689, 1615, 1523, 1453, 1227, 1186, 1013, 722 cm^{-1}.
HRMS (EI-TOF) $[M]^+$ calcd. for $C_{17}H_{25}N_3O_2$: 303.1947; found: 303.1951.

(2R,5S)-2-[4-(dimethoxymethyl)phenyl]-5-isopropyl-3,6-dimethoxy-2,5-dihydropyrazine (**3ka**)

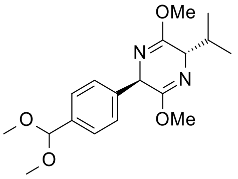

Following general procedure **A** and starting from 4-chlorobenzaldehyde dimethyl acetal (95.2 mg, 0.5 mmol), the title compound was obtained as a yellow oil (145 mg, 0.434 mmol, 87%, dr = 19:1).
Before isolation GC-analysis of the crude reaction mixture showed a dr = 19:1.

^{1}H NMR (400 MHz, $CDCl_3$): δ = 7.42 - 7.40 (m, 2 H), 7.25 - 7.23 (m, 2 H), 5.38 (s, 1 H), 5.10 (d, *J* = 3.5 Hz, 1 H), 4.10 (dd, *J* = 3.5, 3.4 Hz, 1 H), 3.72 (s, 3 H), 3.62 (s, 3 H), 3.33 (s, 6 H), 2.39 (sptd, *J* = 6.9, 3.4 Hz, 1 H), 1.12 (d, *J* = 6.9 Hz, 3 H), 0.77 (d, *J* = 6.9 Hz, 3 H) ppm.
^{13}C NMR (101 MHz, acetone-d_6): δ = 164.8, 163.3, 141.9, 138.6, 128.3, 127.5, 103.8, 61.1, 60.7, 52.9, 52.9, 32.0, 19.7, 16.7 ppm.
Carbon missing due to overlap of signals.
IR (ATR): $\tilde{\nu}$ = 2946, 2832, 1692, 1461, 1437, 1306, 1238, 1100, 1052, 1009 cm^{-1}.
HRMS (EI-TOF) $[M]^+$ calcd. for $C_{18}H_{27}N_2O_4$: 335.1965; found: 335.1964.

(2*S*,5*R*)-2-isopropyl-3,6-dimethoxy-5-[4-(trifluoromethyl)phenyl]-2,5-dihydropyrazine (**3la**)

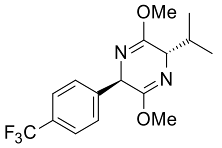

Following general procedure **A** and starting from 4-chlorbenzotrifluoride (90.3 mg, 0.5 mmol), the title compound was obtained as a colorless oil (151 mg, 0.460 mmol, 92%, dr = >25:1).
Before isolation GC-analysis of the crude reaction mixture showed a dr = >25:1.

^{1}H NMR (300 MHz, $CDCl_3$): δ = 7.61 - 7.59 (m, 2 H), 7.39 - 7.36 (m, 2 H), 5.15 (d, *J* = 3.7 Hz, 1 H), 4.12 (dd, *J* = 3.7, 3.5 Hz, 1 H), 3.74 (s, 3 H), 3.64 (s, 3 H), 2.39 (sptd, *J* = 6.8, 3.5 Hz, 1 H), 1.12 (d, *J* = 6.9 Hz, 3 H), 0.78 (d, *J* = 6.8 Hz, 3 H) ppm.
^{13}C NMR (75 MHz, $CDCl_3$): δ = 164.9, 161.5, 144.3, 129.6 (q, *J* = 32.4 Hz), 128.1, 125.3 (q, *J* = 3.9 Hz), 124.2 (q, *J* = 272 Hz), 60.8, 59.6, 52.8, 52.8, 31.7, 19.1 16.5 ppm.
^{19}F NMR (41 MHz, $CDCl_3$): δ = −60.5 ppm.
IR (ATR): $\tilde{\nu}$ = 2963, 2948, 1697, 1674, 1619, 1332, 1233, 1197, 1166, 1111 cm^{-1}.
HRMS (EI-TOF) $[M]^+$ calcd. for $C_{16}H_{19}F_3N_2O_2$: 328.1399; found: 328.1397.

4-[(2*R*,5*S*)-5-isopropyl-3,6-dimethoxy-2,5-dihydropyrazin-2-yl]-*N*,*N*-dimethyl-benzamide (**3ma**)

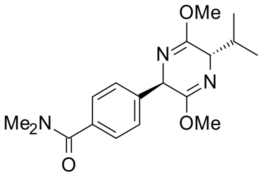

Following general procedure **A** and starting from 4-chloro-*N*,*N*-dimethyl-benzamide (93.7 mg, 0.5 mmol), the title compound was obtained as a yellow oil (149 mg, 0.450 mmol, 90%, dr = 14:1).
Before isolation GC-analysis of the crude reaction mixture showed a dr = 19:1.

^{1}H NMR (300 MHz, $CDCl_3$): δ = 7.30 - 7.28 (m, 2 H), 7.18 - 7.16 (m, 2 H), 5.01 (d, *J* = 3.6 Hz, 1 H), 4.00 (dd, *J* = 3.6, 3.4 Hz, 1 H), 3.63 (s, 3 H), 3.52 (s, 3 H), 3.00 (br. s., 3 H), 2.89 (br. s., 3 H), 2.28 (sptd, *J* = 6.8, 3.4 Hz, 1 H), 1.01 (d, *J* = 6.9 Hz, 3 H), 0.67 (d, *J* = 6.8 Hz, 3 H) ppm.
^{13}C NMR (75 MHz, $CDCl_3$): δ = 171.5, 164.7, 161.9, 141.8, 135.3, 127.7, 127.1, 60.7, 59.7, 52.7, 52.7, 39.6, 35.3, 31.6, 19.1, 16.5 ppm.
IR (ATR): $\tilde{\nu}$ = 3235, 2945, 2871, 1737, 1691, 1633, 1570, 1460 1236, 1080 cm^{-1}.
HRMS (EI-TOF) $[M]^+$ calcd. for $C_{18}H_{25}N_3O_3$: 331.1896; found: 331.1893.

ethyl 4-[(2*R*,5*S*)-5-isopropyl-3,6-dimethoxy-2,5-dihydropyrazin-2-yl]benzoate (**3na**)

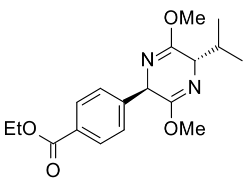

Following general procedure **A** and starting from ethyl 4-chlorobenzoate (94.2 mg, 0.5 mmol), the title compound was obtained as a colorless oil (128 mg, 0.385 mmol, 77%, dr = >25:1).
Before isolation GC-analysis of the crude reaction mixture showed a dr = >25:1.

^{1}H NMR (300 MHz, $CDCl_3$): δ = 8.04 - 7.99 (m, 2 H), 7.34 - 7.30 (m, 2 H), 5.14 (d, *J* = 3.6 Hz, 1 H), 4.37 (q, *J* = 7.2 Hz, 2 H), 4.12 (dd, *J* = 3.6, 3.4 Hz, 1 H), 3.73 (s, 3 H), 3.63 (s, 3 H), 2.39 (sptd, *J* = 6.9, 3.4 Hz, 1 H), 1.39 (t, *J* = 7.1 Hz, 3 H), 1.12 (d, *J* = 6.9 Hz, 3 H), 0.78 (d, *J* = 6.8 Hz, 3 H) ppm.
^{13}C NMR (75 MHz, $CDCl_3$): δ = 166.4, 164.7, 161.6, 145.3, 129.6, 129.5, 127.7, 60.9, 60.7, 59.8, 52.8, 52.8, 31.7, 19.1, 16.5, 14.3 ppm.
IR (ATR): $\tilde{\nu}$ = 2960, 2871, 1718, 1691, 1610, 1306, 1271, 1236, 1101, 1007 cm^{-1}.
HRMS (EI-TOF) $[M]^+$ calcd. for $C_{18}H_{24}N_2O_4$: 332.1736; found: 332.1741.

[4-[(2*R*,5*S*)-5-isopropyl-3,6-dimethoxy-2,5-dihydropyrazin-2-yl]phenyl]-phenyl-methanone (**3oa**)

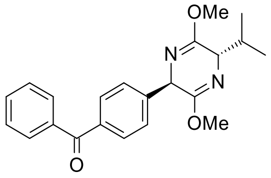

Following general procedure **A** and starting from 4-chlorobenzophenone (109 mg, 0.5 mmol), the title compound was obtained as a yellow oil (83 mg, 0.228 mmol, 46%, dr = >25:1).

^{1}H NMR (300 MHz, $CDCl_3$): δ = 7.84 - 7.77 (m, 4 H), 7.62 - 7.57 (m, 1 H), 7.52 - 7.46 (m, 2 H), 7.39 - 7.35 (m, 2 H), 5.18 (d, *J* = 3.6 Hz, 1 H), 4.14 (dd, *J* = 3.6, 3.5 Hz, 1 H), 3.76 (s, 3 H), 3.66 (s, 3 H), 2.40 (sptd, *J* = 6.9, 3.5 Hz, 1 H), 1.13 (d, *J* = 6.9 Hz, 3 H), 0.79 (d, *J* = 6.8 Hz, 3 H) ppm.
^{13}C NMR (75 MHz, $CDCl_3$): δ = 196.4, 164.8, 161.6, 144.9, 137.7, 136.6, 132.3, 130.2, 130.0, 128.2, 127.7, 60.8, 59.8, 52.8, 52.8, 31.7, 19.1, 16.6 ppm.
IR (ATR): $\tilde{\nu}$ = 2944, 2870, 1737, 1691, 1656, 1605, 1306, 1275, 1236, 700 cm^{-1}.
HRMS (EI-TOF) $[M]^+$ calcd. for $C_{22}H_{24}N_2O_3$: 364.1787; found: 364.1787.

(2*S*,5*R*)-2-isopropyl-3,6-dimethoxy-5-[4-(4,4,5,5-tetramethyl-1,3,2-dioxaborolan-2-yl)phenyl]-2,5-dihydropyrazine (**3pa**)

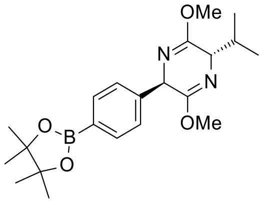

Following general procedure **A** and starting from 4-chlorophenylboronic acid pinacol ester (122 mg, 0.5 mmol), the title compound was obtained as a colorless oil (150 mg, 0.388 mmol, 78%, dr = 13:1).
Before isolation GC-analysis of the crude reaction mixture showed a dr = 11:1.

^{1}H NMR (300 MHz, $CDCl_3$): δ = 7.80 - 7.77 (m, 2 H), 7.26 - 7.23 (m, 2 H), 5.10 (d, *J* = 3.6 Hz, 1 H), 4.12 (dd, *J* = 3.6, 3.4 Hz, 1 H), 3.71 (s, 3 H), 3.62 (s, 3 H), 2.39 (sptd, *J* = 6.9, 3.4 Hz, 1 H), 1.34 (s, 12 H), 1.12 (d, *J* = 6.9 Hz, 3 H), 0.77 (d, *J* = 6.8 Hz, 3 H) ppm.
^{13}C NMR (75 MHz, $CDCl_3$): δ = 164.3, 162.2, 143.5, 134.9, 127.1, 83.7, 60.7, 60.2, 52.7, 52.7, 31.6, 24.9, 24.8, 19.1, 16.5 ppm.
Carbon directly attached to the boron was not detected, likely due to quadrupolar relaxation.[6]
IR (ATR): $\tilde{\nu}$ = 2975, 2944, 1692, 1611, 1307, 1194, 1142, 1057, 1009, 656 cm^{-1}.
HRMS (EI-TOF) $[M]^+$ calcd. for $C_{21}H_{31}BN_2O_4$: 385.2414; found: 385.2413.

[4-[(2*R*,5*S*)-5-isopropyl-3,6-dimethoxy-2,5-dihydropyrazin-2-yl]phenyl]-trimethyl-silane (**3qa**)

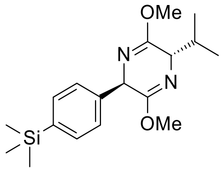

Following general procedure **A** and starting from 4-chlorophenyltrimethylsilane (95.2 mg, 0.5 mmol), the title compound was obtained as a yellow solid (135 mg, 0.406 mmol, 81%, dr = >25:1).

m.p.: 53-60 °C.
^{1}H NMR (300 MHz, $CDCl_3$): δ = 7.52 - 7.49 (m, 2 H), 7.25 - 7.23 (m, 2 H), 5.10 (d, *J* = 3.6 Hz, 1 H), 4.12 (dd, *J* = 3.6, 3.4 Hz, 1 H), 3.73 (s, 3 H), 3.66 (s, 3 H), 2.41 (sptd, *J* = 6.9, 3.4 Hz, 1 H), 1.14 (d, *J* = 6.9 Hz, 3 H), 0.78 (d, *J* = 6.8 Hz, 3 H), 0.27 (s, 9 H) ppm.
^{13}C NMR (75 MHz, $CDCl_3$): δ = 164.2, 162.4, 141.0, 139.3, 133.4, 127.0, 60.6, 60.1, 52.7, 52.7, 31.5, 19.2, 16.5, –1.1 ppm.
IR (ATR): $\tilde{\nu}$ = 2956, 2908, 1691, 1674, 1599, 1247, 1234, 1197, 1054, 825 cm^{-1}.
HRMS (EI-TOF) $[M]^+$ calcd. for $C_{18}H_{28}N_2O_2Si$: 332,1920; found: 332.1924.

[4-[(2R,5S)-5-isopropyl-3,6-dimethoxy-2,5-dihydropyrazin-2-yl]phenyl] methanesulfonate (**3ra**)

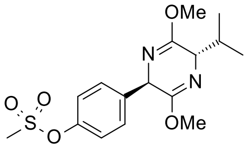

Following general procedure **A** and starting from 4-chlorophenyl methanesulfonate (103 mg, 0.5 mmol), the title compound was obtained as a colorless solid (140 mg, 0.395 mmol, 79%, dr = >25:1).
Before isolation GC-analysis of the crude reaction mixture showed a dr = 16:1.

m.p.: 78-82 °C.
^{1}H NMR (300 MHz, $CDCl_3$): δ = 7.33 - 7.23 (m, 4 H), 5.11 (d, *J* = 3.6 Hz, 1 H), 4.10 (dd, *J* = 3.6, 3.5 Hz, 1 H), 3.73 (s, 3 H), 3.64 (s, 3 H), 3.13 (s, 3 H), 2.38 (sptd, *J* = 6.9, 3.5 Hz, 1 H), 1.11 (d, *J* = 6.9 Hz, 3 H), 0.77 (d, *J* = 6.8 Hz, 3 H) ppm.
^{13}C NMR (75 MHz, $CDCl_3$): δ = 164.8, 161.7, 148.4, 139.8, 129.4, 121.8, 60.7, 59.2, 52.8, 52.8, 37.2, 31.6, 19.1, 16.5 ppm.
IR (ATR): $\tilde{\nu}$ = 2958, 2929, 1736, 1692, 1500, 1364, 1330, 1309, 1150, 1004 cm^{-1}.
HRMS (EI-TOF) $[M]^+$ calcd. for $C_{16}H_{22}N_2O_5S$: 354.1249; found: 354.1253.

2-[4-[(2*R*,5*S*)-5-isopropyl-3,6-dimethoxy-2,5-dihydropyrazin-2-yl]phenyl]ethynyl-trimethyl-silane (**3sa**)

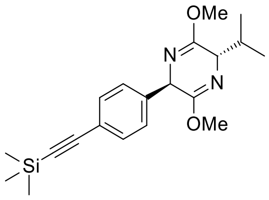

Following general procedure **A** and starting from ((4-chlorophenyl)ethynyl)trimethylsilane (108 mg, 0.5 mmol), the title compound was obtained as a colorless oil (69 mg, 0.194 mmol, 39%, dr = >25:1).

^{1}H NMR (300 MHz, $CDCl_3$): δ = 7.45 - 7.41 (m, 2 H), 7.20 - 7.16 (m, 2 H), 5.07 (d, *J* = 3.6 Hz, 1 H), 4.10 (dd, *J* = 3.6, 3.4 Hz, 1 H), 3.73 (s, 3 H), 3.62 (s, 3 H), 2.38 (sptd, *J* = 6.8, 3.4 Hz, 1 H), 1.11 (d, *J* = 6.9 Hz, 3 H), 0.77 (d, *J* = 6.8 Hz, 3 H), 0.25 (s, 9 H) ppm.
^{13}C NMR (75 MHz, $CDCl_3$): δ = 164.6, 162.0, 140.8, 131.9, 127.6, 122.2, 105.0, 94.1, 60.7, 59.8, 52.7, 52.7, 31.6, 19.1, 16.5, 0.0 ppm.
IR (ATR): $\tilde{\nu}$ = 2959, 2901, 2157, 1692, 1594, 1460, 1305, 1235, 863, 839 cm^{-1}.
HRMS (EI-TOF) $[M]^+$ calcd. for $C_{20}H_{28}N_2O_2Si$: 356.1920; found: 356.1924.

(2*S*,5*R*)-2-isopropyl-3,6-dimethoxy-5-(3-pyridyl)-2,5-dihydropyrazine (**3ta**)

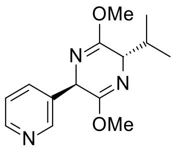

Following general procedure **A** and starting from 3-chloropyridine (56.8 mg, 0.5 mmol), the title compound was obtained as a yellow oil (96 mg, 0.367 mmol, 74%, dr = 4:1).
Before isolation GC-analysis of the crude reaction mixture showed a dr = 4:1.

^{1}H NMR (300 MHz, $CDCl_3$): δ = 8.56 - 8.51 (m, 2 H), 7.57 - 7.53 (m, 1 H), 7.29 - 7.25 (m, 1 H), 5.12 (d, *J* = 3.7 Hz, 1 H), 4.11 (dd, *J* = 3.7, 3.5 Hz, 1 H), 3.74 (s, 3 H), 3.64 (s, 3 H), 2.38 (sptd, *J* = 6.9, 3.4 Hz, 1 H), 1.11 (d, *J* = 6.9 Hz, 3 H), 0.78 (d, *J* = 6.8 Hz, 3 H) ppm.
^{13}C NMR (75 MHz, $CDCl_3$): δ = 165.1, 161.3, 149.4, 148.5, 136.0, 135.2, 123.3, 60.9, 57.6, 52.8, 52.8, 31.8, 19.1, 16.6 ppm.
IR (ATR): $\tilde{\nu}$ = 2945, 2871, 1691, 1578, 1460, 1435, 1307, 1235, 1193, 975 cm^{-1}.
HRMS (EI-TOF) $[M]^+$ calcd. for $C_{14}H_{19}N_3O_2$: 261.1477; found: 261.1483.

(2*S*,5*R*)-2-isopropyl-3,6-dimethoxy-5-(3-thienyl)-2,5-dihydropyrazine (**3ua**)

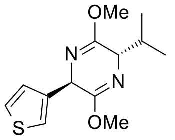

Following general procedure **A** and starting from 3-chlorothiophene (60.5 mg, 0.5 mmol), the title compound was obtained as a yellow oil (118 mg, 0.443 mmol, 89%, dr = >25:1).
Before isolation GC-analysis of the crude reaction mixture showed a dr = 24:1.

^{1}H NMR (300 MHz, $CDCl_3$): δ = 7.28 - 7.25 (m, 1 H), 7.17 - 7.13 (m, 1 H), 7.05 (dd, *J* = 5.0, 1.3 Hz, 1 H), 5.23 (d, *J* = 3.5 Hz, 1 H), 4.03 (dd, *J* = 3.5, 3.4 Hz, 1 H), 3.76 (s, 3 H), 3.69 (s, 3 H), 2.38 (sptd, *J* = 6.8, 3.4 Hz, 1 H), 1.11 (d, *J* = 6.9 Hz, 3 H), 0.76 (d, *J* = 6.8 Hz, 3 H) ppm.
^{13}C NMR (75 MHz, $CDCl_3$): δ = 164.5, 162.0, 140.9, 127.1, 125.4, 121.9, 60.4, 56.0, 52.8, 52.7, 31.3, 19.2, 16.5 ppm.
IR (ATR): $\tilde{\nu}$ = 2959, 2944, 1691, 1460, 1435, 1304, 1235, 1192, 1008, 751 cm^{-1}.
HRMS (EI-TOF) $[M]^+$ calcd. for $C_{13}H_{18}N_2O_2S$: 266.1089; found: 266.1095.

6-[(2*R*,5*S*)-5-isopropyl-3,6-dimethoxy-2,5-dihydropyrazin-2-yl]quinoline (**3va**)

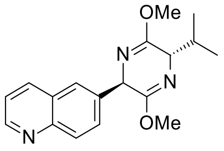

Following general procedure **A** and starting from 6-chloroquinoline (83.5 mg, 0.5 mmol), the title compound was obtained as a yellow solid (116 mg, 0.373 mmol, 75%, dr = 15:1).

m.p.: 92-97 °C.
^{1}H NMR (300 MHz, $CDCl_3$): δ = 8.89 (dd, *J* = 4.3, 1.7 Hz, 1 H), 8.15 - 8.12 (m, 1 H), 8.08 (d, *J* = 8.8 Hz, 1 H), 7.69 (d, *J* = 1.9 Hz, 1 H), 7.59 (dd, *J* = 8.8, 2.1 Hz, 1 H), 7.38 (dd, *J* = 8.3, 4.3 Hz, 1 H), 5.28 (d, *J* = 3.7 Hz, 1 H), 4.18 (dd, *J* = 3.7, 3.4 Hz, 1 H), 3.74 (s, 3 H), 3.63 (s, 3 H), 2.41 (sptd, *J* = 6.9, 3.4 Hz, 1 H), 1.13 (d, *J* = 6.9 Hz, 3 H), 0.80 (d, *J* = 6.8 Hz, 3 H) ppm.
^{13}C NMR (75 MHz, $CDCl_3$): δ = 164.7, 161.9, 150.2, 147.7, 138.8, 136.1, 129.5, 129.5, 128.0, 126.3, 121.2, 60.8, 59.9, 52.8, 52.8, 31.7, 19.1, 16.5 ppm.
IR (ATR): $\tilde{\nu}$ = 2957, 2867, 1689, 1500, 1435, 1366, 1313, 1235, 1213, 1192 cm^{-1}.
HRMS (EI-TOF) $[M]^+$ calcd. for $C_{18}H_{21}N_3O_2$: 311.1634; found: 311.1640.

(2*S*,5*R*)-2-isopropyl-3,6-dimethoxy-5-(3-methylbenzothiophen-5-yl)-2,5-dihydropyrazine (**3wa**)

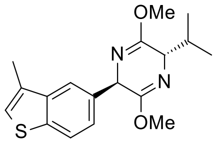

Following general procedure **A** and starting from 5-chloro-3-methylbenzo[b]thiophene (94.2 mg, 0.5 mmol), the title compound was obtained as a white solid (151 mg, 0.457 mmol, 91%, dr = >25:1).
Before isolation GC-analysis of the crude reaction mixture showed a dr = 14:1.

m.p.: 73-77 °C.
^{1}H NMR (300 MHz, $CDCl_3$): δ = 7.83 - 7.80 (m, 1 H), 7.63 (d, *J* = 1.7 Hz, 1 H), 7.22 (dd, *J* = 8.3, 1.7 Hz, 1 H), 7.08 (d, *J* = 1.0 Hz, 1 H), 5.25 (d, *J* = 3.6 Hz, 1 H), 4.21 (dd, *J* = 3.6, 3.5 Hz, 1 H), 3.75 (s, 3 H), 3.67 (s, 3 H), 2.46 - 2.45 (m, 3 H), 2.49 - 2.39 (m, 1 H), 1.17 (d, *J* = 6.9 Hz, 3 H), 0.83 (d, *J* = 6.8 Hz, 3 H) ppm.

^{13}**C NMR** (75 MHz, $CDCl_3$): δ = 164.1, 162.6, 139.8, 139.4, 136.7, 132.1, 123.9, 122.7, 122.0, 120.9, 60.8, 60.3, 52.7, 52.7, 31.7, 19.2, 16.5, 13.9 ppm.
IR (ATR): $\tilde{\nu}$ = 3008, 2963, 2944, 1681, 1464, 1439, 1385, 1369, 1236, 1228 cm^{-1}.
HRMS (EI-TOF) $[M]^+$ calcd. for $C_{18}H_{22}N_2O_2S$: 330.1402; found: 330.1403.

(2*R*,5*S*)-2-(1,3-benzodioxol-5-yl)-5-isopropyl-3,6-dimethoxy-2,5-dihydropyrazine (**3xa**)

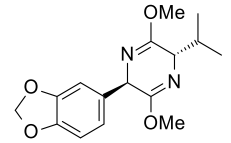

Following general procedure **A** and starting from 5-chlorobenzo-1,3-dioxole (79.9 mg, 0.5 mmol), the title compound was obtained as a colorless oil (124 mg, 0.407 mmol, 82%, dr = >25:1).
Before isolation GC-analysis of the crude reaction mixture showed a dr = 13:1.

^{1}**H NMR** (300 MHz, $CDCl_3$): δ = 6.79 - 6.69 (m, 3 H), 5.94 (s, 2 H), 5.00 (d, *J* = 3.6 Hz, 1 H), 4.10 (dd, *J* = 3.6, 3.4 Hz, 1 H), 3.72 (s, 3 H), 3.65 (s, 3 H), 2.38 (sptd, *J* = 6.9, 3.4 Hz, 1 H), 1.11 (d, *J* = 7.0 Hz, 3 H), 0.76 (d, *J* = 6.8 Hz, 3 H) ppm.
^{13}**C NMR** (75 MHz, $CDCl_3$): δ = 164.2, 162.4, 147.6, 146.8, 134.5, 121.1, 108.1, 108.1, 101.0, 60.6, 59.7, 52.7, 52.7, 31.6, 19.1, 16.5 ppm.
IR (ATR): $\tilde{\nu}$ = 2945, 2872, 1739, 1690, 1503, 1488, 1366, 1306, 1232, 1038 cm^{-1}.
HRMS (EI-TOF) $[M]^+$ calcd. for $C_{16}H_{20}N_2O_4$: 304.1423; found: 304.1428.

(2*R*,5*S*)-2-(3-bicyclo[3.2.1]oct-2-enyl)-5-isopropyl-3,6-dimethoxy-2,5-dihydropyrazine (**3ya**)

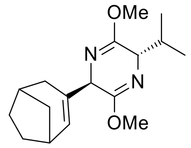

Following general procedure **A** and starting from 3-chloro-bicyclo[3.2.1]oct-2-ene (72.8 mg, 0.5 mmol), the title compound was obtained as a colorless oil (102 mg, 0.351 mmol, 70%, dr = >25:1).
Before isolation GC-analysis of the crude reaction mixture showed a dr = 18:1.

^{1}**H NMR** (300 MHz, $CDCl_3$): δ = 5.91 - 5.87 (m, 1 H), 4.35 (d, *J* = 3.4 Hz, 1 H), 3.93 - 3.90 (m, 1 H), 3.70 - 3.69 (m, 6 H), 2.45 - 2.25 (m, 3 H), 2.11 - 1.36 (m, 8 H), 1.08 - 1.05 (m, 3 H), 0.69 (dd, *J* = 6.8, 0.8 Hz, 3 H) ppm.
^{13}**C NMR** (75 MHz, $CDCl_3$): δ = 164.0, 163.9, 162.7, 162.4, 133.6, 133.2, 132.4, 132.3, 62.0, 61.5, 60.5, 52.6, 52.6, 52.5, 52.5, 36.3, 36.1, 35.5, 35.3, 35.3, 35.2, 32.9, 32.9, 31.4, 31.3, 30.2, 19.1, 16.5, 16.4 ppm.
Complexity due to conformational diastereomers.[7]
IR (ATR): $\tilde{\nu}$ = 2941, 2863, 1688, 1460, 1434, 1366, 1304, 1231, 1193, 1056 cm^{-1}.
HRMS (EI-TOF) $[M]^+$ calcd. for $C_{17}H_{26}N_2O_2$: 290.1994; found: 290.2000.

methyl 4-[(2*R*,5*S*)-5-isopropyl-3,6-dimethoxy-2,5-dihydropyrazin-2-yl]-2,2-dimethyl-pent-4-enoate (**3za**)

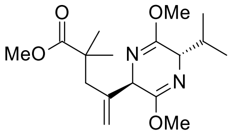

Following general procedure **A** and starting from methyl 4-chloro-2,2-dimethyl-4-pentenoate (89.2 mg, 0.5 mmol), the title compound was obtained as a colorless oil (90 mg, 0.277 mmol, 56%, dr = >25:1).
Before isolation GC-analysis of the crude reaction mixture showed a dr = >25:1.

^{1}**H NMR** (300 MHz, $CDCl_3$): δ = 4.98 (s, 1 H), 4.84 - 4.83 (m, 1 H), 4.45 (d, *J* = 3.5 Hz, 1 H), 3.94 (dd, *J* = 3.4, 3.4 Hz, 1 H), 3.70 (s, 3 H), 3.69 (s, 3 H), 3.66 (s, 3 H), 2.36 - 2.33 (m, 2 H), 2.31 - 2.26 (m, 1 H), 1.23 (s, 3 H), 1.22 (s, 3 H), 1.07 (d, *J* = 6.9 Hz, 3 H), 0.69 (d, *J* = 6.8 Hz, 3 H) ppm.
^{13}**C NMR** (75 MHz, $CDCl_3$): δ = 178.3, 164.0, 162.4, 144.5, 114.8, 62.2, 60.5, 52.6, 52.5, 51.7, 42.6, 42.0, 31.4, 25.8, 25.8, 19.1, 16.5 ppm.
IR (ATR): $\tilde{\nu}$ = 2947, 2872, 1732, 1690, 1460, 1435, 1304, 1235, 1193, 1010 cm^{-1}.
HRMS (EI-TOF) $[M]^+$ calcd. for $C_{17}H_{28}N_2O_4$: 324.2049; found: 324.2054.

ethyl 2-[4-[(2*R*,5*S*)-5-isopropyl-3,6-dimethoxy-2,5-dihydropyrazin-2-yl]phenoxy]-2-methyl-propanoate (**3Aa**)

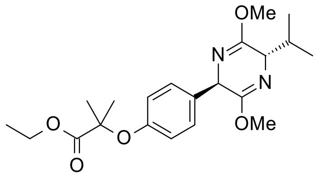

Following general procedure **A** and starting from clofibrate (124 mg, 0.5 mmol), the title compound was obtained as a colorless solid (136 mg, 0.348 mmol, 70%, dr = >25:1).
Before isolation GC-analysis of the crude reaction mixture showed a dr = 12:1.

m.p.: 49-53 °C.
^{1}**H NMR** (300 MHz, $CDCl_3$): δ = 7.12 - 7.07 (m, 2 H), 6.81 - 6.76 (m, 2 H), 5.03 (d, *J* = 3.6 Hz, 1 H), 4.22 (q, *J* = 7.1 Hz, 2 H), 4.07 (dd, *J* = 3.6, 3.4 Hz, 1 H), 3.72 (s, 3 H), 3.62 (s, 3 H), 2.38 (sptd, *J* = 6.8, 3.4 Hz, 1 H), 1.58 (s, 6 H), 1.24 (t, *J* = 7.1 Hz, 3 H), 1.11 (d, *J* = 6.9 Hz, 3 H), 0.75 (d, *J* = 6.8 Hz, 3 H) ppm.
^{13}**C NMR** (75 MHz, $CDCl_3$): δ = 174.3, 164.3, 162.5, 154.7, 134.0, 128.4, 118.8, 79.0, 61.3, 60.5, 59.3, 52.7, 52.6, 31.5, 25.4, 25.3, 19.1, 16.5, 14.0 ppm.
IR (ATR): $\tilde{\nu}$ = 2946, 1730, 1694, 1668, 1508, 1232, 1177, 1144, 970, 731 cm^{-1}.
HRMS (ESI-TOF) $[M + H]^+$ calcd. for $C_{21}H_{31}N_2O_5$: 391.2227; found: 391.2234.

(2*S*,5*R*)-2-isopropyl-3,6-dimethoxy-5-[4-[phenyl-[2-(1-piperidyl)ethoxy]methyl]phenyl]-2,5-dihydropyrazine (**3Ba**)

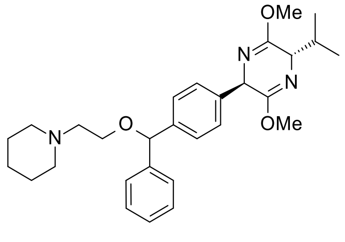

Following general procedure **A** and starting from racemic cloperastine (170 mg, 0.5 mmol), the title compound was obtained as a yellow oil (200 mg, 0.419 mmol, 84%, dr = >25:1).

^{1}H NMR (300 MHz, $CDCl_3$): δ = 7.36 - 7.28 (m, 6 H), 7.26 - 7.16 (m, 3 H), 5.37 (s, 1 H), 5.07 (d, *J* = 3.6 Hz, 1 H), 4.08 (dd, *J* = 3.6, 3.4 Hz, 1 H), 3.71 (s, 3 H), 3.64 - 3.58 (m, 2 H), 3.62 (s, 3 H), 2.70 - 2.65 (m, 2 H), 2.50 - 2.47 (m, 4 H), 2.43 - 2.31 (m, 1 H), 1.59 (quin, *J* = 5.6 Hz, 4 H), 1.47 - 1.39 (m, 2 H), 1.11 (d, *J* = 6.9 Hz, 3 H), 0.76 (d, *J* = 6.8 Hz, 3 H) ppm.
^{13}C NMR (75 MHz, $CDCl_3$): δ = 164.3, 162.3, 142.1, 141.3, 139.5, 139.5, 128.3, 127.6, 127.4, 127.0, 127.0, 126.9, 83.7, 66.8, 60.6, 59.7, 58.4, 54.8, 52.7, 31.5, 25.7, 24.1, 19.1, 16.4 ppm.
Complexity due to stereocenter of racemic cloperastine.
IR (ATR): $\tilde{\nu}$ = 2934, 2869, 1738, 1692, 1493, 1453, 1236, 1193, 1095, 1008 cm^{-1}.
HRMS (ESI-TOF) [M + H]$^+$ calcd. for $C_{29}H_{40}N_3O_3$: 478.3064; found: 478.3057.

ethyl 4-[14-[(2*R*,5*S*)-5-isopropyl-3,6-dimethoxy-2,5-dihydropyrazin-2-yl]-4-azatricyclo[9.4.0.03,8]pentadeca-1(15),3,5,7,11,13-hexaen-2-ylidene]piperidine-1-carboxylate (**3Ca**)

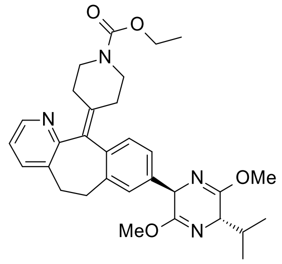

Following general procedure **A** and starting from loratadine (195 mg, 0.5 mmol), the title compound was obtained as a yellow solid (230 mg, 0.433 mmol, 87%, dr = 14:1).

m.p.: 61-67 °C.
^{1}H NMR (300 MHz, $CDCl_3$): δ = 8.40 - 8.38 (m, 1 H), 7.45 - 7.42 (m, 1 H), 7.14 - 6.99 (m, 4 H), 5.03 - 5.01 (m, 1 H), 4.13 (q, *J* = 7.0 Hz, 2 H), 4.10 - 4.07 (m, 1 H), 3.87 - 3.77 (m, 2 H), 3.70 - 3.69 (m, 3 H), 3.62 - 3.62 (m, 3 H), 3.46 - 3.29 (m, 2 H), 3.17 - 3.07 (m, 2 H), 2.90 - 2.78 (m, 2 H), 2.51 - 2.25 (m, 1 H), 2.49 - 2.28 (m, 4 H), 1.24 (t, *J* = 7.1 Hz, 3 H), 1.10 (dd, *J* = 6.8, 1.6 Hz, 3 H), 0.74 (d, *J* = 6.8 Hz, 3 H) ppm.
^{13}C NMR (75 MHz, $CDCl_3$): δ = 164.2, 162.3, 162.2, 155.5, 146.3, 146.3, 139.5, 139.5, 138.1, 137.9, 137.5, 137.4, 137.3, 136.8, 133.8, 133.8, 129.5, 129.4, 128.6, 128.2, 125.5, 125.1, 122.1, 61.2, 60.6, 59.8, 52.7, 52.7, 44.8, 44.8, 31.9, 31.9, 31.6, 31.5, 31.5, 30.7, 30.5, 19.1, 16.4, 14.6 ppm.
Complexity due to ring torsion of the central seven-membered ring leading to conformational diastereomers.[8,9]
IR (ATR): $\tilde{\nu}$ = 2943, 2869, 1689, 1432, 1383, 1221, 1058, 1111, 995, 825 cm^{-1}.
HRMS (ESI-TOF) [M + H]$^+$ calcd. for $C_{31}H_{39}N_4O_4$: 531.2966; found: 531.2956.

methyl (2*R*)-2-amino-2-(4-methoxyphenyl)acetate (**6a**) [CAS: 78307-39-8]

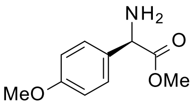

Following general procedure **B** and starting from bis-lactim ether **3aa** (72.6 mg, 0.25 mmol), the title compound was obtained as a yellow oil (39.3 mg, 0.201 mmol, 81%, 96% ee).

[α]$_D$^{20} = –84.5° (c 0.1, EtOH) (lit. [α]$_D$^{20} = –81.7° (c 1, EtOH))[10]
^{1}H NMR (300 MHz, $CDCl_3$): *δ* = 7.33 - 7.28 (m, 2 H), 6.92 - 6.87 (m, 2 H), 4.58 (s, 1 H), 3.81 (s, 3 H), 3.71 (s, 3 H), 1.77 (br. s., 2 H) ppm.
^{13}C NMR (75 MHz, $CDCl_3$): *δ* = 174.7, 159.4, 132.5, 127.9, 114.2, 58.1, 55.3, 52.3 ppm
IR (ATR): $\tilde{\nu}$ = 3382, 2953, 2838, 1734, 1610, 1510, 1437, 1249, 1174, 726 cm^{-1}.
HRMS (ESI-TOF) [M + H]$^+$ calcd. for $C_{10}H_{14}NO_3$: 196.0968; found: 196.0962.
Chiral HPLC (210 nm), Chiralpak® IA:

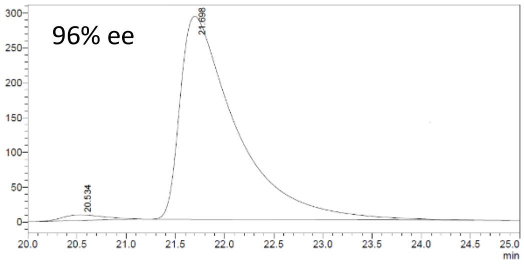

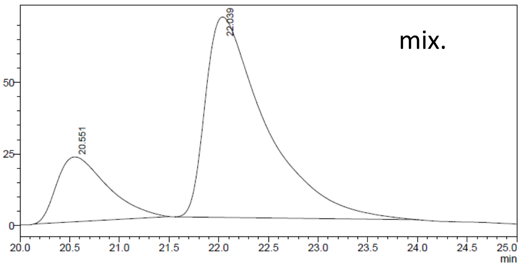

Peak	Time [min]	Area [%]
1	20.534	1.938
2	21.698	98.062

Peak	Time [min]	Area [%]
1	20.551	21.304
2	22.039	78.696

The NMR data are similar to those reported in literature.[11]

methyl (2*R*)-2-amino-2-phenyl-acetate (**6f**) [CAS: 24461-61-8]

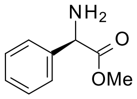

Following general procedure **B** and starting from bis-lactim ether **3fa** (68.6mg, 0.25 mmol), the title compound was obtained as a yellow oil (36.0 mg, 0.218 mmol, 87%, 91% ee).

[α]$_D$^{20} = –198.5 (c 0.1, $CHCl_3$), (lit. [α]$_D$^{20} = 202.3° (c 0.49, $CHCl_3$), 91% ee for *S*-enantiomer)[12]
^{1}H NMR (300 MHz, $CDCl_3$): *δ* = 7.41 - 7.31 (m, 5 H), 4.63 (s, 1 H), 3.71 (s, 3 H), 1.88 (br. s., 2 H) ppm.
^{13}C NMR (75 MHz, $CDCl_3$): *δ* = 174.5, 140.3, 128.8, 128.0, 126.8, 58.7, 52.4 ppm.
IR (ATR): $\tilde{\nu}$ = 3383, 2952,1732, 1601, 1494, 1454, 1435, 1217, 1165, 697 cm^{-1}.
HRMS (ESI-TOF) [M + H]$^+$ calcd. for $C_9H_{12}NO_2$: 166.0863; found: 166.0864.
Chiral HPLC (210 nm), Chiralpak® IA:

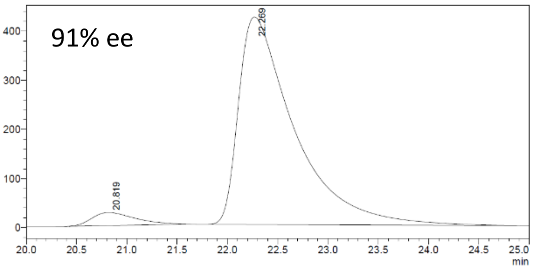

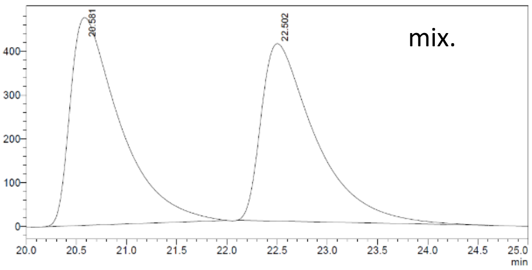

Peak	Time [min]	Area [%]
1	20.819	4.599
2	22.269	95.401

Peak	Time [min]	Area [%]
1	20.581	51.693
2	22.502	48.307

The NMR data are similar to those reported in literature.[13]

methyl (2*R*)-2-amino-2-(4-fluorophenyl)acetate (**6g**) [CAS: 170902-76-8]

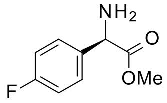

Following general procedure **B** and starting from bis-lactim ether **3ga** (69.6mg, 0.25 mmol), the title compound was obtained as a yellow oil (44.0 mg, 0.240 mmol, 96%, 85% ee).

$[\alpha]_D^{20}$ = –89.1° (c 0.05, EtOH) ($[\alpha]_D^{20}$ = –110.2° (c 1, EtOH) for commercially available substance)
^{1}H NMR (300 MHz, $CDCl_3$): *δ* = 7.40 - 7.33 (m, 2 H), 7.07 - 7.00 (m, 2 H), 4.62 (s, 1 H), 3.71 (s, 3 H), 1.91 (br. s., 2 H) ppm.
^{13}C NMR (75 MHz, $CDCl_3$): *δ* = 174.3, 162.4 (d, *J* = 245.9 Hz), 135.9 (d, *J* = 3.3 Hz), 128.5 (d, *J* = 8.3 Hz), 115.6 (d, *J* = 21.5 Hz), 58.0, 52.4 ppm.
^{19}F NMR (41 MHz, $CDCl_3$): δ = –111.8 - –112.5 (m) ppm.
IR (ATR): $\tilde{\nu}$ = 3384, 2955, 1736, 1682, 1602, 1507, 1437, 1221, 1157, 1089 cm^{-1}.
HRMS (ESI-TOF) $[M + H]^+$ calcd. for $C_9H_{11}FNO_2$: 184.0768; found: 184.0771.
Chiral HPLC (210 nm), Chiralpak® IA:

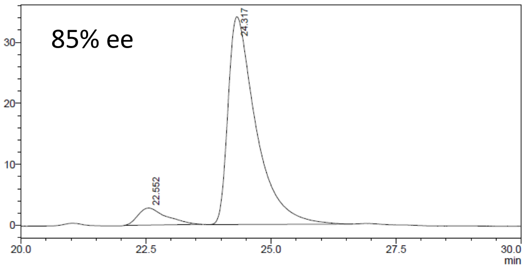

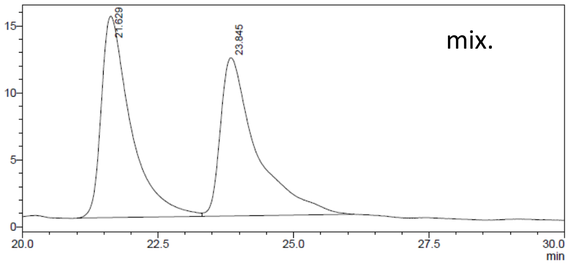

Peak	Time [min]	Area [%]
1	22.552	7.604
2	24.317	92.396

Peak	Time [min]	Area [%]
1	21.629	51.092
2	23.845	48.908

6. Crystal Structure Determination of (*R,S*)-3aa

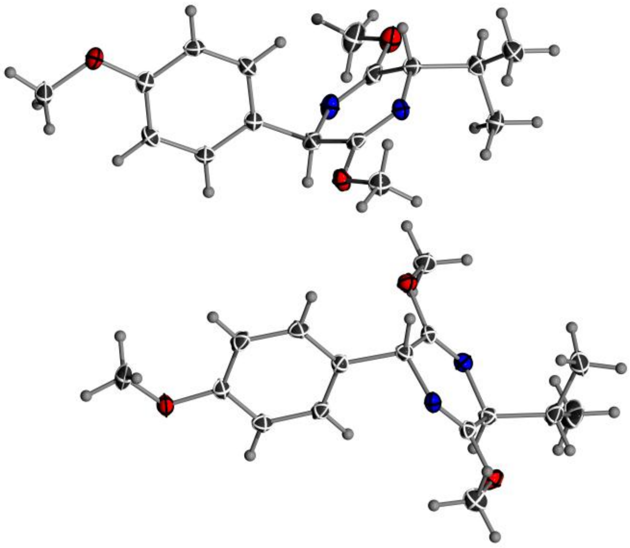

Figure S1. ORTEP plot of (*R,S*)-**3aa**. Ellipsoids are drawn at the 50% probability level.

Single crystals of (*R,S*)-**3aa** were grown by slow evaporation of a saturated solution of (*R,S*)-**3aa** in *n*-hexane. A crystal was taken up in perfluorinated oil and mounted onto a fiber loop on a Rigaku Oxford diffraction XtaLAB Synergy diffractometer equipped with a Hybrid Pixel Array detector. The crystal was kept at 99.98(10) K during data collection. The obtained diffraction data was analyzed using CrysAlisPro software package. Using Olex2,[14] the structure was solved with the ShelXT[15] structure solution program using Intrinsic Phasing and refined with the ShelXL[16] refinement package using Least Squares minimization.

Table S3. Crystal data and structure refinement for **3aa**.

CCDC No.	CCDC 2347922	
Empirical formula	$C_{32}H_{44}N_4O_6$	
Formula weight	580.729	
Temperature	99.98(10) K	
Wavelength	1.54184 Å	
Crystal system	Orthorhombic	
Space group	$P2_12_12_1$	
Unit cell dimensions	a = 7.8893(2) Å	a= 90°.
	b = 9.6176(2) Å	b= 90°.
	c = 41.2385(7) Å	g = 90°.
Volume	3129.02(12) $Å^3$	
Z	4	
Density (calculated)	1.233 Mg/m^3	
Absorption coefficient	0.693 mm^{-1}	
F(000)	1252.237	
Crystal size	0.578 x 0.347 x 0.252 mm^3	
Theta range for data collection	4.29 to 76.88°.	
Index ranges	$-9 \le h \le 9$, $-9 \le k \le 11$, $-42 \le l \le 50$	
Reflections collected	12503	
Independent reflections	5357 [R_{int} = 0.0500]	
Completeness to theta = 67.6840°	99.88 %	
Absorption correction	Semi-empirical from equivalents	
Max. and min. transmission	1.00000 and 0.06843	
Refinement method	Full-matrix least-squares on F^2	
Data / restraints / parameters	5357 / 0 / 389	
Goodness-of-fit on F^2	1.0273	
Final R indices [I>2sigma(I)]	$R1$ = 0.0482, w$R2$ = 0.1298	
R indices (all data)	$R1$ = 0.0514, w$R2$ = 0.1322	
Absolute structure parameter	-0.04(12)	
Largest diff. peak and hole	0.3698 and -0.2523 e.$Å^{-3}$	

Table S4. Atomic coordinates (x 10^4) and equivalent isotropic displacement parameters (Å^{2}x 10^3) for **3aa**. U(eq) is defined as one third of the trace of the orthogonalized U^{ij} tensor.

	x	y	z	U(eq)
O(15)	-2452(2)	1439(2)	3860(1)	21(1)
O(17)	3533(2)	4031(2)	3956(1)	21(1)
O(38)	4590(2)	6909(2)	3538(1)	20(1)
O(34)	10645(2)	8426(2)	2575(1)	21(1)
O(13)	4602(2)	1501(2)	2535(1)	22(1)
O(36)	9317(2)	7629(2)	4456(1)	27(1)
N(26)	5377(2)	8052(2)	4000(1)	18(1)
N(2)	-583(2)	2973(2)	3636(1)	17(1)
N(5)	1735(2)	2409(2)	4166(1)	17(1)
N(23)	8566(2)	6571(2)	3984(1)	21(1)
C(27)	5720(3)	7214(2)	3772(1)	16(1)
C(3)	-949(3)	2116(2)	3861(1)	16(1)
C(7)	2090(3)	3073(2)	3343(1)	16(1)
C(28)	8212(3)	6961(2)	3404(1)	16(1)
C(10)	3810(3)	2084(3)	2796(1)	17(1)
C(6)	2097(3)	3270(2)	3944(1)	16(1)
C(29)	8623(3)	8363(2)	3362(1)	18(1)
C(8)	2745(3)	3979(3)	3114(1)	20(1)
C(31)	9828(3)	7873(2)	2838(1)	17(1)
C(32)	9418(3)	6480(2)	2874(1)	18(1)
C(9)	3596(3)	3511(3)	2841(1)	21(1)
C(30)	9404(3)	8819(2)	3082(1)	19(1)
C(1)	1105(3)	3585(2)	3638(1)	16(1)
C(12)	2343(3)	1656(2)	3297(1)	18(1)
C(33)	8618(3)	6041(3)	3158(1)	18(1)
C(11)	3187(3)	1164(2)	3028(1)	19(1)
C(4)	115(3)	1682(3)	4148(1)	17(1)
C(24)	8226(3)	7441(3)	4207(1)	21(1)
C(25)	6705(3)	8374(3)	4236(1)	19(1)
C(22)	7374(3)	6459(2)	3714(1)	18(1)
C(35)	11034(3)	7495(3)	2315(1)	24(1)
C(40)	5959(3)	8401(3)	4583(1)	22(1)
C(42)	5345(3)	6962(3)	4682(1)	23(1)
C(18)	4626(3)	3756(3)	4227(1)	25(1)
C(19)	-836(3)	1864(3)	4473(1)	23(1)
C(14)	5006(3)	2406(3)	2270(1)	26(1)
C(39)	2967(3)	7581(3)	3563(1)	26(1)
C(16)	-3569(3)	1691(3)	3591(1)	25(1)
C(41)	4565(3)	9477(3)	4610(1)	28(1)
C(21)	-1117(3)	3391(3)	4552(1)	30(1)
C(20)	103(4)	1113(3)	4745(1)	36(1)
C(37)	10907(3)	6917(3)	4439(1)	32(1)

Table S5. Anisotropic displacement parameters (Å^{2}x 10^3) for **3aa**. The anisotropic displacement factor exponent takes the form: $-2\pi^2[h^2a^{*2}U^{11} + ... + 2\ h\ k\ a^*b^*U^{12}]$

	U^{11}	U^{22}	U^{33}	U^{23}	U^{13}	U^{12}
O(15)	16(1)	24(1)	22(1)	2(1)	-2(1)	-2(1)
O(17)	19(1)	24(1)	18(1)	-1(1)	0(1)	-6(1)
O(38)	19(1)	25(1)	16(1)	-2(1)	-2(1)	2(1)
O(34)	23(1)	23(1)	17(1)	2(1)	4(1)	-2(1)
O(13)	25(1)	25(1)	16(1)	-1(1)	6(1)	2(1)
O(36)	17(1)	45(1)	20(1)	0(1)	-4(1)	-2(1)
N(26)	19(1)	20(1)	15(1)	0(1)	0(1)	1(1)
N(2)	16(1)	19(1)	16(1)	-1(1)	2(1)	2(1)
N(5)	17(1)	18(1)	17(1)	1(1)	-1(1)	0(1)
N(23)	21(1)	26(1)	16(1)	5(1)	0(1)	3(1)
C(27)	18(1)	16(1)	15(1)	5(1)	-1(1)	-3(1)
C(3)	15(1)	16(1)	16(1)	-2(1)	1(1)	1(1)
C(7)	15(1)	21(1)	13(1)	1(1)	-1(1)	-2(1)
C(28)	14(1)	18(1)	15(1)	1(1)	-1(1)	1(1)
C(10)	11(1)	25(1)	15(1)	-3(1)	-1(1)	1(1)
C(6)	15(1)	16(1)	16(1)	-4(1)	3(1)	0(1)
C(29)	19(1)	17(1)	17(1)	-4(1)	-1(1)	2(1)
C(8)	24(1)	14(1)	21(1)	1(1)	1(1)	-1(1)
C(31)	12(1)	23(1)	15(1)	3(1)	-2(1)	2(1)

C(32)	20(1)	18(1)	18(1)	-2(1)	0(1)	3(1)
C(9)	23(1)	22(1)	18(1)	5(1)	2(1)	-5(1)
C(30)	19(1)	14(1)	23(1)	-1(1)	-1(1)	-2(1)
C(1)	19(1)	14(1)	15(1)	-1(1)	1(1)	0(1)
C(12)	18(1)	19(1)	16(1)	3(1)	0(1)	-4(1)
C(33)	21(1)	14(1)	20(1)	0(1)	1(1)	-1(1)
C(11)	20(1)	15(1)	21(1)	0(1)	0(1)	0(1)
C(4)	18(1)	17(1)	17(1)	1(1)	0(1)	-1(1)
C(24)	20(1)	27(1)	16(1)	6(1)	-1(1)	-5(1)
C(25)	22(1)	19(1)	17(1)	0(1)	0(1)	-4(1)
C(22)	23(1)	15(1)	17(1)	3(1)	1(1)	0(1)
C(35)	22(1)	32(1)	17(1)	-1(1)	3(1)	0(1)
C(40)	22(1)	25(1)	17(1)	-2(1)	1(1)	-2(1)
C(42)	23(1)	28(1)	18(1)	4(1)	4(1)	1(1)
C(18)	22(1)	32(2)	22(1)	-1(1)	-2(1)	-5(1)
C(19)	22(1)	32(1)	15(1)	2(1)	2(1)	-7(1)
C(14)	22(1)	36(2)	18(1)	2(1)	6(1)	0(1)
C(39)	19(1)	35(2)	23(1)	-1(1)	-4(1)	0(1)
C(16)	22(1)	29(1)	24(1)	0(1)	-7(1)	2(1)
C(41)	33(1)	26(1)	27(1)	-6(1)	4(1)	0(1)
C(21)	29(1)	38(2)	22(1)	-7(1)	3(1)	1(1)
C(20)	42(2)	45(2)	20(1)	10(1)	-2(1)	-6(1)
C(37)	18(1)	56(2)	24(1)	8(1)	-3(1)	1(1)

7. Crystal Structure Determination of [Pip]trYPhos

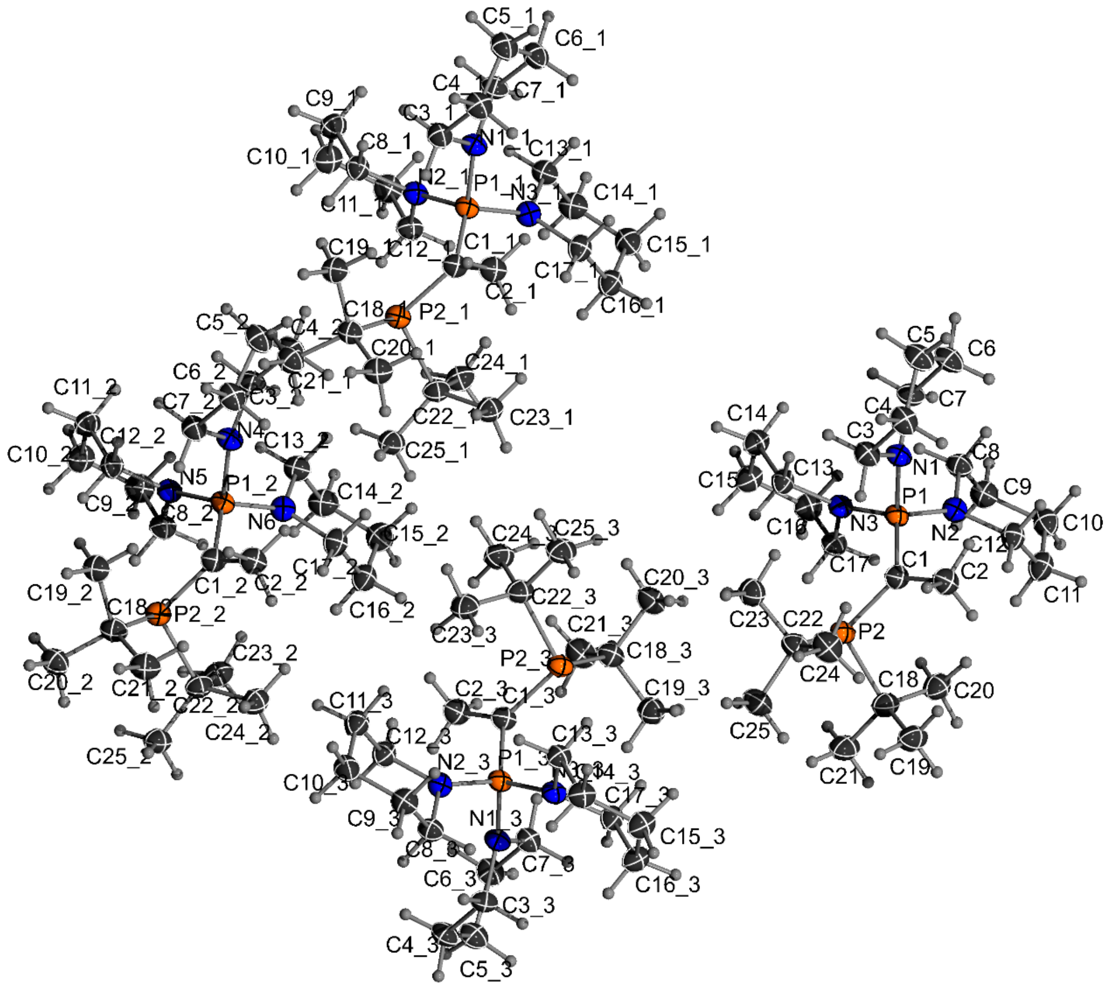

Figure S2. ORTEP plot of **[Pip]trYPhos**. Ellipsoids are drawn at the 50% probability level.

Single crystals of **[Pip]trYPhos** were grown by slow evaporation of a saturated solution of **[Pip]trYPhos** in C_6D_6. A crystal was taken up in perfluorinated oil and mounted onto a fiber loop on a Rigaku Synergy. The crystal was kept at 100(2) K during data collection. The

obtained diffraction data was analyzed using CrysAlisPro software package. Using Olex2,[14] the structure was solved with the ShelXT[15] structure solution program using Intrinsic Phasing and refined with the ShelXL[16] refinement package using Least Squares minimization.

Table S6. Crystal data and structure refinement for **^{Pip}trYPhos**.

CCDC No.	CCDC 2432059	
Empirical formula	C25 H51 N3 P2	
Formula weight	455.62	
Temperature	100(2) K	
Wavelength	1.54184 Å	
Crystal system	Triclinic	
Space group	P-1	
Unit cell dimensions	a = 17.50500(10) Å	a= 66.3050(10)°.
	b = 18.2908(2) Å	b= 68.1190(10)°.
	c = 20.1417(2) Å	g = 72.6290(10)°.
Volume	5395.24(10) Å^3	
Z	8	
Density (calculated)	1.122 Mg/m^3	
Absorption coefficient	1.566 mm^{-1}	
F(000)	2016	
Crystal size	0.340 x 0.260 x 0.240 mm^3	
Theta range for data collection	2.503 to 77.013°.	
Index ranges	-21<=h<=22, -23<=k<=23, -25<=l<=24	
Reflections collected	72972	
Independent reflections	22040 [R(int) = 0.0410]	
Completeness to theta = 67.684°	99.7 %	
Absorption correction	Gaussian	
Max. and min. transmission	0.791 and 0.056	
Refinement method	Full-matrix least-squares on F^2	
Data / restraints / parameters	22040 / 0 / 1109	
Goodness-of-fit on F^2	1.043	
Final R indices [I>2sigma(I)]	R1 = 0.0553, wR2 = 0.1521	
R indices (all data)	R1 = 0.0611, wR2 = 0.1589	
Extinction coefficient	n/a	
Largest diff. peak and hole	0.798 and -0.748 e.Å^{-3}	

Table S7. Atomic coordinates (x 10^4) and equivalent isotropic displacement parameters (Å^{2}x 10^3) for **^{Pip}trYPhos**. U(eq) is defined as one third of the trace of the orthogonalized U^{ij} tensor.

	x	y	z	U(eq)
N(1)	8831(1)	3122(1)	-201(1)	28(1)
C(1)	8590(1)	1570(1)	593(1)	29(1)
N(2)	8586(1)	2318(1)	-954(1)	29(1)
C(2)	9521(1)	1253(1)	526(1)	35(1)
N(3)	7324(1)	2828(1)	3(1)	27(1)
N(4)	3779(1)	5698(1)	7315(1)	29(1)
N(5)	2266(1)	5400(1)	7554(1)	28(1)
N(6)	3546(1)	4699(1)	6741(1)	29(1)
C(3)	8967(1)	3186(1)	451(1)	31(1)
C(4)	9822(1)	3405(1)	234(1)	36(1)
C(5)	9930(1)	4185(1)	-440(1)	39(1)
C(6)	9748(1)	4115(1)	-1094(1)	38(1)
C(7)	8892(1)	3879(1)	-843(1)	31(1)
C(8)	8313(1)	2927(1)	-1604(1)	32(1)
C(9)	8070(1)	2532(1)	-2017(1)	35(1)
C(10)	8799(1)	1902(1)	-2276(1)	37(1)
P(1)	8331(1)	2418(1)	-98(1)	26(1)
C(11)	9099(1)	1294(1)	-1602(1)	38(1)
C(12)	9302(1)	1717(1)	-1184(1)	33(1)
C(13)	6894(1)	3356(1)	469(1)	30(1)
C(14)	6255(1)	4031(1)	113(1)	34(1)
C(15)	5630(1)	3676(1)	19(1)	36(1)
C(16)	6089(1)	3086(1)	-426(1)	34(1)
C(17)	6740(1)	2444(1)	-67(1)	30(1)

C(18)	7948(1)	24(1)	1280(1)	35(1)
C(19)	8629(1)	-587(1)	1622(1)	43(1)
C(20)	8207(1)	102(1)	443(1)	39(1)
P(2)	7737(1)	1105(1)	1294(1)	28(1)
C(21)	7123(1)	-312(1)	1668(1)	44(1)
C(22)	7775(1)	1039(1)	2254(1)	34(1)
C(23)	7395(1)	1899(1)	2290(1)	39(1)
C(24)	8633(1)	812(1)	2393(1)	43(1)
C(25)	7201(1)	474(1)	2901(1)	42(1)
P11	8316(1)	7466(1)	5002(1)	26(1)
N11	8849(1)	8182(1)	4814(1)	28(1)
C11	8471(1)	6704(1)	5793(1)	29(1)
P21	7556(1)	6313(1)	6456(1)	27(1)
N21	7338(1)	7924(1)	5001(1)	28(1)
C21	9356(1)	6427(1)	5874(1)	34(1)
N31	8627(1)	7189(1)	4226(1)	29(1)
C31	8948(1)	8369(1)	5421(1)	31(1)
C41	9827(1)	8529(1)	5215(1)	36(1)
C51	10028(1)	9218(1)	4462(1)	38(1)
C61	9861(1)	9047(1)	3849(1)	36(1)
C71	8979(1)	8866(1)	4104(1)	32(1)
C81	6897(1)	8458(1)	5461(1)	31(1)
C91	6303(1)	9157(1)	5072(1)	36(1)
C101	5686(1)	8839(1)	4929(1)	40(1)
C111	6155(1)	8239(1)	4495(1)	37(1)
C121	6752(1)	7569(1)	4902(1)	31(1)
C131	8488(1)	7712(1)	3491(1)	32(1)
C141	8308(1)	7232(1)	3114(1)	37(1)
C151	9026(1)	6526(1)	3016(1)	39(1)
C161	9179(1)	6002(1)	3783(1)	35(1)
C171	9323(1)	6506(1)	4160(1)	33(1)
C181	7578(1)	6222(1)	7425(1)	31(1)
C191	7739(1)	7045(1)	7333(1)	35(1)
C201	8225(1)	5550(1)	7767(1)	37(1)
C211	6700(1)	6131(1)	7986(1)	37(1)
C221	7678(1)	5239(1)	6460(1)	30(1)
C231	8557(1)	4727(1)	6384(1)	35(1)
C241	7439(1)	5375(1)	5754(1)	36(1)
C251	7048(1)	4763(1)	7146(1)	36(1)
P12	3259(1)	4966(1)	7519(1)	26(1)
C12	3461(1)	4202(1)	8291(1)	29(1)
P22	2582(1)	3764(1)	8978(1)	27(1)
C22	4362(1)	3956(1)	8334(1)	34(1)
C32	3914(1)	6369(1)	6593(1)	32(1)
C42	4790(1)	6560(1)	6343(1)	36(1)
C52	4933(1)	6766(1)	6948(1)	38(1)
C62	4726(1)	6095(1)	7713(1)	35(1)
C72	3856(1)	5913(1)	7914(1)	31(1)
C82	1675(1)	5034(1)	7475(1)	31(1)
C92	1060(1)	5694(1)	7083(1)	37(1)
C102	602(1)	6300(1)	7510(1)	39(1)
C112	1228(1)	6632(1)	7629(1)	36(1)
C122	1833(1)	5938(1)	8010(1)	31(1)
C132	3367(1)	5221(1)	6024(1)	33(1)
C142	3159(1)	4740(1)	5666(1)	38(1)
C152	3883(1)	4048(1)	5538(1)	41(1)
C162	4072(1)	3519(1)	6288(1)	36(1)
C172	4242(1)	4024(1)	6650(1)	34(1)
C182	2656(1)	3646(1)	9938(1)	31(1)
C192	2788(1)	4474(1)	9860(1)	35(1)
C202	1805(1)	3504(1)	10530(1)	38(1)
C212	3347(1)	2990(1)	10230(1)	38(1)
C222	2732(1)	2699(1)	8947(1)	31(1)
C232	2430(1)	2852(1)	8269(1)	37(1)
C242	3630(1)	2226(1)	8806(1)	37(1)
C252	2166(1)	2168(1)	9652(1)	37(1)
P13	6615(1)	142(1)	7559(1)	26(1)
N13	6104(1)	-549(1)	7654(1)	28(1)
C13	6410(1)	981(1)	6836(1)	30(1)
P23	7296(1)	1400(1)	6151(1)	29(1)
N23	6323(1)	275(1)	8405(1)	28(1)
C23	5492(1)	1328(1)	6865(1)	35(1)
N33	7607(1)	-294(1)	7505(1)	27(1)
C33	6023(1)	-1305(1)	8298(1)	31(1)
C43	5155(1)	-1511(1)	8548(1)	35(1)
C53	4959(1)	-1567(1)	7893(1)	37(1)
C63	5092(1)	-792(1)	7212(1)	35(1)
C73	5963(1)	-606(1)	7002(1)	31(1)
C83	6534(1)	-326(1)	9087(1)	31(1)

C93	6768(1)	70(1)	9506(1)	34(1)
C103	6066(1)	749(1)	9702(1)	36(1)
C113	5837(1)	1351(1)	8989(1)	36(1)
C123	5630(1)	920(1)	8582(1)	32(1)
C133	8184(1)	75(1)	7602(1)	30(1)
C143	8799(1)	-578(1)	8000(1)	34(1)
C153	9271(1)	-1182(1)	7569(1)	38(1)
C163	8659(1)	-1519(1)	7441(1)	35(1)
C173	8054(1)	-834(1)	7052(1)	30(1)
C183	7306(1)	1417(1)	5190(1)	34(1)
C193	7670(1)	540(1)	5202(1)	38(1)
C203	7913(1)	1949(1)	4541(1)	41(1)
C213	6465(1)	1646(1)	5014(1)	41(1)
C223	7103(1)	2501(1)	6108(1)	35(1)
C233	6788(1)	2480(1)	6932(1)	40(1)
C243	6474(1)	3106(1)	5696(1)	43(1)
C253	7944(1)	2804(1)	5746(1)	43(1)

8. Crystal Structure Determination of ^{Pip}AdYPhos

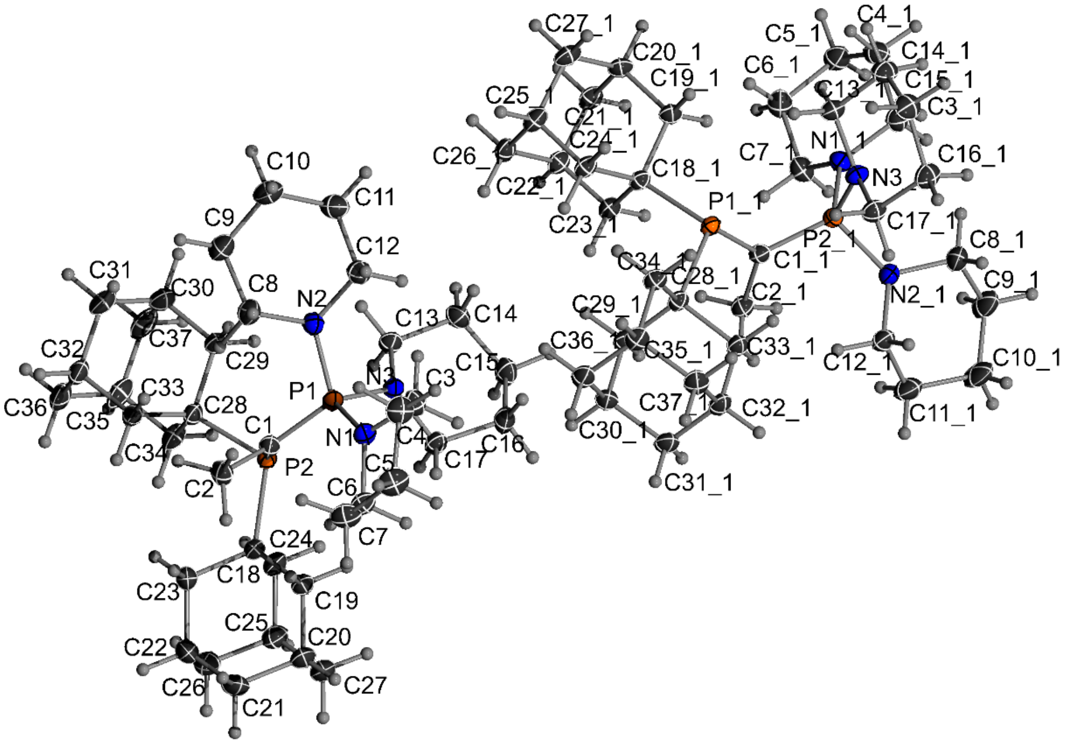

Figure S3. ORTEP plot of ^{Pip}**AdYPhos**. Ellipsoids are drawn at the 50% probability level.

Single crystals of ^{Pip}**AdYPhos** were grown by slow evaporation of a saturated solution of ^{Pip}**AdYPhos** in C_6D_6. A crystal was taken up in perfluorinated oil and mounted onto a fiber loop on a Rigaku Synergy. The crystal was kept at 100(2) K during data collection. The obtained diffraction data was analyzed using CrysAlisPro software package. Using Olex2,[14] the structure was solved with the ShelXT[15] structure solution program using Intrinsic Phasing and refined with the ShelXL[16] refinement package using Least Squares minimization.

Table S8. Crystal data and structure refinement for **^{Pip}adYPhos**.

CCDC No.	CCDC 2432058	
Empirical formula	$C_{37} H_{63} N_3 P_2$	
Formula weight	611.84	
Temperature	100(2) K	
Wavelength l	1.54184 Å (Cu Ka)	
Crystal system	triclinic	
Space group	P -1 (no. 2)	
Unit cell dimensions	a = 14.59150(10) Å	a= 102.6970(10)°
	b = 17.3944(2) Å	b= 97.8470(10)°
	c = 18.46770(10) Å	g = 114.7430(10)°
Volume	4012.35(7) $Å^3$	
Z	4	
Density r(calculated)	1.013 g/cm^3	
Absorption coefficient µ	1.161 mm^{-1}	
F(000)	1344	
Crystal size	0.420 x 0.170 x 0.150 mm^3	
Theta range for data collection	2.538 to 67.999°	
Index ranges	-17 <= h <= 17, -20 <= k< = 20, -22 <= l< = 20	
Reflections collected	50620	
Independent reflections	14574 [Rint = 0.0354, Rsigma = 0.0324]	
Completeness to theta = 67.684°	99.7 %	
Absorption correction	gaussian	
Max. and min. transmission	1.000 and 0.294	
Refinement method	full-matrix least-squares on F^2	
Data / restraints / parameters	14574 / 0 / 759	
Goodness-of-fit on F^2	1.045	
Final R indices [I > 2 s(I)]	R1 = 0.0376, wR2 = 0.0951	
R indices (all data)	R1 = 0.0426, wR2 = 0.0978	
Extinction coefficient	n/a	
Largest diff. peak and hole	0.431 and -0.307 $e^-Å^{-3}$	

Table S9. Atomic coordinates (x 10^4) and equivalent isotropic displacement parameters ($Å^2$x 10^3) for **^{Pip}adYPhos**. U(eq) is defined as one third of the trace of the orthogonalized U^{ij} tensor.

	x	y	z	U(eq)
N1	0.00206(9)	0.31173(8)	0.71262(7)	0.0216(2)
C1	0.09984(11)	0.44342(10)	0.85055(8)	0.0208(3)
C2	-0.00216(11)	0.41558(10)	0.87548(8)	0.0251(3)
N3	0.20929(9)	0.40597(8)	0.74018(6)	0.0194(2)
C3	-0.01893(11)	0.23416(10)	0.64946(8)	0.0265(3)
C4	-0.13001(12)	0.16100(10)	0.63495(9)	0.0302(3)
C5	-0.20855(12)	0.19527(11)	0.61768(10)	0.0345(4)
C6	-0.18108(12)	0.28117(11)	0.67943(10)	0.0311(3)
C7	-0.06741(11)	0.34945(10)	0.69256(9)	0.0244(3)
C8	0.08989(13)	0.26594(11)	0.87572(9)	0.0314(3)
C9	0.16054(16)	0.24706(13)	0.92942(10)	0.0394(4)
C10	0.18858(15)	0.17831(12)	0.88534(10)	0.0378(4)
P1	0.11269(3)	0.36857(2)	0.78242(2)	0.01817(8)
C11	0.23454(13)	0.20807(11)	0.82121(10)	0.0327(4)
C12	0.16117(12)	0.22670(10)	0.76987(9)	0.0264(3)
C13	0.31974(11)	0.43547(10)	0.77652(8)	0.0219(3)
C14	0.37578(12)	0.41961(11)	0.71537(9)	0.0271(3)
C15	0.36543(12)	0.46540(12)	0.65528(9)	0.0301(3)
C16	0.25053(12)	0.43801(11)	0.62215(9)	0.0283(3)
C17	0.19919(11)	0.45457(10)	0.68620(8)	0.0230(3)
C18	0.16011(11)	0.63473(10)	0.88212(8)	0.0214(3)
C19	0.08237(12)	0.60039(10)	0.80293(8)	0.0251(3)

C20	0.04784(13)	0.66989(11)	0.79013(9)	0.0297(3)
C21	-0.00575(14)	0.68987(12)	0.85271(10)	0.0350(4)
C22	0.07172(14)	0.72603(12)	0.93157(10)	0.0340(4)
C23	0.10531(13)	0.65573(11)	0.94388(9)	0.0265(3)
C24	0.25433(12)	0.72176(10)	0.88452(9)	0.0254(3)
C25	0.22020(13)	0.79140(11)	0.87271(9)	0.0307(3)
C26	0.16752(15)	0.81077(11)	0.93542(10)	0.0364(4)
C27	0.14279(13)	0.75508(11)	0.79415(9)	0.0311(3)
C28	0.27130(11)	0.56887(10)	0.98957(8)	0.0207(3)
C29	0.33618(12)	0.51759(11)	0.98375(8)	0.0248(3)
C30	0.39427(13)	0.52709(11)	1.06376(9)	0.0306(3)
C31	0.31585(13)	0.49134(11)	1.11060(9)	0.0300(3)
C32	0.25359(12)	0.54352(11)	1.11834(8)	0.0275(3)
C33	0.19473(11)	0.53180(10)	1.03764(8)	0.0233(3)
C34	0.34796(12)	0.66740(10)	1.03299(8)	0.0263(3)
C35	0.40658(13)	0.67768(11)	1.11323(9)	0.0324(4)
C36	0.32759(14)	0.64198(11)	1.15960(9)	0.0330(4)
C37	0.46882(13)	0.62559(12)	1.10540(9)	0.0360(4)
P2	0.21079(3)	0.55051(2)	0.88486(2)	0.01793(8)
N2	0.13726(9)	0.29382(8)	0.81457(7)	0.0219(2)
P1_1	0.27164(3)	0.11691(2)	0.39572(2)	0.01672(8)
N1_1	0.53087(9)	0.18850(8)	0.32158(7)	0.0201(2)
C1_1	0.35584(10)	0.20199(9)	0.36193(8)	0.0189(3)
P2_1	0.40515(3)	0.16510(2)	0.29234(2)	0.01773(8)
N2_1	0.39623(9)	0.21154(8)	0.22305(7)	0.0218(2)
C2_1	0.38315(11)	0.30054(9)	0.38783(8)	0.0224(3)
N3_1	0.35068(9)	0.05565(8)	0.25019(7)	0.0203(2)
C3_1	0.58889(11)	0.15725(10)	0.27405(9)	0.0241(3)
C4_1	0.66663(12)	0.13897(11)	0.32194(9)	0.0285(3)
C5_1	0.74111(12)	0.22120(11)	0.38889(9)	0.0297(3)
C6_1	0.67969(12)	0.25447(12)	0.43598(9)	0.0302(3)
C7_1	0.60041(11)	0.26940(10)	0.38571(9)	0.0252(3)
C8_1	0.44099(14)	0.20029(12)	0.15752(9)	0.0320(4)
C9_1	0.48645(14)	0.28588(13)	0.13633(11)	0.0393(4)
C10_1	0.40350(15)	0.31547(14)	0.11847(11)	0.0410(4)
C11_1	0.35135(14)	0.32050(11)	0.18485(10)	0.0332(4)
C12_1	0.31099(11)	0.23316(10)	0.20328(9)	0.0249(3)
C13_1	0.37008(11)	-0.00582(9)	0.28585(8)	0.0225(3)
C14_1	0.36005(13)	-0.08541(10)	0.22430(9)	0.0289(3)
C15_1	0.25325(13)	-0.13274(10)	0.16634(9)	0.0309(3)
C16_1	0.23130(13)	-0.06650(10)	0.13445(9)	0.0291(3)
C17_1	0.24459(11)	0.01156(10)	0.19911(8)	0.0244(3)
C18_1	0.34142(10)	0.13962(9)	0.49877(8)	0.0182(3)
C19_1	0.42171(11)	0.10435(10)	0.48846(8)	0.0212(3)
C20_1	0.48531(11)	0.11336(10)	0.56590(8)	0.0239(3)
C21_1	0.54260(11)	0.21099(10)	0.61593(8)	0.0237(3)
C22_1	0.46299(11)	0.24519(10)	0.62856(8)	0.0221(3)
C23_1	0.40184(11)	0.23717(9)	0.55004(8)	0.0203(3)
C24_1	0.26814(11)	0.08453(9)	0.54137(8)	0.0209(3)
C25_1	0.33062(11)	0.09194(10)	0.61906(8)	0.0233(3)
C26_1	0.38792(11)	0.18938(10)	0.66910(8)	0.0238(3)
C27_1	0.41031(12)	0.05774(10)	0.60621(9)	0.0257(3)
C28_1	0.14889(10)	0.13023(9)	0.39530(8)	0.0189(3)
C29_1	0.15299(11)	0.20752(9)	0.45803(8)	0.0211(3)
C30_1	0.04607(11)	0.20725(10)	0.44590(8)	0.0242(3)
C31_1	0.01667(12)	0.21787(11)	0.36683(9)	0.0267(3)
C32_1	0.00953(11)	0.14005(11)	0.30407(9)	0.0261(3)
C33_1	0.11600(11)	0.14039(10)	0.31624(8)	0.0225(3)
C34_1	0.06177(10)	0.04152(9)	0.39774(8)	0.0214(3)
C35_1	-0.04465(11)	0.04156(10)	0.38649(8)	0.0246(3)
C36_1	-0.03718(11)	0.11907(10)	0.44997(9)	0.0255(3)
C37_1	-0.07443(11)	0.05235(11)	0.30788(9)	0.0275(3)

Table S10. Anisotropic displacement parameters (Å^{2}x 10^3) for **^{Pip}adYPhos**. The anisotropic displacement factor exponent takes the form: $-2\pi^2[h^2a^{*2}U^{11} + ... + 2 h k a^*b^*U^{12}]$

	U^{11}	U^{22}	U^{33}	U^{23}	U^{13}	U^{12}
N1	0.0176(6)	0.0253(6)	0.0195(6)	0.0046(5)	0.0022(5)	0.0096(5)
C1	0.0180(6)	0.0266(7)	0.0189(7)	0.0078(6)	0.0051(5)	0.0111(6)
C2	0.0205(7)	0.0319(8)	0.0233(7)	0.0082(6)	0.0070(6)	0.0123(6)
N3	0.0168(5)	0.0247(6)	0.0176(6)	0.0072(5)	0.0041(4)	0.0103(5)
C3	0.0234(7)	0.0314(8)	0.0205(7)	0.0033(6)	0.0035(6)	0.0117(6)
C4	0.0256(8)	0.0263(8)	0.0294(8)	0.0037(6)	0.0013(6)	0.0076(6)
C5	0.0209(7)	0.0330(9)	0.0379(9)	0.0083(7)	-0.0019(7)	0.0061(7)
C6	0.0185(7)	0.0334(8)	0.0394(9)	0.0124(7)	0.0027(6)	0.0109(6)
C7	0.0205(7)	0.0272(7)	0.0238(7)	0.0085(6)	0.0012(6)	0.0104(6)
C8	0.0388(9)	0.0364(9)	0.0323(8)	0.0198(7)	0.0186(7)	0.0220(7)
C9	0.0576(11)	0.0438(10)	0.0307(9)	0.0203(8)	0.0156(8)	0.0306(9)
C10	0.0480(10)	0.0398(9)	0.0377(9)	0.0206(8)	0.0113(8)	0.0271(8)
P1	0.01535(16)	0.02213(17)	0.01666(17)	0.00621(14)	0.00354(13)	0.00842(14)
C11	0.0323(8)	0.0327(8)	0.0403(9)	0.0152(7)	0.0104(7)	0.0193(7)
C12	0.0278(8)	0.0251(7)	0.0286(8)	0.0094(6)	0.0090(6)	0.0134(6)
C13	0.0167(6)	0.0272(7)	0.0225(7)	0.0091(6)	0.0044(5)	0.0104(6)
C14	0.0228(7)	0.0334(8)	0.0314(8)	0.0119(7)	0.0111(6)	0.0165(6)
C15	0.0268(8)	0.0401(9)	0.0291(8)	0.0146(7)	0.0146(6)	0.0165(7)
C16	0.0280(8)	0.0378(9)	0.0214(7)	0.0120(7)	0.0094(6)	0.0151(7)
C17	0.0203(7)	0.0283(7)	0.0223(7)	0.0108(6)	0.0056(6)	0.0115(6)
C18	0.0254(7)	0.0265(7)	0.0175(7)	0.0085(6)	0.0060(6)	0.0160(6)
C19	0.0273(7)	0.0335(8)	0.0199(7)	0.0081(6)	0.0045(6)	0.0194(7)
C20	0.0333(8)	0.0409(9)	0.0245(8)	0.0131(7)	0.0058(6)	0.0249(7)
C21	0.0382(9)	0.0456(10)	0.0410(10)	0.0216(8)	0.0164(8)	0.0314(8)
C22	0.0492(10)	0.0437(10)	0.0297(8)	0.0166(7)	0.0198(7)	0.0348(9)
C23	0.0346(8)	0.0330(8)	0.0218(7)	0.0113(6)	0.0119(6)	0.0219(7)
C24	0.0289(8)	0.0287(8)	0.0217(7)	0.0111(6)	0.0056(6)	0.0149(6)
C25	0.0391(9)	0.0296(8)	0.0295(8)	0.0145(7)	0.0090(7)	0.0188(7)
C26	0.0572(11)	0.0326(9)	0.0291(8)	0.0100(7)	0.0103(8)	0.0297(8)
C27	0.0410(9)	0.0396(9)	0.0284(8)	0.0182(7)	0.0122(7)	0.0281(8)
C28	0.0209(7)	0.0259(7)	0.0168(7)	0.0087(6)	0.0043(5)	0.0114(6)
C29	0.0249(7)	0.0335(8)	0.0209(7)	0.0103(6)	0.0052(6)	0.0174(6)
C30	0.0299(8)	0.0420(9)	0.0272(8)	0.0151(7)	0.0042(6)	0.0220(7)
C31	0.0340(8)	0.0349(8)	0.0236(8)	0.0147(7)	0.0028(6)	0.0170(7)
C32	0.0310(8)	0.0360(8)	0.0199(7)	0.0141(6)	0.0080(6)	0.0163(7)
C33	0.0236(7)	0.0304(8)	0.0194(7)	0.0114(6)	0.0064(6)	0.0136(6)
C34	0.0291(8)	0.0267(8)	0.0191(7)	0.0088(6)	0.0026(6)	0.0096(6)
C35	0.0346(9)	0.0299(8)	0.0206(8)	0.0081(6)	-0.0036(6)	0.0070(7)
C36	0.0446(10)	0.0380(9)	0.0158(7)	0.0085(7)	0.0030(7)	0.0198(8)
C37	0.0252(8)	0.0502(10)	0.0263(8)	0.0178(8)	-0.0025(6)	0.0117(7)
P2	0.01803(16)	0.02270(17)	0.01498(16)	0.00669(13)	0.00400(13)	0.01082(14)
N2	0.0219(6)	0.0247(6)	0.0217(6)	0.0094(5)	0.0076(5)	0.0115(5)
P1_1	0.01502(16)	0.01876(17)	0.01724(17)	0.00751(13)	0.00331(12)	0.00790(13)
N1_1	0.0179(6)	0.0207(6)	0.0213(6)	0.0063(5)	0.0052(5)	0.0087(5)
C1_1	0.0191(6)	0.0193(7)	0.0207(7)	0.0091(5)	0.0051(5)	0.0096(5)
P2_1	0.01771(16)	0.01906(17)	0.01747(17)	0.00767(13)	0.00434(13)	0.00855(13)
N2_1	0.0241(6)	0.0264(6)	0.0202(6)	0.0113(5)	0.0076(5)	0.0140(5)
C2_1	0.0248(7)	0.0204(7)	0.0237(7)	0.0096(6)	0.0077(6)	0.0101(6)
N3_1	0.0194(6)	0.0207(6)	0.0196(6)	0.0065(5)	0.0021(5)	0.0090(5)
C3_1	0.0241(7)	0.0259(7)	0.0253(7)	0.0095(6)	0.0099(6)	0.0126(6)
C4_1	0.0269(8)	0.0308(8)	0.0366(9)	0.0160(7)	0.0127(7)	0.0172(7)
C5_1	0.0210(7)	0.0407(9)	0.0318(8)	0.0161(7)	0.0080(6)	0.0155(7)
C6_1	0.0210(7)	0.0412(9)	0.0251(8)	0.0081(7)	0.0046(6)	0.0130(7)
C7_1	0.0198(7)	0.0239(7)	0.0263(8)	0.0031(6)	0.0046(6)	0.0076(6)
C8_1	0.0390(9)	0.0450(10)	0.0243(8)	0.0167(7)	0.0132(7)	0.0263(8)
C9_1	0.0393(9)	0.0573(11)	0.0411(10)	0.0342(9)	0.0219(8)	0.0277(9)
C10_1	0.0439(10)	0.0535(11)	0.0478(11)	0.0390(9)	0.0222(8)	0.0282(9)
C11_1	0.0376(9)	0.0353(9)	0.0365(9)	0.0204(7)	0.0107(7)	0.0208(7)
C12_1	0.0244(7)	0.0308(8)	0.0239(7)	0.0135(6)	0.0059(6)	0.0145(6)
C13_1	0.0229(7)	0.0212(7)	0.0232(7)	0.0085(6)	0.0030(6)	0.0102(6)
C14_1	0.0340(8)	0.0243(7)	0.0293(8)	0.0073(6)	0.0051(7)	0.0158(7)
C15_1	0.0383(9)	0.0220(7)	0.0260(8)	0.0016(6)	0.0016(7)	0.0131(7)
C16_1	0.0320(8)	0.0266(8)	0.0228(7)	0.0045(6)	0.0005(6)	0.0118(7)

C17_1	0.0230(7)	0.0244(7)	0.0230(7)	0.0064(6)	0.0010(6)	0.0103(6)
C18_1	0.0158(6)	0.0202(7)	0.0188(7)	0.0080(5)	0.0034(5)	0.0079(5)
C19_1	0.0193(7)	0.0251(7)	0.0210(7)	0.0078(6)	0.0034(5)	0.0121(6)
C20_1	0.0203(7)	0.0294(8)	0.0250(7)	0.0094(6)	0.0022(6)	0.0148(6)
C21_1	0.0170(7)	0.0286(8)	0.0225(7)	0.0100(6)	0.0011(5)	0.0081(6)
C22_1	0.0207(7)	0.0222(7)	0.0186(7)	0.0054(6)	0.0005(5)	0.0073(6)
C23_1	0.0197(7)	0.0206(7)	0.0197(7)	0.0075(6)	0.0033(5)	0.0085(6)
C24_1	0.0170(6)	0.0230(7)	0.0211(7)	0.0101(6)	0.0030(5)	0.0068(6)
C25_1	0.0192(7)	0.0270(7)	0.0221(7)	0.0130(6)	0.0033(6)	0.0071(6)
C26_1	0.0219(7)	0.0321(8)	0.0180(7)	0.0098(6)	0.0032(5)	0.0125(6)
C27_1	0.0256(7)	0.0249(7)	0.0240(7)	0.0096(6)	-0.0020(6)	0.0108(6)
C28_1	0.0155(6)	0.0225(7)	0.0187(7)	0.0082(5)	0.0032(5)	0.0085(5)
C29_1	0.0197(7)	0.0246(7)	0.0215(7)	0.0093(6)	0.0053(5)	0.0117(6)
C30_1	0.0237(7)	0.0313(8)	0.0242(7)	0.0107(6)	0.0077(6)	0.0171(6)
C31_1	0.0247(7)	0.0358(8)	0.0296(8)	0.0160(7)	0.0088(6)	0.0198(7)
C32_1	0.0232(7)	0.0406(9)	0.0224(7)	0.0151(7)	0.0054(6)	0.0193(7)
C33_1	0.0213(7)	0.0308(8)	0.0202(7)	0.0111(6)	0.0056(5)	0.0149(6)
C34_1	0.0171(7)	0.0229(7)	0.0230(7)	0.0086(6)	0.0033(5)	0.0080(6)
C35_1	0.0159(7)	0.0300(8)	0.0266(8)	0.0106(6)	0.0041(6)	0.0090(6)
C36_1	0.0197(7)	0.0370(8)	0.0258(8)	0.0144(7)	0.0078(6)	0.0156(6)
C37_1	0.0182(7)	0.0380(9)	0.0248(8)	0.0078(7)	0.0013(6)	0.0140(6)

9. Competition Experiments

General procedure: An oven-dried vial (20 mL) was charged with $[Pd(1\text{-MeNAP})Br]_2$ (4.09 mg, 0.00625 mmol, 2.5 mol%) and ^{Pip}AdYPhos (7.65 mg, 0.0125 mmol, 5 mol%) in a nitrogen filled glovebox. To this reaction vial, a stock solution containing 2-MeTHF (2.0 mL) the corresponding aryl chlorides (0.25 mmol, 1.00 equiv.) and *n*-hexadecane (20 µL) as internal standard were added. To ensure full deprotonation and zincation of the corresponding bis-lactim ethers, LiTMP (73.6 mg, 0.50 mmol, 2.00 equiv.), $ZnCl_2$ (68.1 mg, 0.50 mmol, 2.00 equiv.) and bis-lactim ether (0.50 mmol, 2.00 equiv.) were prestirred for 16 hours at room temperature using 2-MeTHF (0.5 mL) as solvent. This mixture was added to the vial containing catalyst, solvent, aryl chloride and internal standard, which was subsequently stirred for 2 hours at room temperature. After 5 min, 10 min, 20 min, 40 min, 60 min and 120 min small aliquots (0.2 mL) were removed from the reaction mixture, quenched with 0.5 mL of MeOH and analyzed by GC analysis.

Competition experiments between aryl chlorides: For kinetic studies involving aryl chlorides **1a** and **1l**, three stock solutions were prepared. Stock solution **I** was prepared by combining 1.0 equiv. of **1a** with 1.0 equiv. of **1l** in the same mixture. Stock solutions **II** and **III** each contained 1.0 equiv. of either **1a** or **1l**, respectively. The initial amount of aryl chloride n_0(ArCl) was normalized to 0.25 mmol by calibrating against the ratio of aryl chloride to internal standard. The decay of aryl chloride n(ArCl) was monitored in parallel experiments and in a one-pot experiment by removing small aliquots (0.2 mL) from the reaction mixture, quenching it with 0.5 mL of MeOH and analyzing it by GC analysis. The concentration was determined by dividing n(ArCl) by the amount of solvent in the reaction mixture: 2 mL (2-MeTHF from stock solution **I-III**) + 0.5 mL (base solution) = 2.5 mL (overall solvent).

1) One-pot experiment

t (min)	0	5	10	20	40	60	120
n(**1a**) (mmol)	0,25	0,1505	0,138475	0,13855	0,136425	0,1381	0,1368
c(**1a**) (mol/L)	0,1	0,0602	0,05539	0,05542	0,05457	0,05524	0,05472
n(**1l**) (mmol)	0,25	0,097975	0,0755	0,072175	0,071375	0,069125	0,068325
c(**1l**) (mol/L)	0,1	0,03919	0,0302	0,02887	0,02855	0,02765	0,02733

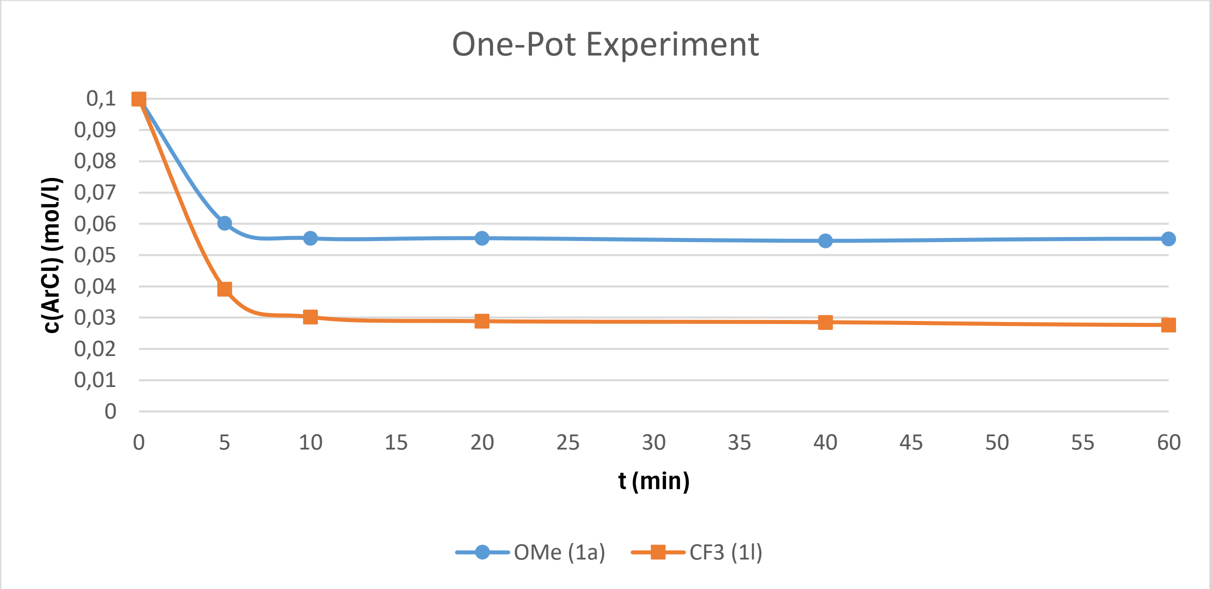

The one-pot experiment shows two competing reactions, which go to completion within the first 10 min, so that the data points at 60 min definitely mark the reaction endpoint. Both reactions influence the depletion of bis-lactim ether **2a**:

$$\mathbf{1a} + \mathbf{2a} \rightarrow \mathbf{3aa} \text{ (with rate } k_{1a})$$
$$\mathbf{1l} + \mathbf{2a} \rightarrow \mathbf{3la} \text{ (with rate } k_{1l})$$

The initial concentrations of the reagents are:

$$[\mathbf{1a}]_0 = [\mathbf{1}\boldsymbol{l}]_0 = a_0 = 0.1 \text{ mol/l}$$
$$[\mathbf{2a}]_0 = c_0 = 0.2 \text{ mol/l}$$

We define the progress of reaction as follows:

$$[\mathbf{1a}] = a_0 - x \text{ (with x = amount of reacted } \mathbf{1a} \text{ to product } \mathbf{3aa})$$
$$[\mathbf{1}\boldsymbol{l}] = a_0 - y \text{ (with y = amount of reacted } \mathbf{1l} \text{ to product } \mathbf{3la})$$
$$[\mathbf{2}\boldsymbol{a}] = c_0 - x - y$$

Experiments with different amounts of bis-lactim ether **2a** (Table S2) showed that the conversion is influenced by the concentration of ArCl and bis-lactim ether **2a**. This suggests that the reaction follows second order kinetics. The second order rate laws are:

$$\frac{dx}{dt} = k_{1a}[\mathbf{1a}][\mathbf{2a}] = k_{1a}(a_0 - x)(c_0 - x - y)$$
$$\frac{dy}{dt} = k_{1l}[\mathbf{1l}][\mathbf{2a}] = k_{1l}(a_0 - y)(c_0 - x - y)$$

If we divide both rate laws we would be able to determine the ratio between k_{1a} and k_{1l}. The shared term for the concentration of **2a** would be canceled out in both cases facilitating the analysis of the data points:

$$\frac{dx}{dy} = \frac{k_{1a}(a_0 - x)}{k_{1l}(a_0 - y)}$$

Separation of variables and integration on both sites from 0 to x and 0 to y, respectively:

$$\frac{dx}{a_0 - x} = \frac{k_{1a}}{k_{1l}} \cdot \frac{dy}{a_0 - y}$$

$$\int_0^x \frac{dx}{a_0 - x} = \frac{k_{1a}}{k_{1l}} \cdot \int_0^y \frac{dy}{a_0 - y}$$

$$-\ln(a_0 - x) + \ln(a_0) = \frac{k_{1a}}{k_{1l}} \cdot (-\ln(a_0 - y) + \ln(a_0))$$

$$\ln\left(\frac{a_0}{a_0 - x}\right) = \frac{k_{1a}}{k_{1l}} \cdot \ln\left(\frac{a_0}{a_0 - y}\right)$$

The ratio of k_{1a} to k_{1l} can be calculated with the following equation:

$$\frac{k_{1a}}{k_{1l}} = \frac{\ln\left(\frac{a_0}{a_0 - x}\right)}{\ln\left(\frac{a_0}{a_0 - y}\right)} = \frac{\ln\left(\frac{a_0}{[\mathbf{1a}]}\right)}{\ln\left(\frac{a_0}{[\mathbf{1l}]}\right)}$$

With a_0 = 0.1 mol/l, [**1a**](120 min) = 0.05472 mol/l and [**1l**](120 min) = 0.02733 mol/l after 120 minutes:

$$\frac{k_{1a}}{k_{1l}} \approx 0.464$$

or

$$\frac{k_{1l}}{k_{1a}} \approx 2.2$$

The one-pot experiment between electron-deficient aryl chloride **1l** and electron-rich aryl chloride **1a** shows a ratio in rates of approximately k_{1l}:k_{1a} = 2.2:1.

2) Parallel experiments

t (min)	0	5	10	20	40	60	120
n(**1a**) (mmol)	0,25	0,128525	0,079025	0,03435	0,0025	0,0025	0,0025
c(**1a**) (mol/L)	0,1	0,05141	0,03161	0,01374	0,001	0,001	0,001
n(**1l**) (mmol)	0,25	0,10425	0,0539	0,015	0,0025	0,0025	0,0025
c(**1l**) (mol/L)	0,1	0,0417	0,02156	0,006	0,001	0,001	0,001

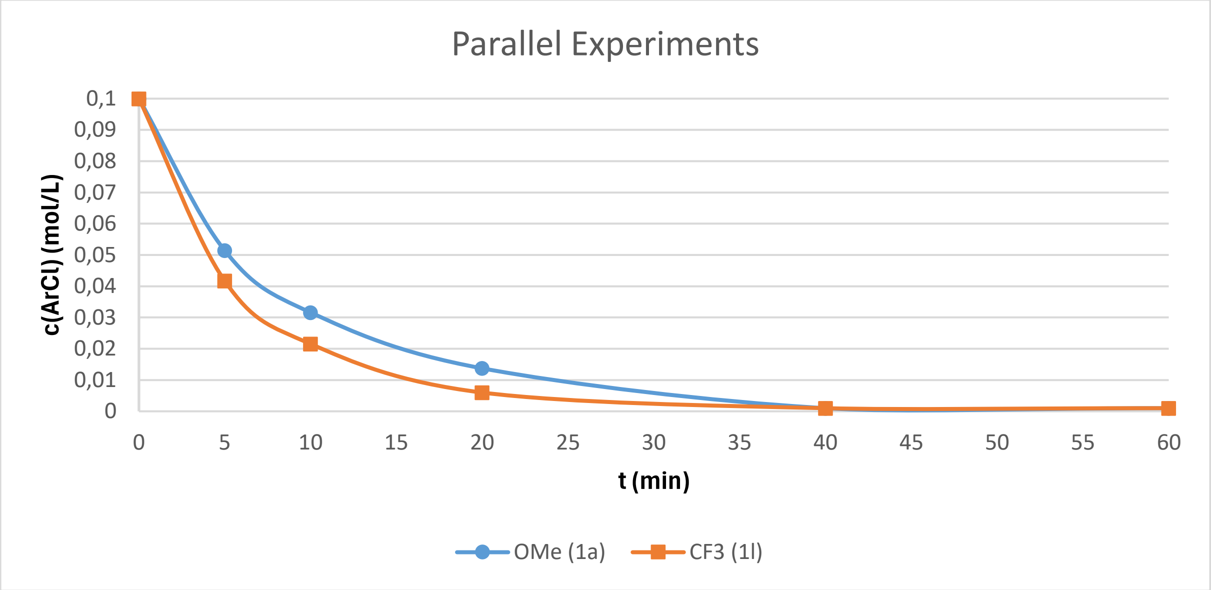

Parallel experiments show full conversion of the corresponding aryl chloride after 60 minutes. The reaction between **1a** or **1l** with **2a** is:

$$\mathbf{1a} + \mathbf{2a} \rightarrow \mathbf{3aa}\ \text{(with rate } k_{1a}\text{)}$$
$$\mathbf{1l} + \mathbf{2a} \rightarrow \mathbf{3la}\ \text{(with rate } k_{1l}\text{)}$$

Experiments with different amounts of bis-lactim ether **2a** (Table S2) showed that the conversion is influenced by the concentration of ArCl and bis-lactim ether **2a**. This suggests that the reaction follows second order kinetics:

$$\frac{d[\mathbf{1a}]}{dt} = -k_{1a}[\mathbf{1a}][\mathbf{2a}]\ \text{(for the reaction with stock solution } \mathbf{II}\text{)}$$

$$\frac{d[\mathbf{1l}]}{dt} = -k_{1l}[\mathbf{1l}][\mathbf{2a}]\ \text{(for the reaction with stock solution } \mathbf{III}\text{)}$$

For simplification we considered only the first three data points (early- to mid-stage) and approximated the reaction as a pseudo-first-order reaction:

$$\frac{d[\mathbf{1a}]}{dt} = -k'_{1a}[\mathbf{1a}]\ \text{ with }\ k'_{1a} = k_{1a}[\mathbf{2}\boldsymbol{a}]$$

$$\frac{d[\mathbf{1l}]}{dt} = -k'_{1l}[\mathbf{1l}]\ \text{ with }\ k'_{1l} = k_{1l}[\mathbf{2}\boldsymbol{a}]$$

Integrated form for pseudo-first order kinetics is:

$$[\mathbf{1a}](t) = [\mathbf{1a}]_0 \cdot e^{-k'_{1a}\cdot \mathbf{t}}\ \text{(for the reaction with stock solution } \mathbf{II}\text{)}$$

$$[\mathbf{1l}](t) = [\mathbf{1l}]_0 \cdot e^{-k'_{1l}\cdot \mathbf{t}}\ \text{(for the reaction with stock solution } \mathbf{III}\text{)}$$

Taking the natural log on both sites:

$$\ln([\mathbf{1a}](t)) = \ln([\mathbf{1a}]_0) - k'_{1a} \cdot t\ \text{(for the reaction with stock solution } \mathbf{II}\text{)}$$

$$\ln([\mathbf{1l}](t)) = \ln([\mathbf{1l}]_0) - k'_{1l} \cdot t\ \text{(for the reaction with stock solution } \mathbf{III}\text{)}$$

Plotting the ln([ArCl](t)) vs. t (min) for early to mid-stage data points should give a straight line with slope = $-$ k'_{ArCl}.

t (min)	0	5	10	20	40	60	120
ln(c(**1a**))	-2,302585	-2,967922	-3,454281	-4,287443	-6,907755	-6,907755	-6,907755
ln(c(**1l**))	-2,302585	-3,177254	-3,836915	-5,115995	-6,907755	-6,907755	-6,907755

For the linear fit only the data points within the first 20 minutes were considered:

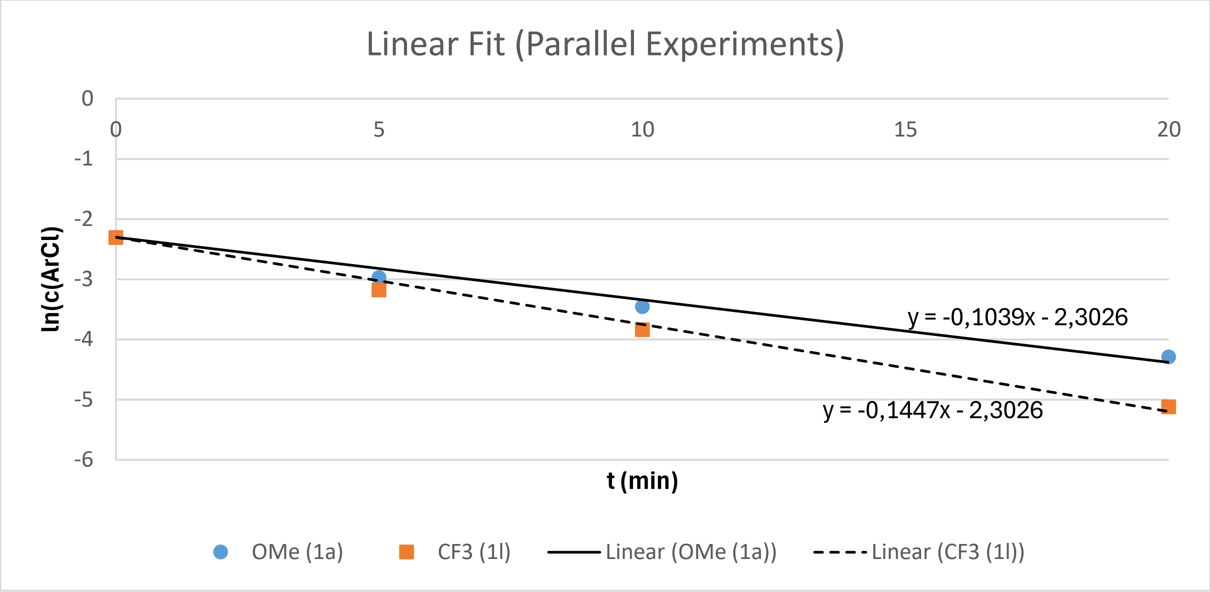

The parallel reaction of aryl chloride **1a** with **2a** results in k'_{1a} = 0.1039 min^{-1}, while the parallel experiment of **1l** with **2a** yields k'_{1l} = 0.1447 min^{-1}.

The ratio of both reaction rates is defined as follows:

$$\frac{k_{1a}}{k_{1l}} = \frac{k'_{1a}\,[\mathbf{2a}]_0}{k'_{1l}\,[\mathbf{2a}]_0} = \frac{k'_{1a}}{k'_{1l}} \approx 0.718$$

or

$$\frac{k_{1l}}{k_{1a}} \approx 1.4$$

In the parallel experiments the reaction rates in the reaction of electron-deficient aryl chloride **1l** and bis-lactim ether **2a** and electron-rich aryl chloride **1a** and bis-lactim ether **2a** show a ratio of approximately k_{1l}:k_{1a} = 1.4:1.

Competition experiments between bis-lactim ethers: For kinetic studies involving bis-lactim ether **2a** and **2b**, two distinct base solutions were prepared. Base solutions **I** and **II** each contained 2.0 equivalent of either **2a** or **2b**, respectively. The one-pot experiment was started by simultaneously adding 1.0 equivalent of **2a** solution and 1.0 equivalent of **2b** solution to the reaction mixture. The initial amount of aryl chloride n_0(ArCl) was normalized to 0.25 mmol by calibrating against the ratio of aryl chloride to internal standard. The amount of remaining aryl chloride n(ArCl) was monitored in parallel experiments and in a one-pot experiment. For the one-pot experiment the amount of remaining aryl chloride n(ArCl) was extrapolated from the yield of the formed bis-lactim ether product **3aa** or **3ab**. The concentration was determined by dividing n(ArCl) by the amount of solvent in the reaction mixture: 2 mL (2-MeTHF from stock solution **I-III**) + 0.5 mL (base solution) = 2.5 mL (overall solvent).

1) One-pot experiment

t (min)	0	5	10	20	40	60	120
yield **3aa** (%)	0	30,23	36,26	39,62	40,46	39,73	39,01
yield **3ab** (%)	0	47,16	53,7	57,09	57,59	56,5	57,53
$[\mathbf{1a}]_{\mathbf{3aa}}$ (mol/l) extrapolated from formation of **3aa**	0,1	0,06977	0,06374	0,06038	0,05954	0,06027	0,06099
$[\mathbf{1a}]_{\mathbf{3ab}}$ (mol/l) extrapolated from formation of **3ab**	0,1	0,05284	0,0463	0,04291	0,04241	0,0435	0,04247

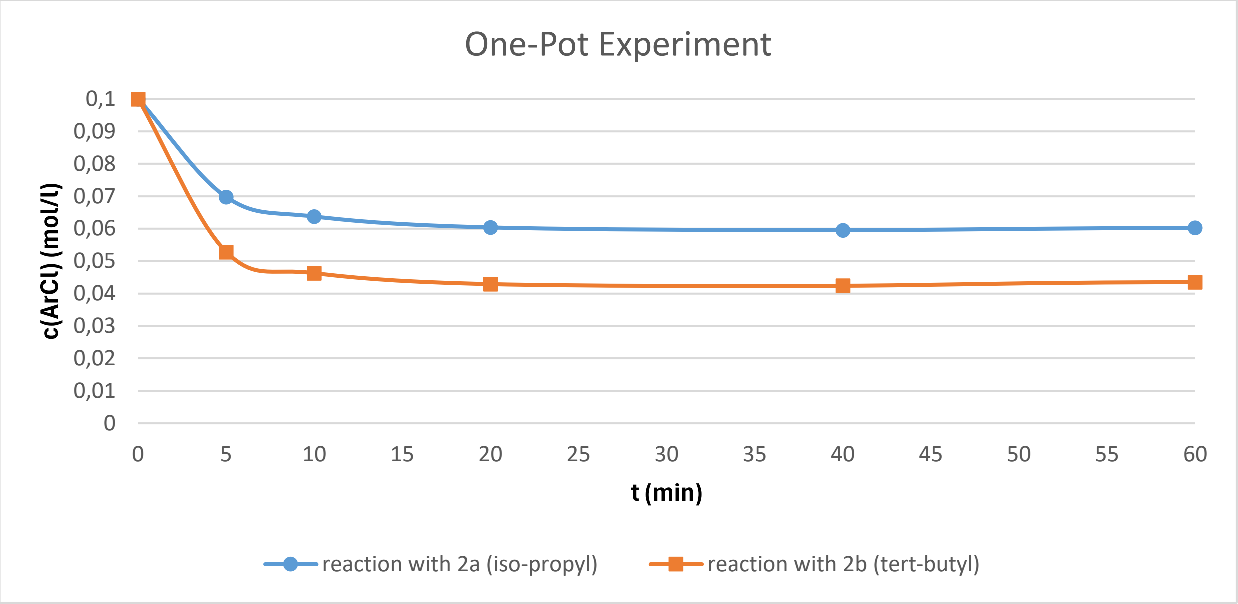

The one-pot experiment shows two competing reactions, which equilibrate after 20 min. Both reactions influence the depletion of aryl chloride **1a**:

$$\mathbf{1a} + \mathbf{2a} \rightarrow 3\mathbf{aa}\ (\text{with rate } k_{2a})$$
$$\mathbf{1a} + \mathbf{2b} \rightarrow \mathbf{3ab}\ (\text{with rate } k_{2b})$$

The initial concentrations of the reagents are:

$$[\mathbf{1a}]_0 = [\mathbf{2}a]_0 = [\mathbf{2b}]_0 = a_0 = 0.1\ \text{mol/l}$$

We define the extent of reaction as follows:

$$[\mathbf{2a}] = a_0 - x\ (\text{with } x = \text{amount of reacted } \mathbf{1a} \text{ to product } \mathbf{3aa})$$
$$[\mathbf{2b}] = a_0 - y\ (\text{with } y = \text{amount of reacted } \mathbf{1a} \text{ to product } \mathbf{3ab})$$
$$[\mathbf{1a}] = a_0 - x - y$$

Experiments with different amounts of bis-lactim ether **2a** (Table S2) showed that the conversion is influenced by the concentration of ArCl and bis-lactim ether **2a**. This suggests that the reaction follows second order kinetics. The second order rate laws are:

$$\frac{dx}{dt} = k_{2a}[\mathbf{1a}][\mathbf{2a}] = k_{1a}(a_0 - x - y)(a_0 - x)$$
$$\frac{dy}{dt} = k_{2b}[\mathbf{1a}][\mathbf{2a}] = k_{1l}(a_0 - x - y)(a_0 - y)$$

If we divide both rate laws we would be able to determine the ratio between k_{2a} and k_{2b}. The shared term for the concentration of **2a** would be canceled out in both cases facilitating the analysis of the data points:

$$\frac{dx}{dy} = \frac{k_{2a}(a_0 - x)}{k_{2b}(a_0 - y)}$$

Separation of variables and integration on both sites from 0 to x and 0 to y, respectively:

$$\frac{dx}{a_0 - x} = \frac{k_{2a}}{k_{2b}} \cdot \frac{dy}{a_0 - y}$$

$$\int_0^x \frac{dx}{a_0 - x} = \frac{k_{2a}}{k_{2b}} \cdot \int_0^y \frac{dy}{a_0 - y}$$

$$-\ln(a_0 - x) + \ln(a_0) = \frac{k_{2a}}{k_{2b}} \cdot (-\ln(a_0 - y) + \ln(a_0))$$

$$\ln\left(\frac{a_0}{a_0 - x}\right) = \frac{k_{2a}}{k_{2b}} \cdot \ln\left(\frac{a_0}{a_0 - y}\right)$$

The ratio of k_{2a} to k_{2b} can be calculated with the following equation:

$$\frac{k_{2a}}{k_{2b}} = \frac{\ln\left(\frac{a_0}{a_0 - x}\right)}{\ln\left(\frac{a_0}{a_0 - y}\right)} = \frac{\ln\left(\frac{a_0}{[\mathbf{1a}]_{3aa}}\right)}{\ln\left(\frac{a_0}{[\mathbf{1a}]_{3ab}}\right)}$$

With a_0 = 0.1 mol/l, $[\mathbf{1a}]_{\mathbf{3aa}}$ = 0.06099 mol/l and $[\mathbf{1a}]_{\mathbf{3ab}}$ = 0.04247 mol/l after 120 minutes:

$$\frac{k_{2a}}{k_{2b}} \approx 0.577$$

or

$$\frac{k_{2b}}{k_{2a}} \approx 1.7$$

The one-pot experiment between aryl chloride **1a** with bis-lactim ether **2a** and **2b** shows a ratio in rates of approximately k_{2b}:k_{2a} = 1.7:1.

2) Parallel experiments

t (min)	0	5	10	20	40	60	120
c(**1a**) (mol/l) from the reaction with **2a**	0,1	0,05141	0,03161	0,01374	0,001	0,001	0,001
c(**1a**) (mol/l) from the reaction with **2b**	0,1	0,02592	0,01308	0,004	0,001	0,001	0,001

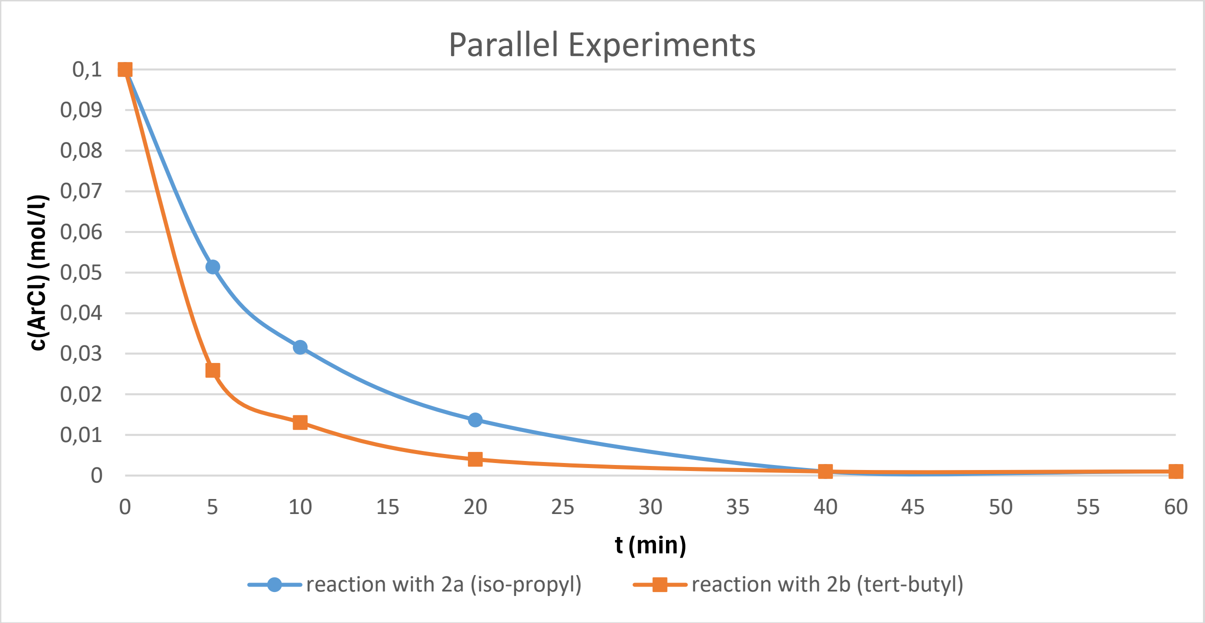

Parallel experiments show full conversion of the corresponding aryl chloride after 60 minutes. The reaction between **1a** with **2a** or **2b** is:

$$\mathbf{1a} + \mathbf{2a} \rightarrow \mathbf{3aa} \text{ (with rate } k_{2a})$$
$$\mathbf{1a} + \mathbf{2b} \rightarrow \mathbf{3ab} \text{ (with rate } k_{2b})$$

Experiments with different amounts of bis-lactim ether **2a** (Table S2) showed that the conversion is influenced by the concentration of ArCl and bis-lactim ether **2a**. This suggests that the reaction follows second order kinetics:

$$\frac{d[\mathbf{1a}]}{dt} = -k_{2a}[\mathbf{1a}][\mathbf{2a}] \text{ (for the reaction with base solution } \mathbf{I})$$

$$\frac{d[\mathbf{1a}]}{dt} = -k_{2b}[\mathbf{1a}][\mathbf{2b}] \text{ (for the reaction with stock solution } \mathbf{II})$$

For simplification we considered only the first three data points (early- to mid-stage) and approximated the reaction as a pseudo-first-order reaction:

$$\frac{d[\mathbf{1a}]}{dt} = -k'_{2a}[\mathbf{1a}] \text{ with } k'_{1a} = k_{2a}[\boldsymbol{2a}]$$

$$\frac{d[\mathbf{1a}]}{dt} = -k'_{2b}[\mathbf{1a}] \text{ with } k'_{2b} = k_{2b}[\boldsymbol{2b}]$$

Integrated form for pseudo-first order kinetics is:

$$[\mathbf{1a}](t) = [\mathbf{1a}]_0 \cdot e^{-k'_{2a} \cdot t} \text{ (for the reaction with stock solution } \mathbf{I}\text{)}$$

$$[\mathbf{1a}](t) = [\mathbf{1a}]_0 \cdot e^{-k'_{2b} \cdot t} \text{ (for the reaction with base solution } \mathbf{II}\text{)}$$

Taking the natural log on both sites:

$$\ln([\mathbf{1a}](t)) = \ln([\mathbf{1a}]_0) - k'_{2a} \cdot t \text{ (for the reaction with base solution } \mathbf{I}\text{)}$$

$$\ln([\mathbf{1l}](t)) = \ln([\mathbf{1l}]_0) - k'_{2b} \cdot t \text{ (for the reaction with base solution } \mathbf{II}\text{)}$$

Plotting the ln([ArCl](t)) vs. t (min) for early to mid-stage data points should give a straight line with slope = $-$ $k'_{\text{bis-lactim ether}}$.

t (min)	0	5	10	20	40	60	120
ln(c(**1a**)) from the reaction with **2a**	-2,302585	-2,967922	-3,454281	-4,287443	-6,907755	-6,907755	-6,907755
ln(c(**1l**)) from the reaction with **2b**	-2,302585	-3,652740	-4,336670	-5,521460	-6,907755	-6,907755	-6,907755

For the linear fit only the data points within the first 20 minutes were considered:

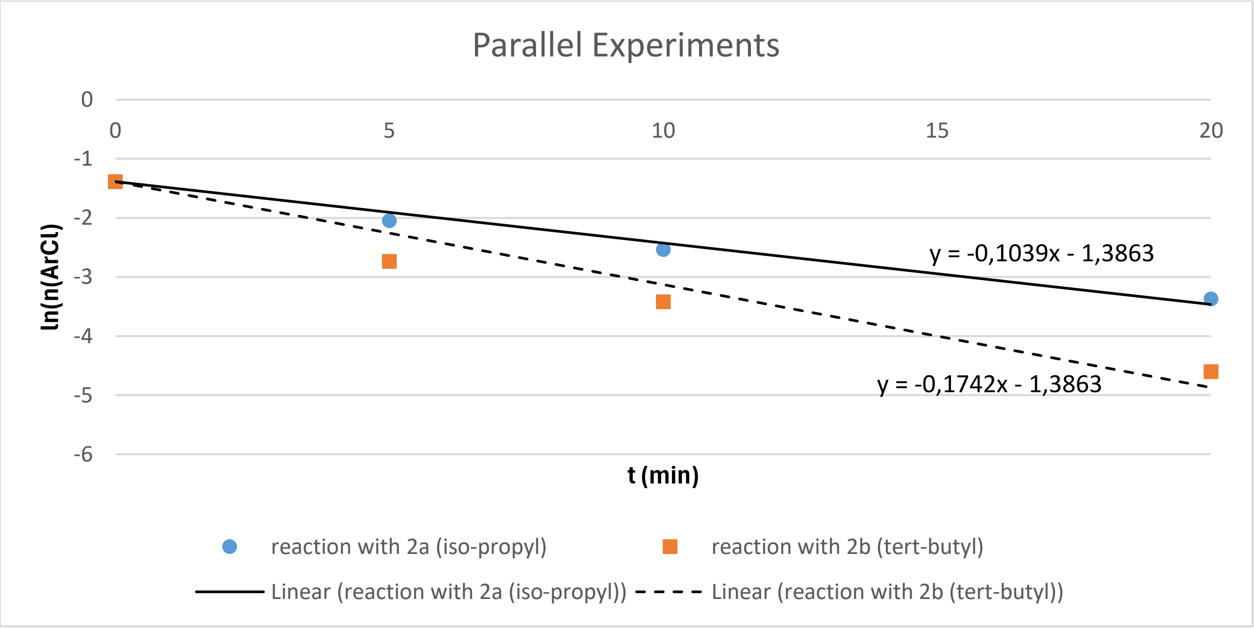

The parallel reaction of aryl chloride **1a** with **2a** results in k'_{2a} = 0.1039 min^{-1}, while the parallel experiment of **1a** with **2b** yields k'_{2b} = 0.1742 min^{-1}.

The ratio of both reaction rates is defined as follows:

$$\frac{k_{2a}}{k_{2b}} = \frac{k'_{2a}\,[\mathbf{2a}]_0}{k'_{2b}\,[\mathbf{2b}]_0} = \frac{k'_{2a}}{k'_{2b}} \approx 0.596$$

or

$$\frac{k_{2b}}{k_{2a}} \approx 1.7$$

In the parallel experiments the reaction rates in the reaction of aryl chloride **1a** with bis-lactim ether **2b** and aryl chloride **1a** and bis-lactim ether **2a** show a ratio of approximately k_{2a}:k_{2b} = 1.7:1.

10. Stereochemistry for Compound 3ea

Comparing the NMR data – both chemical shifts and coupling constants – with those of other isolated compounds and compounds of literature reveals the following: While trans-diastereomers typically display a doublet at 5.00–5.26 ppm with a coupling constant of J = 3.6–3.8 Hz, compound **3ea** shows a more downfield-shifted doublet at 5.40 ppm and a significantly larger J = 7.7 Hz. Literature reports on cis-configured bis-lactim ethers also note a downfield-shift and a higher coupling constant compared to their trans counterparts.[5] This points towards a cis-configuration for **3ea**. However, a perpendicular aryl orientation could also explain the observed shift.

To further evaluate this assignment, we conducted NOE experiments. A cis-isomer would be expected to exhibit NOE correlation between the aryl and isopropyl groups. However, no such correlation was observed in **3ea**. Notably, the NOESY spectrum shows a strong NOE between the doublet at 5.40 ppm and the doublet of doublets at 3.91 ppm.

Phase-sensitive NOESY of **3ea**:

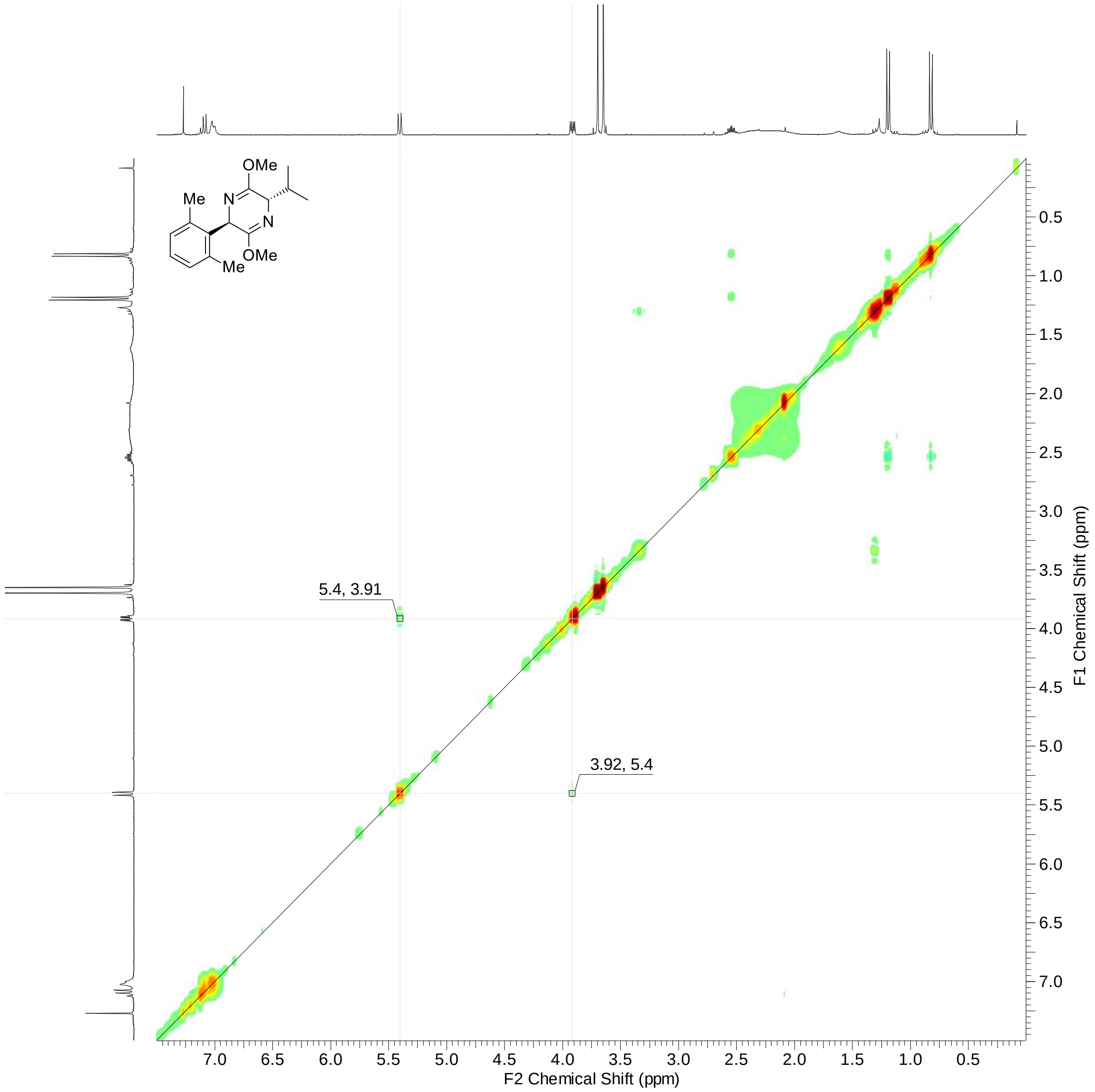

As a comparison, we measured the NOESY spectrum of model compound **3aa**, whose trans-configuration was confirmed by X-ray diffraction. Like **3ea**, **3aa** does not show a NOE correlation between the aryl and isopropyl groups of the bis-lactim ether. Instead, it exhibits a NOE between the methine protons on both sides of the bis-lactim ether, specifically between the signals at 4.96 ppm and 4.01 ppm (similar NOE correlations as for compound **3ea**).

Phase-sensitive NOESY of **3aa** (model substrate):

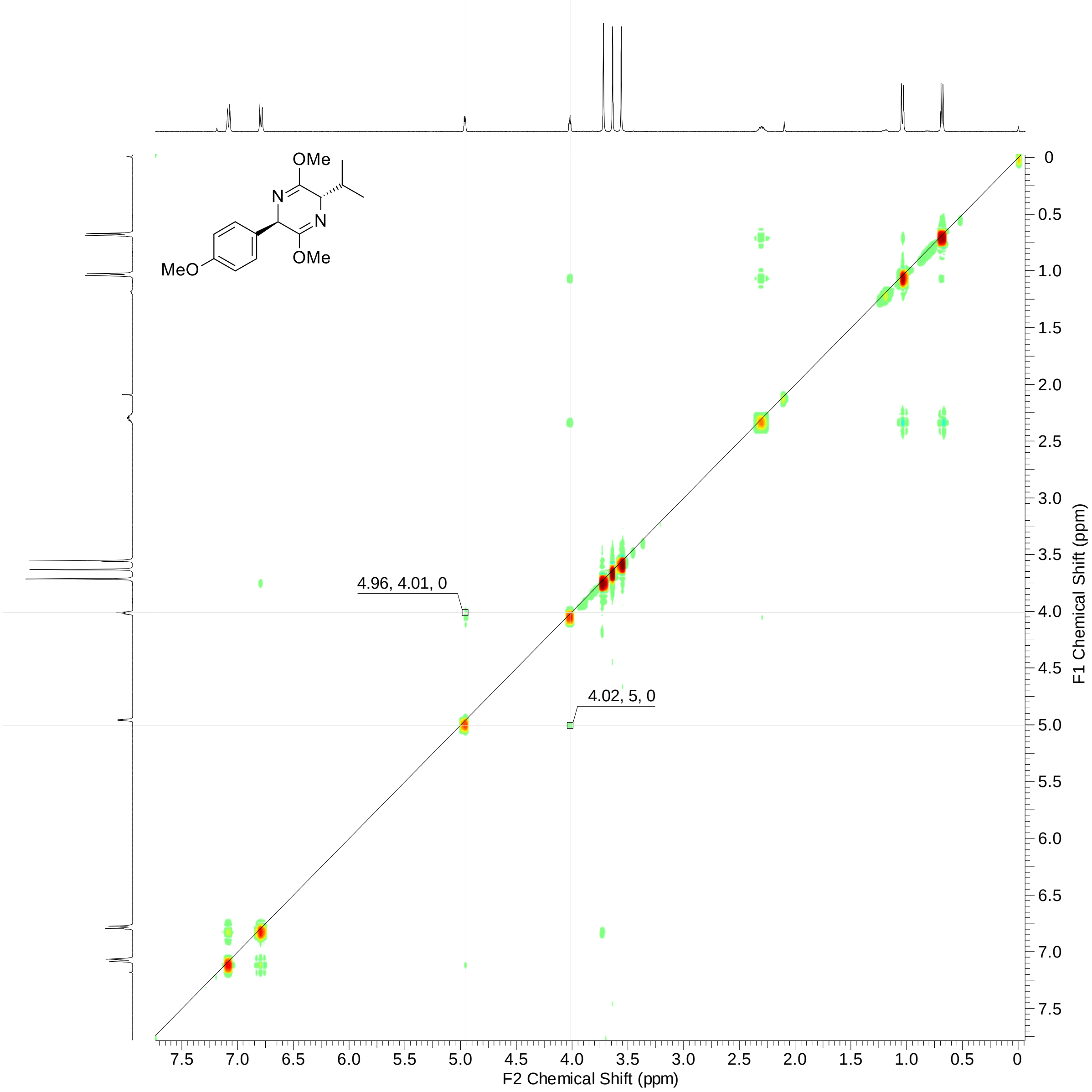

The similarity in NOE patterns between **3ea** and the confirmed trans-isomer **3aa** suggests that **3ea** may also adopt a trans-configuration. However, this evidence alone is not conclusive enough to definitively assign the configuration.

To further probe the stereochemistry of **3ea**, we attempted its synthesis using XPhos as the ligand, expecting this to result in a low diastereomeric ratio. Analysis of the crude reaction mixture by NMR would then allow us to differentiate between possible isomers. Surprisingly, the reaction of the isopropyl-substituted bis-lactim ether with XPhos afforded the same diastereomer with a dr >25:1, precluding further stereochemical investigation by this method.

Overall, neither the coupling constants in 1D-NMR nor 2D-NMR investigations allowed us to pinpoint the absolute stereochemistry of the dominating stereoisomer for compound **3ea**. We have decided to draw it as the expected *trans*-isomer, but a *cis*-configuration cannot be excluded.

11. NMR-Spectra of novel NYPhos Ligands

<u>^{Pip}trYPhos</u>

^{1}H NMR (400 MHz, C_6D_6)

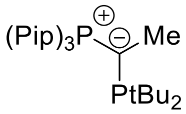

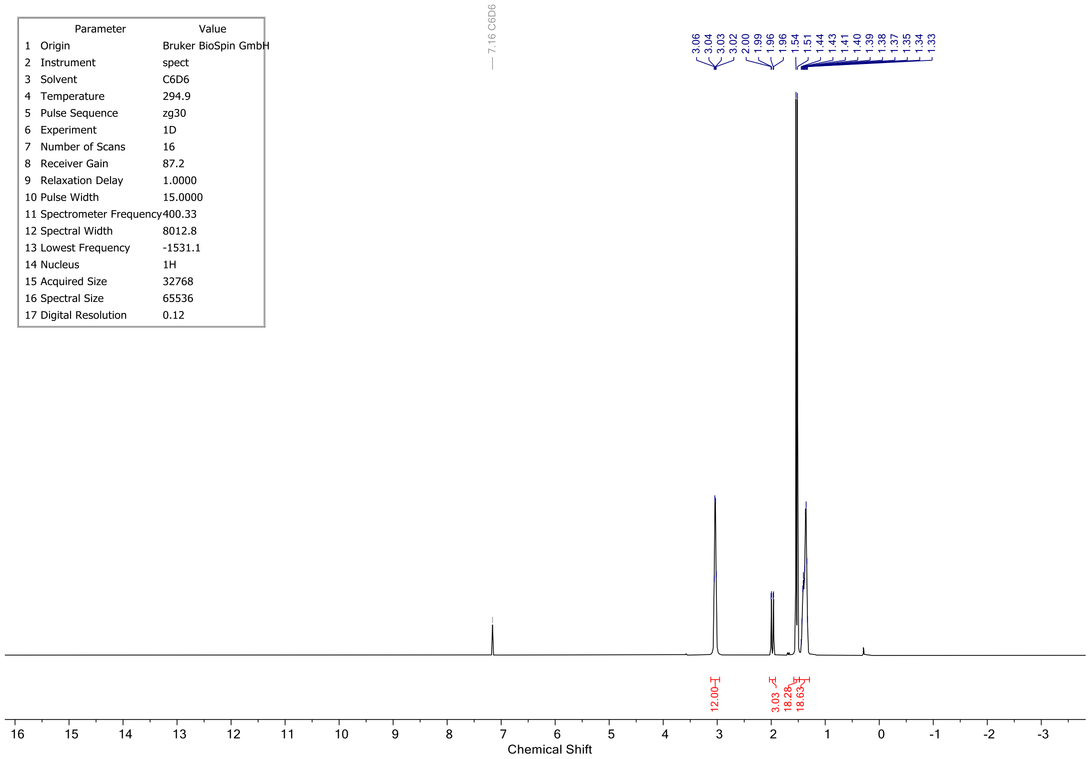

	Parameter	Value
1	Origin	Bruker BioSpin GmbH
2	Instrument	spect
3	Solvent	C6D6
4	Temperature	294.9
5	Pulse Sequence	zg30
6	Experiment	1D
7	Number of Scans	16
8	Receiver Gain	87.2
9	Relaxation Delay	1.0000
10	Pulse Width	15.0000
11	Spectrometer Frequency	400.33
12	Spectral Width	8012.8
13	Lowest Frequency	-1531.1
14	Nucleus	1H
15	Acquired Size	32768
16	Spectral Size	65536
17	Digital Resolution	0.12

^{13}C NMR (101 MHz, C_6D_6)

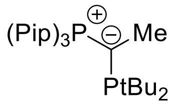

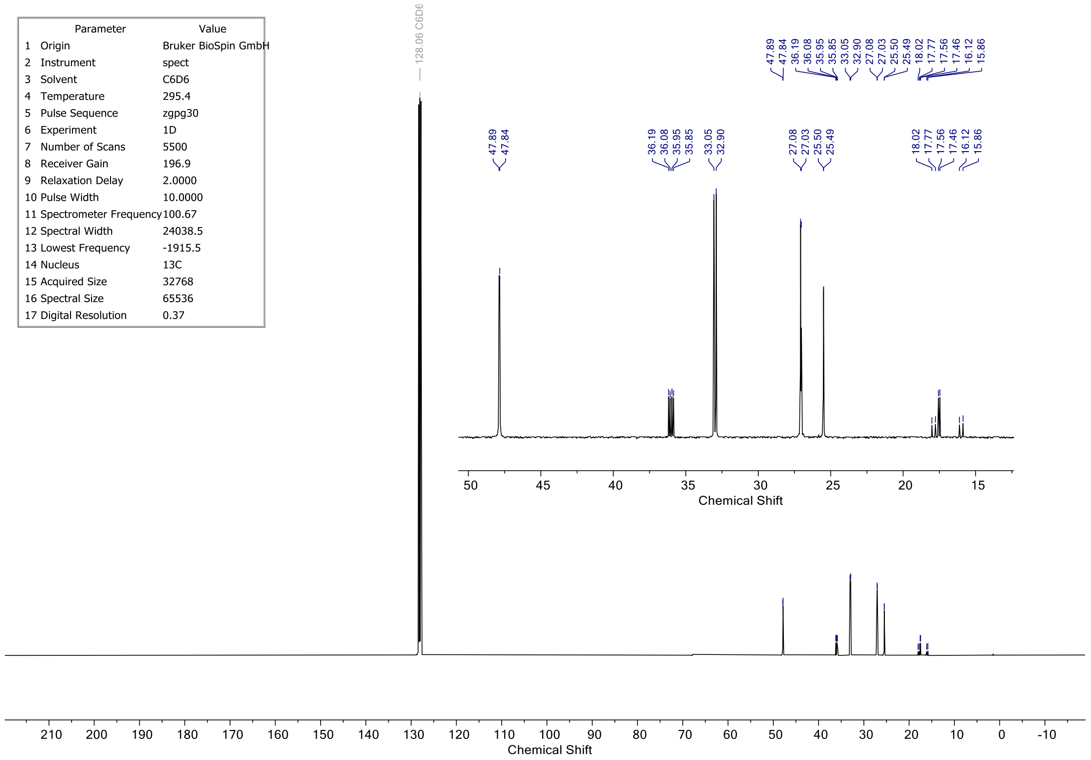

	Parameter	Value
1	Origin	Bruker BioSpin GmbH
2	Instrument	spect
3	Solvent	C6D6
4	Temperature	295.4
5	Pulse Sequence	zgpg30
6	Experiment	1D
7	Number of Scans	5500
8	Receiver Gain	196.9
9	Relaxation Delay	2.0000
10	Pulse Width	10.0000
11	Spectrometer Frequency	100.67
12	Spectral Width	24038.5
13	Lowest Frequency	-1915.5
14	Nucleus	13C
15	Acquired Size	32768
16	Spectral Size	65536
17	Digital Resolution	0.37

^{31}P NMR (162 MHz, C_6D_6)

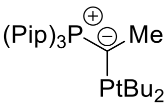

	Parameter	Value
1	Origin	Bruker BioSpin GmbH
2	Instrument	spect
3	Solvent	C6D6
4	Temperature	295.3
5	Pulse Sequence	zgpg30
6	Experiment	1D
7	Number of Scans	16
8	Receiver Gain	196.9
9	Relaxation Delay	2.0000
10	Pulse Width	15.0000
11	Spectrometer Frequency	162.06
12	Spectral Width	64102.6
13	Lowest Frequency	-23948.5
14	Nucleus	31P
15	Acquired Size	32768
16	Spectral Size	65536
17	Digital Resolution	0.98

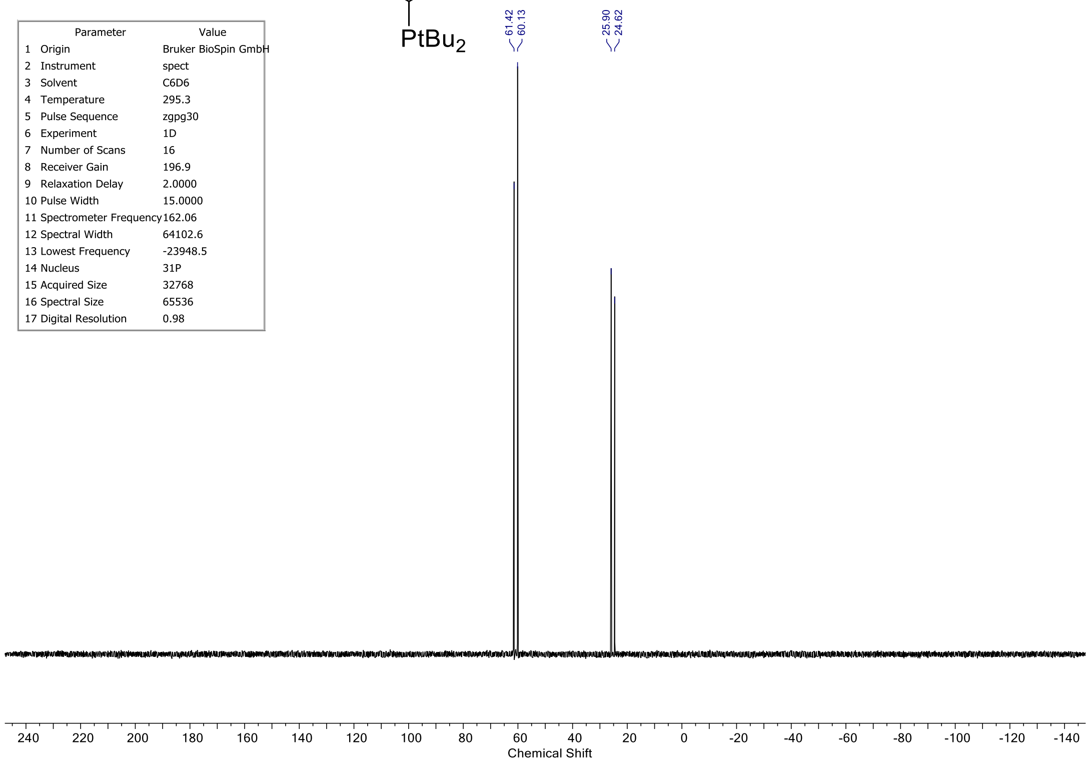

^{Pip}AdYPhos

^{1}H NMR (400 MHz, C_6D_6)

	Parameter	Value
1	Origin	Bruker BioSpin GmbH
2	Instrument	spect
3	Solvent	C6D6
4	Temperature	295.8
5	Pulse Sequence	zg30
6	Experiment	1D
7	Number of Scans	32
8	Receiver Gain	97.5
9	Relaxation Delay	1.0000
10	Pulse Width	15.0000
11	Spectrometer Frequency	400.33
12	Spectral Width	8012.8
13	Lowest Frequency	-1531.0
14	Nucleus	1H
15	Acquired Size	32768
16	Spectral Size	65536
17	Digital Resolution	0.12

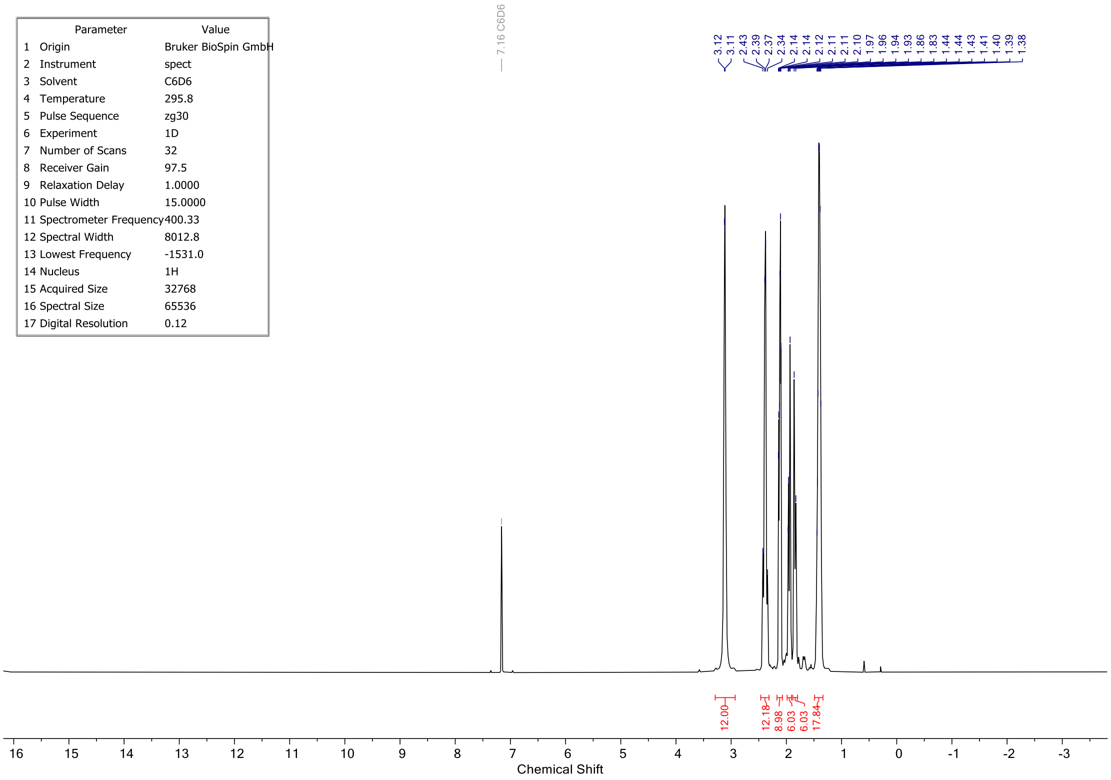

^{13}C NMR (101 MHz, C_6D_6)

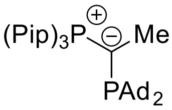

Parameter	Value
1 Origin	Bruker BioSpin GmbH
2 Instrument	spect
3 Solvent	C6D6
4 Temperature	296.1
5 Pulse Sequence	zgpg30
6 Experiment	1D
7 Number of Scans	6500
8 Receiver Gain	196.9
9 Relaxation Delay	2.0000
10 Pulse Width	10.0000
11 Spectrometer Frequency	100.67
12 Spectral Width	24038.5
13 Lowest Frequency	-1915.3
14 Nucleus	13C
15 Acquired Size	32768
16 Spectral Size	65536
17 Digital Resolution	0.37

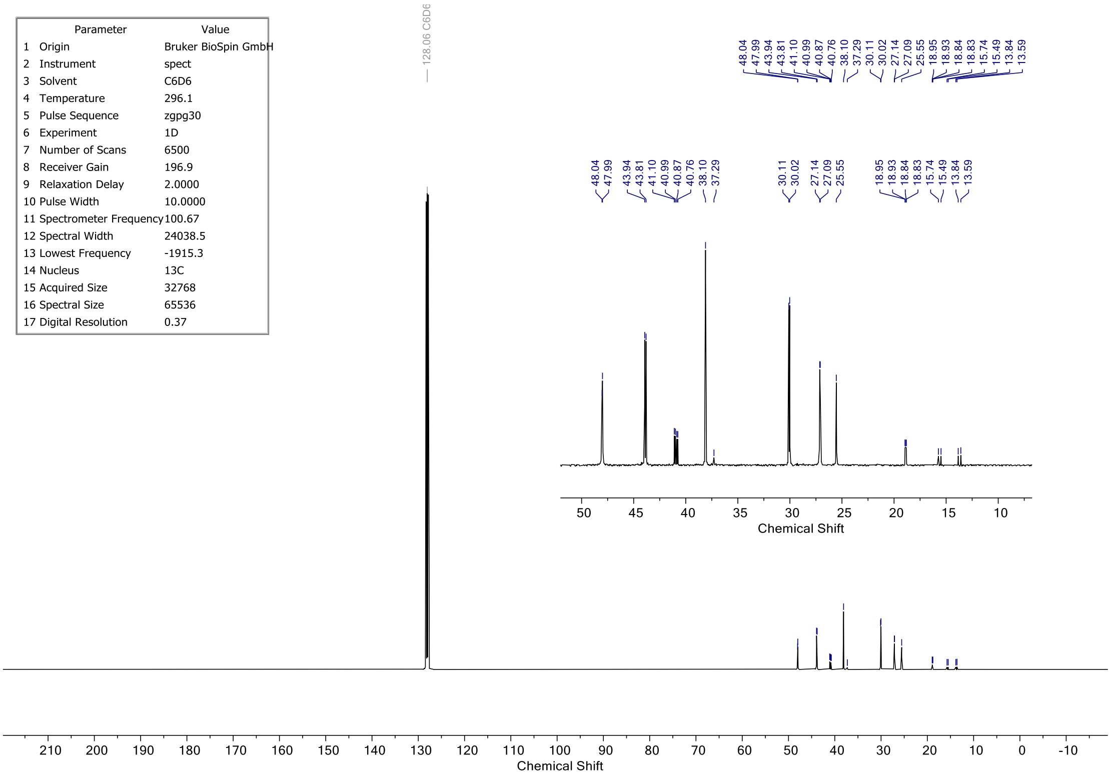

^{31}P NMR (162 MHz, C_6D_6)

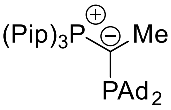

Parameter	Value
1 Origin	Bruker BioSpin GmbH
2 Instrument	spect
3 Solvent	C6D6
4 Temperature	296.3
5 Pulse Sequence	zgpg30
6 Experiment	1D
7 Number of Scans	32
8 Receiver Gain	196.9
9 Relaxation Delay	2.0000
10 Pulse Width	18.0000
11 Spectrometer Frequency	162.06
12 Spectral Width	64102.6
13 Lowest Frequency	-23948.5
14 Nucleus	31P
15 Acquired Size	32768
16 Spectral Size	65536
17 Digital Resolution	0.98

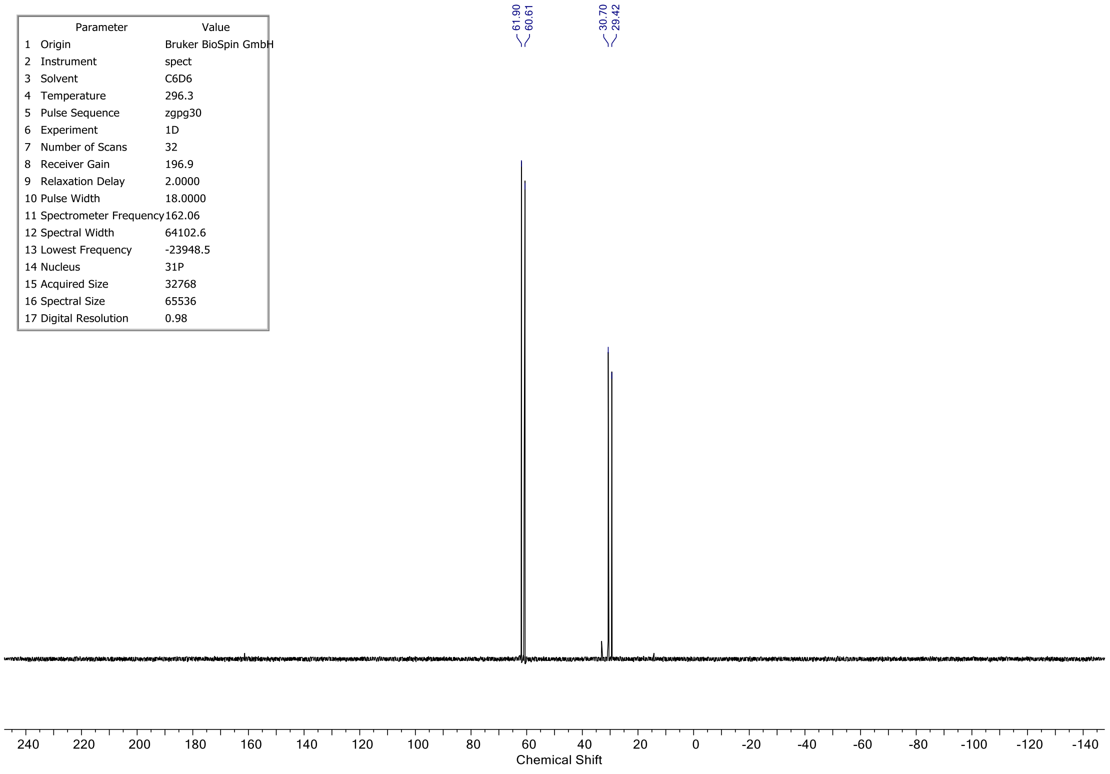

12. NMR-Spectra of Products

(2S,5R)-2-isopropyl-3,6-dimethoxy-5-(4-methoxyphenyl)-2,5-dihydropyrazine (**3aa**) [CAS: 109012-91-1]

^{1}H NMR (300 MHz, $CDCl_3$)

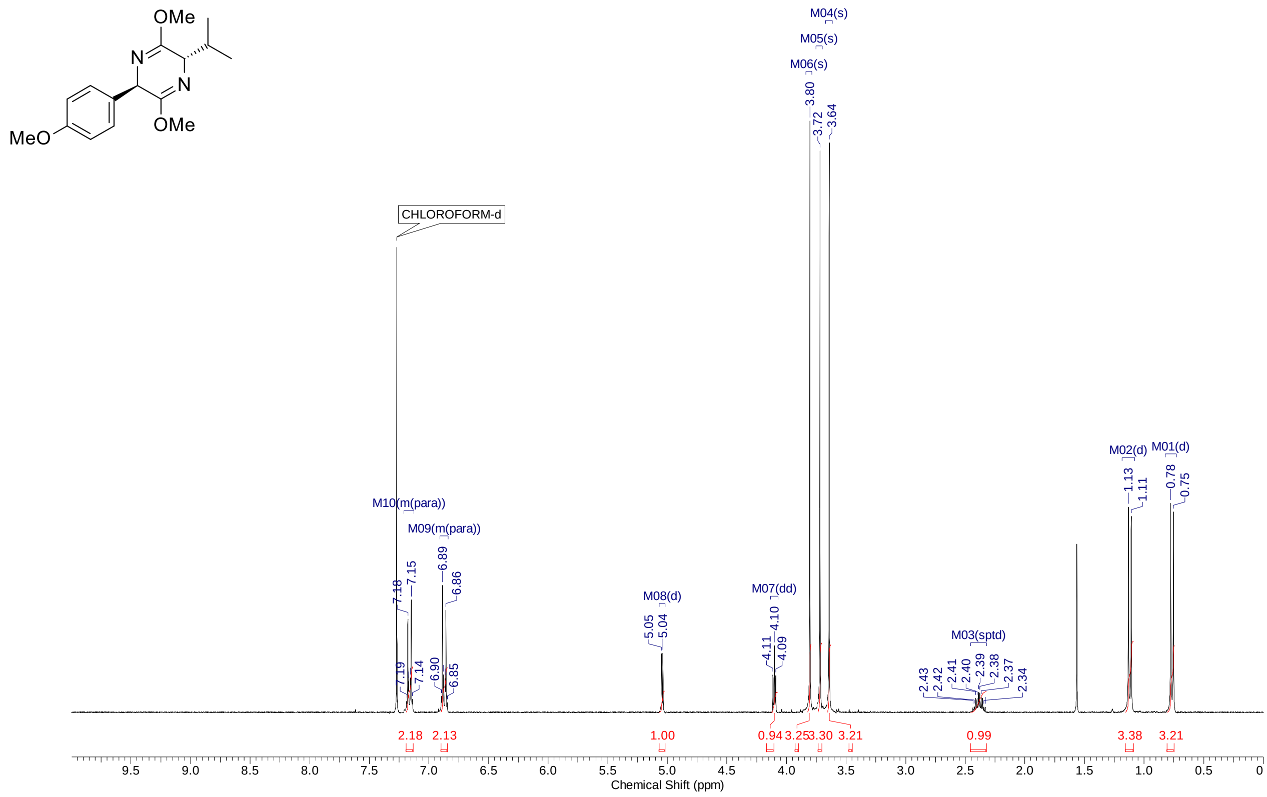

^{13}C NMR (75 MHz, $CDCl_3$)

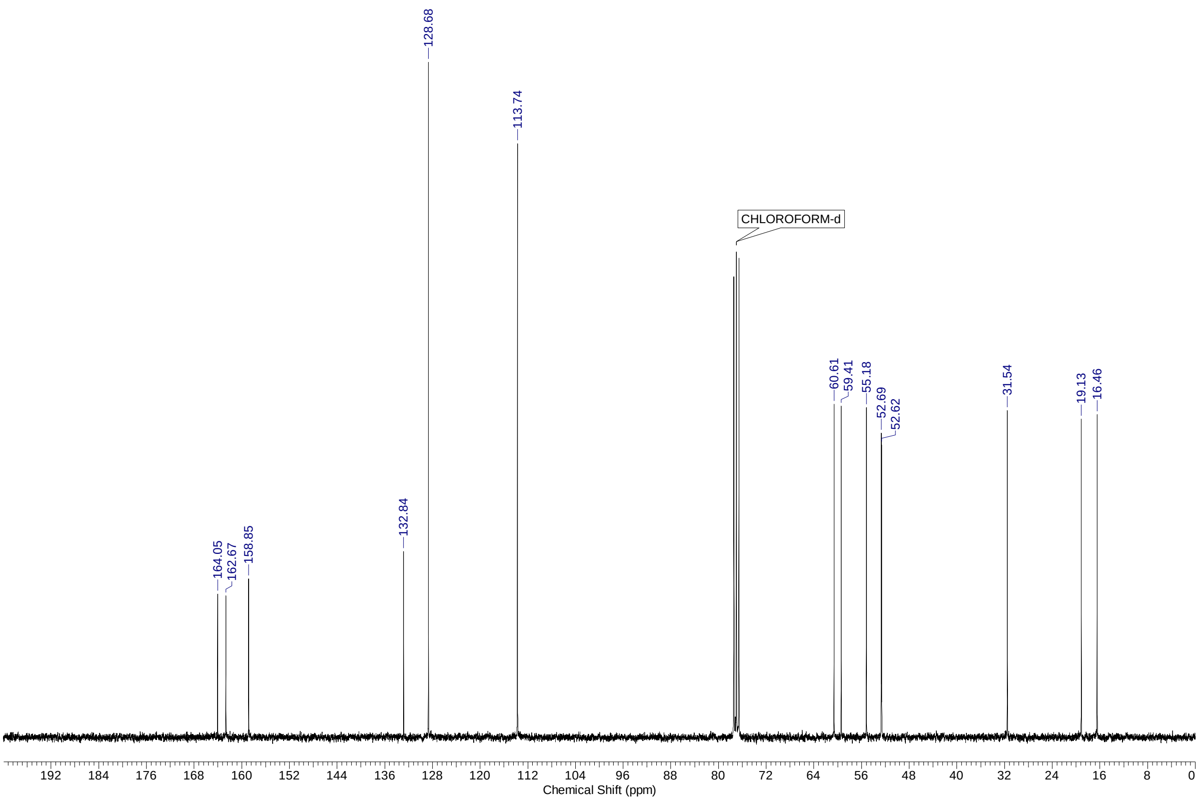

(2*S*,5*R*)-2-isopropyl-3,6-dimethoxy-5-(4-tolyl)-2,5-dihydropyrazine (**3ba**)

^{1}H NMR (300 MHz, $CDCl_3$)

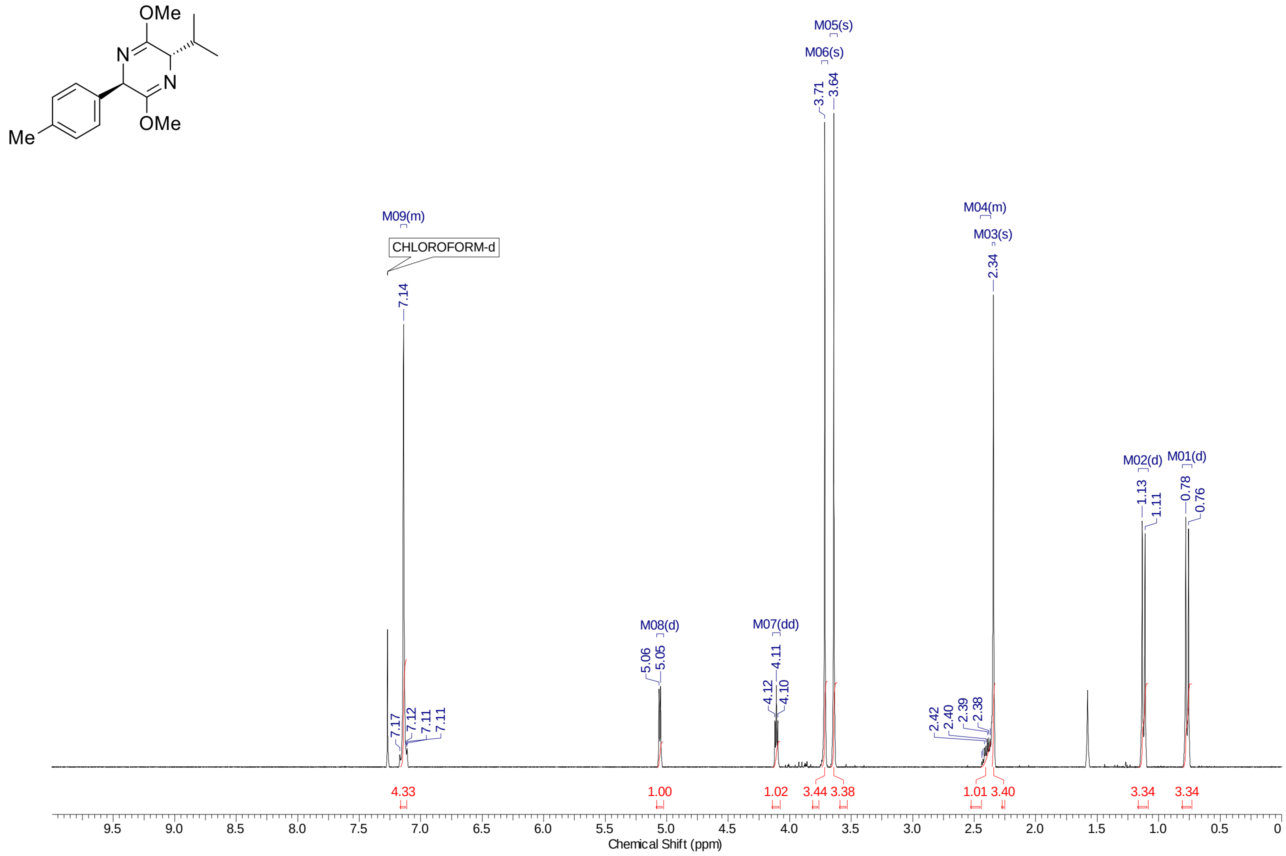

^{13}C NMR (75 MHz, $CDCl_3$)

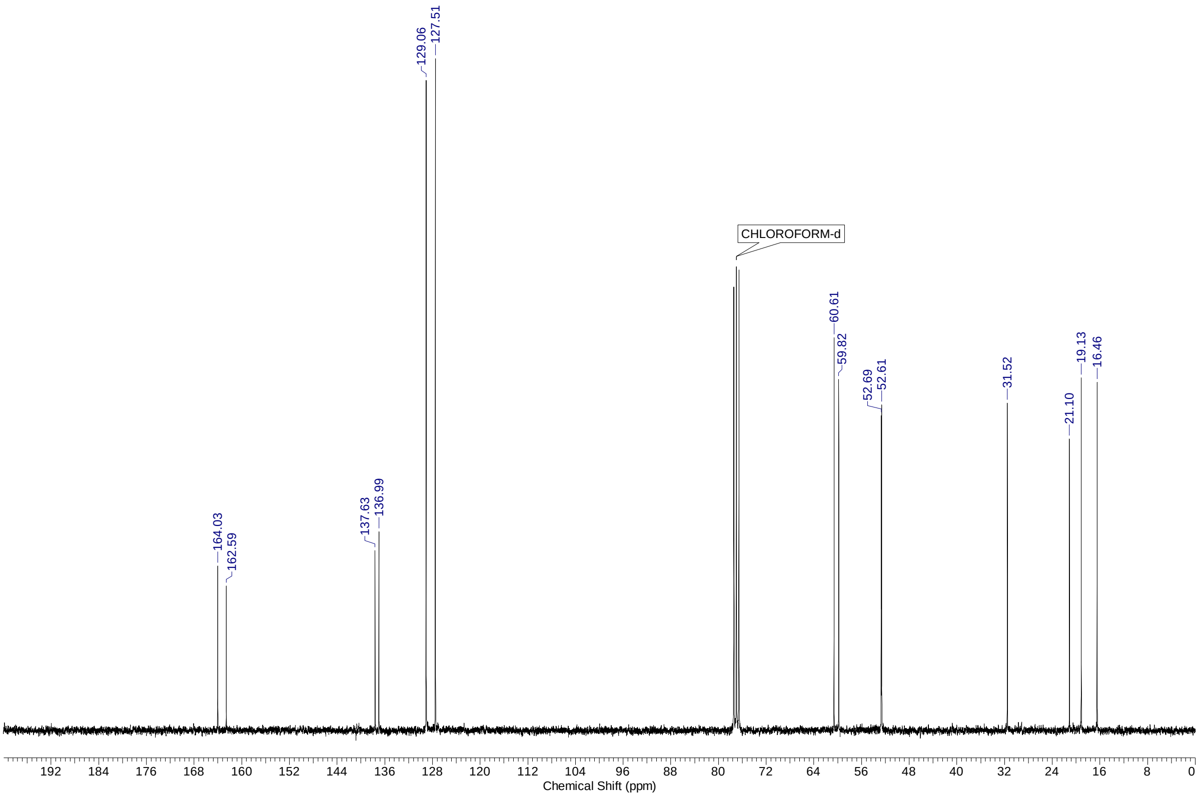

(2*S*,5*R*)-2-isopropyl-3,6-dimethoxy-5-(3-tolyl)-2,5-dihydropyrazine (**3ca**)

^{1}H NMR (300 MHz, $CDCl_3$)

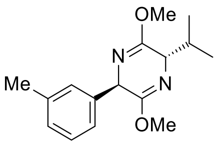

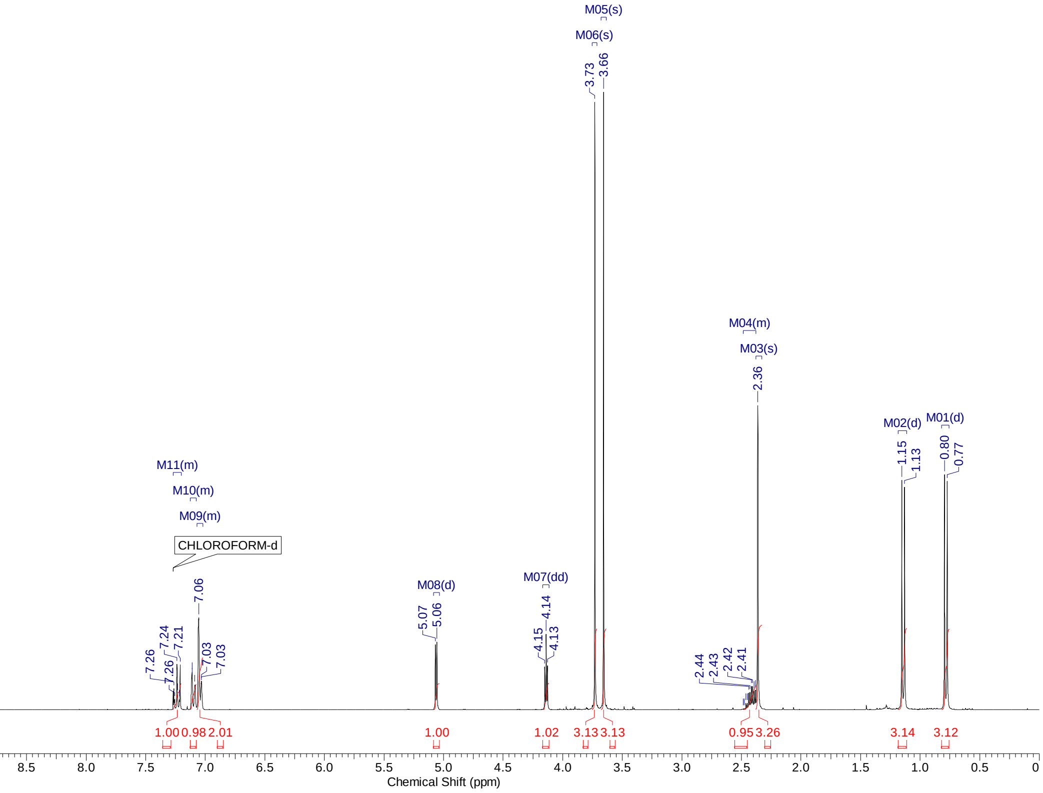

^{13}C NMR (75 MHz, $CDCl_3$)

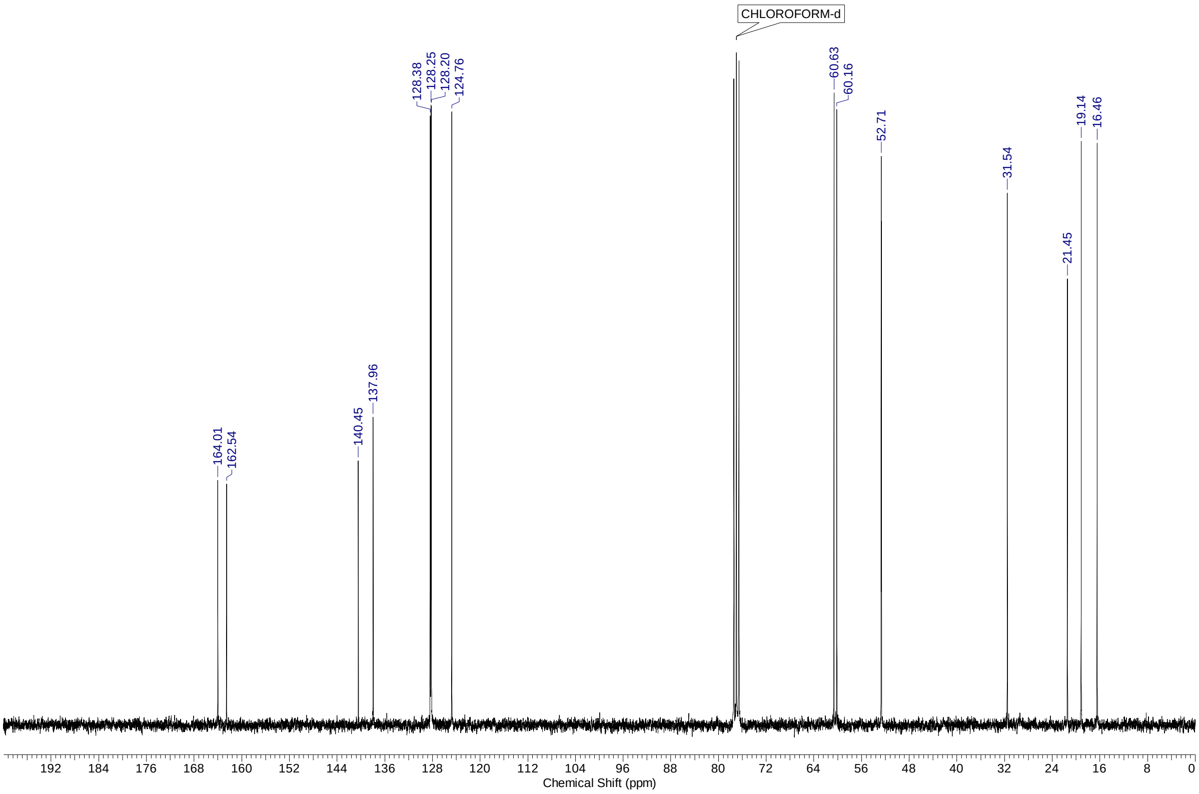

(2*S*,5*R*)-2-isopropyl-3,6-dimethoxy-5-(2-tolyl)-2,5-dihydropyrazine (**3da**)

^{1}H NMR (300 MHz, $CDCl_3$)

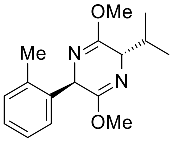

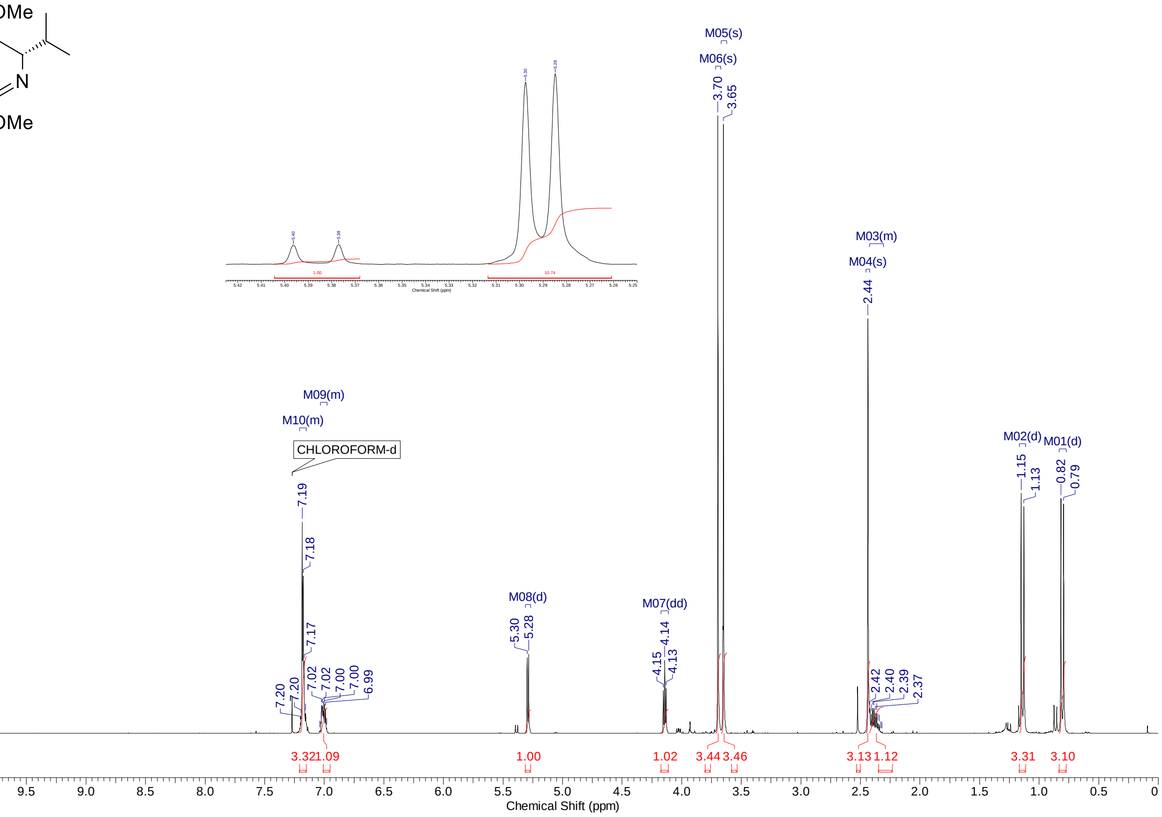

^{13}C NMR (75 MHz, $CDCl_3$)

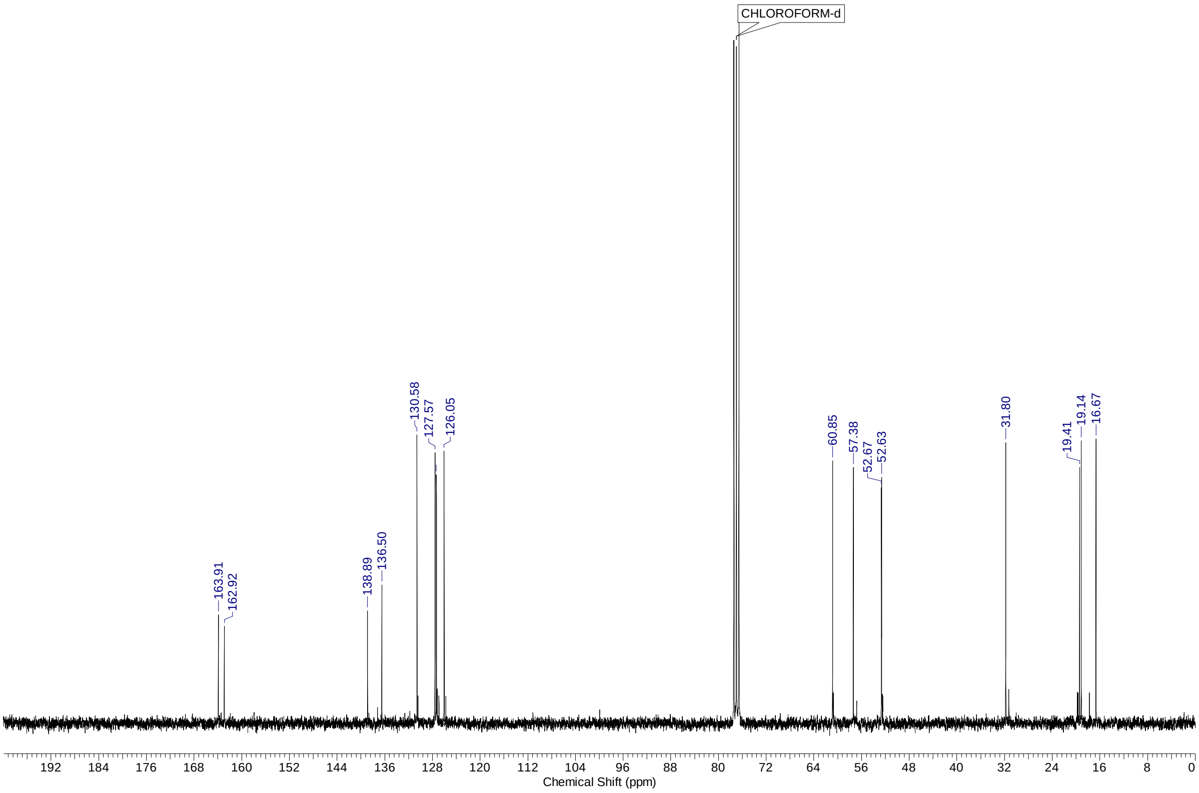

(2*S*,5*S*)-2-(2,6-dimethylphenyl)-5-isopropyl-3,6-dimethoxy-2,5-dihydropyrazine (**3ea**)

^{1}H NMR (300 MHz, $CDCl_3$)

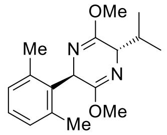

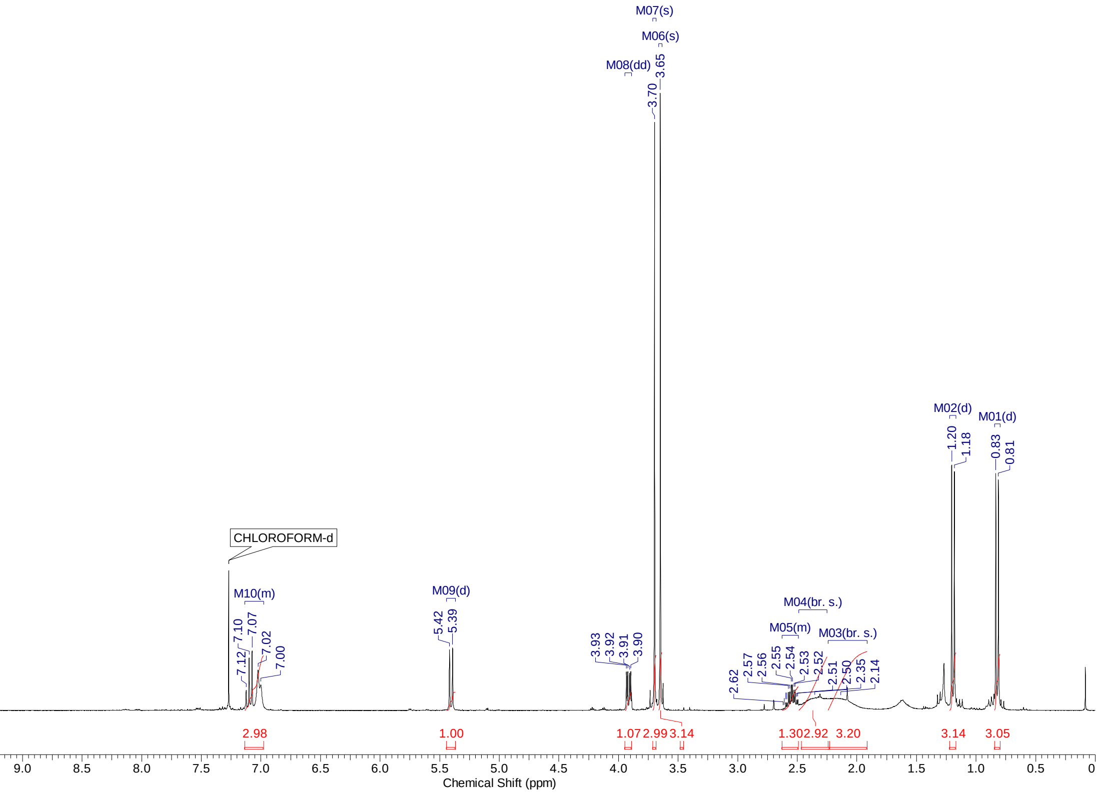

^{13}C NMR (75 MHz, $CDCl_3$)

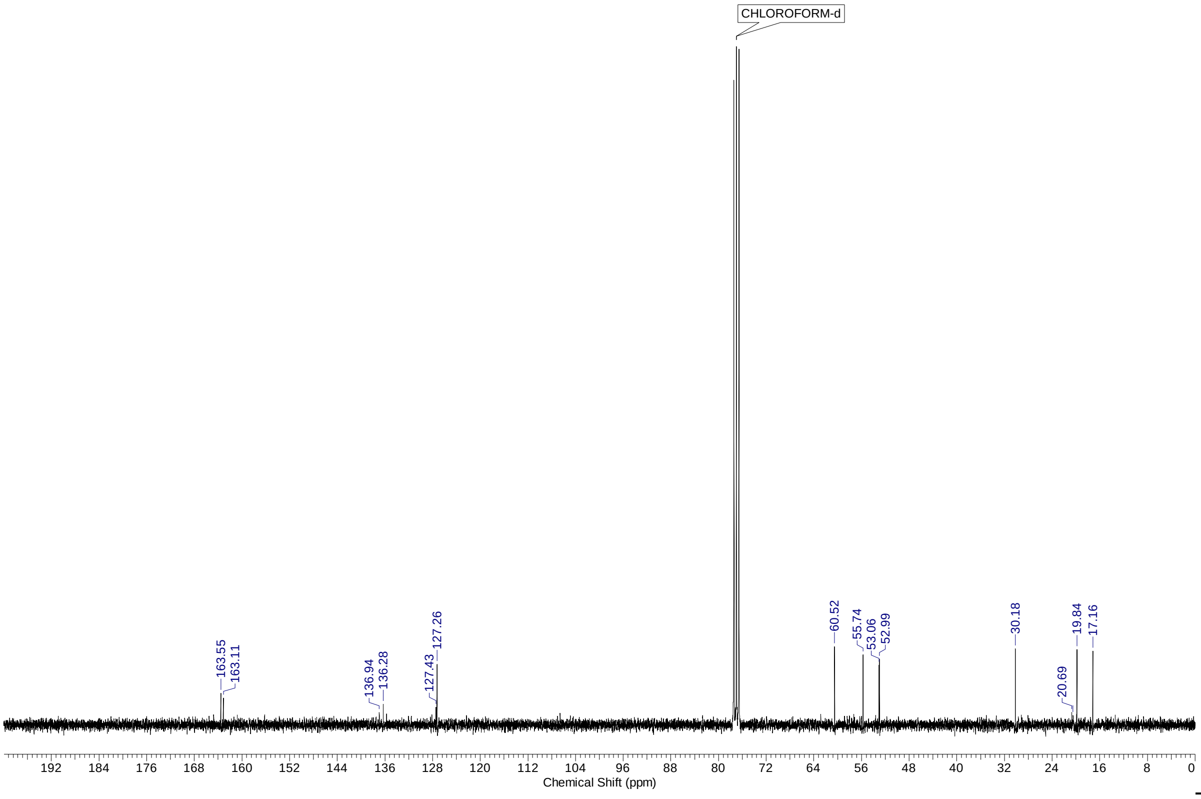

(2*S*,5*R*)-2-isopropyl-3,6-dimethoxy-5-phenyl-2,5-dihydropyrazine (**3fa**) [CAS: 126204-55-5]

^{1}H NMR (300 MHz, $CDCl_3$)

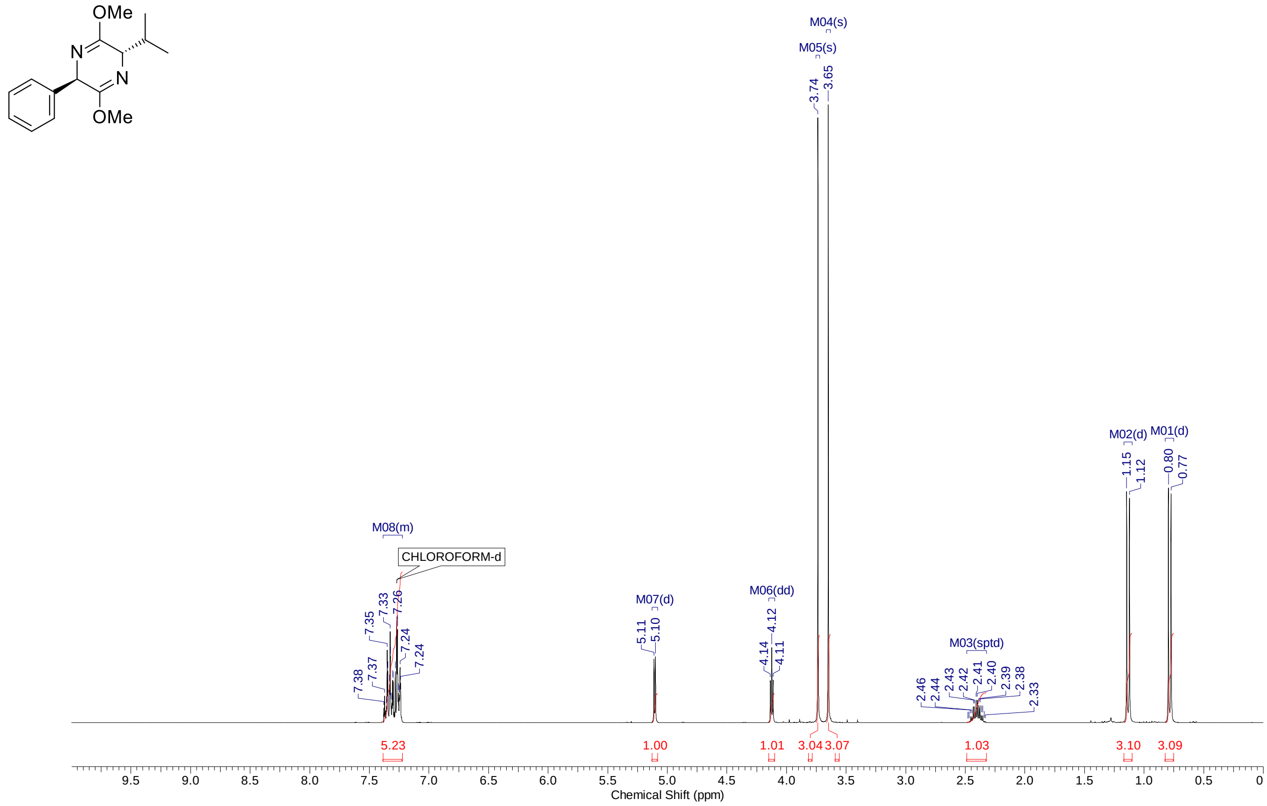

^{13}C NMR (75 MHz, $CDCl_3$)

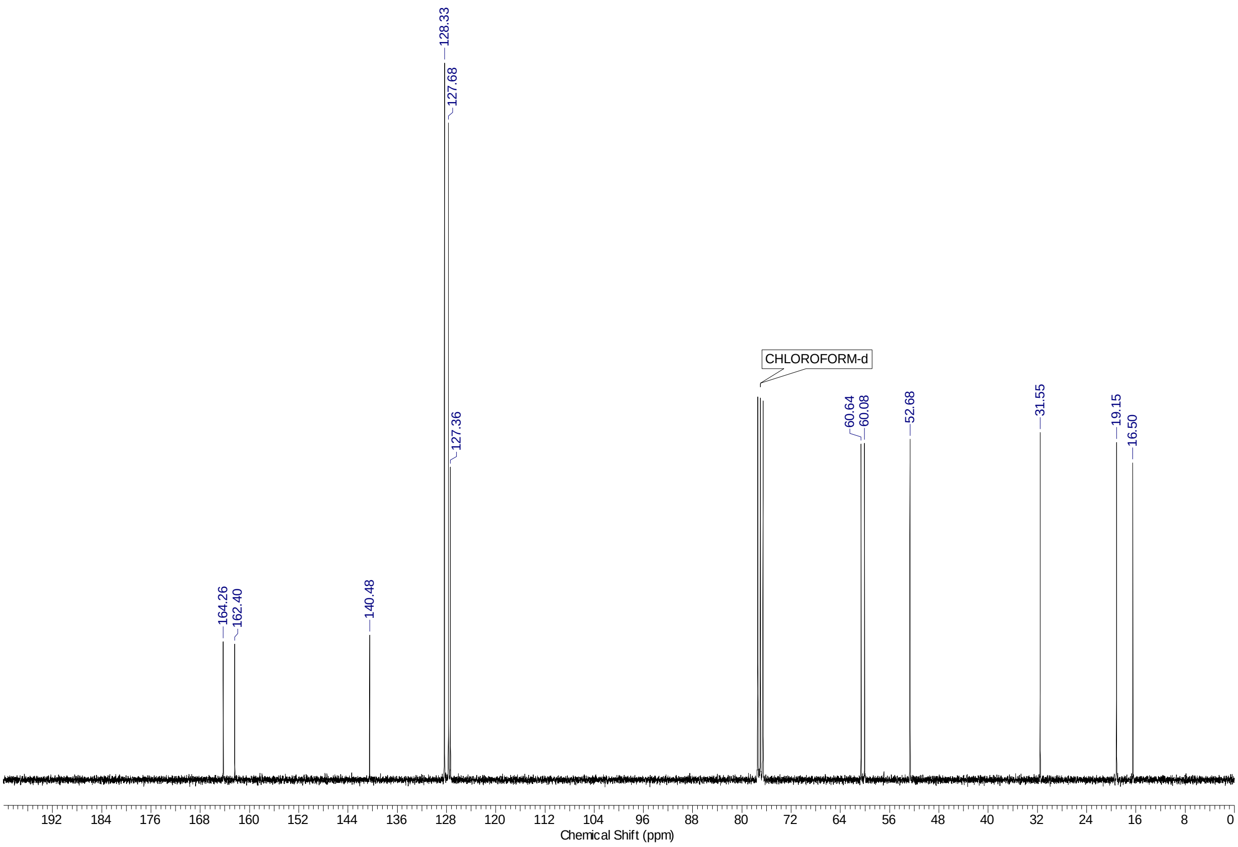

(2*R*,5*S*)-2-(4-fluorophenyl)-5-isopropyl-3,6-dimethoxy-2,5-dihydropyrazine (**3ga**)

^{1}H NMR (300 MHz, $CDCl_3$)

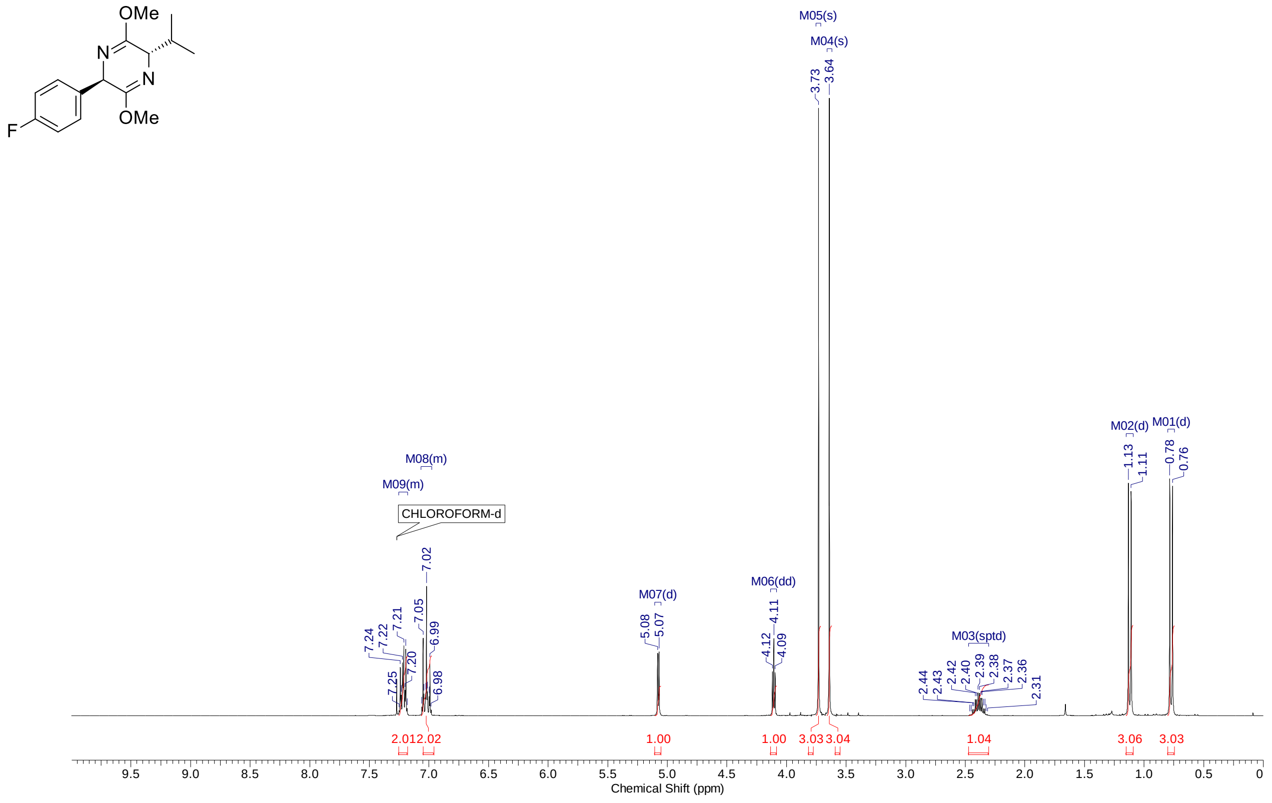

^{13}C NMR (75 MHz, $CDCl_3$)

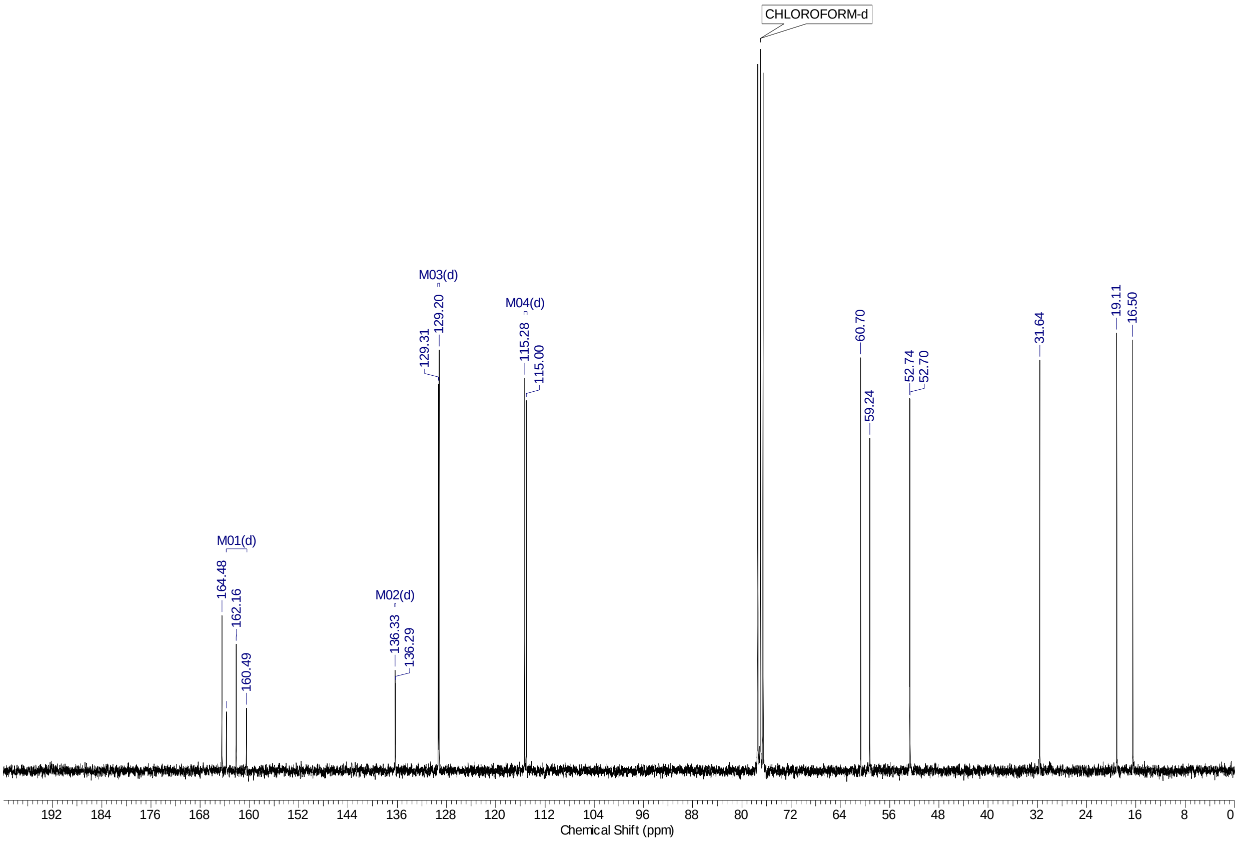

^{19}F NMR (41 MHz, $CDCl_3$)

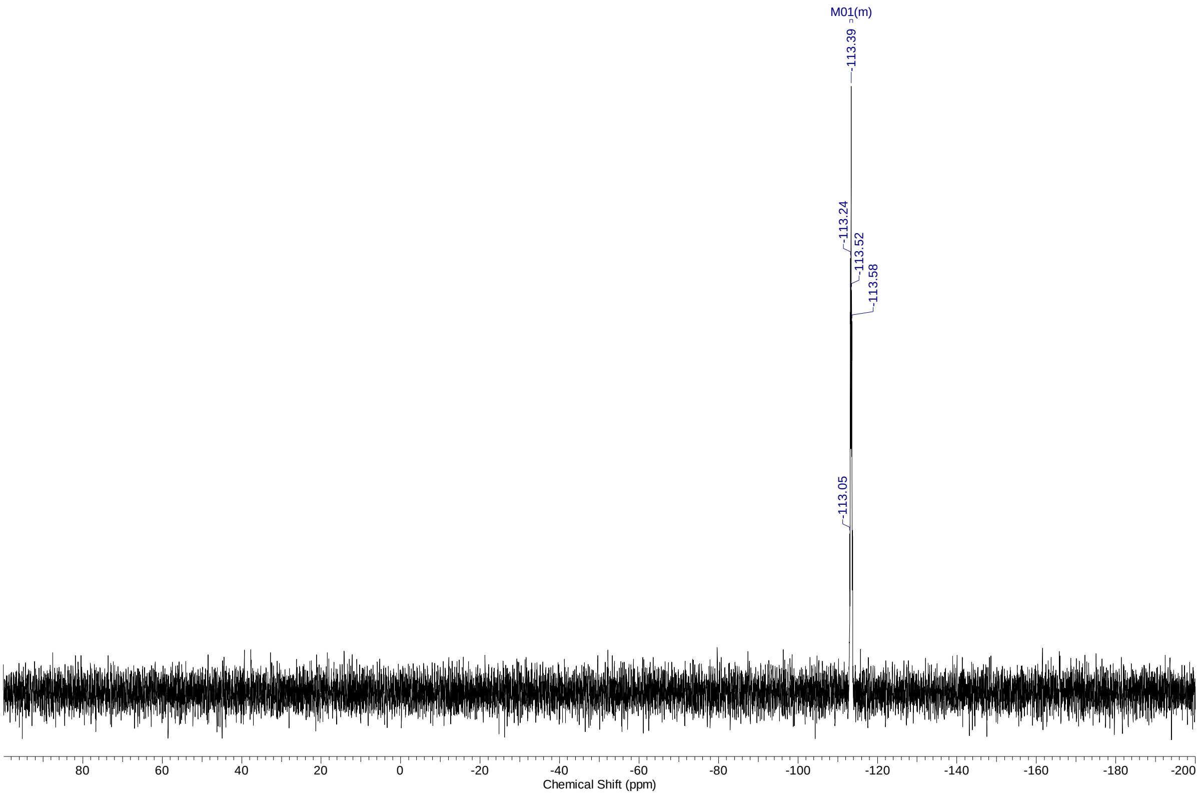

(2*S*,5*R*)-2-isopropyl-3,6-dimethoxy-5-[4-(trifluoromethoxy)phenyl]-2,5-dihydropyrazine (**3ha**)

^{1}H NMR (300 MHz, $CDCl_3$)

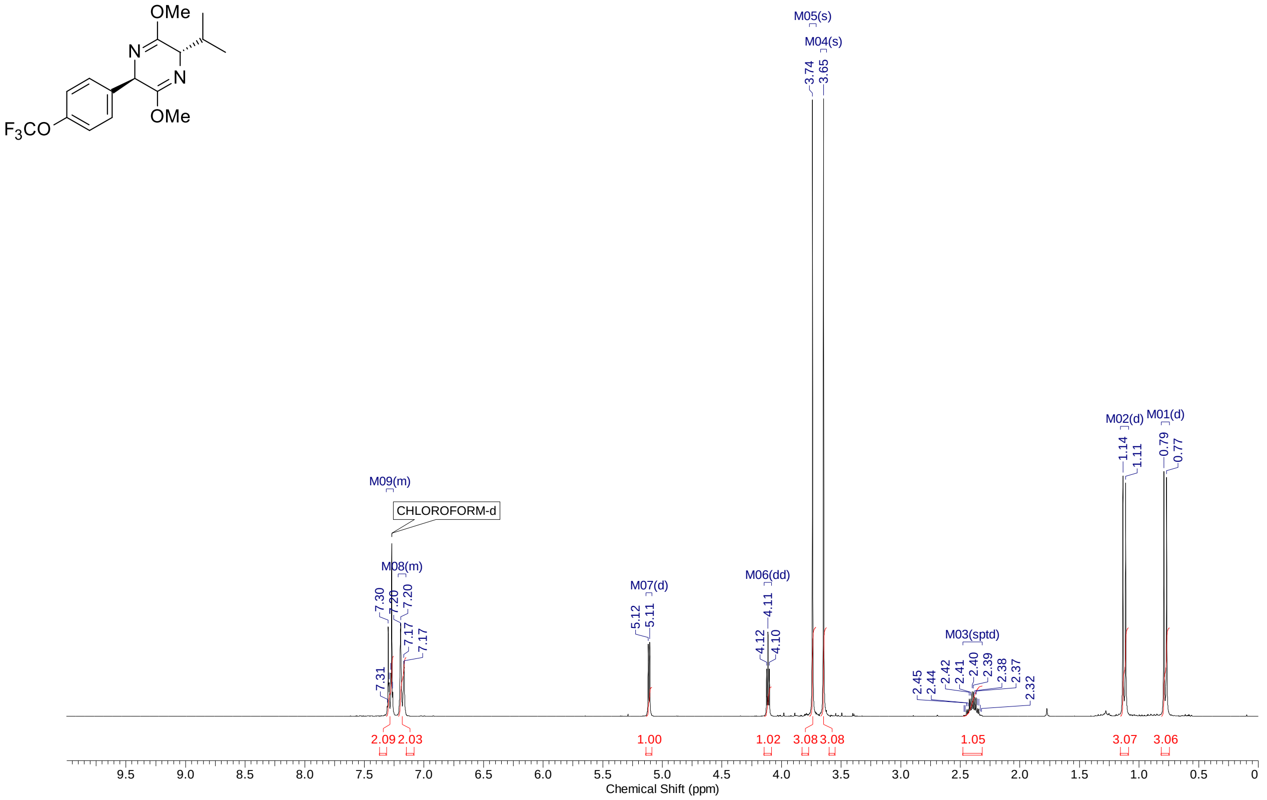

^{13}C NMR (75 MHz, $CDCl_3$)

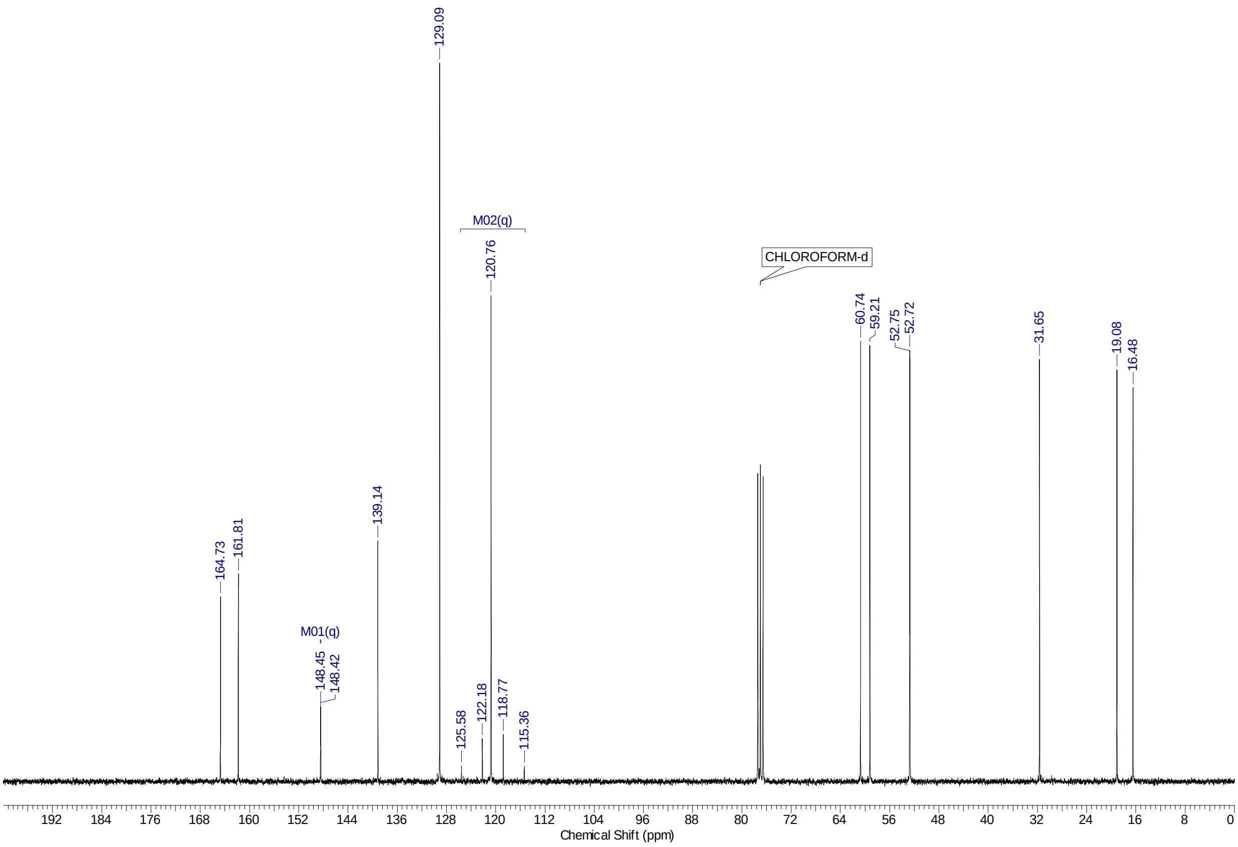

^{19}F NMR (41 MHz, $CDCl_3$)

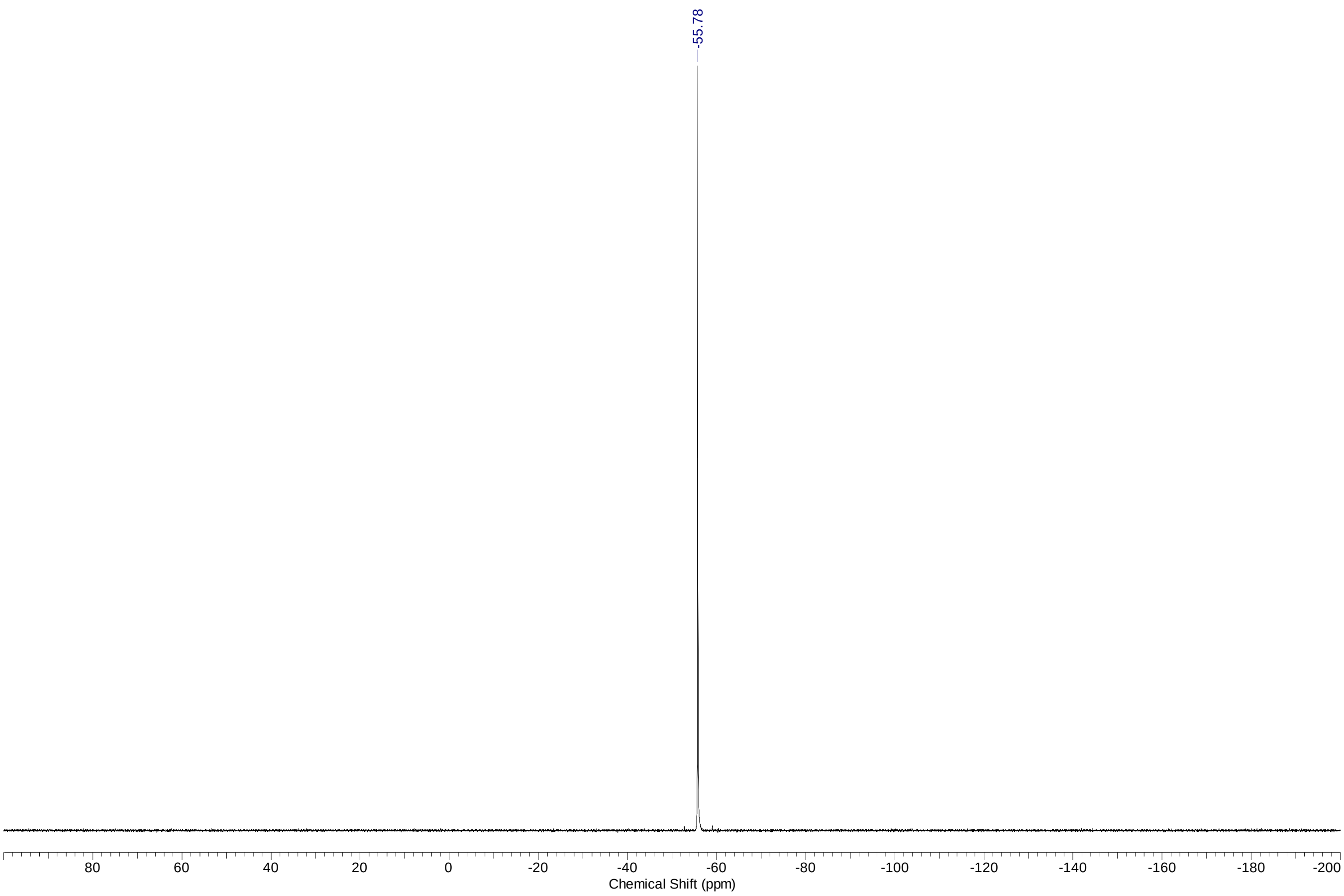

(2*S*,5*R*)-2-isopropyl-3,6-dimethoxy-5-(4-thioanisole)-2,5-dihydropyrazine (**3ia**)

^{1}H NMR (300 MHz, $CDCl_3$)

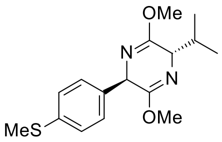

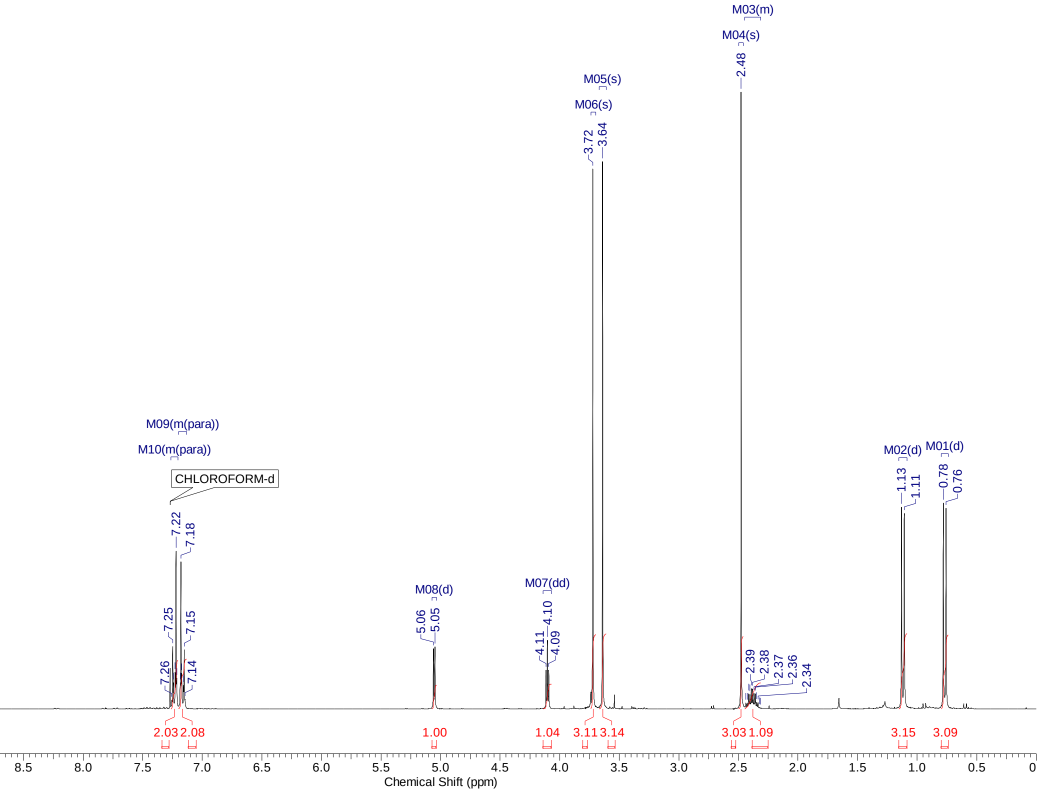

^{13}C NMR (75 MHz, $CDCl_3$)

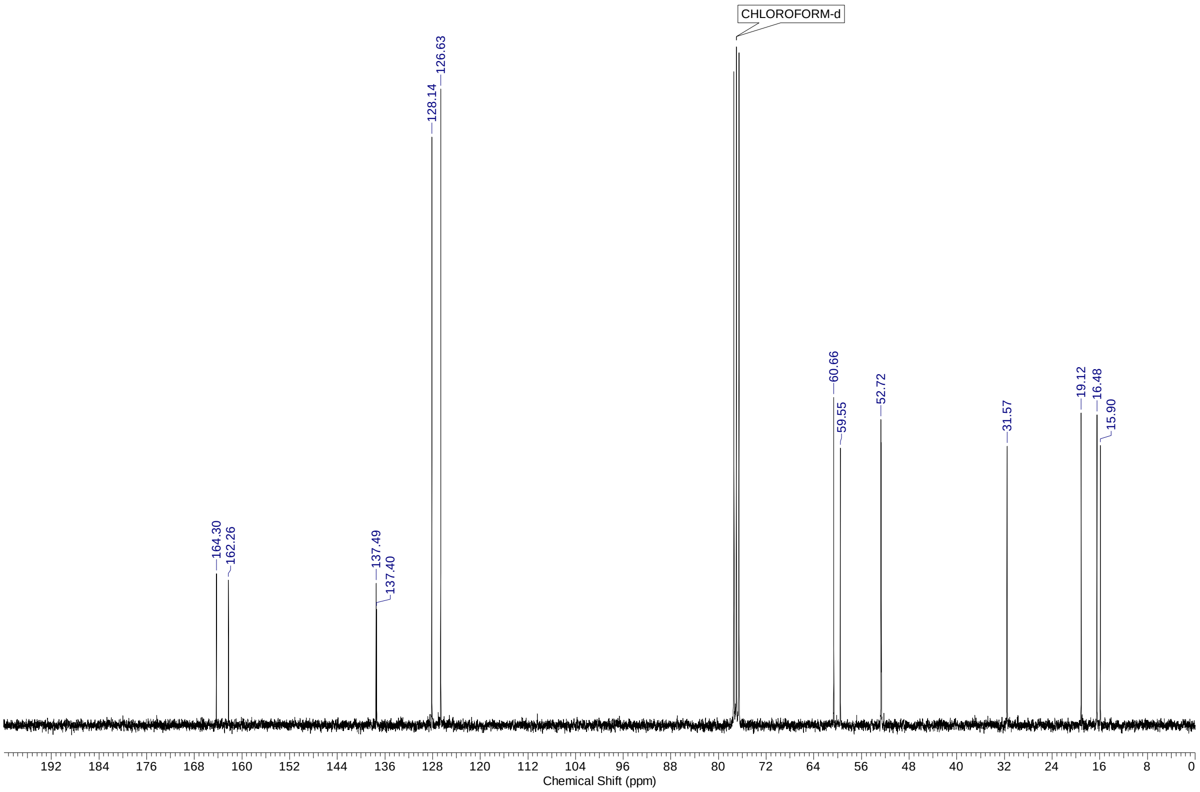

4-[(2*R*,5*S*)-5-isopropyl-3,6-dimethoxy-2,5-dihydropyrazin-2-yl]-*N*,*N*-dimethyl-aniline (**3ja**)

^1H NMR (300 MHz, $CDCl_3$)

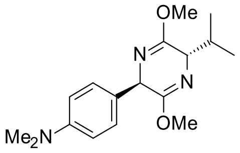

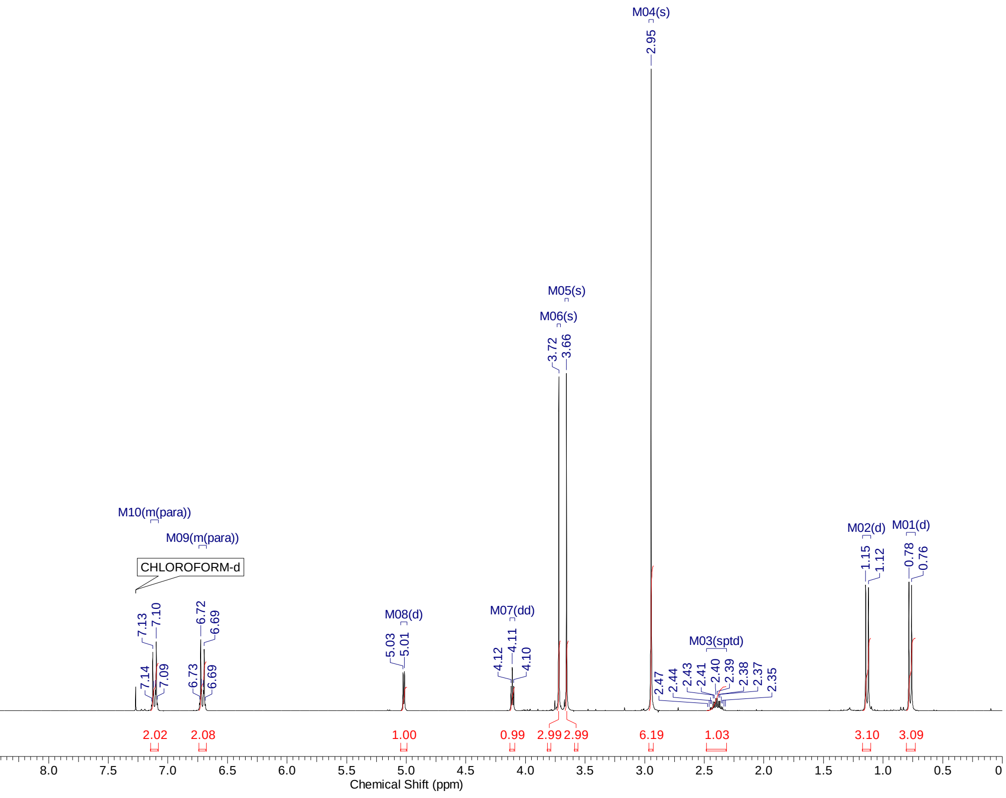

^{13}C NMR (75 MHz, $CDCl_3$)

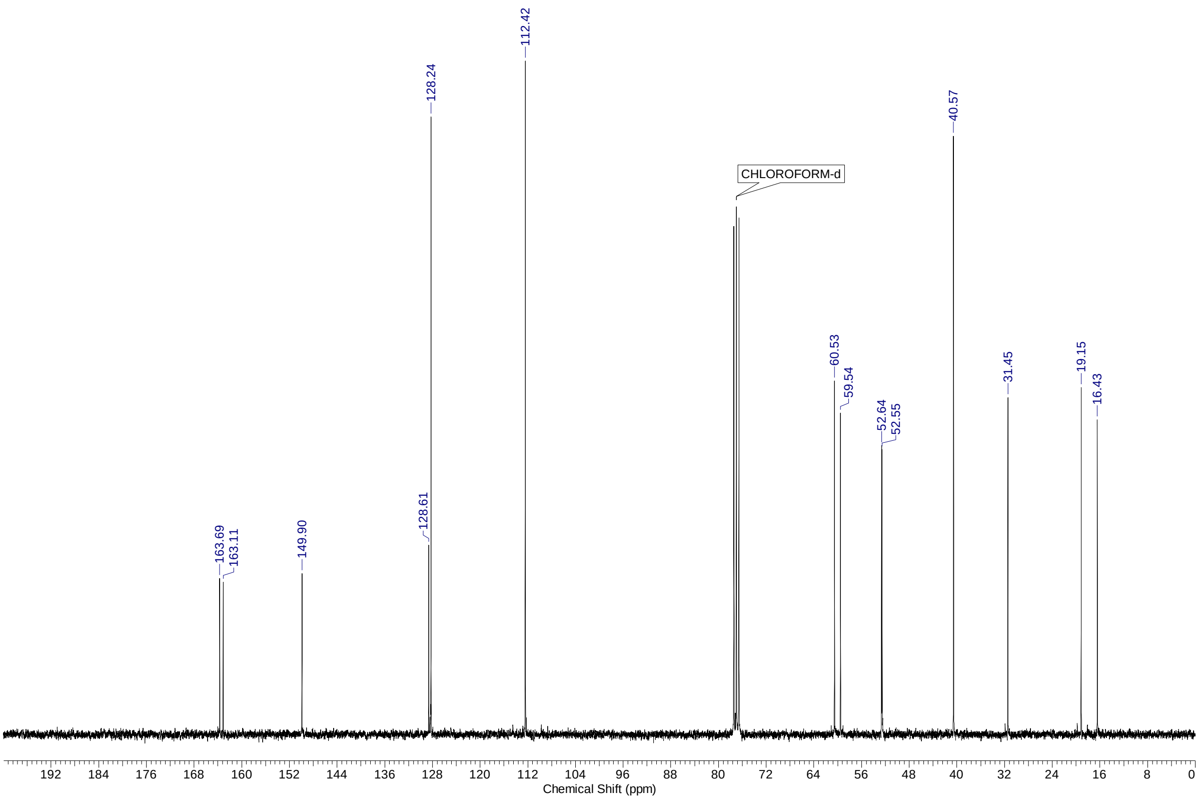

(2R,5S)-2-[4-(dimethoxymethyl)phenyl]-5-isopropyl-3,6-dimethoxy-2,5-dihydropyrazine (**3ka**)

^{1}H NMR (400 MHz, $CDCl_3$)

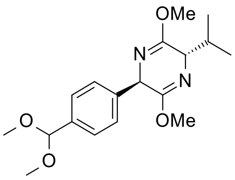

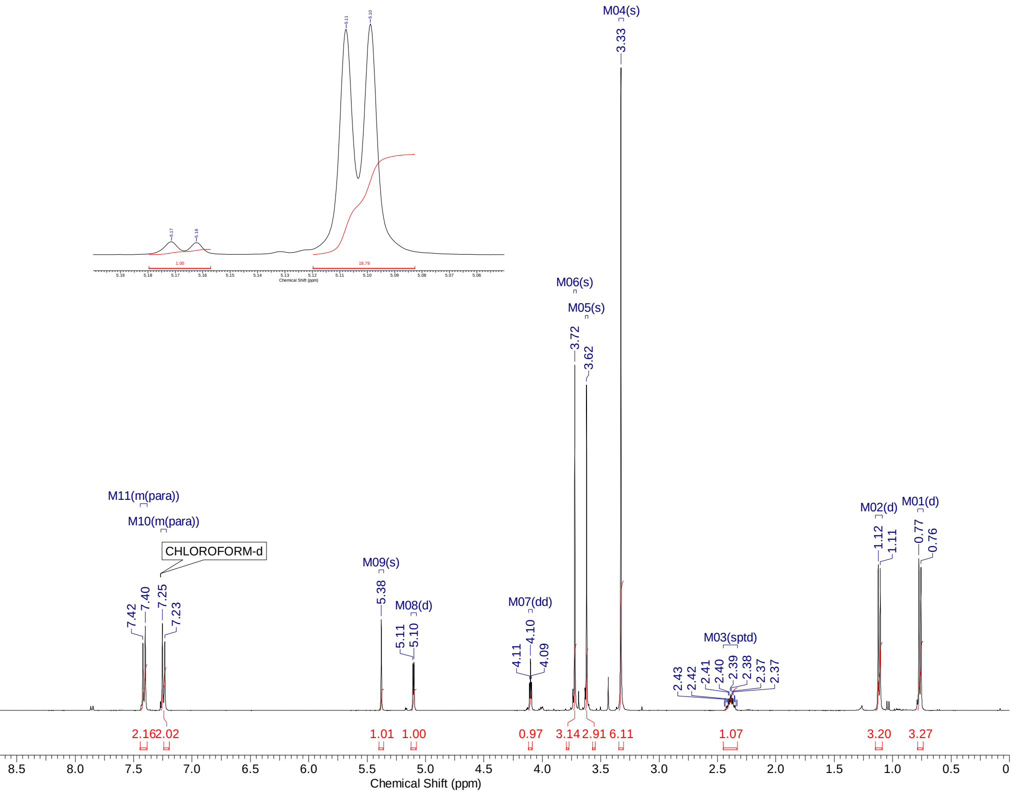

^{13}C NMR (100 MHz, acetone-d6)

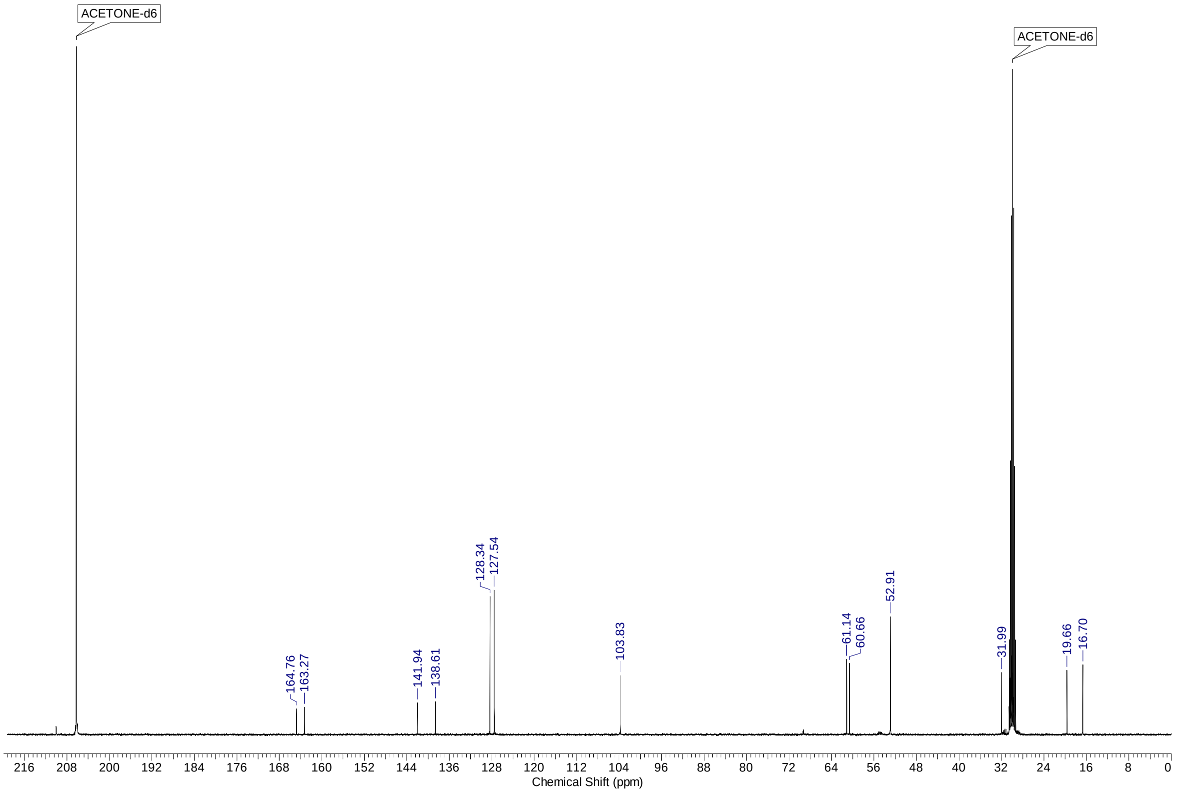

(2*S*,5*R*)-2-isopropyl-3,6-dimethoxy-5-[4-(trifluoromethyl)phenyl]-2,5-dihydropyrazine (**3la**)

^{1}H NMR (300 MHz, $CDCl_3$)

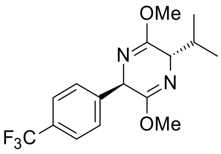

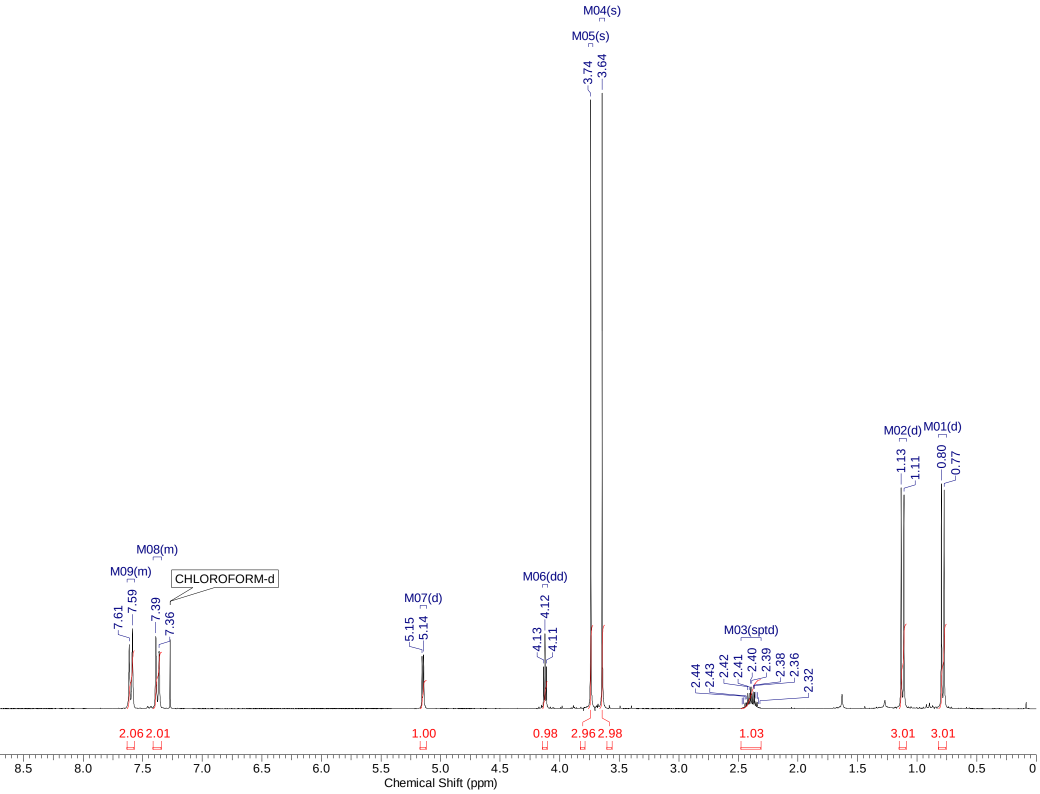

^{13}C NMR (75 MHz, $CDCl_3$)

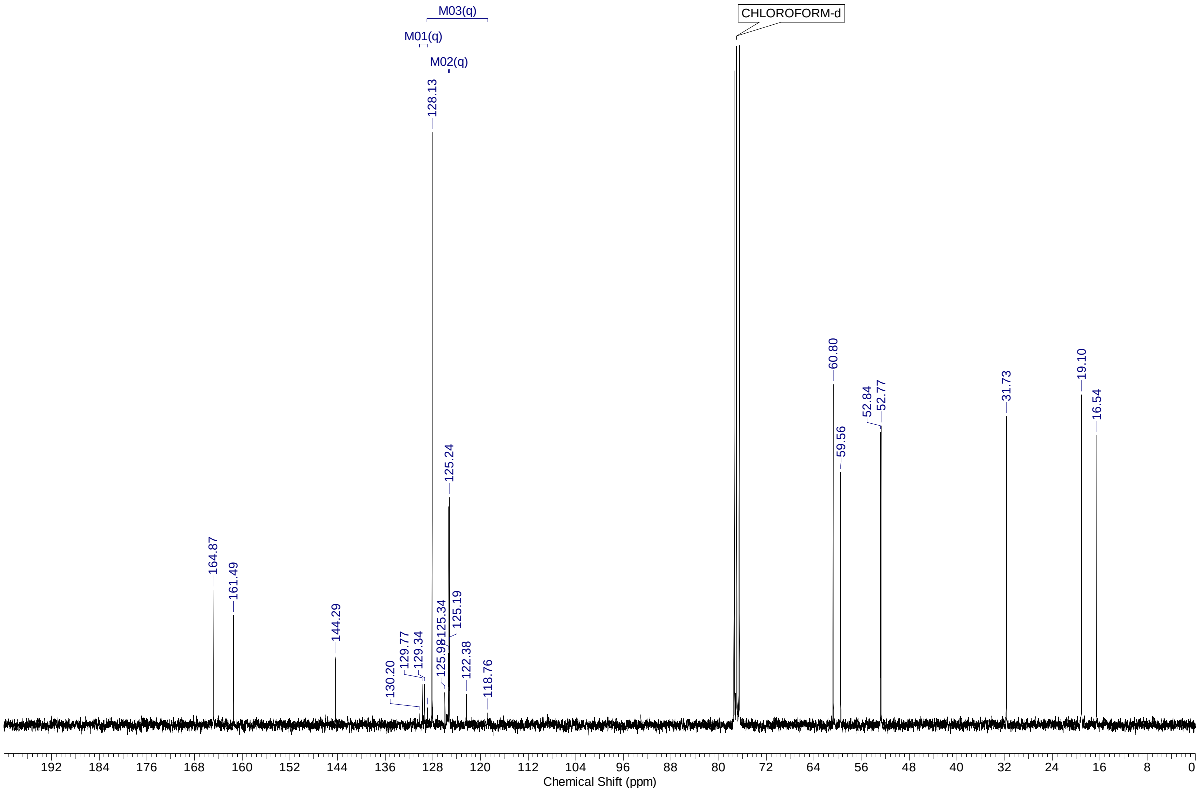

^{19}F NMR (41 MHz, $CDCl_3$)

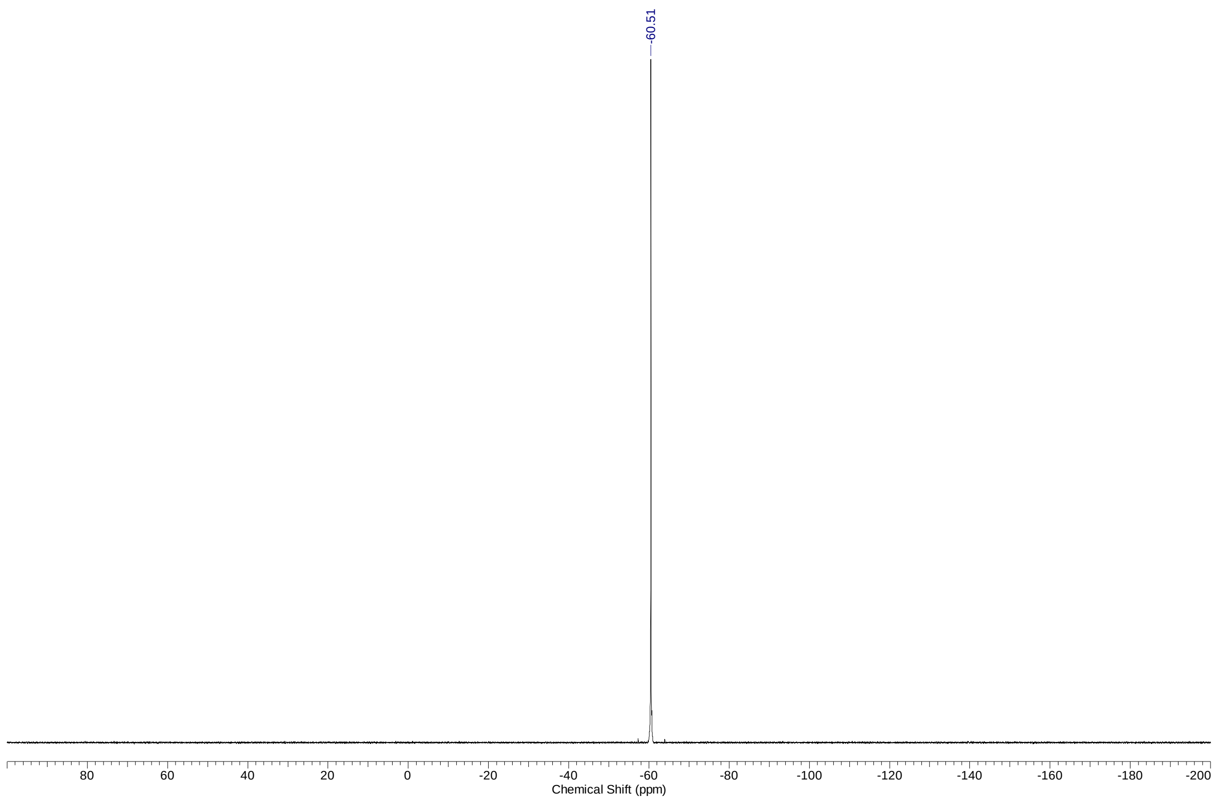

4-[(2*R*,5*S*)-5-isopropyl-3,6-dimethoxy-2,5-dihydropyrazin-2-yl]-*N*,*N*-dimethyl-benzamide (**3ma**)

^{1}H NMR (300 MHz, $CDCl_3$)

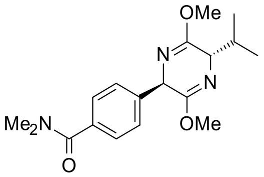

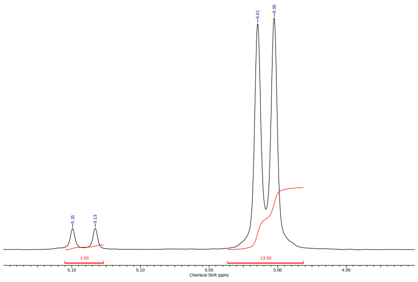

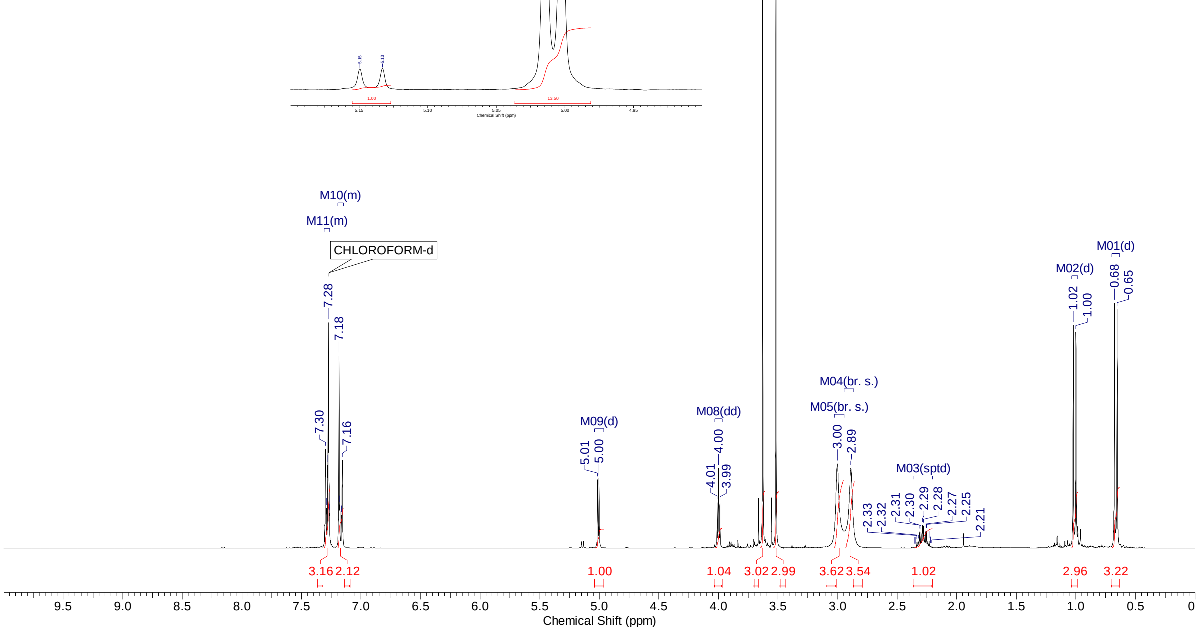

^{13}C NMR (75 MHz, $CDCl_3$)

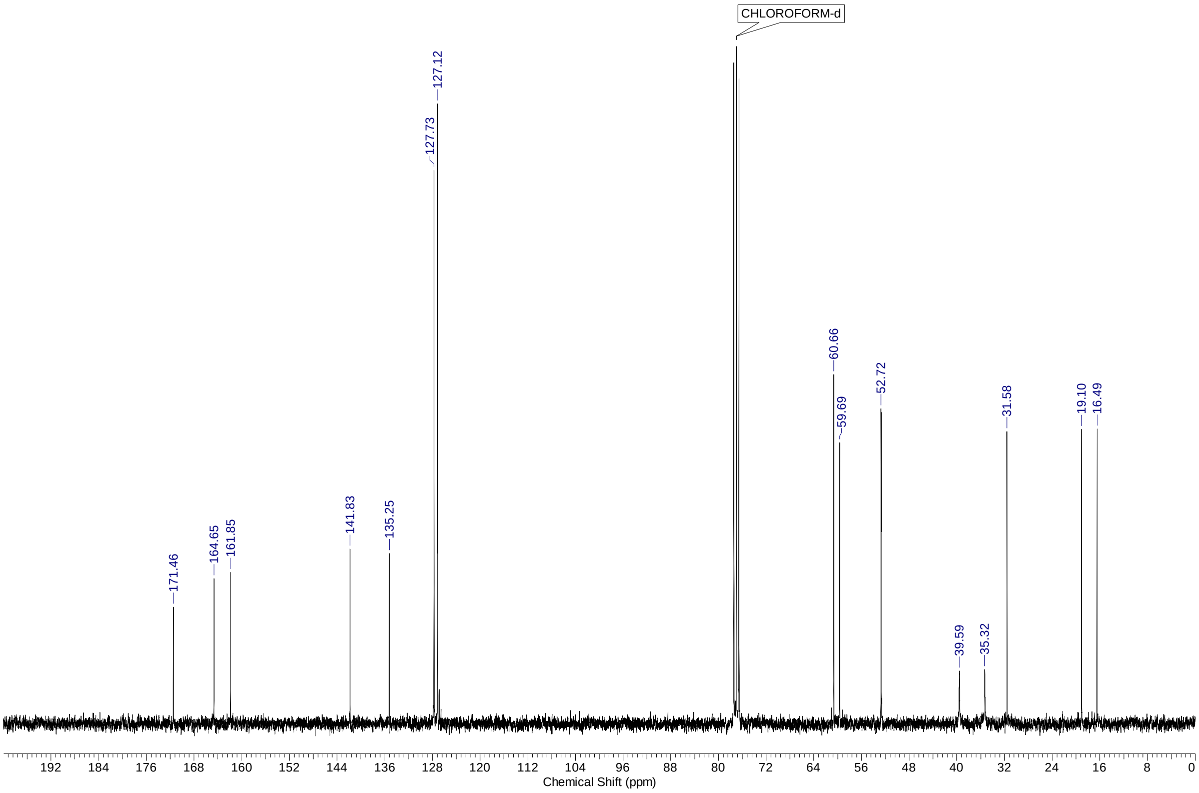

ethyl 4-[(2*R*,5*S*)-5-isopropyl-3,6-dimethoxy-2,5-dihydropyrazin-2-yl]benzoate (**3na**)

^{1}H NMR (300 MHz, $CDCl_3$)

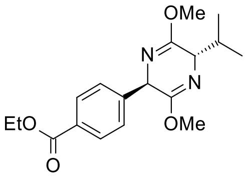

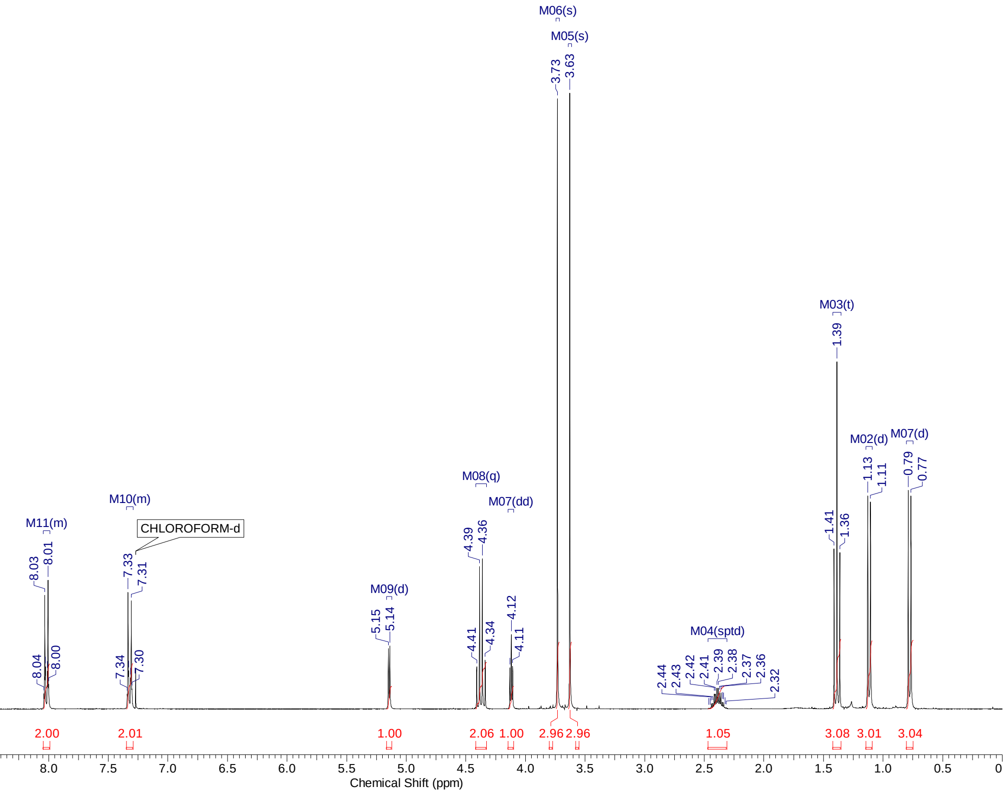

^{13}C NMR (75 MHz, $CDCl_3$)

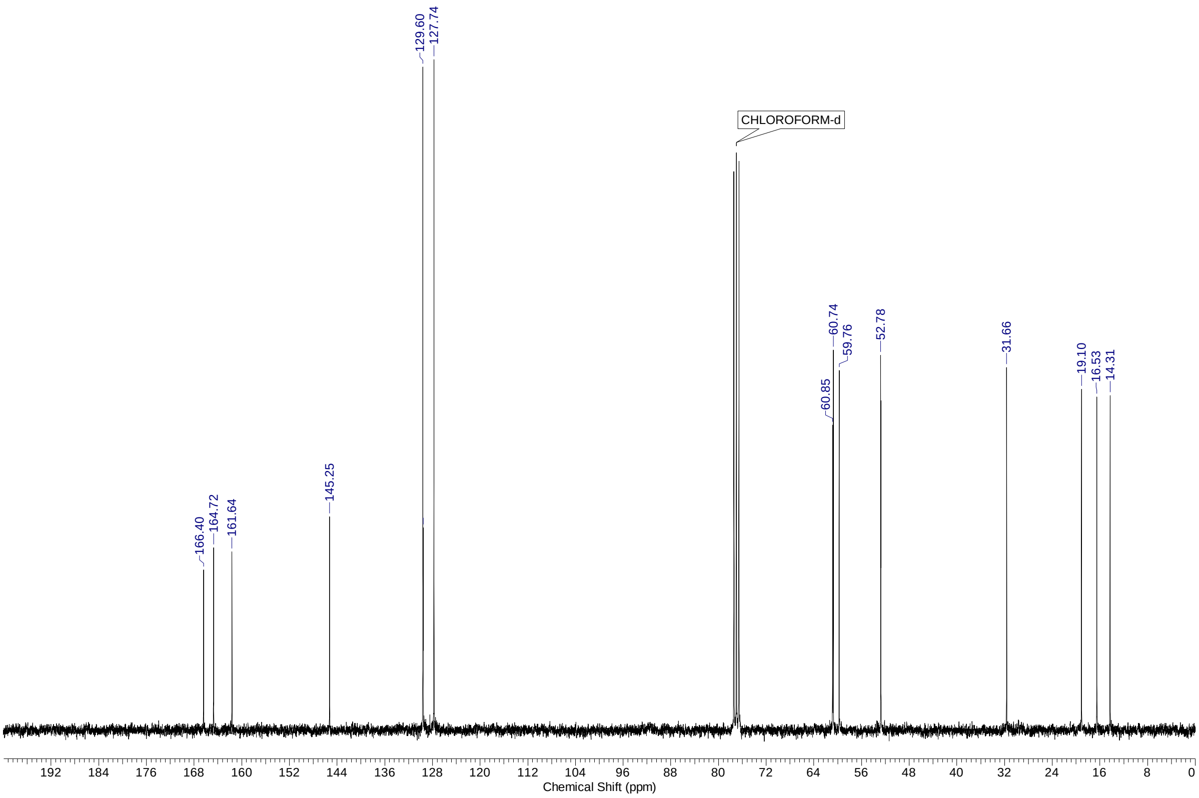

[4-[(2*R*,5*S*)-5-isopropyl-3,6-dimethoxy-2,5-dihydropyrazin-2-yl]phenyl]-phenyl-methanone (**3oa**)

^{1}H NMR (300 MHz, $CDCl_3$)

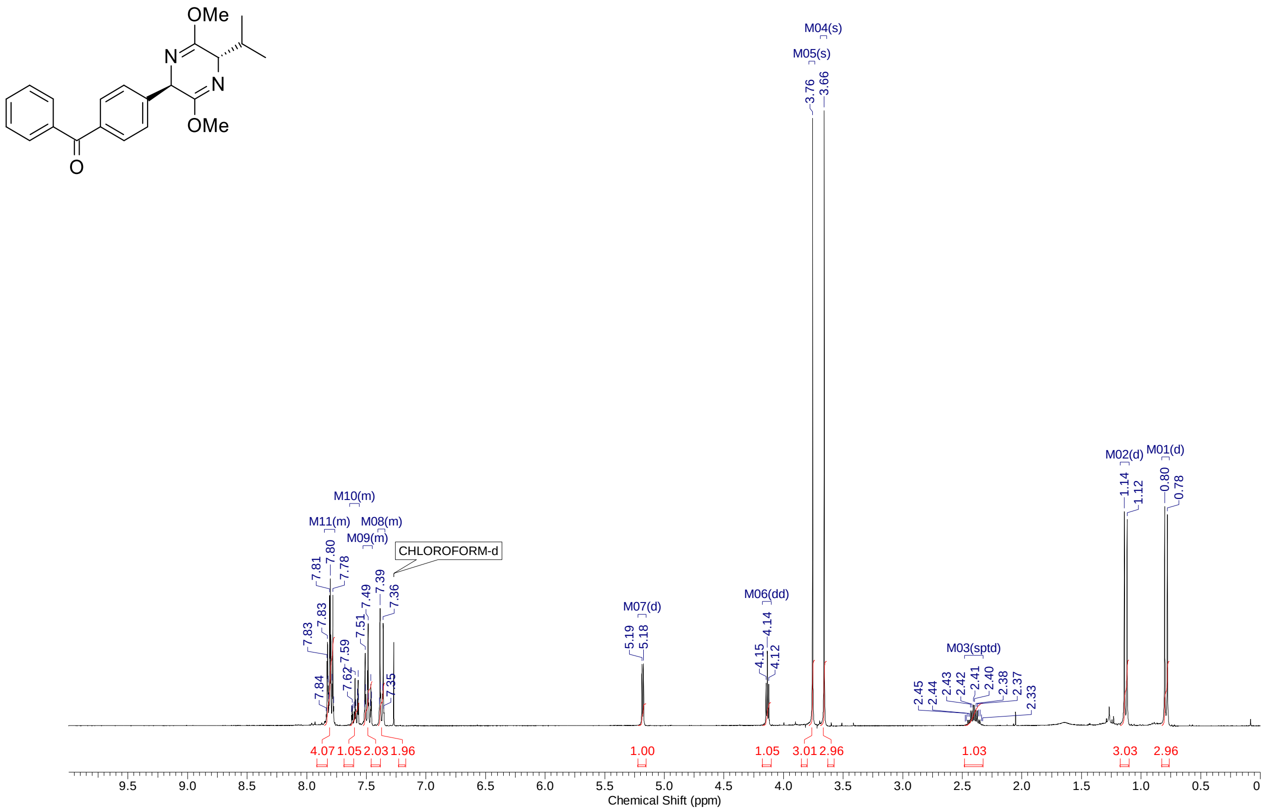

^{13}C NMR (75 MHz, $CDCl_3$)

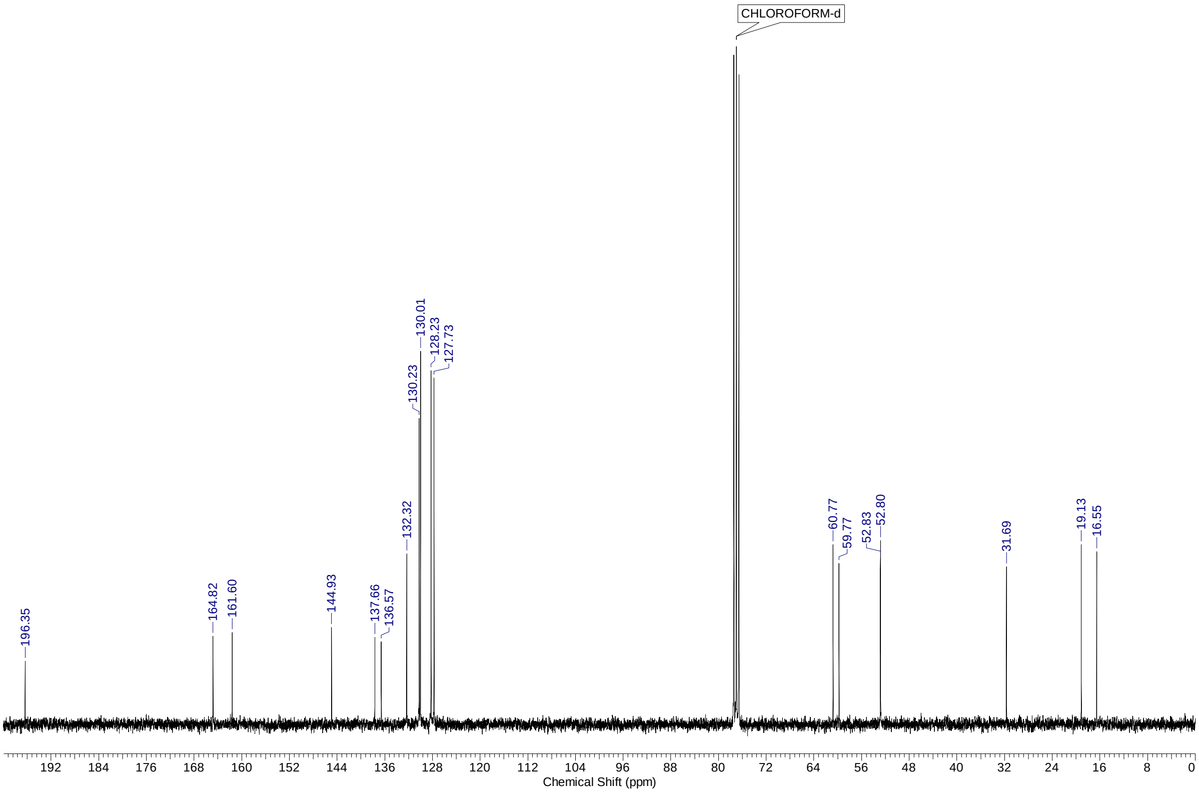

(2*S*,5*R*)-2-isopropyl-3,6-dimethoxy-5-[4-(4,4,5,5-tetramethyl-1,3,2-dioxaborolan-2-yl)phenyl]-2,5-dihydropyrazine (**3pa**)

^{1}H NMR (300 MHz, $CDCl_3$)

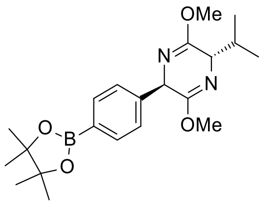

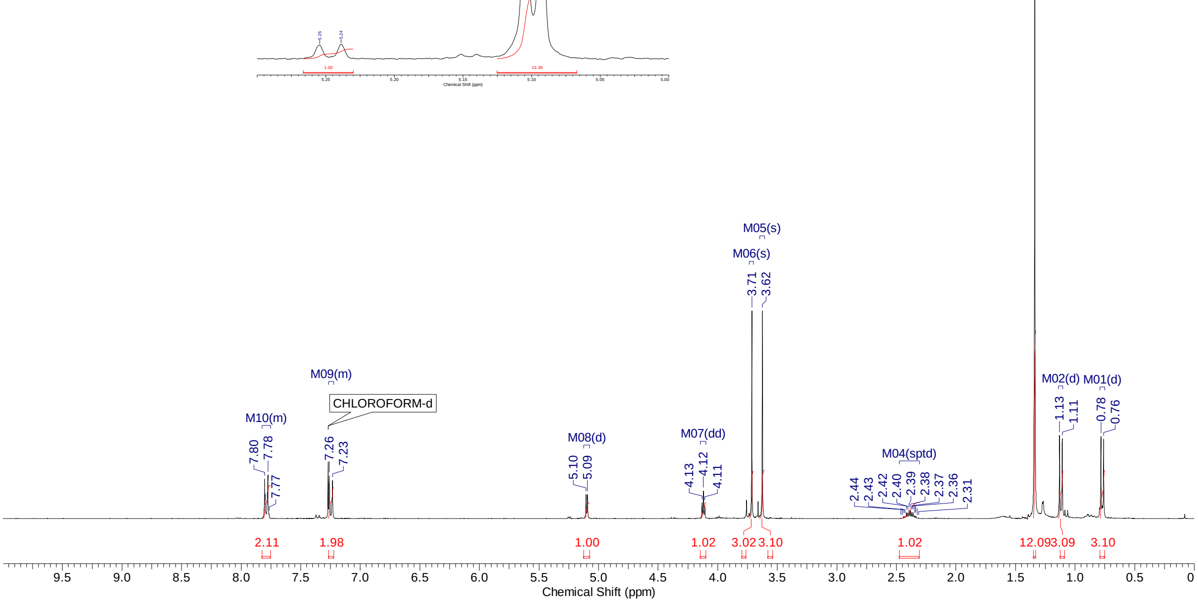

^{13}C NMR (75 MHz, $CDCl_3$)

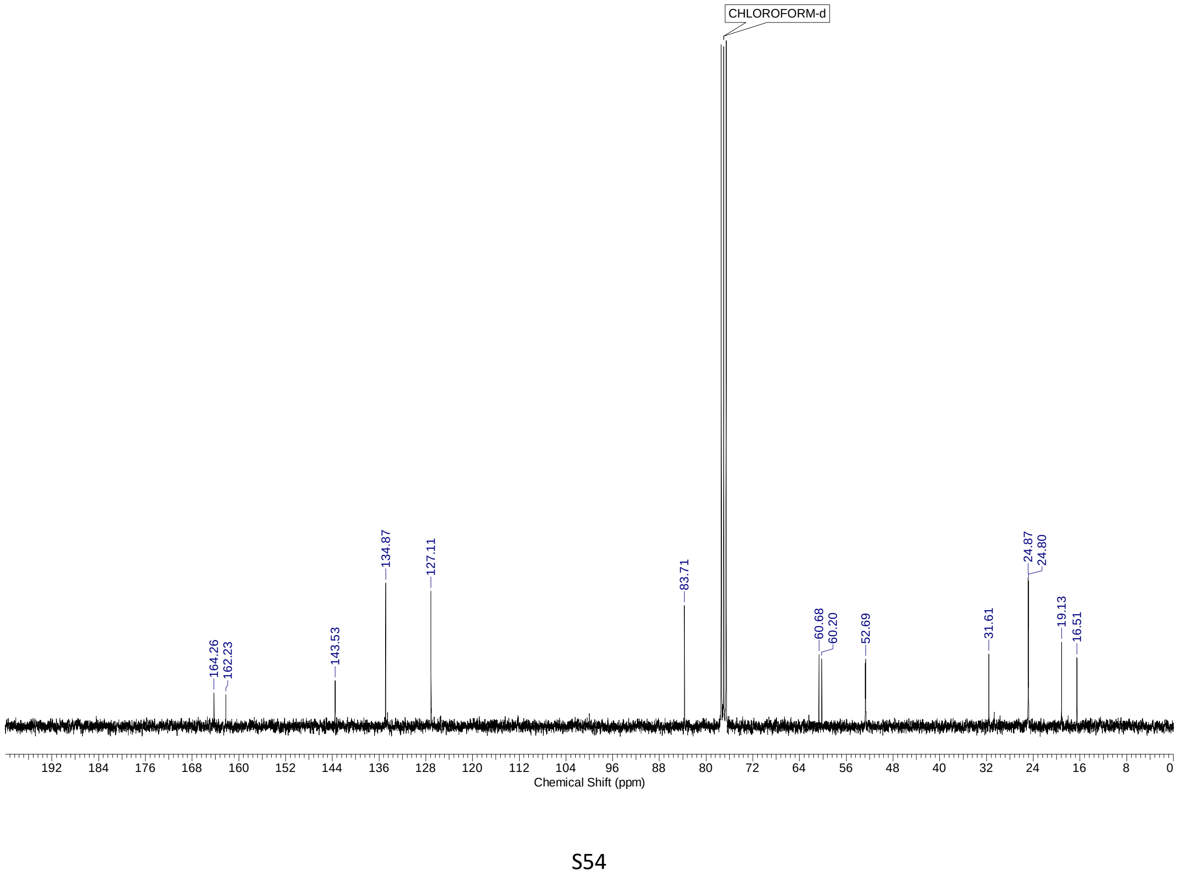

4-[(2*R*,5*S*)-5-isopropyl-3,6-dimethoxy-2,5-dihydropyrazin-2-yl]phenyl]-trimethyl-silane (**3qa**)

^{1}H NMR (300 MHz, CDCl$_3$)

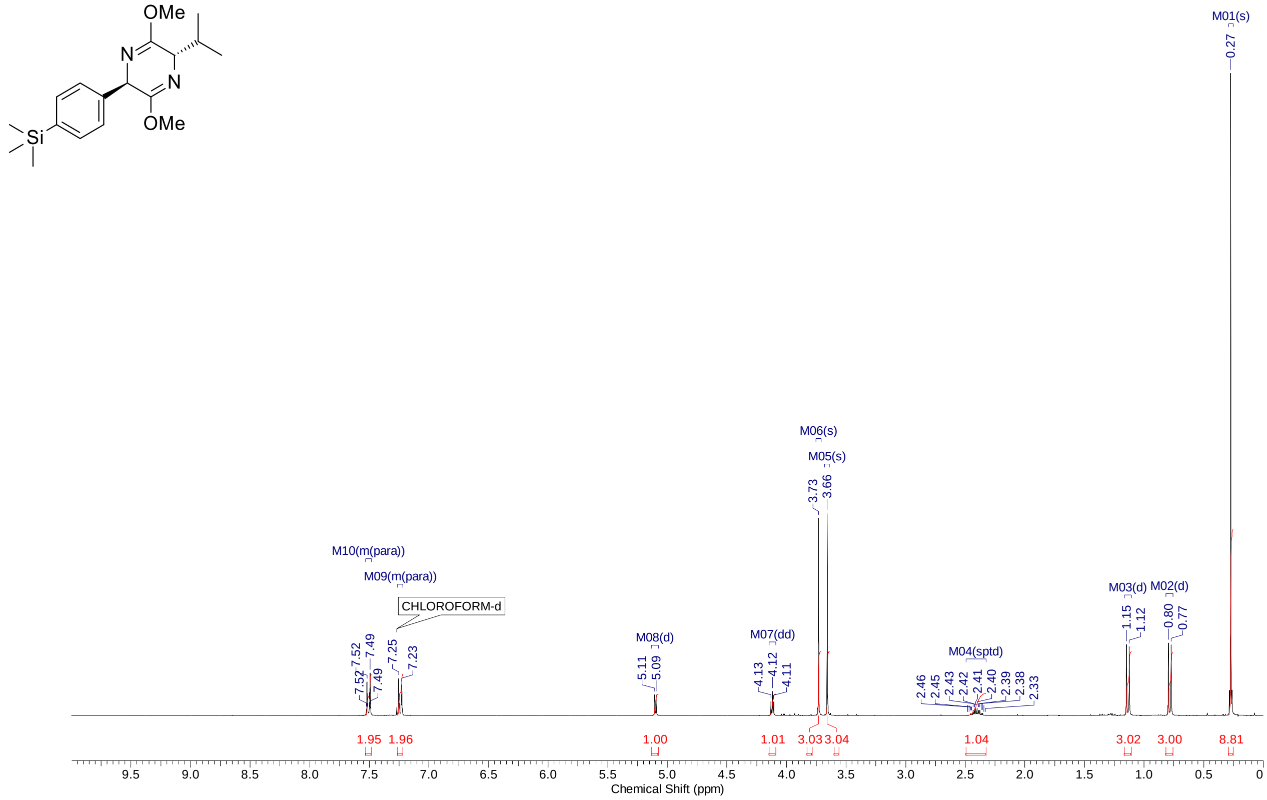

^{13}C NMR (75 MHz, CDCl$_3$)

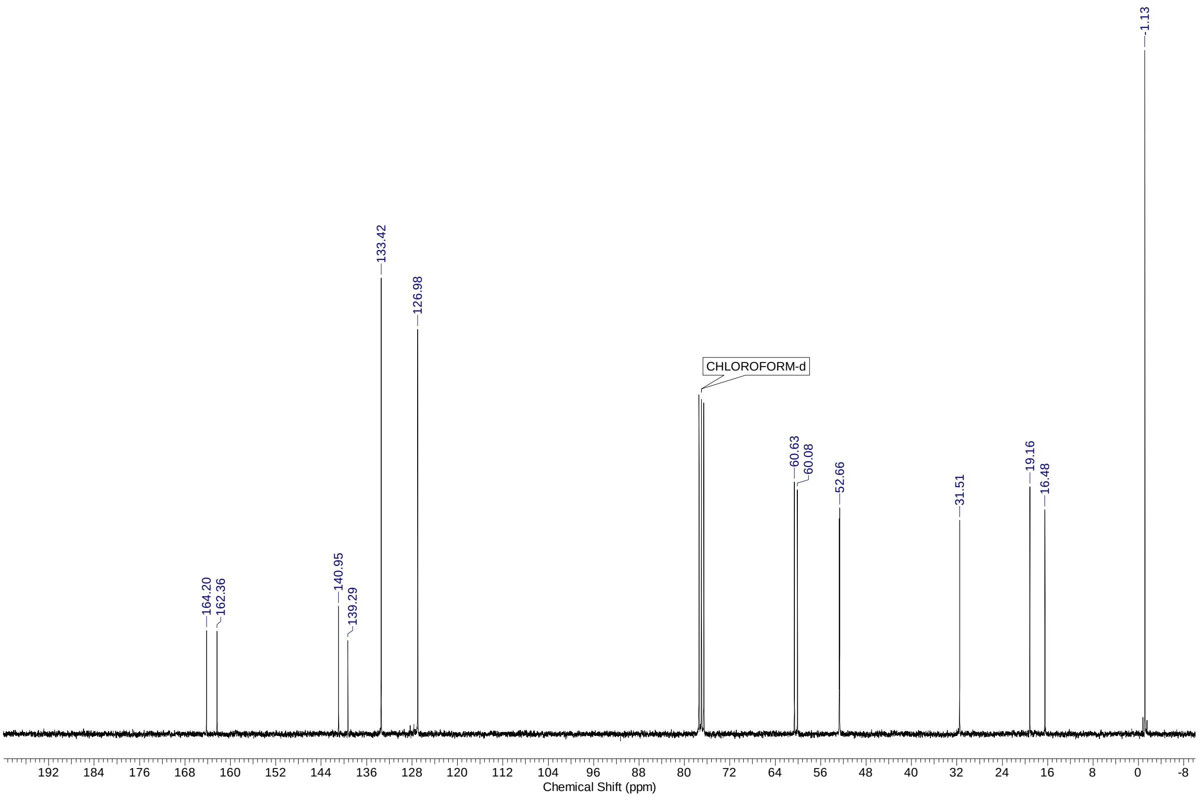

[4-[(2*R*,5*S*)-5-isopropyl-3,6-dimethoxy-2,5-dihydropyrazin-2-yl]phenyl] methanesulfonate (**3ra**)

^{1}H NMR (300 MHz, $CDCl_3$)

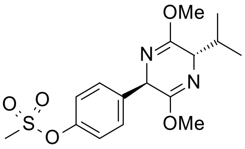

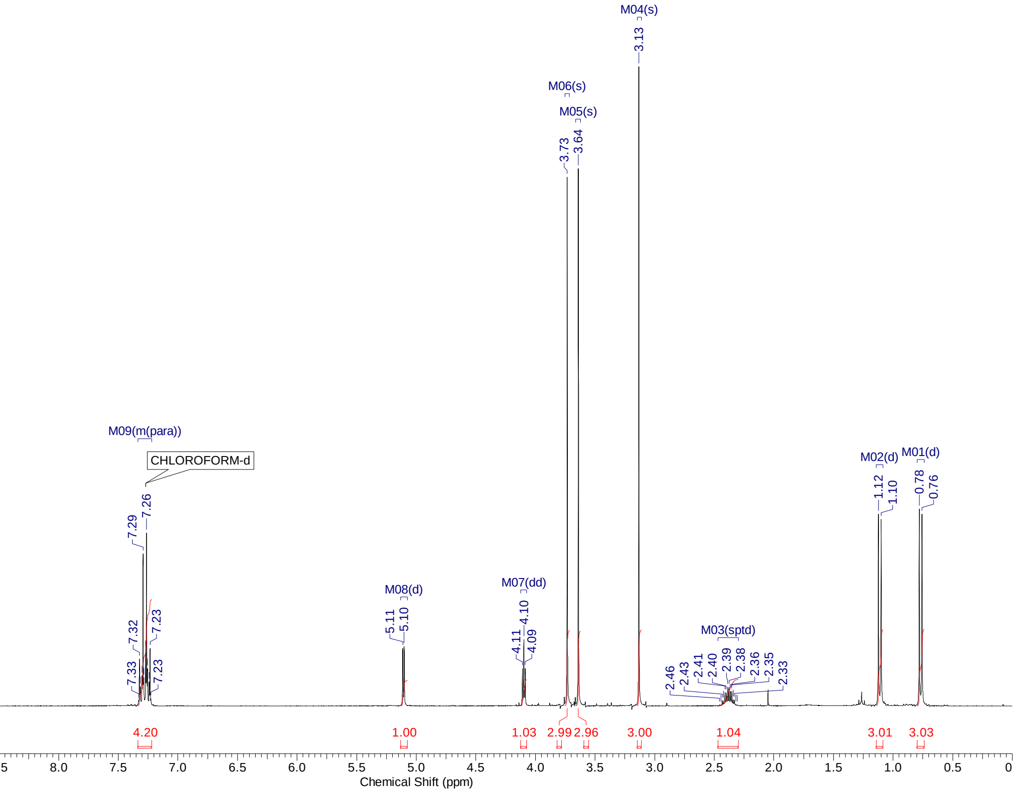

^{13}C NMR (75 MHz, $CDCl_3$)

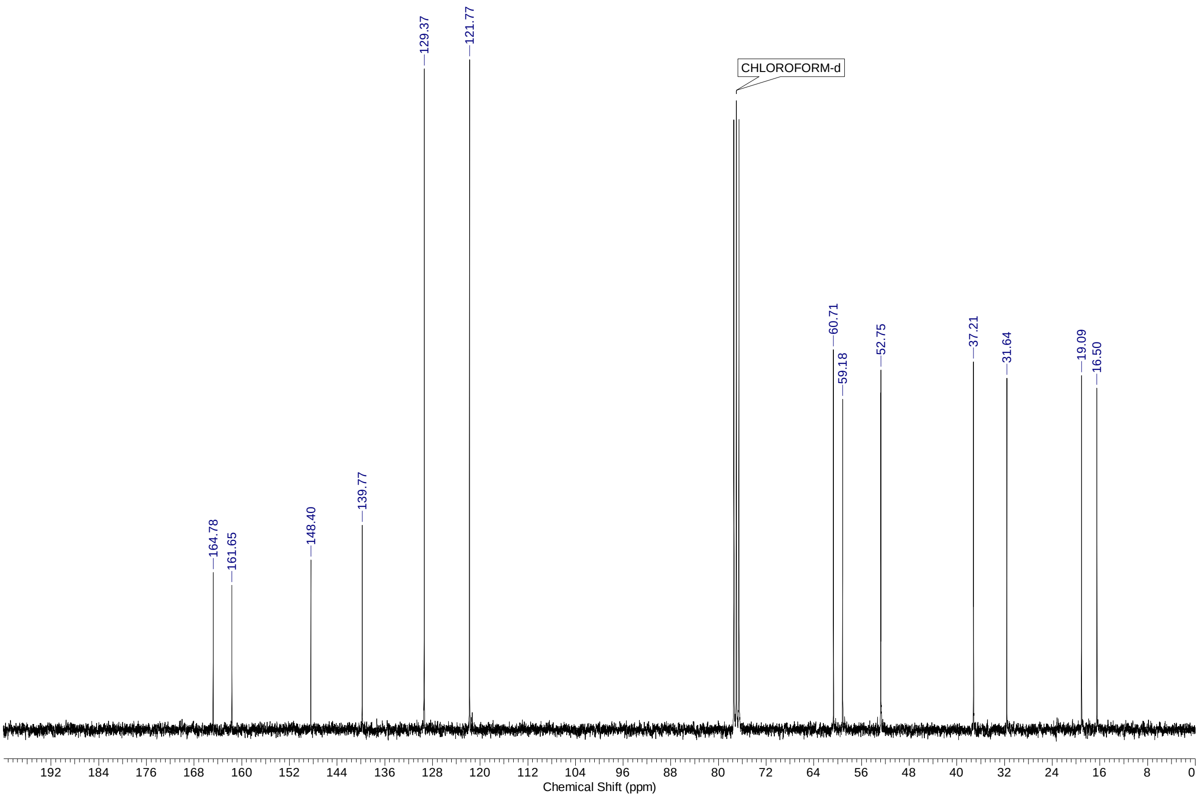

2-[4-[(2*R*,5*S*)-5-isopropyl-3,6-dimethoxy-2,5-dihydropyrazin-2-yl]phenyl]ethynyl-trimethyl-silane (**3sa**)

^{1}H NMR (300 MHz, $CDCl_3$)

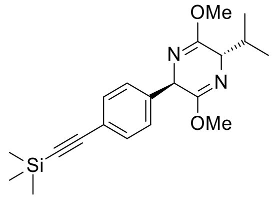

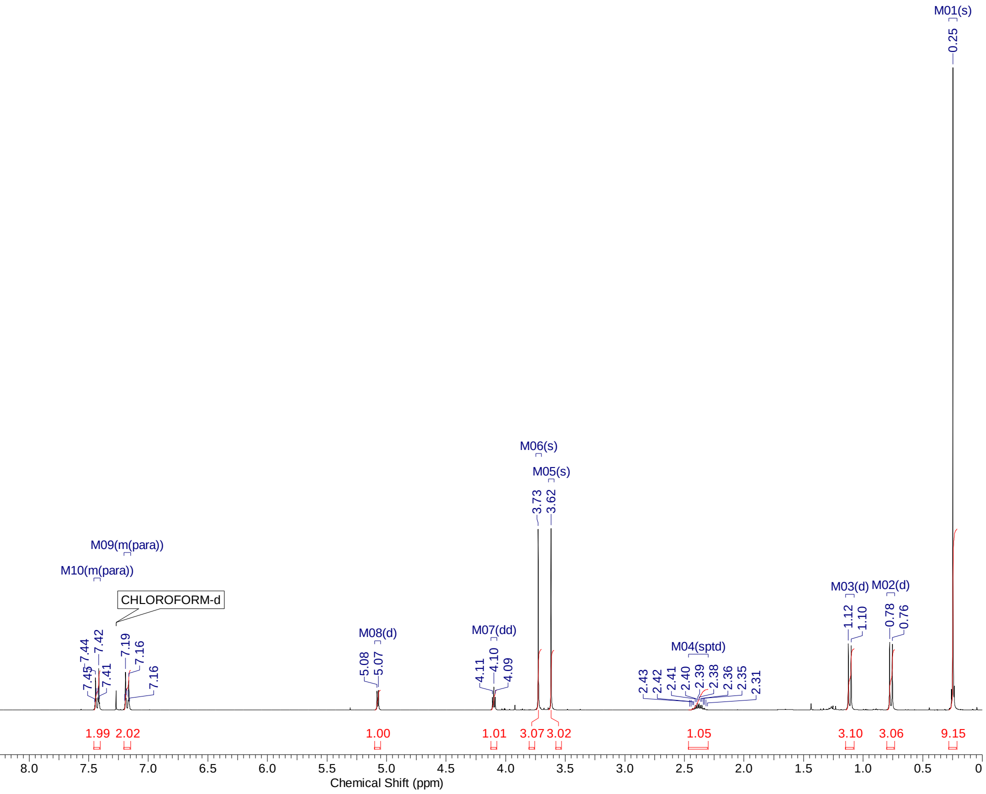

^{13}C NMR (75 MHz, $CDCl_3$)

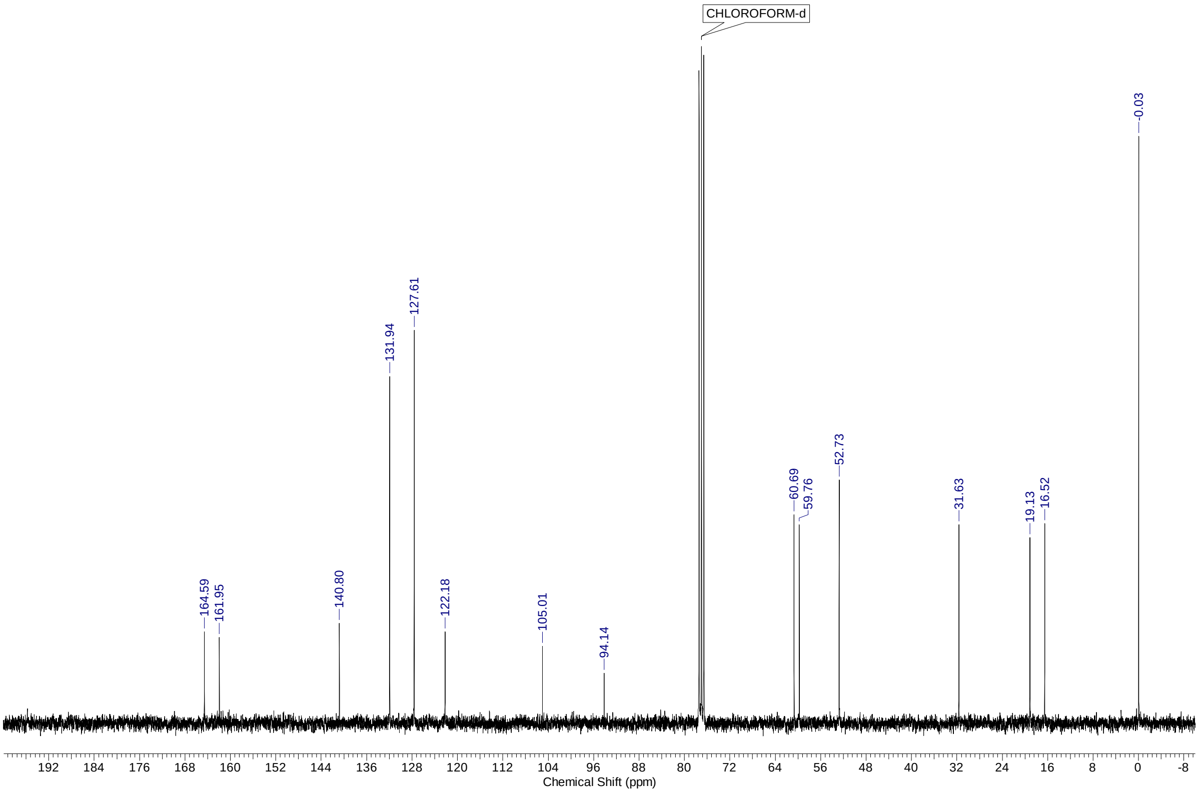

(2*S*,5*R*)-2-isopropyl-3,6-dimethoxy-5-(3-pyridyl)-2,5-dihydropyrazine (**3ta**)

^{1}H NMR (300 MHz, $CDCl_3$)

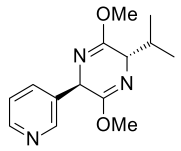

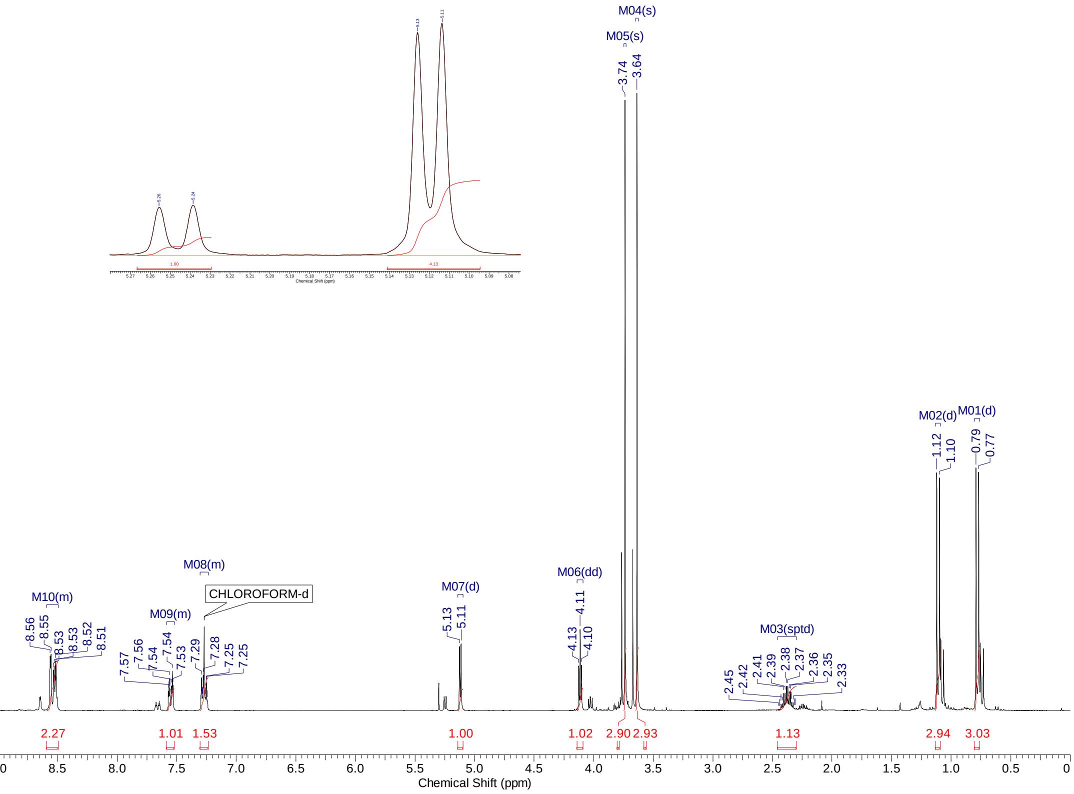

^{13}C NMR (75 MHz, $CDCl_3$)

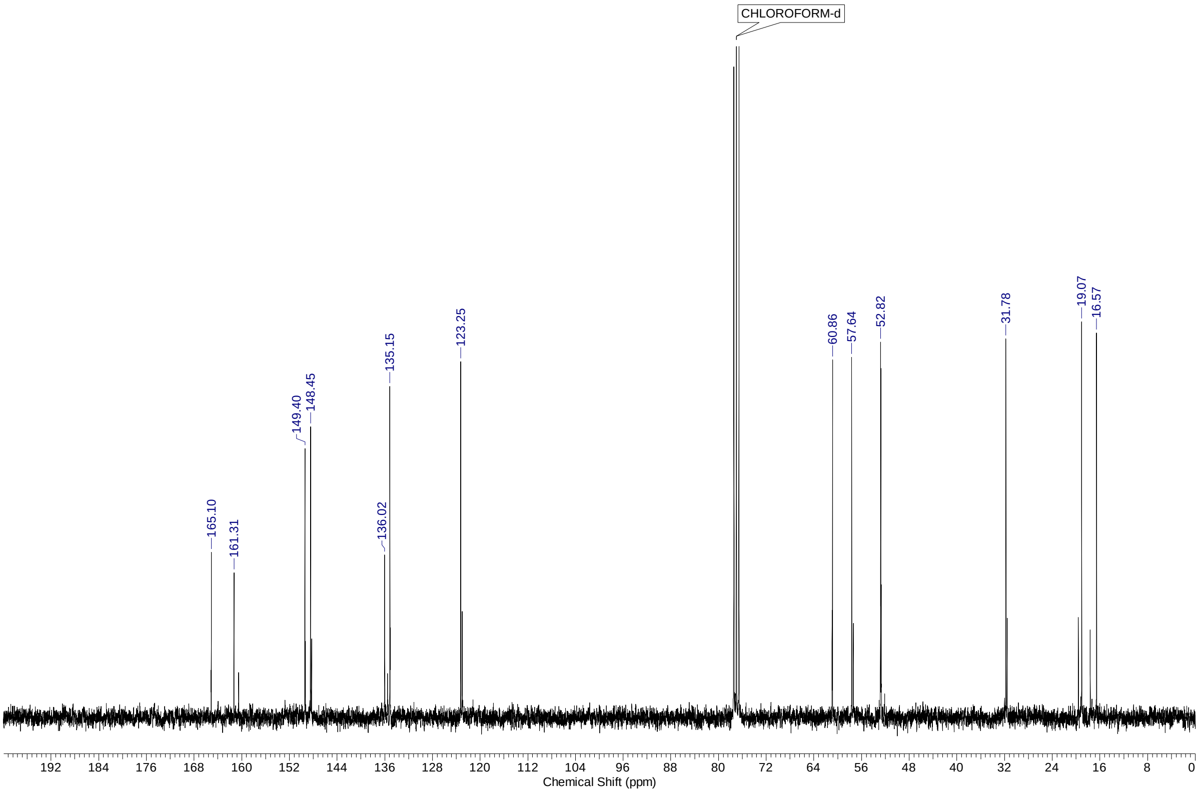

(2S,5R)-2-isopropyl-3,6-dimethoxy-5-(3-thienyl)-2,5-dihydropyrazine (**3ua**)

^{1}H NMR (300 MHz, $CDCl_3$)

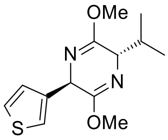

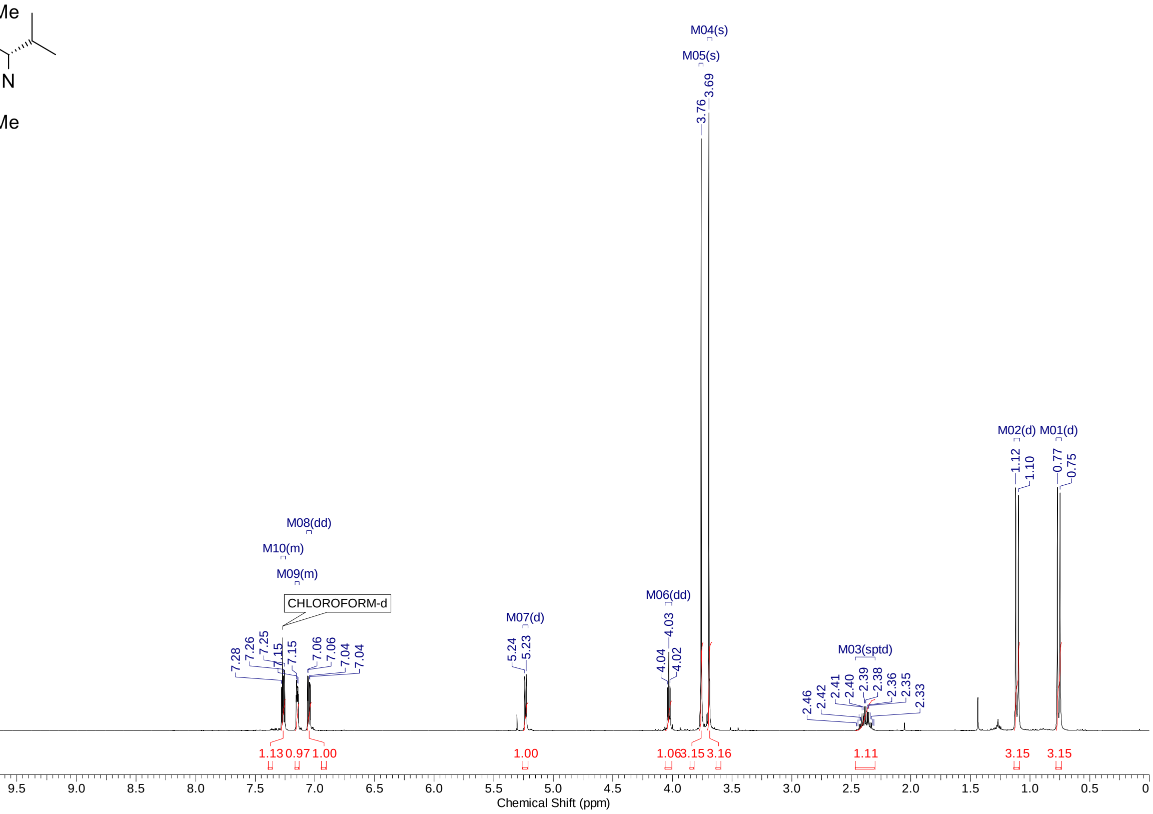

^{13}C NMR (75 MHz, $CDCl_3$)

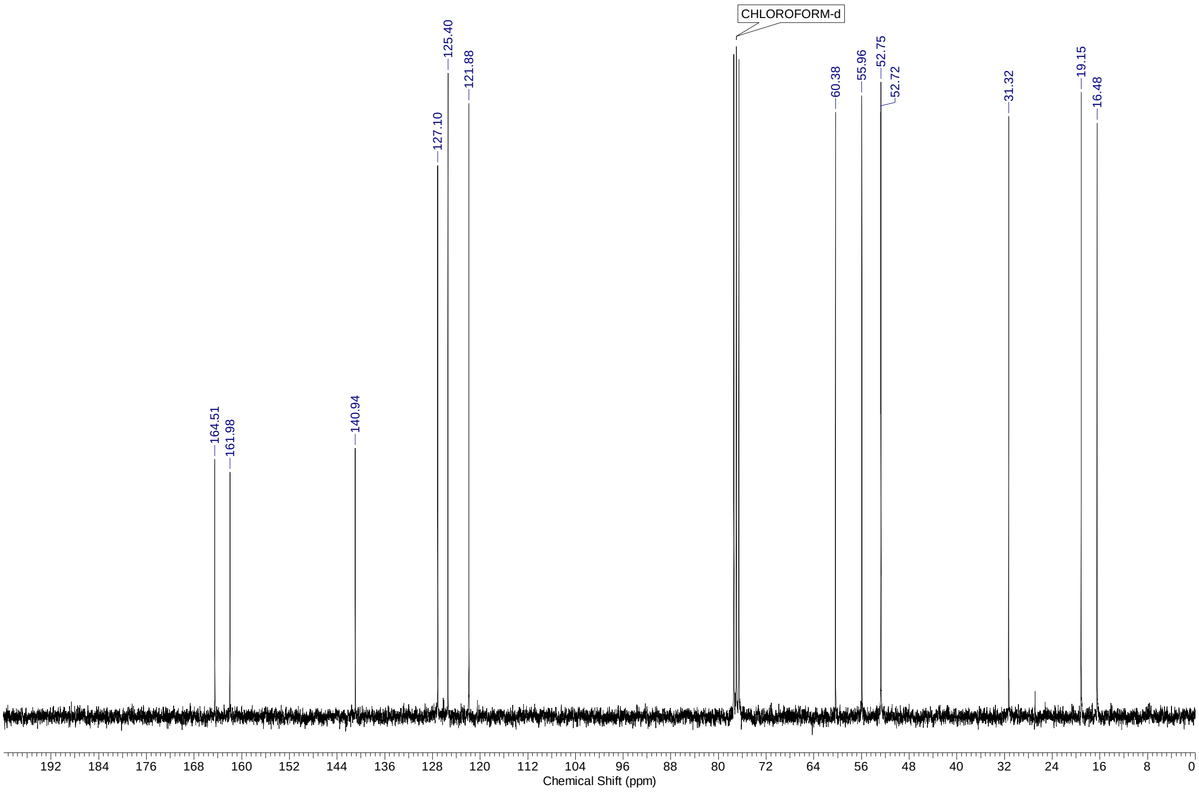

6-[(2*R*,5*S*)-5-isopropyl-3,6-dimethoxy-2,5-dihydropyrazin-2-yl]quinoline (**3va**)

^{1}H NMR (300 MHz, $CDCl_3$)

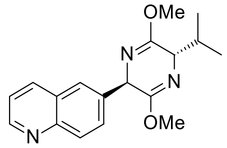

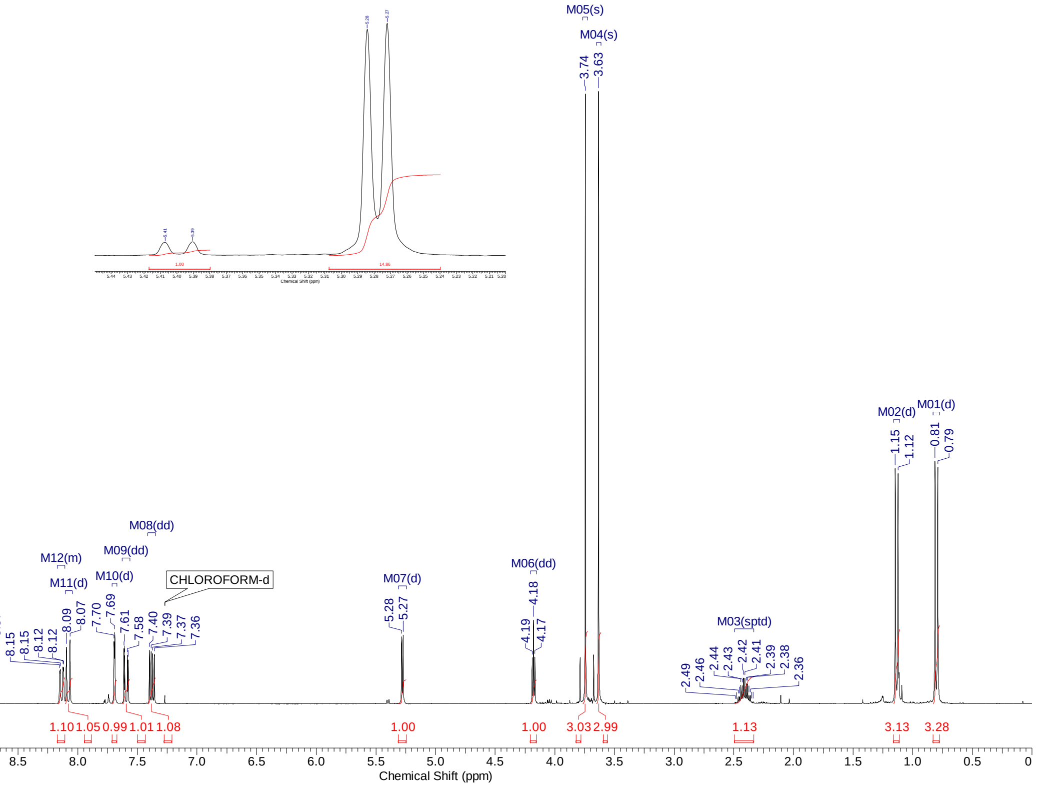

^{13}C NMR (75 MHz, $CDCl_3$)

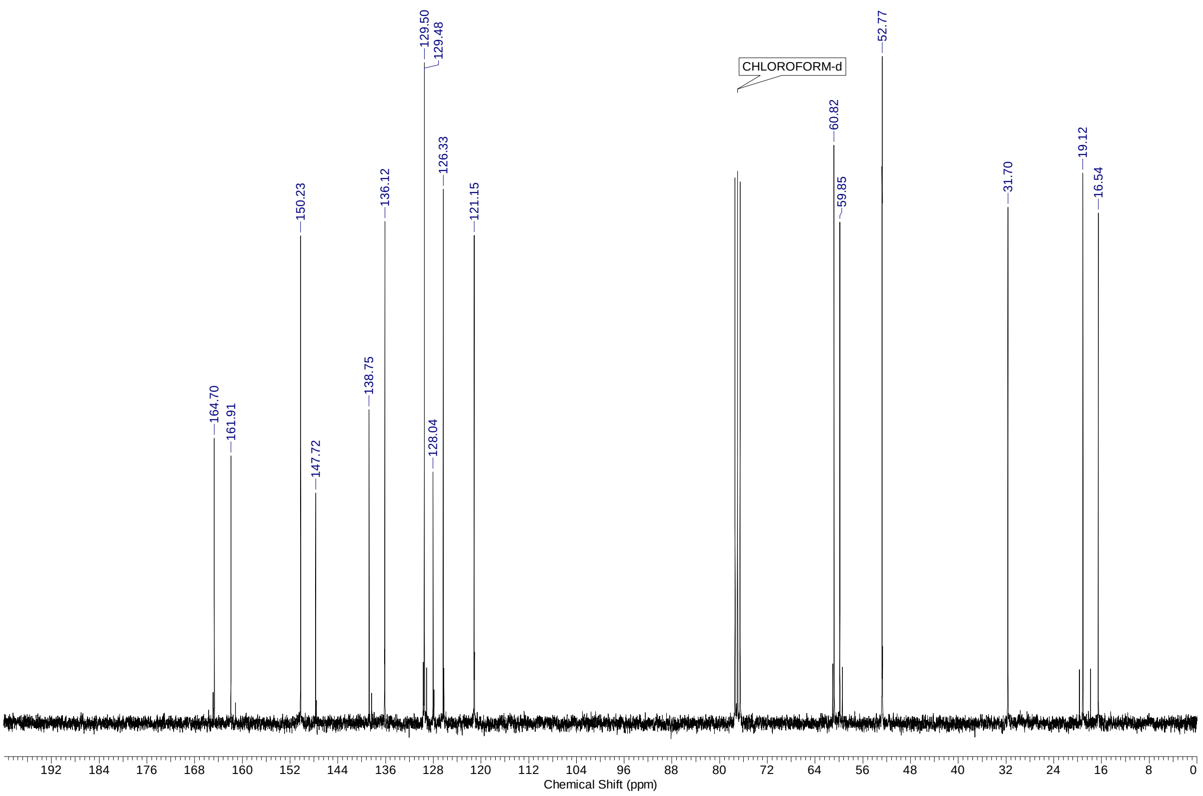

(2*S*,5*R*)-2-isopropyl-3,6-dimethoxy-5-(3-methylbenzothiophen-5-yl)-2,5-dihydropyrazine (**3wa**)

^{1}H NMR (300 MHz, $CDCl_3$)

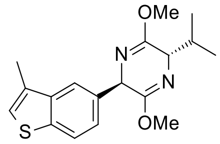

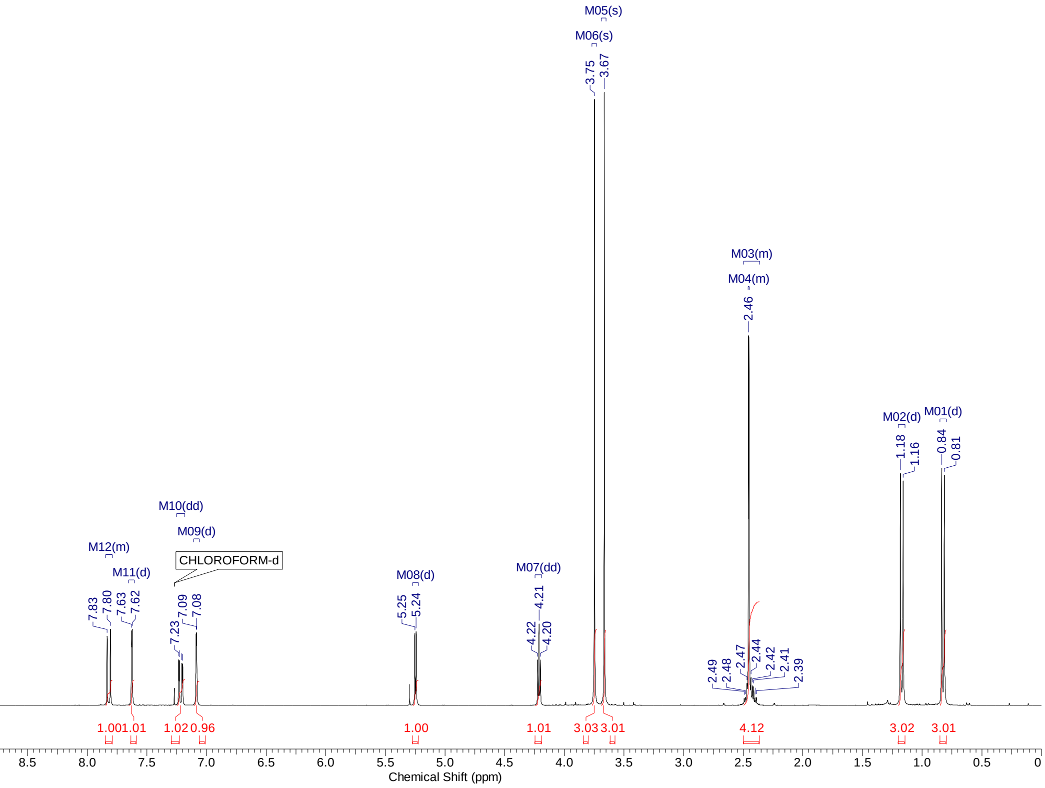

^{13}C NMR (75 MHz, $CDCl_3$)

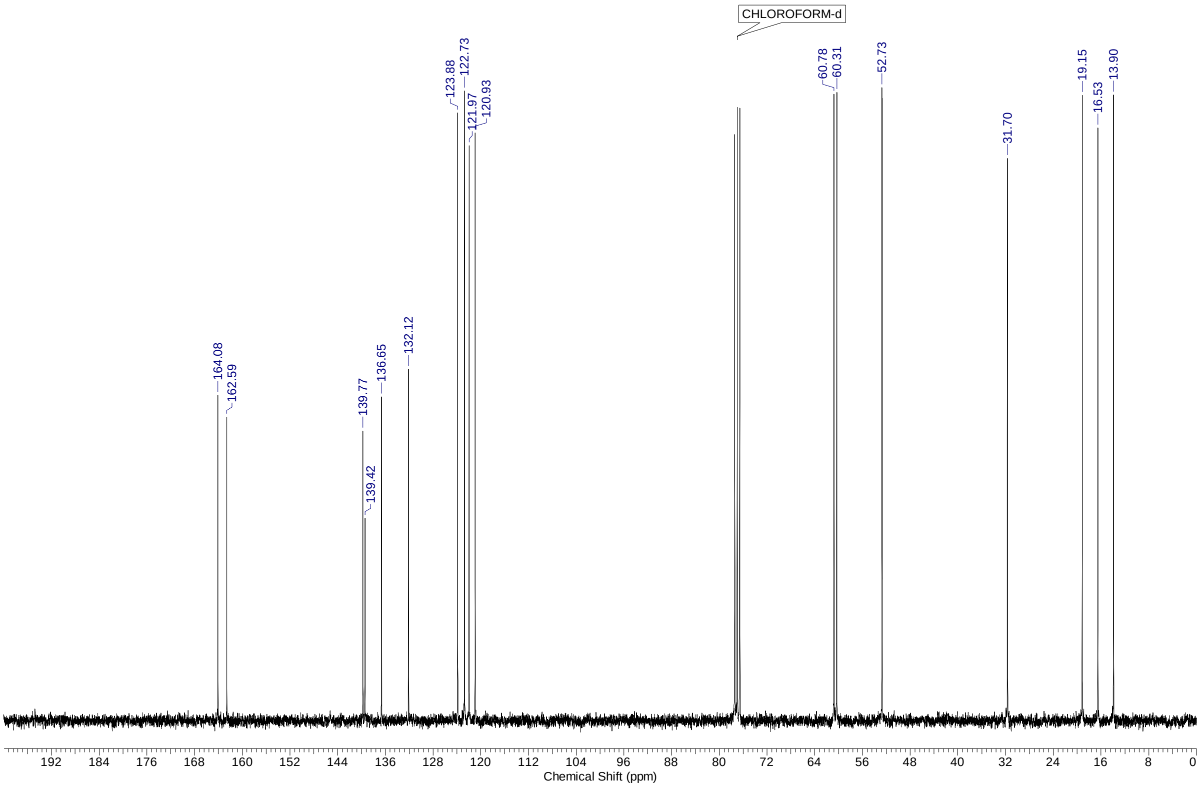

(2*R*,5*S*)-2-(1,3-benzodioxol-5-yl)-5-isopropyl-3,6-dimethoxy-2,5-dihydropyrazine (**3xa**)

^{1}H NMR (300 MHz, $CDCl_3$)

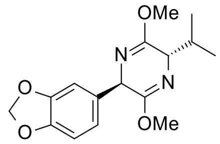

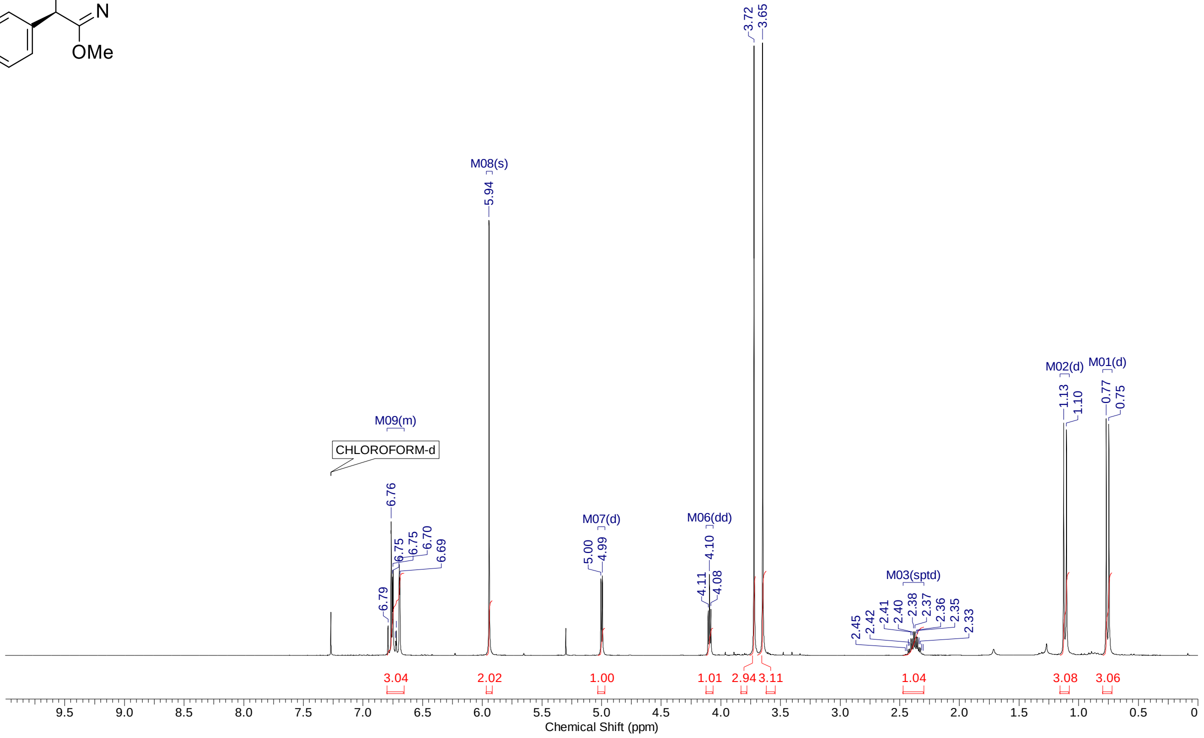

^{13}C NMR (75 MHz, $CDCl_3$)

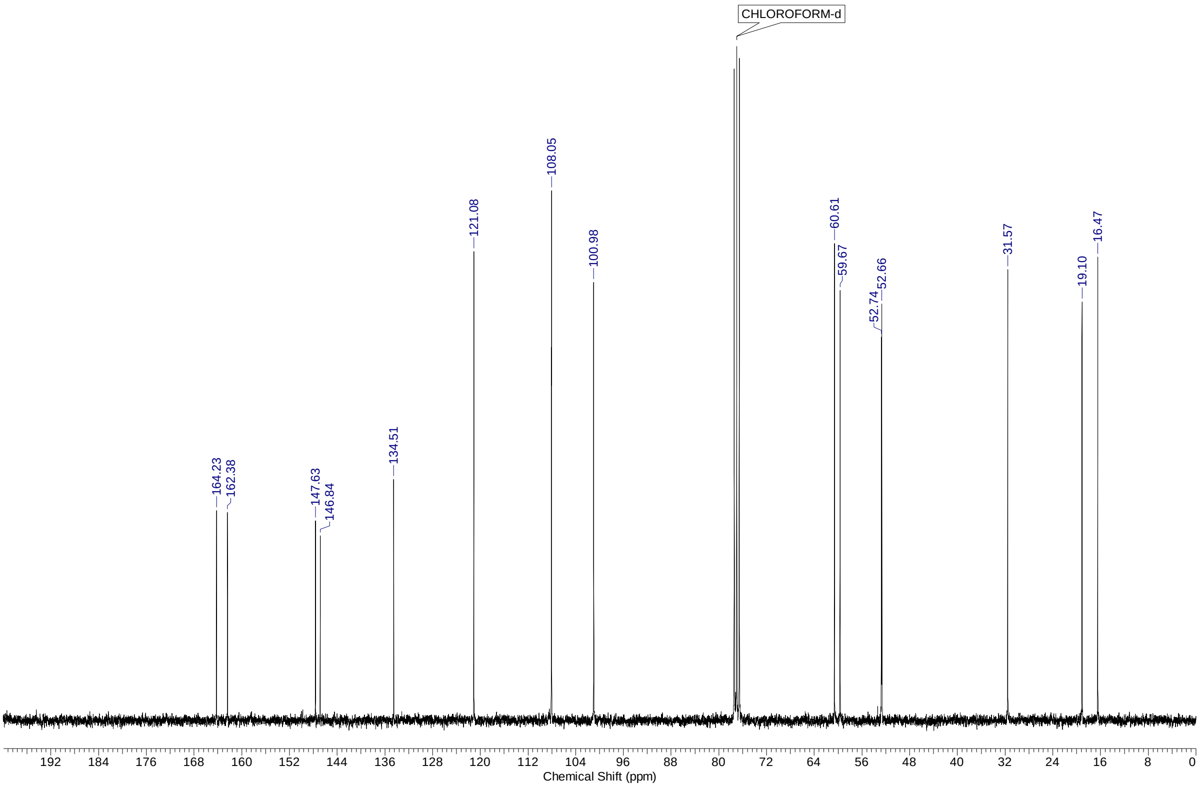

(2*R*,5*S*)-2-(3-bicyclo[3.2.1]oct-2-enyl)-5-isopropyl-3,6-dimethoxy-2,5-dihydropyrazine (**3ya**)

^{1}H NMR (300 MHz, $CDCl_3$)

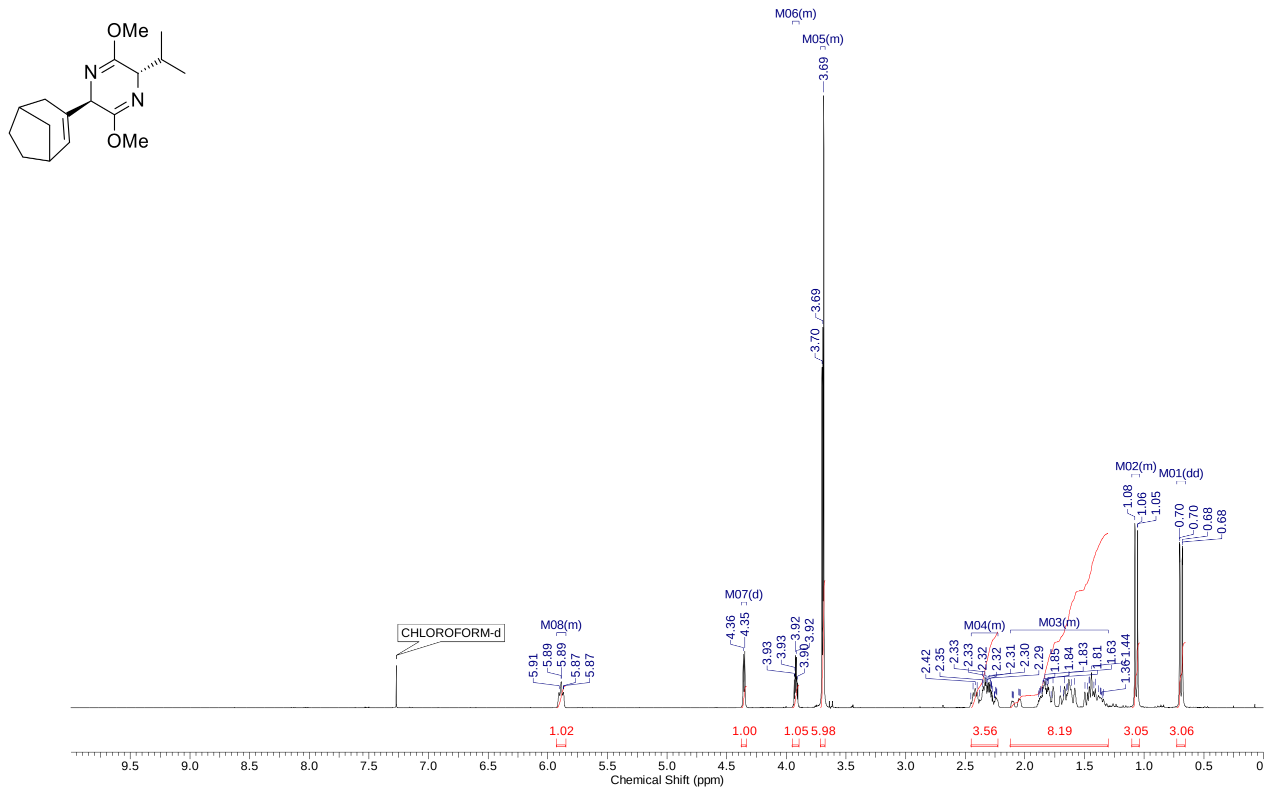

^{13}C NMR (75 MHz, $CDCl_3$)

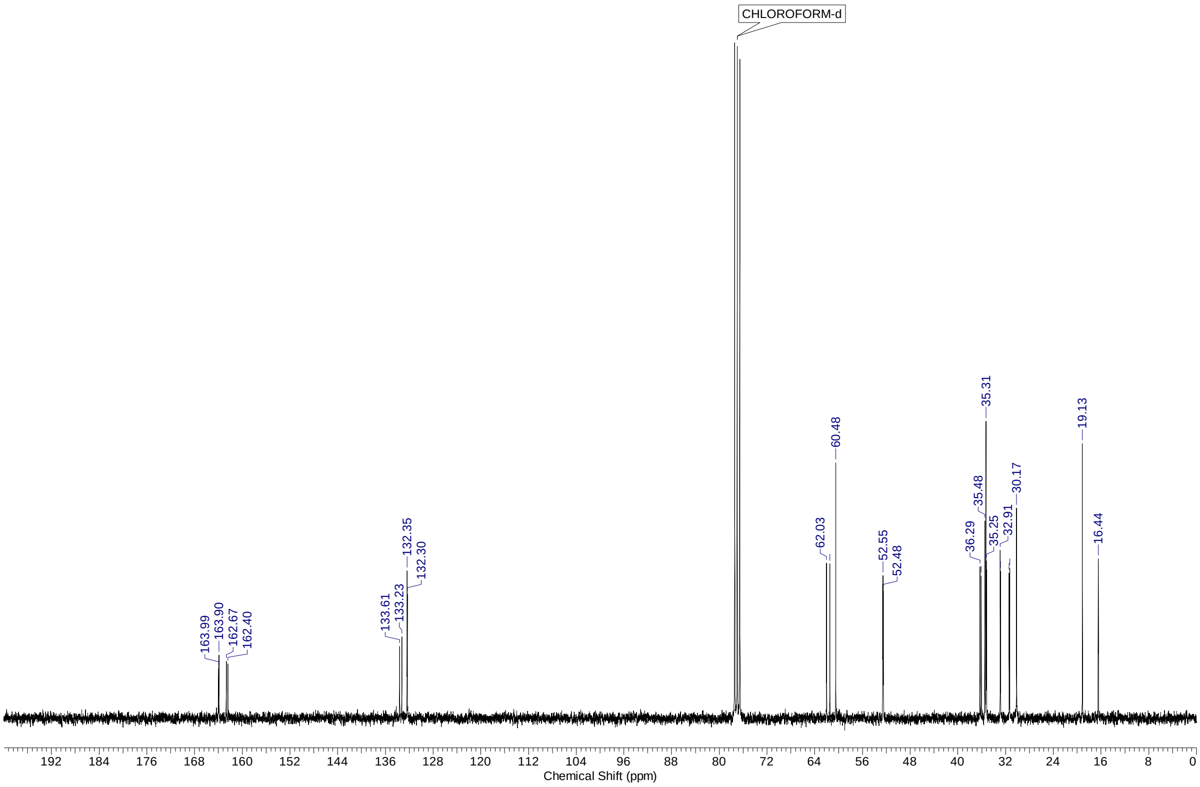

methyl 4-[(2*R*,5*S*)-5-isopropyl-3,6-dimethoxy-2,5-dihydropyrazin-2-yl]-2,2-dimethyl-pent-4-enoate (**3za**)

^{1}H NMR (300 MHz, $CDCl_3$)

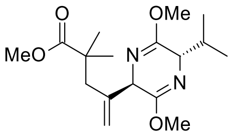

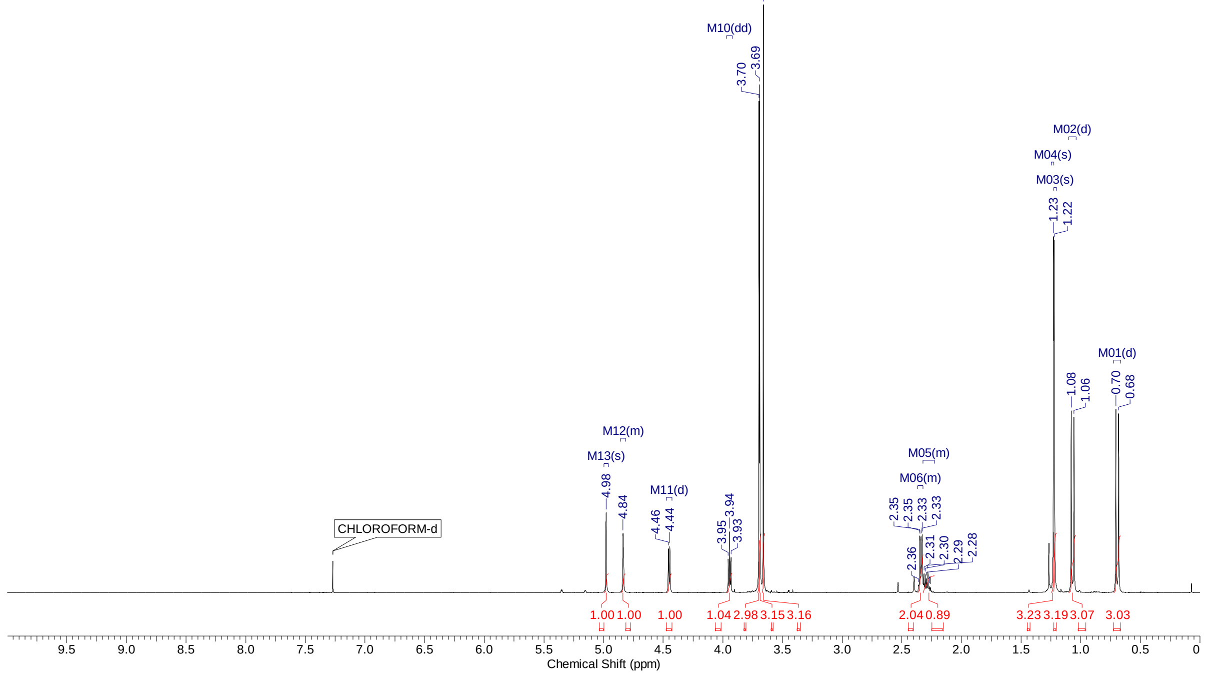

^{13}C NMR (75 MHz, $CDCl_3$)

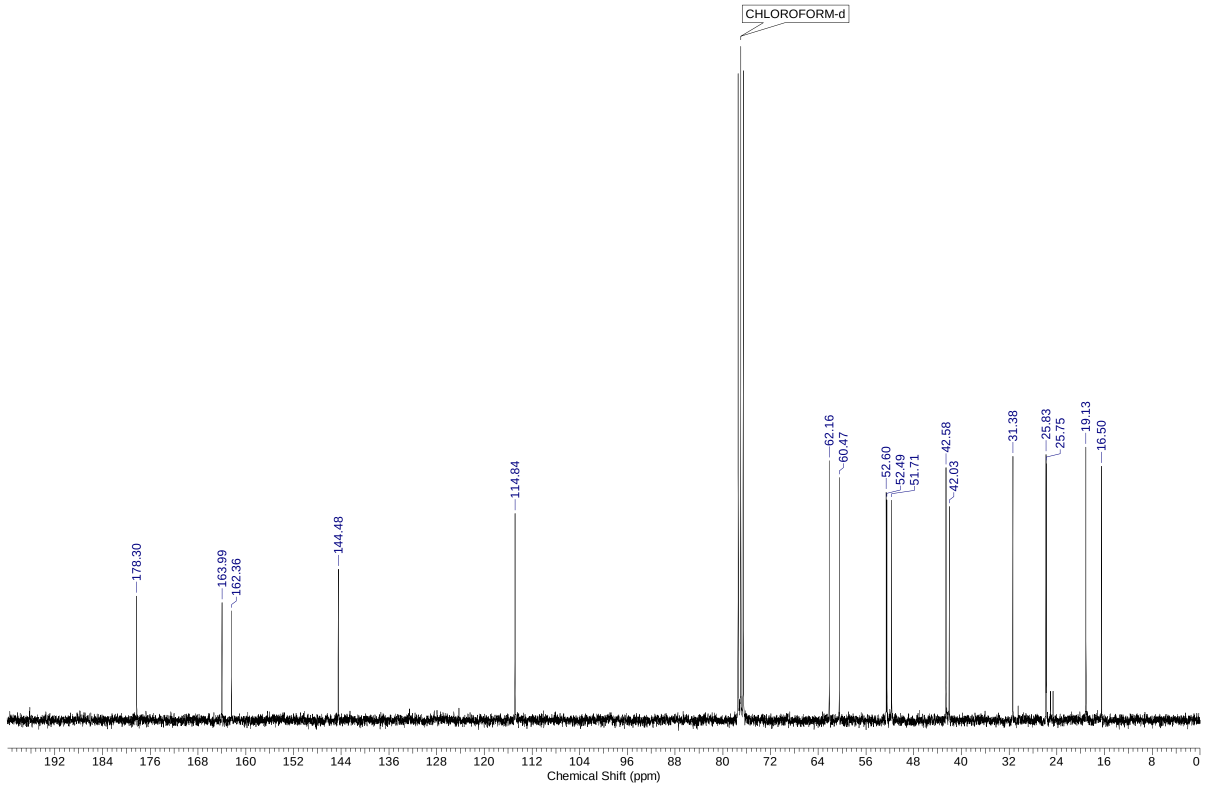

ethyl 2-[4-[(2*R*,5*S*)-5-isopropyl-3,6-dimethoxy-2,5-dihydropyrazin-2-yl]phenoxy]-2-methyl-propanoate (**3Aa**)

^{1}H NMR (300 MHz, $CDCl_3$)

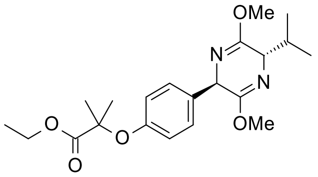

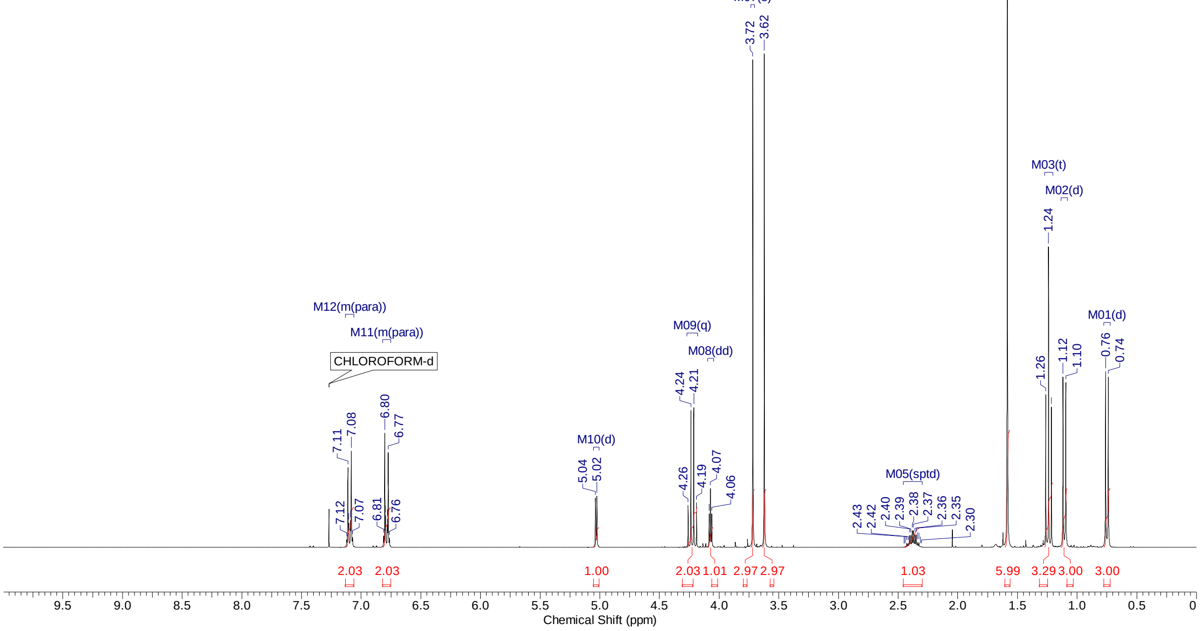

^{13}C NMR (75 MHz, $CDCl_3$)

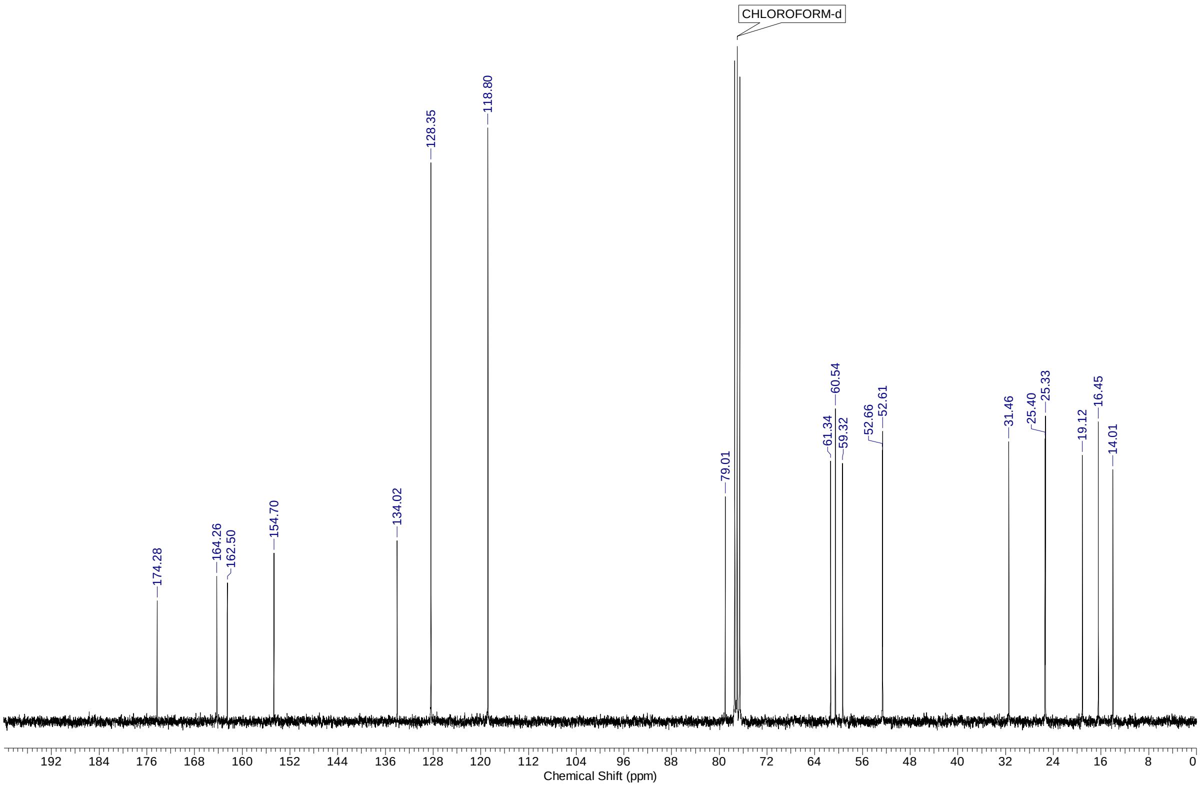

(2*S*,5*R*)-2-isopropyl-3,6-dimethoxy-5-[4-[phenyl-[2-(1-piperidyl)ethoxy]methyl]phenyl]-2,5-dihydropyrazine (**3Ba**)

^{1}H NMR (300 MHz, $CDCl_3$)

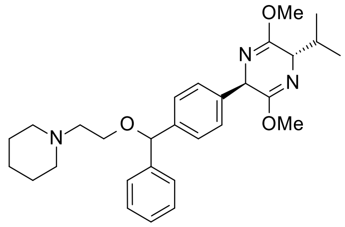

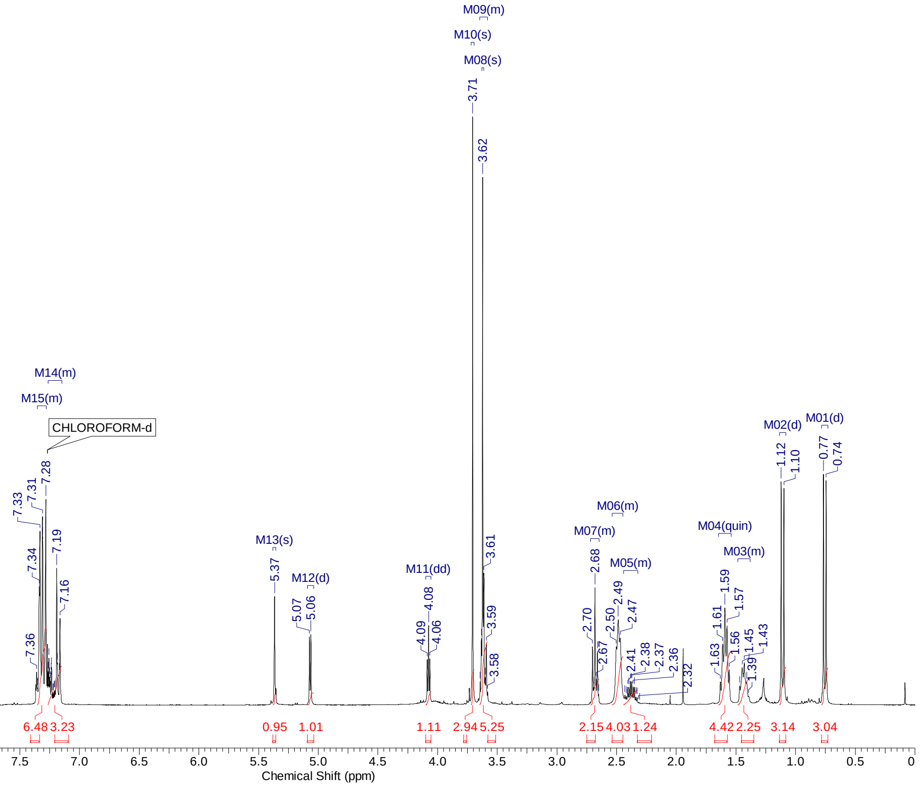

^{13}C NMR (75 MHz, $CDCl_3$)

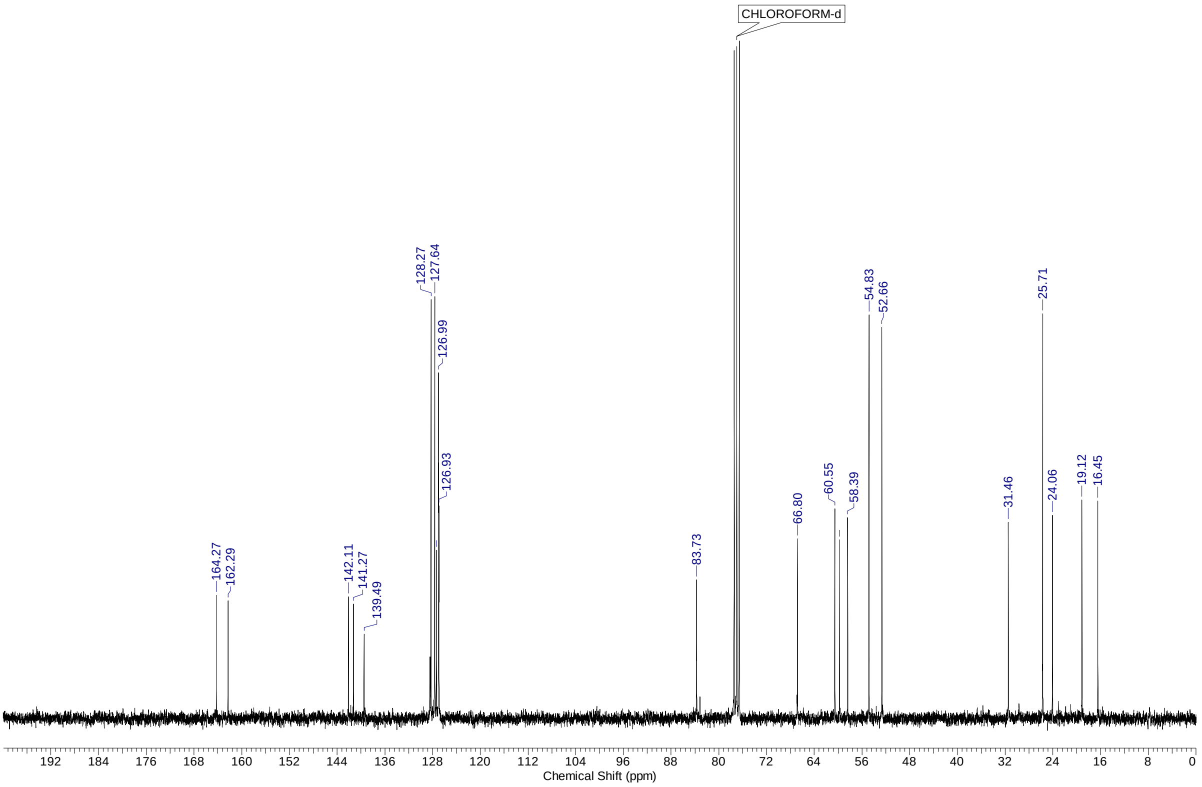

ethyl 4-[14-[(2R,5S)-5-isopropyl-3,6-dimethoxy-2,5-dihydropyrazin-2-yl]-4-azatricyclo[9.4.0.03,8]pentadeca-1(15),3,5,7,11,13-hexaen-2-ylidene]piperidine-1-carboxylate (**3Ca**)

^{1}H NMR (300 MHz, $CDCl_3$)

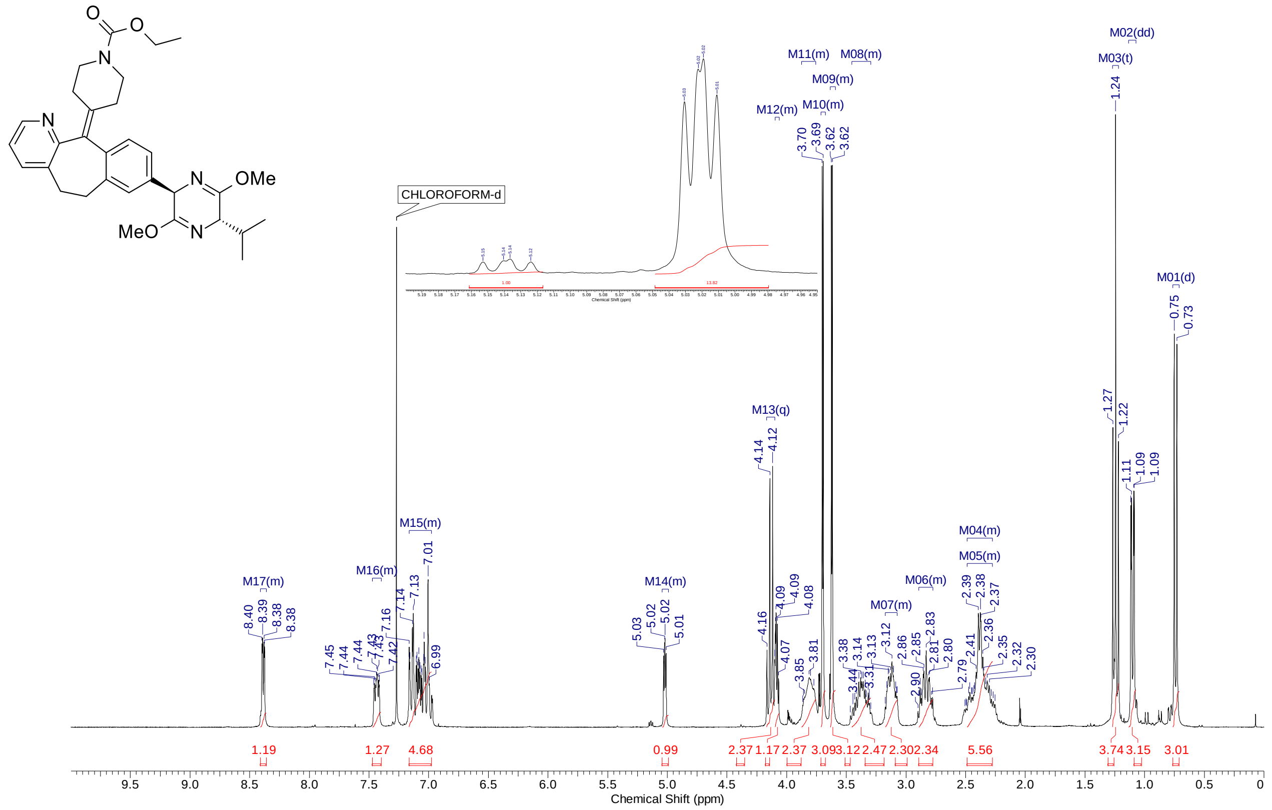

^{13}C NMR (75 MHz, $CDCl_3$)

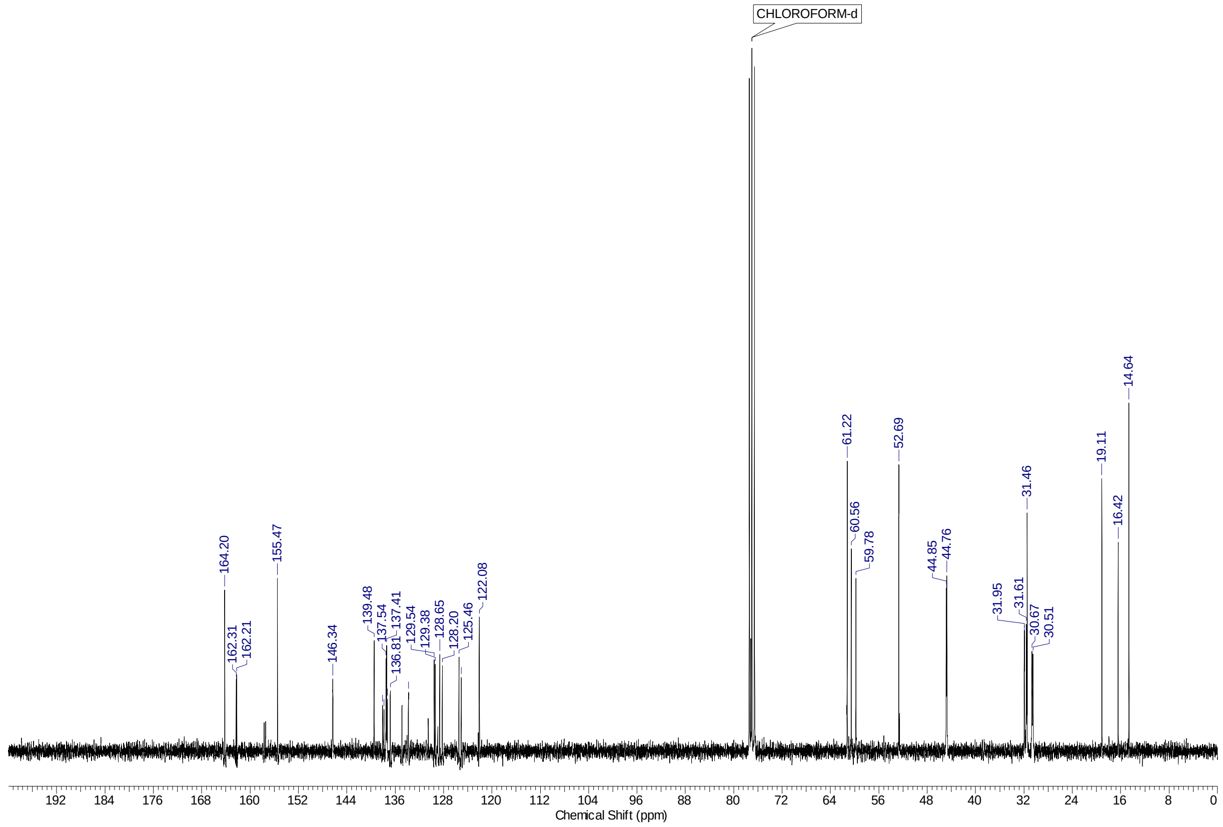

methyl (2*R*)-2-amino-2-(4-methoxyphenyl)acetate (**6a**) [CAS: 78307-39-8]

^{1}H NMR (300 MHz, $CDCl_3$)

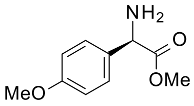

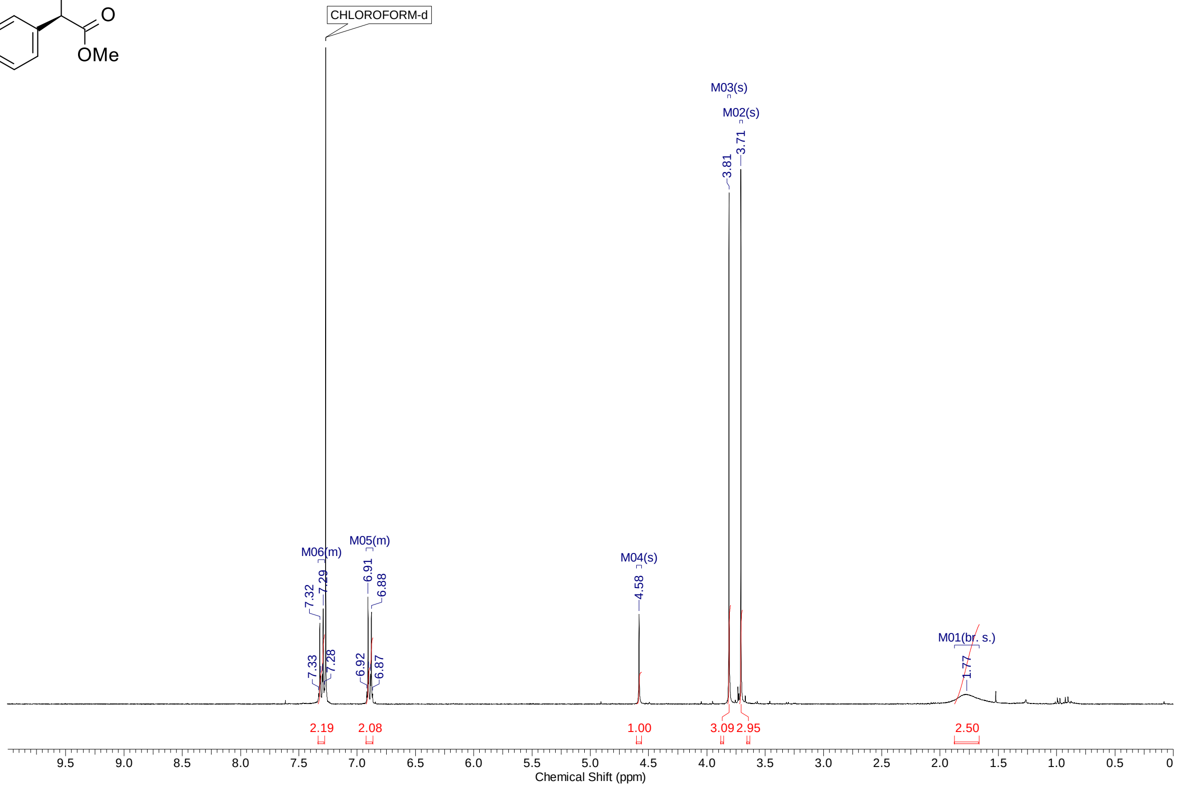

^{13}C NMR (75 MHz, $CDCl_3$)

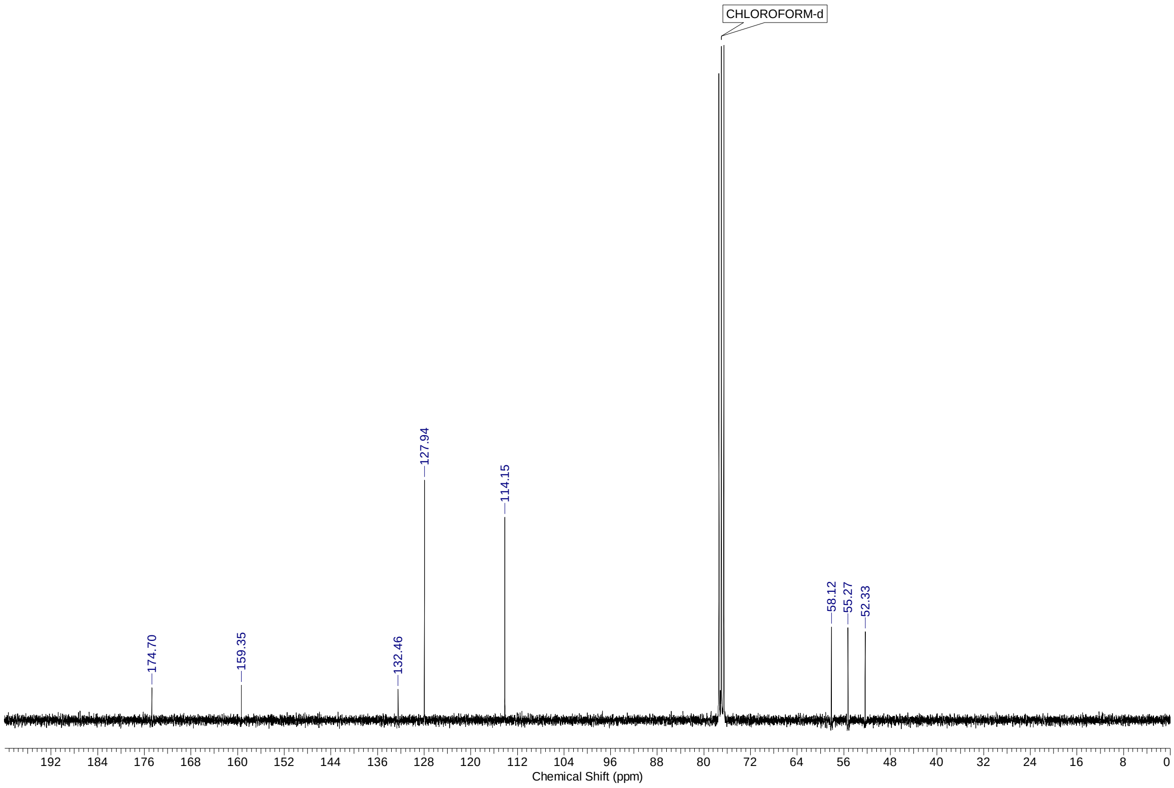

methyl (2*R*)-2-amino-2-phenyl-acetate (**6f**) [CAS: 24461-61-8]

^{1}H NMR (300 MHz, $CDCl_3$)

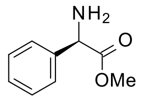

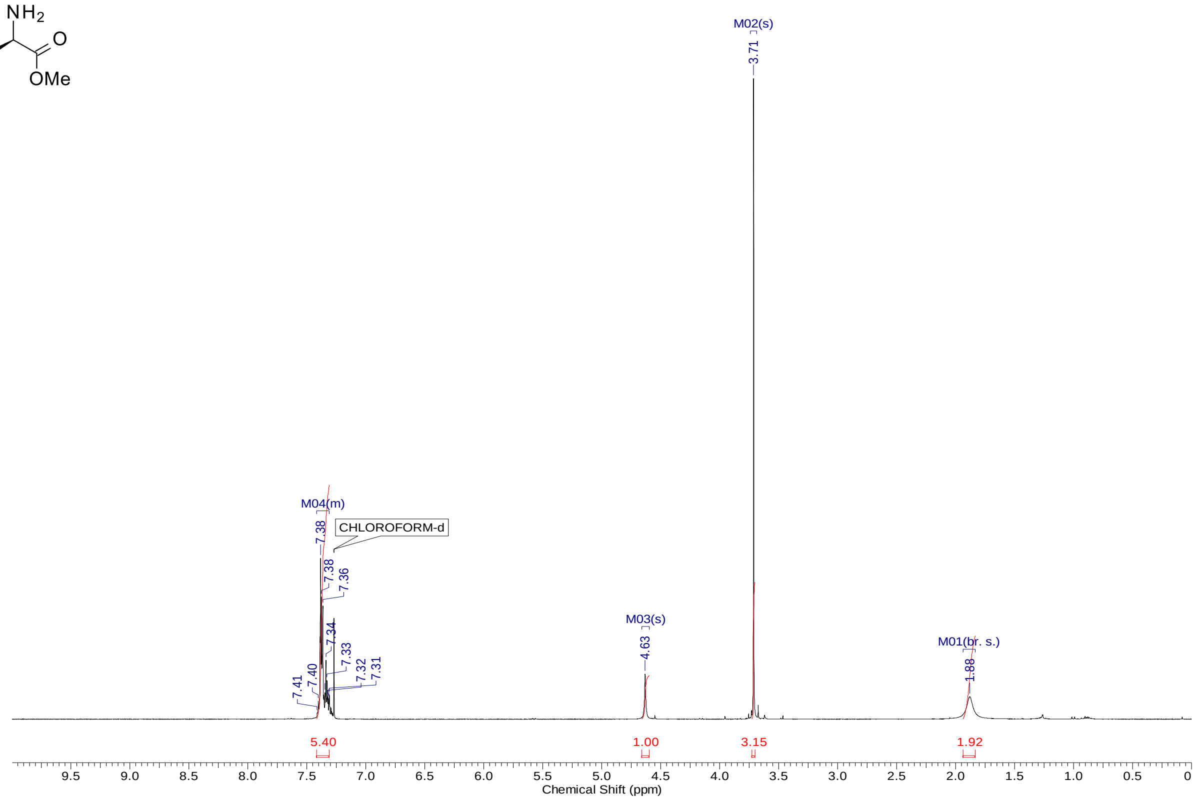

^{13}C NMR (75 MHz, $CDCl_3$)

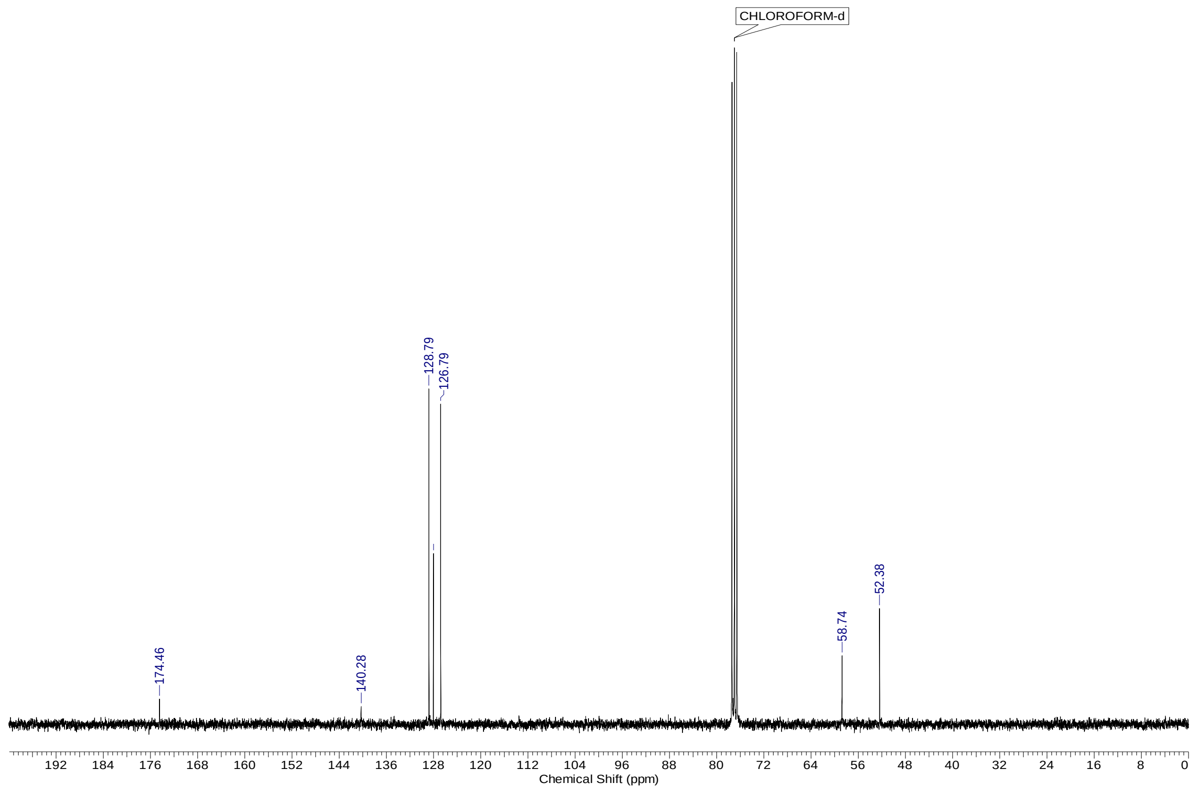

methyl (2R)-2-amino-2-(4-fluorophenyl)acetate (**6g**) [CAS: 170902-76-8]

^{1}H NMR (300 MHz, $CDCl_3$)

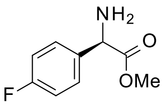

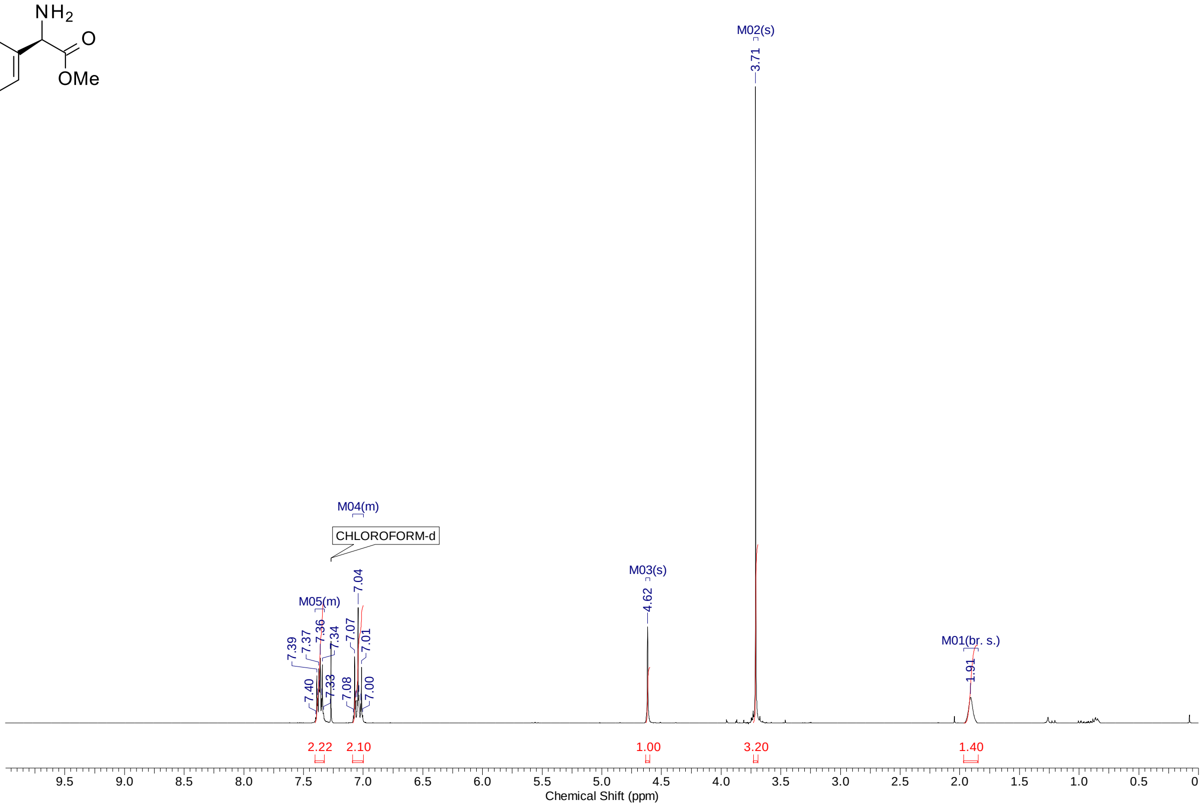

^{13}C NMR (75 MHz, $CDCl_3$)

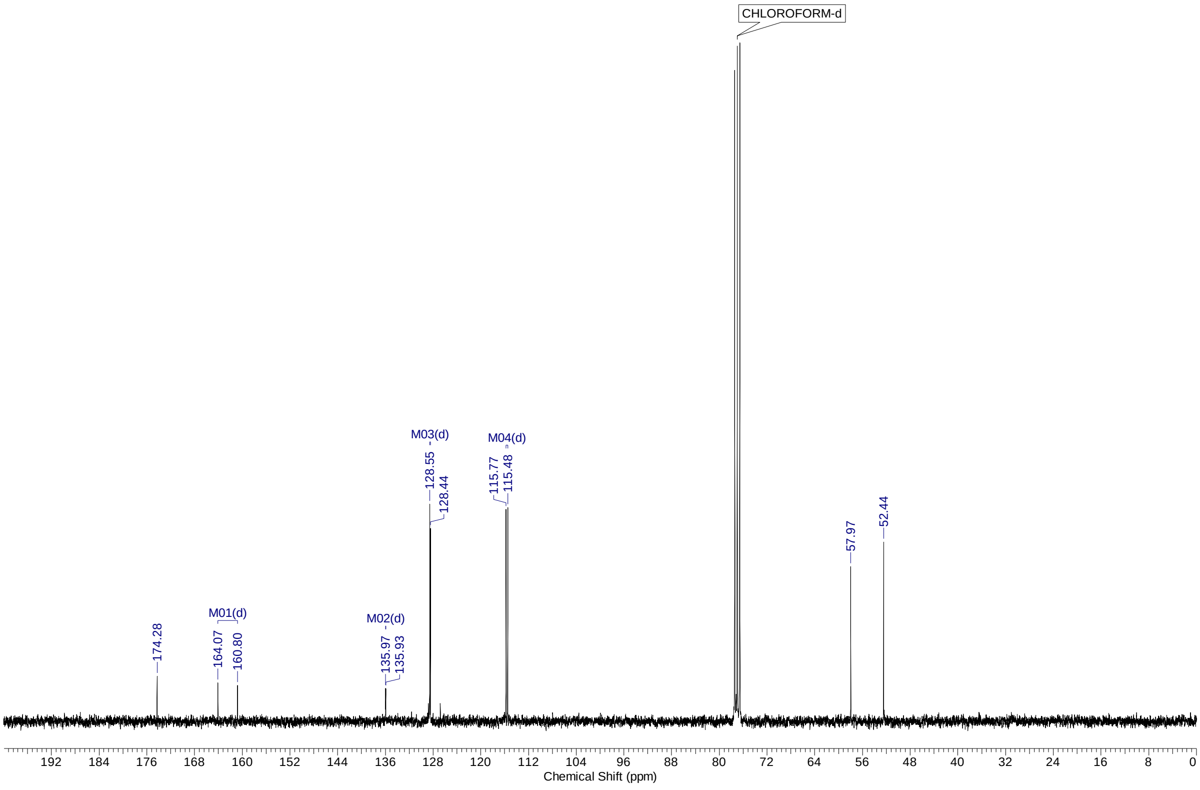

^{19}F NMR (41 MHz, $CDCl_3$)

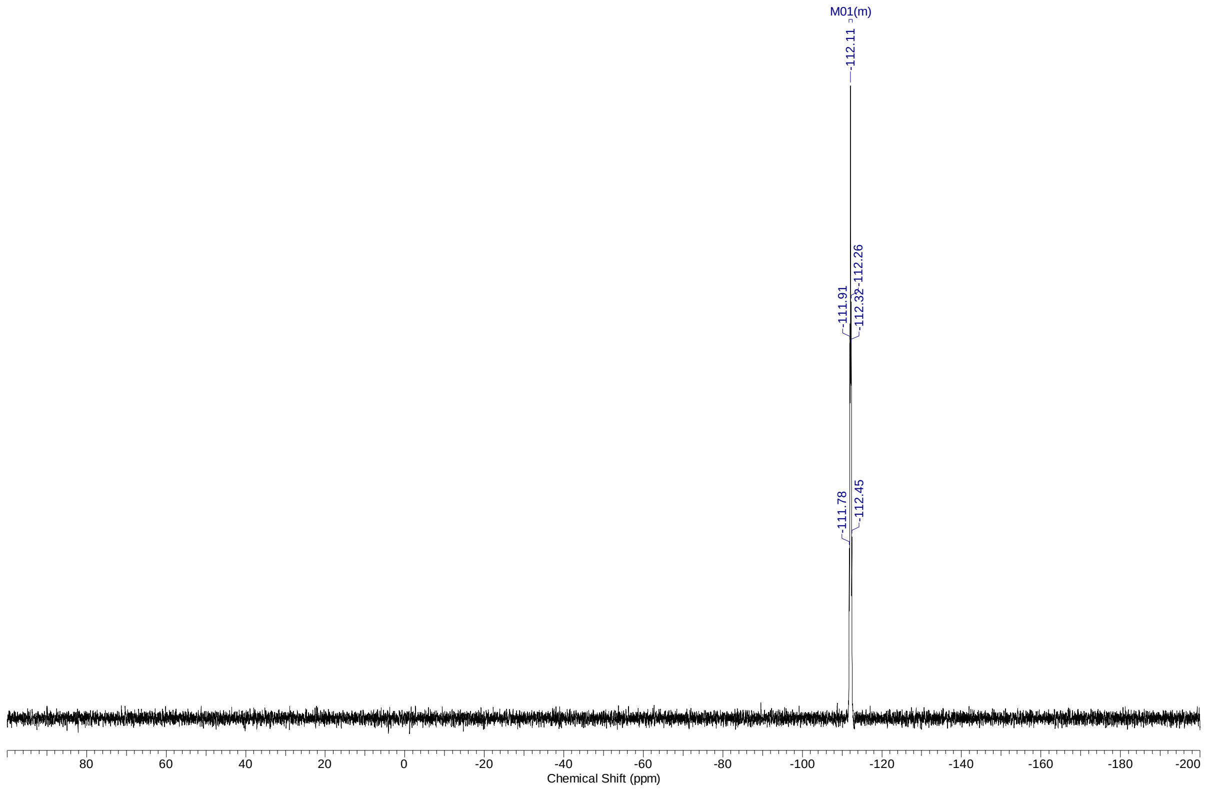

13. Computational and statistical studies

13.1 Computational Details

General considerations

Density functional theory (DFT)[17] calculations were performed with version 5.0.4 of the ORCA software package.[18] All calculations were carried out without symmetry restrictions. Conformational searches were performed at stationary points using two complementary approaches. 1) CREST[19] (GFN2-xTB[20] level) to broadly sample conformational space and 2) systematic manual perturbation of key torsions, followed by DFT refinement. While CREST was useful for generating diverse initial guesses, it frequently overestimated secondary metal-ligand interactions and occasionally missed relevant conformers in our Pd-ligand systems.

All the structures were optimized at the PBE0 level of theory with the Def2SVP basis sets for all atoms.[21] The Grimme's D3 dispersion correction with Becke-Johnson damping ("D3BJ") was also applied.[22] Solvent corrections were included by using the polarizable continuum model (PCM) for THF.[23] Frequency calculations were performed to verify that intermediates have no imaginary frequency, whereas the transition state structures have only one imaginary frequency. The reported Gibbs free energies were calculated at 298.15 K. Single-point energy calculations were carried out at PBE0-D3BJ, Def2TZVP + LANL2TZ(f)/SMD(THF) level of theory based on the energy-optimized structures.[24] The level of theory was selected based on precedent in related cross-coupling studies with the investigated systems. To confirm the robustness of our conclusions, we recomputed all relevant single-point energies at the PBE0-D3BJ/def2-QZVP level/SMD(THF)[21], which showed only minor energetic differences (-0.4 to +1.2 kcal(mol), confirming the reliability of the reported trends Structures were visualized using the program CYLview20. The final energy-optimized structures of all transition state structures, intermediates, starting reagents and products are given in the xyz file.

Evaluation of secondary metal ligand interaction

Second-order perturbation theory obtained by NBO analysis reveals a significantly stronger stabilizing interaction between the amine nitrogen lone pair and the Pd–C antibonding orbital in Int2 with ^{pip}adYPhos, with E(2) = 18.49 kcal/mol, compared to the Pd σ*(C–H) backdonation observed in the keYPhos Int2 with E(2) = 2.58 kcal(mol). This highlights the pronounced donor character of the amine and its key role in stabilizing the metal center, whereas the Pd–CH interaction represents a much weaker, secondary agostic interaction.

Evaluation of zincated bis-lactim ether

We considered several structures of the zincated bis-lactim ether differing in the number of THF coordinated to zinc and its orientation relative to the *iso*-propyl group (syn and anti). The different isomers are depicted in Figure S4, their energies are given in Table S11. While the bis-THF complex was found to be the most stable isolated species, one THF ligand must be displaced to allow substrate coordination to the palladium center. The liberation of THF is considered when calculating the energies in the energy profile in Fig. 2 in the manuscript relative to the bis-THF complex **2-ZnCl_N_2_THF**.

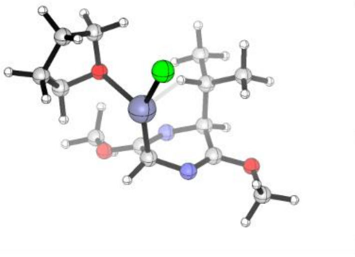

2-ZnCl_syn_1_THF

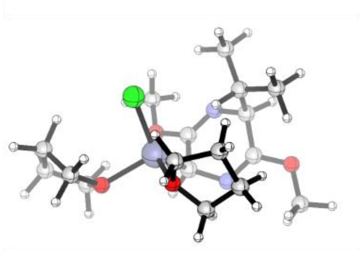

2-ZnCl_syn_2_THF

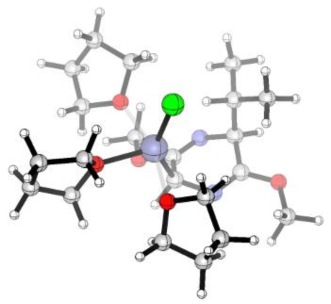

2-ZnCl_syn_3_THF

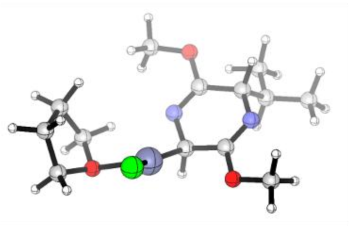

2-ZnCl_anti_1_THF

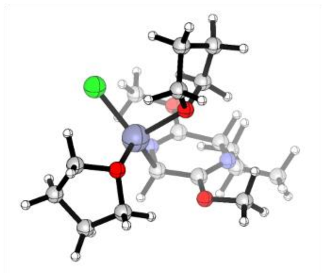

2-ZnCl_anti_2_THF

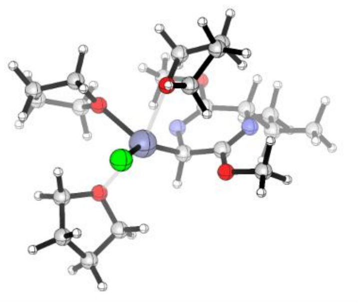

2-ZnCl_anti_3_THF

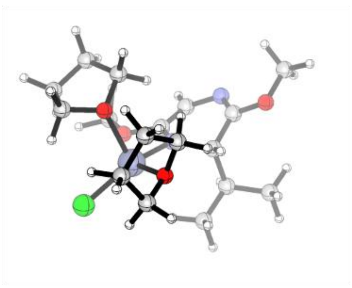

2-ZnCl_N_2_THF

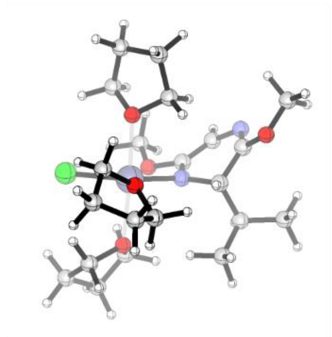

2-ZnCl_N_3_THF

Figure S4. Optimized structures of different metalated species of bis-lactim ether **2a**.

Table S11. Absolute energies (Hartree) of optimized geometries of metalated bis-lactim ethers. ΔG values relative to the most stable species are given in kcal/mol and are corrected for any additional THF molecules.

Species	E	G	ΔG [kcal/mol]
**2-ZnCl_syn_1_THF**	-3083.062797	-3082.766588	7.50
**2-ZnCl_syn_2_THF**	-3315.368012	-3314.961698	1.75
**2-ZnCl_syn_3_THF**	-3547.656227	-3547.142476	5.00
**2-ZnCl_anti_1_THF**	-3083.056648	-3082.760299	11.44
**2-ZnCl_anti_2_THF**	-3315.358366	-3314.953105	7.14
**2-ZnCl_anti_3_THF**	-3547.649907	-3547.136690	8.63
**2-ZnCl_N_2_THF**	-3315.370288	-3314.964489	0.00
**2-ZnCl_N_3_THF**	-3547.656731	-3547.143258	4.51

Optimized Structures and Energies of other Reactants and Products

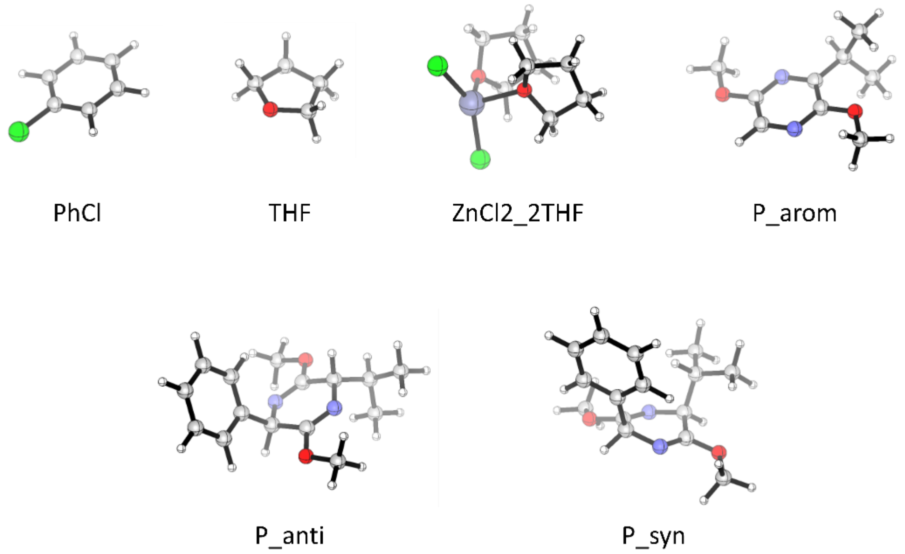

Figure S5. Optimized structures of reactants and products.

Table S12. Absolute energies (Hartree) of optimized geometries of other reactants and products.

Species	E	G
	**def2-TZVP+LANL2TZ(f)**	
**PhCl**	-691.5316	-691.472733
**THF**	-232.272647	-232.1859558
**ZnCl2_2THF**	-3315.370288	-3314.964489
**P_arom**	-610.8810919	-610.7003617
**P_anti**	-842.9535328	-842.6782546
**P_syn**	-842.9519873	-842.6752731
	**def2-QZVP**	
**PhCl**	-691.5585655	-691.499699
**THF**	-232.288255	-232.201564
**ZnCl2_2THF**	-3164.115484	-3163.92721
**P_arom**	-610.9174063	-610.736676
**P_anti**	-843.0036387	-842.728361
**P_syn**	-843.0021119	-842.725398

Optimized Structures and Energies for Transition States and Intermediates with keYPhos

The following two figures display the energy-optimized structures for the reaction pathways shown in Figure 3 with keYPhos as ligand. Table S13 gives the energies of all structures.

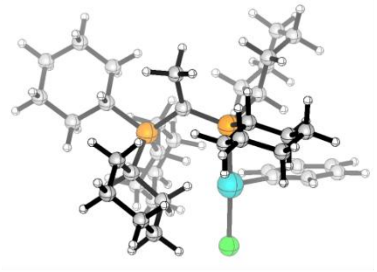

Int1

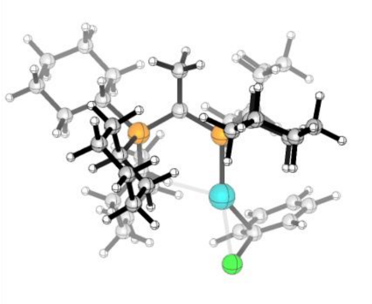

TS1

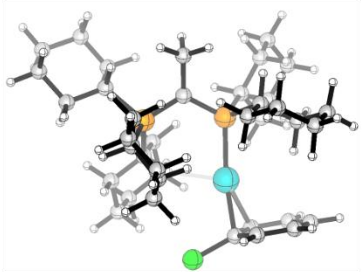

Int2

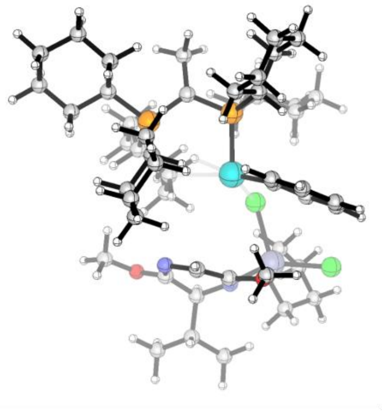

TS2a

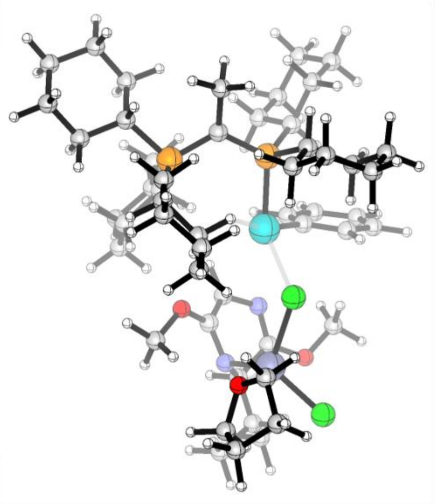

TS2b

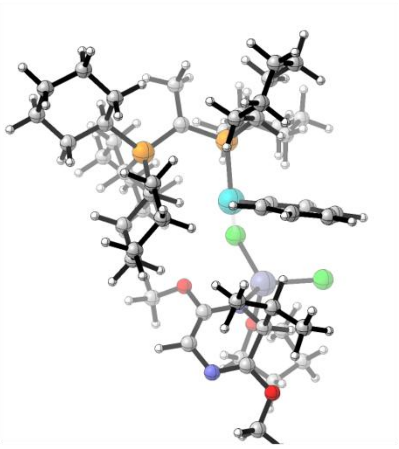

TS2c

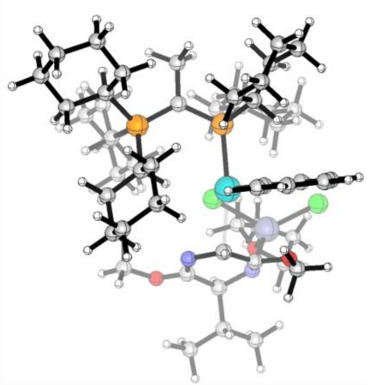

Int3a

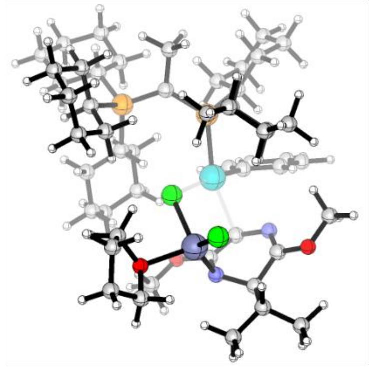

Int3b

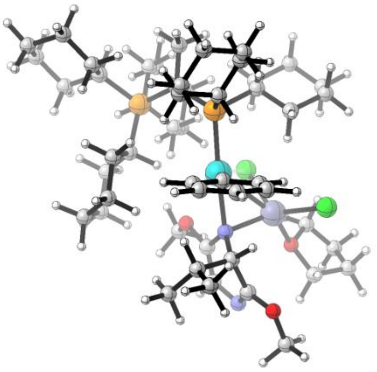

Int3c

Figure S6. Optimized structures of transition states and intermediates with keYPhos.

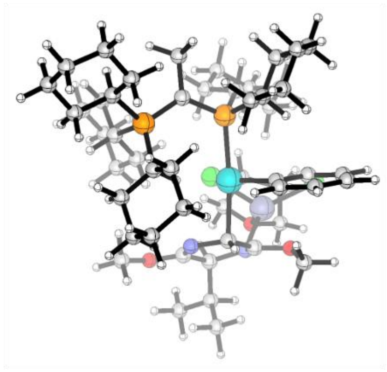

TS3a

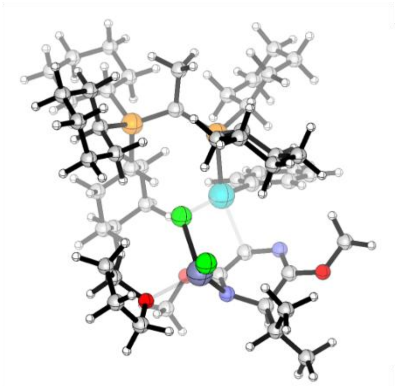

TS3b

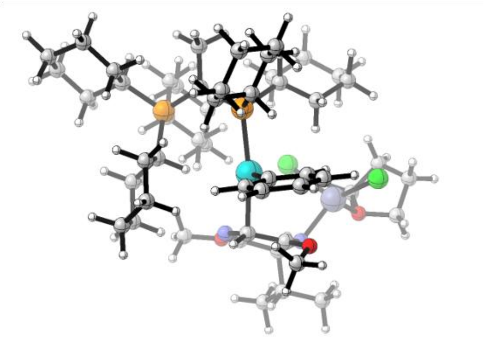

TS3c

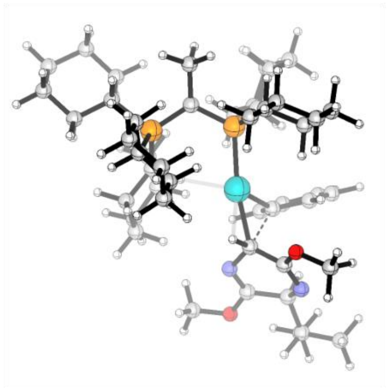

TS4a

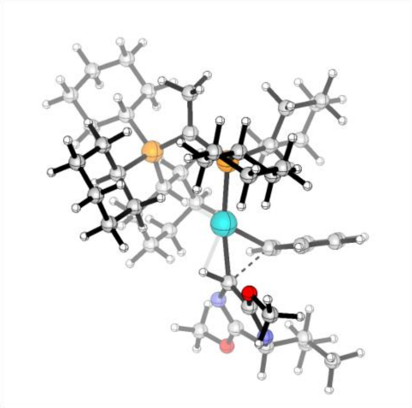

TS4b

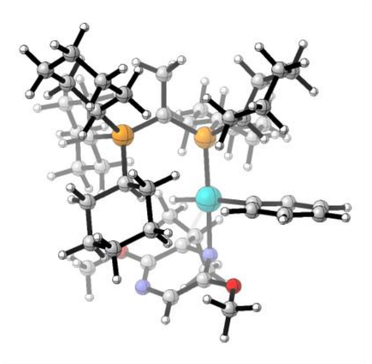

TS4c

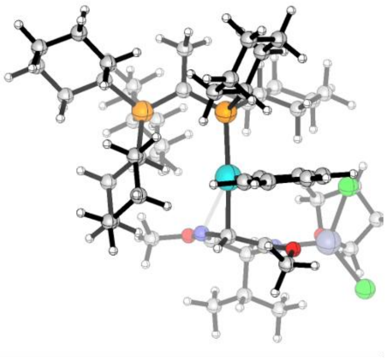

Int4a

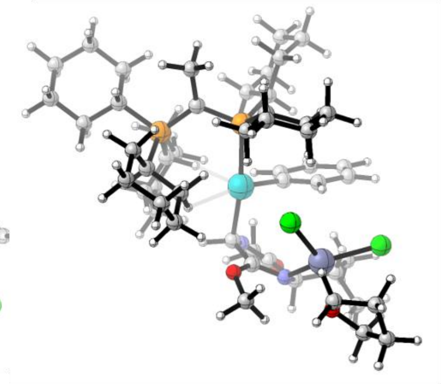

Int4b

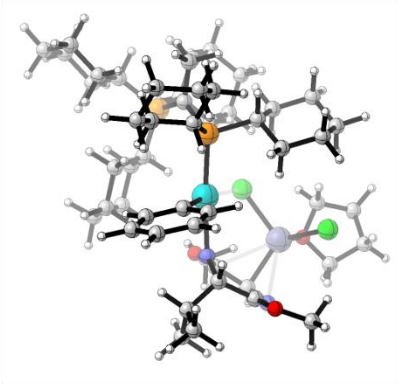

Int4c

Figure S7. Optimized structures of transition states and intermediates with keYPhos.

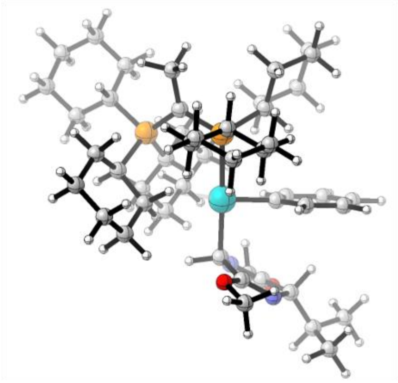

Int5a

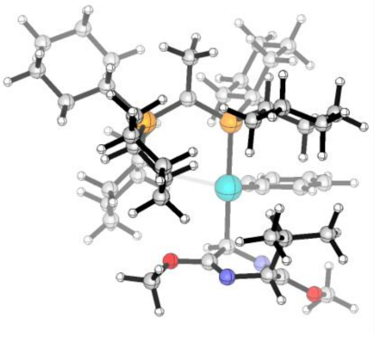

Int5b

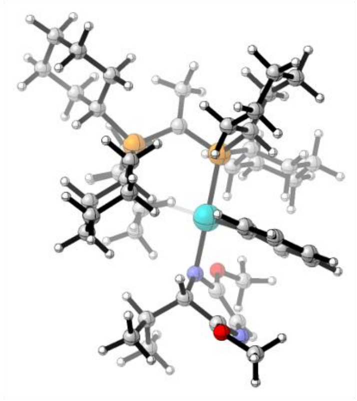

Int5c

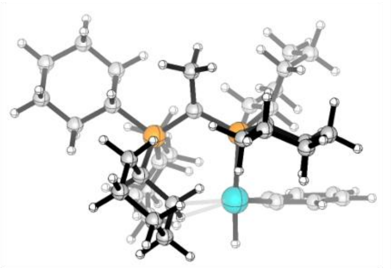

Int6c

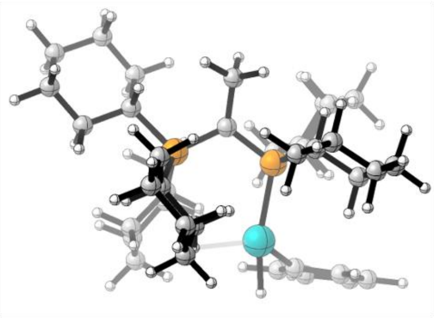

TS5c

Figure S8. Optimized structures of transition states and intermediates with keYPhos.

Table S13. Absolute energies (Hartree) of optimized geometries of intermediates with keYPhos.

Species	E	G
	**def2-TZVP+LANL2TZ(f)**	
**Int1**	-2755.39713	-2754.55386
**TS1**	-2755.38182	-2754.53741
**Int2**	-2755.4384	-2754.59092
**TS2a**	-5838.51686	-5837.34216
**Int3a**	-5838.5292	-5837.35387
**TS3a**	-5838.52891	-5837.35281
**Int4a**	-5838.53938	-5837.36466
**Int5a**	-2906.79462	-2905.72898
**TS4a**	-2906.76813	-2905.7046
**TS2b**	-5838.51605	-5837.34376
**Int3b**	-5838.5317	-5837.35692
**TS3b**	-5838.52108	-5837.3469
**Int4b**	-5838.52406	-5837.34825
**Int5b**	-2906.79545	-2905.72891
**TS4b**	-2906.77353	-2905.70936
**TS2c**	-5838.52308	-5837.34974
**Int3c**	-5838.53393	-5837.35802
**TS3c**	-5838.5264	-5837.34929
**Int4c**	-5838.53043	-5837.3549
**Int5c**	-2906.78714	-2905.7218
**TS4c**	-2906.76635	-2905.70565
**Int6**	-2295.89097	-2295.03697
**TS5c**	-2295.88323	-2295.03283
	**Def2-QZVP**	
**Int1**	-2755.532782	-2754.68951
**TS1**	-2755.517386	-2754.67297
**Int2**	-2755.5759	-2754.72842
**TS2a**	-5838.761863	-5837.58716
**Int3a**	-5838.773467	-5837.59814
**TS3a**	-5838.773035	-5837.59693
**Int4a**	-5838.784217	-5837.60949
**Int5a**	-2906.954318	-2905.88868
**TS4a**	-2906.927045	-2905.86351

**TS2b**	-5838.760726	-5837.58843
**Int3b**	-5838.776039	-5837.60127
**TS3b**	-5838.765503	-5837.59132
**Int4b**	-5838.76804	-5837.59223
**Int5b**	-2906.955061	-2905.88852
**TS4b**	-2906.932497	-2905.86832
**TS2c**	-5838.767902	-5837.59456
**Int3c**	-5838.778036	-5837.60212
**TS3c**	-5838.770487	-5837.59338
**Int4c**	-5838.77465	-5837.59912
**Int5c**	-2906.94734	-2905.882
**TS4c**	-2906.925694	-2905.86426
**Int6**	-2296.014929	-2295.16093
**TS5c**	-2296.006807	-2295.15641

Optimized Structures and Energies for Transition States and Intermediates with ^{Pip}adYPhos

The following two figures display the energy-optimized structures for the reaction pathways shown in Figure 3 with ^{Pip}adYPhos as ligand. Table S14 gives the energies of all structures.

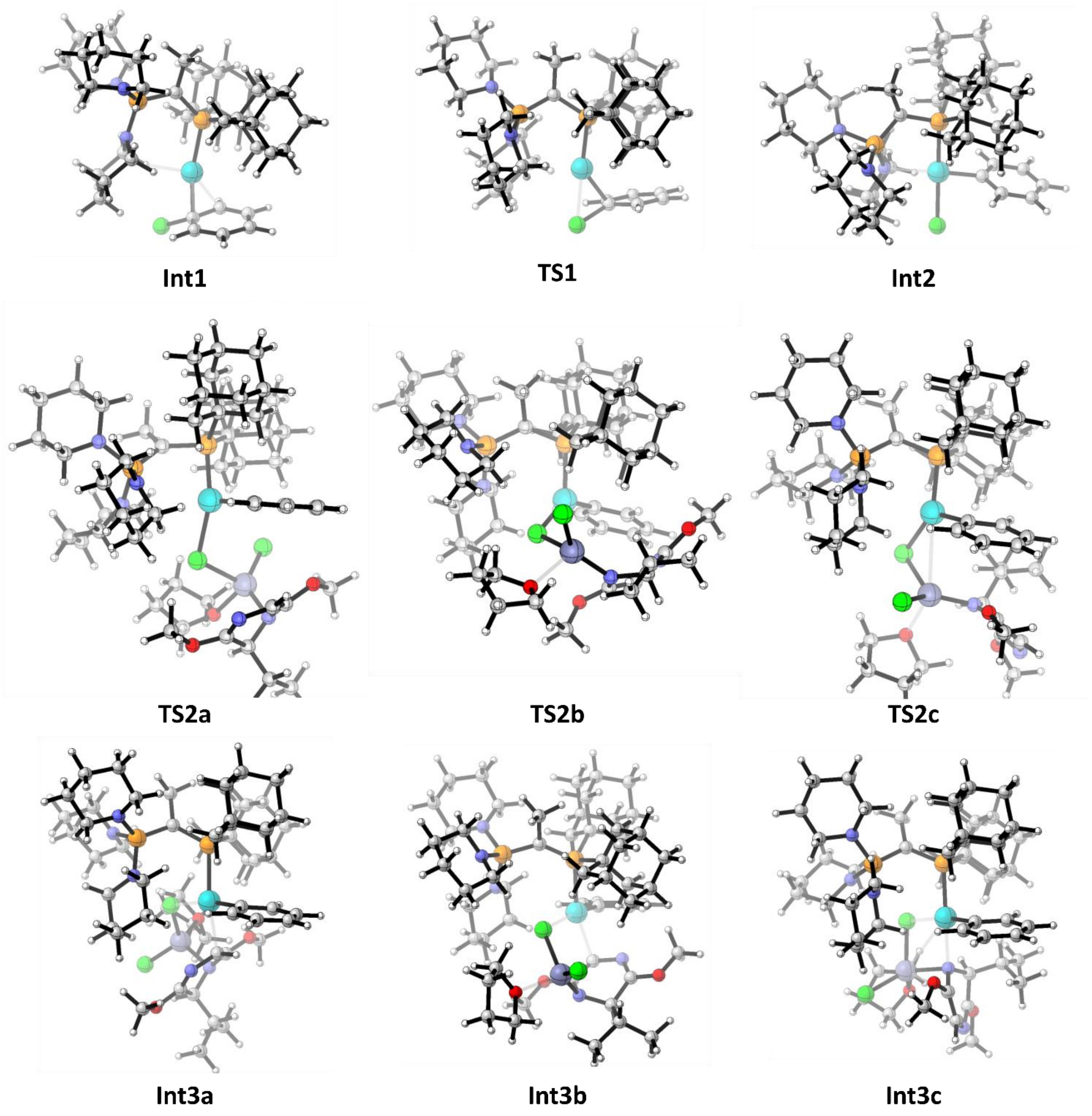

Figure S9. Optimized structures of transition states and intermediates with ^{pip}adYPhos.

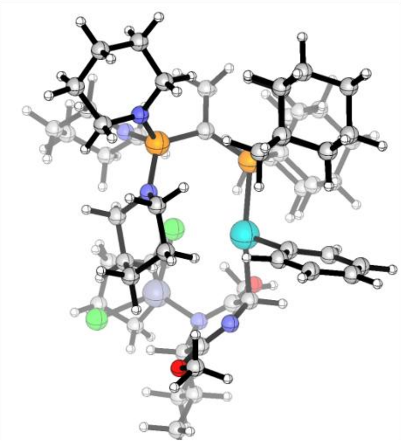

TS3a

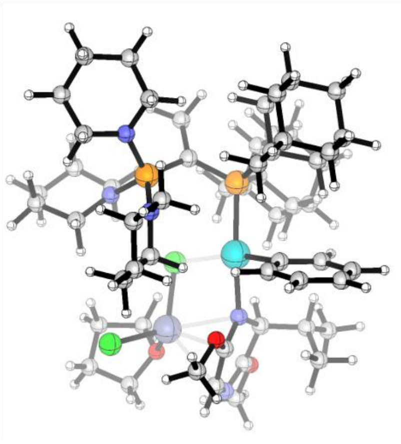

TS3c

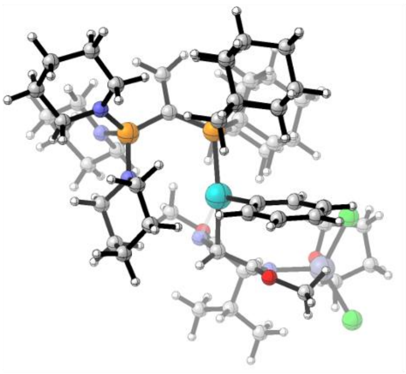

Int4a

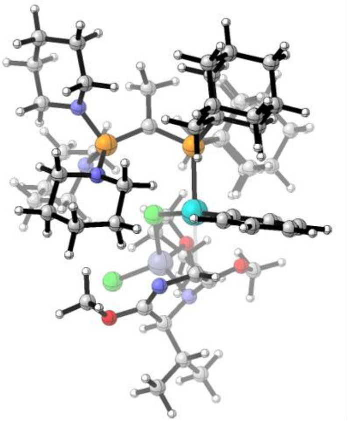

Int4c

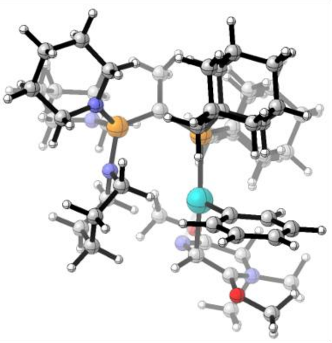

Int5a

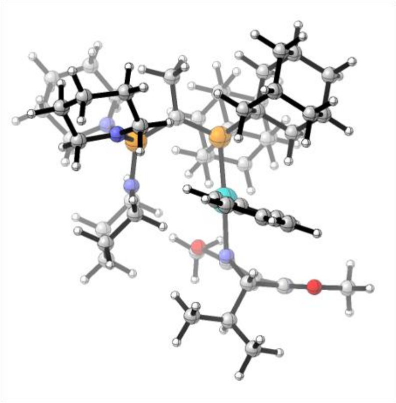

Int5c

Figure S10. Optimized structures of transition states and intermediates with ^{pip}adYPhos.

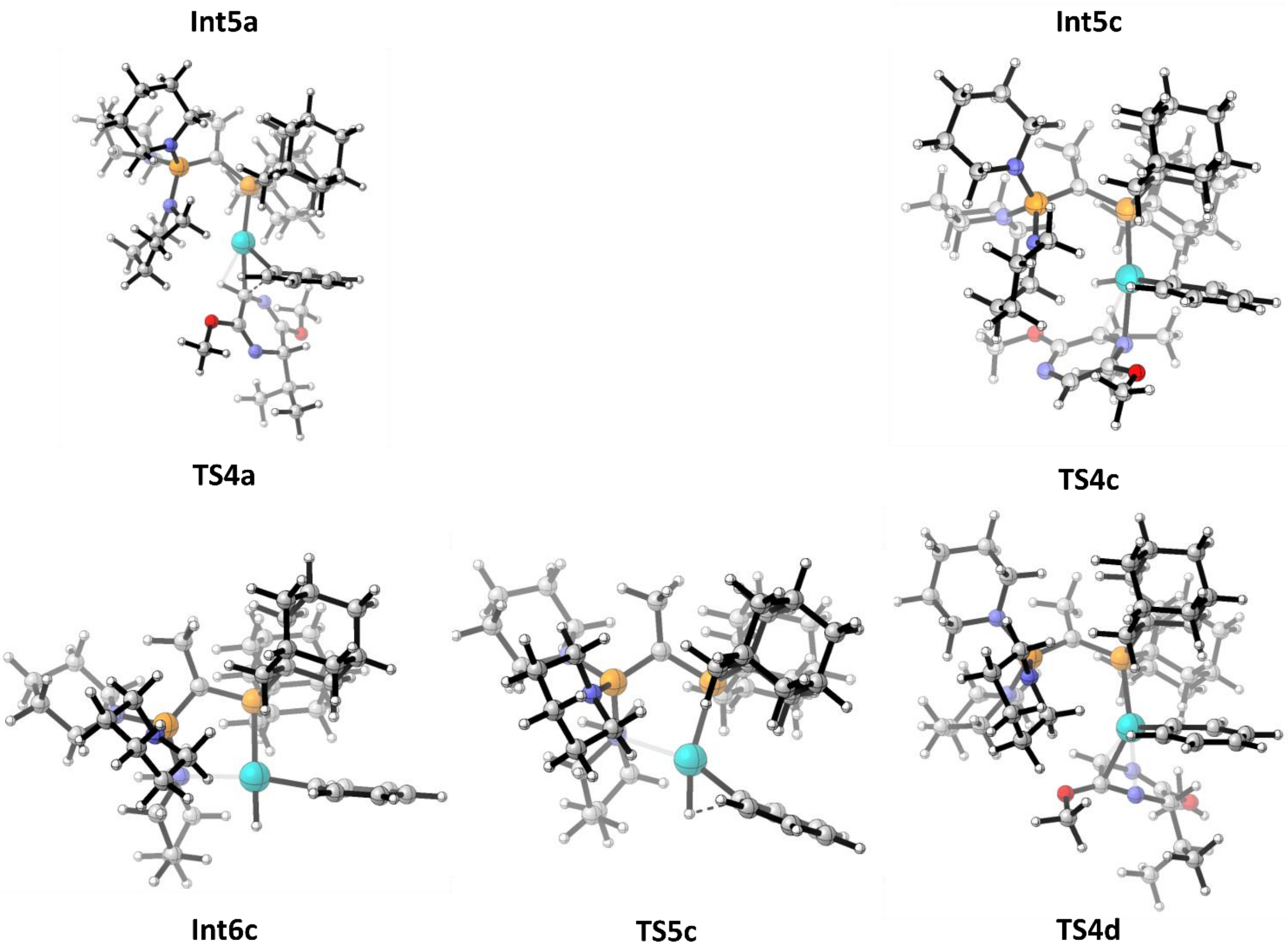

Figure S11. Optimized structures of transition states and intermediates with ^{pip}adYPhos.

Table S14. Absolute energies (Hartree) of optimized geometries of intermediates with [pip]adYPhos.

Species	E	G
	**def2-TZVP+LANL2TZ(f)**	
**Int1**	-3112.97868	-3112.02637
**TS1**	-3112.95611	-3112.00266
**Int2**	-3113.01477	-3112.05719
**TS2a**	-6196.09681	-6194.81836
**Int3a**	-6196.10135	-6194.81979
**TS3a**	-6196.0983	-6194.81593
**Int4a**	-6196.11987	-6194.83657
**Int5a**	-3264.3796	-3263.20642
**TS4a**	-3264.35344	-3263.18199
**TS2b**	-6196.08232	-6194.80273
**Int3b**	-6196.09514	-6194.81279
**Int3c**	-6196.10691	-6194.82242
**TS3c**	-6196.08704	-6194.80421
**Int4c**	-6196.10391	-6194.82027
**Int5c**	-3264.35519	-3263.18162
**TS4c**	-3264.3343	-3263.16418
**Int6c**	-2653.47793	-2652.5145
**TS5**	-2653.46985	-2652.50975
	**Def2-QZVP**	
**Int1**	-3113.13356	-3112.18125
**TS1**	-3113.11087	-3112.15743
**Int2**	-3113.17134	-3112.21376
**TS2a**	-6196.36076	-6195.08231
**Int3a**	-6196.36514	-6195.08358
**TS3a**	-6196.36136	-6195.07898
**Int4a**	-6196.38368	-6195.10038
**Int5a**	-3264.55818	-3263.385
**TS4a**	-3264.5313	-3263.35986
**TS2b**	-6196.34598	-6195.06639
**Int3b**	-6196.35831	-6195.07596
**TS2c**	-6196.36283	-6195.08247
**Int3c**	-6196.37038	-6195.0859
**TS3c**	-6196.35053	-6195.06771

**Int4c**	-6196.36205	-6195.07842
**Int5c**	-3264.53466	-3263.36109
**TS4c**	-3264.5138	-3263.34368
**Int6c**	-2653.62101	-2652.65758
**TS5**	-2653.61268	-2652.65258

Comparison between basis set and ecp combinations

Table S15. Relative energies of transition states and intermediates for keYPhos and pipadYPhos on using single point energies from two different basis set and ECP combinations and their relative difference. All energies given in kcal/mol.

Species	ΔG (def2-TZVP+LANL2TZ(f))	ΔG (def2-QZVP)	difference
	**keYPhos**		
**Int1**	23.3	24.4	1.2
**TS1**	33.6	34.8	1.2
**Int2**	0.0	0.0	0.0
**TS2a**	17.1	17.8	0.6
**Int3a**	9.8	10.9	1.1
**TS3a**	10.4	11.6	1.2
**Int4a**	3.0	3.8	0.7
**Int5a**	0.8	0.7	0.0
**TS4a**	16.1	16.5	0.5
**TS2b**	16.1	17.0	0.8
**Int3b**	7.9	8.9	1.1
**TS3b**	14.2	15.2	1.0
**Int4b**	13.3	14.6	1.3
**Int5b**	0.8	0.8	0.0
**TS4b**	13.1	13.5	0.4
**TS2c**	12.4	13.1	0.8
**Int3c**	7.2	8.4	1.2
**TS3c**	12.7	13.9	1.2
**Int4c**	9.1	10.3	1.1
**Int5c**	5.3	4.9	-0.4
**TS4c**	16.5	16.0	-0.4
**Int6**	-4.5	-4.9	-0.4
**TS5c**	-1.9	-2.0	-0.2
	**pipadYPhos**		
**Int1**	23.3	24.4	1.2
**TS1**	33.6	34.8	1.2
**Int2**	0.0	0.0	0.0
**TS2a**	17.1	17.8	0.6
**Int3a**	9.8	10.9	1.1
**TS3a**	10.4	11.6	1.2
**Int4a**	3.0	3.8	0.7
**Int5a**	0.8	0.7	0.0

**TS4a**	16.1	16.5	0.5
**TS2b**	16.1	17.0	0.8
**Int3b**	7.9	8.9	1.1
**TS3b**	14.2	15.2	1.0
**Int4b**	13.3	14.6	1.3
**Int5b**	0.8	0.8	0.0
**TS4b**	13.1	13.5	0.4
**TS2c**	12.4	13.1	0.8
**Int3c**	7.2	8.4	1.2
**TS3c**	12.7	13.9	1.2
**Int4c**	9.1	10.3	1.1
**Int5c**	5.3	4.9	-0.4
**TS4c**	16.5	16.0	-0.4
**Int6**	-4.5	-4.9	-0.4
**TS5c**	-1.9	-2.0	0.8

Isomerisation

An interconnection between the anti-transmetalation and N-metalation pathways was found with ^{Pip}adYPhos. A transition state between **Int5c** and **Int5a** could be localized with an energy barrier of 5.7 kcal/mol. This interconversion would still require the reaction to first proceed via the energetically less favourable N-transmetalation.

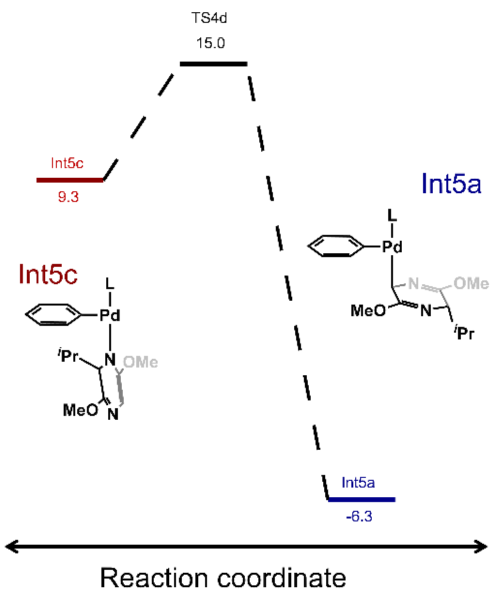

Figure S12: Energy profile (in kcal/mol) for the interconvertion in the mechanism with ^{Pip}adYPhos

13.2 Computational workflow for the parametrization

The full parameterization workflow used in this work was inspired by the *kraken* phosphine library reported by Gensch, Sigman, Aspuru-Guzik and coworkers, but has been partially adapted for this project.[25] Since the selectivity determining steps occur after the oxidative addition, we chose to parameterize all phosphine ligands in a *cis*-[L_1Pd(Ph)Cl] oxidative addition complex (Figure S13). This complex is also closer to the selectivity determining transmetalation step, making it a better starting structure for evaluating differences in the electronic and steric properties induced by the free ligands. All properties were extracted for the full complex.

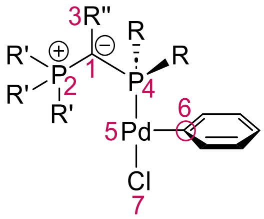

Figure S13: Example *cis*-[L_1Pd(Ph)Cl] oxidative addition complex with a generic YPhos ligand and atom numbering.

Input geometries were created manually and preoptimized at the GFN2-xTB[ALPB(THF)] level of theory using the xTB software version 6.4.1.[26-28] To obtain a full coverage of the chemical conformer space, several very different conformers were created as input for a subsequent conformer ensemble generation using CREST version 2.11 at the same level of theory as the preoptimization.[29] The relative energy threshold was set to 12.0 kcal/mol and the temperature to 353.15 K. Additionally, constraints were added to preserve the planar geometry of the ylidic moiety in the YPhos ligands and the quadratic planar *cis*-geometry of the full complex. The dihedral angle between the ylidic carbon **1**, the phosphonium moiety **2**, the backbone substituent **3** and the phosphine moiety **4** was set to zero degrees (Figure S13). The angle between the phosphine moiety **4**, Pd **5** and Cl **7** was set to 180 degrees, while the angles between phosphine **4**, Pd **5** and phenyl-moiety **6** as well as phosphine-moiety **6**, Pd **5** and Cl **7** were fixed to 90 degrees, respectively. The force constant was set to 0.15 E_h/Bohr^2. For every ligand, multiple CREST runs using different input geometries were performed and the resulting conformer ensembles were concatenated. To reduce the number of conformers passed to the DFT workflow while maintaining structural diversity, the conformer ensemble was pruned using an RMSD cutoff. Initially, all conformers were aligned to the lowest energy conformer, and RMSD values between all structures were calculated. Higher energy structures within an RMSD threshold of 0.2 Å to another conformer were removed. If more than 150 conformers remained, this threshold was incrementally increased by 0.2 Å. This process was repeated until the conformer count reached 150. The pruned conformer ensemble is refined using Part0 of the CENSO software package version 1.2.0.[30] Part0 calculates the free energy with the electronic energy at the B97-D3/def2-SV(P) level of theory with thermostatistical contributions at the GFN2-xTB[ALPB(THF)] level, using the geometries obtained by CREST.[31-33] These improved relative energies of the conformer ensemble were used to exclude all conformers above 6.0 kcal/mol.

Next, a conformer selection for the following DFT-level geometry optimization was performed. To this end, a set of steric properties was calculated for every remaining conformer and the conformers with the highest or lowest value of these properties were selected. These properties include: Sterimol B_1 & B_5, buried volume, far buried volume, max delta between two quadrants of buried volume, most occupied octant of buried volume, pyramidalization of phosphor-donor, (Sterimol B_5 of backbone), (P–C–P backbone angle), (buried volume of the phosphonium-moiety). Entries in brackets are for YPhos ligands only. A full definition of every property can be found below, in the properties section. Additionally, conformers relevant to the Boltzmann distribution were selected. Starting from the lowest energy conformer and progressing to higher energy conformers within a 4 kcal/mol window, the RMSD between the conformer and all previously selected structures was calculated. If a threshold of 2.5 Å was surpassed, the conformer was added to the selection and the threshold was multiplied by 1.15 times. If the threshold was not met, the conformer was skipped. If fewer than 8 conformers were selected, the process was repeated with a smaller initial threshold of 2 Å or 1 Å. Finally, if more than 15 structures were chosen for the Boltzmann distribution, the RMSD between all selected structures was calculated, and the structure with the lowest average deviation was removed. This was repeated until 15 conformers remained.

The selected conformers are forwarded to a geometry optimization and subsequent frequency calculation in the gas phase at 353.15 K at the r^{2}SCAN-3c level of theory using the ORCA software version 5.0.4.[34-37] Ground states were verified by the absence of any imaginary frequencies. All following single points were performed at the PBE0-D3BJ/def2-TZVP)/SDD(Pd) and PBE0-D3BJ/def2-TZVP/SMD(THF)/SDD(Pd) levels of theory.[32,38-40] NBO analysis was performed using the software version 7.0.[41] Single points were performed for the neutral molecule in the gas and solvation phase as well as the radical anions (ra) and cations (rc) in the gas phase.

All DFT-level optimized geometries with the respective free energies at PBE0-D3BJ/def2-TZVP/SDD(Pd)//r^{2}SCAN-3c and PBE0-D3BJ/def2-TZVP/SMD(THF)/SDD(Pd)//r^{2}SCAN-3c level of theory are given in an additional file.

Properties

Properties describe the individual conformer, while descriptors are condensed from the conformers properties and describe the ligand itself. Properties listed with brackets are available for YPhos ligands only.

Structural properties

The *morfeus* software package for python by Kjell Jorner was used for most of the properties obtained from the geometry itself.[42] These geometries are marked with an asterisk. If not stated otherwise, default settings by *morfeus* are used for every property.

Sterimol *B₁* & *B₅:**[43] The sterimol vectors B_1 and B_5 describe the minimum and maximum rotational size of a molecule or fragment with respect to a defined rotation axis. This axis was defined as the Pd–P bond (direction 1), the Pd–X bond with X being a secondary donor (direction 2) (Figure S14). Only the phosphine ligand itself was considered for the Sterimol calculation. All remaining atoms (Cl- and Ph-ligands) were excluded.

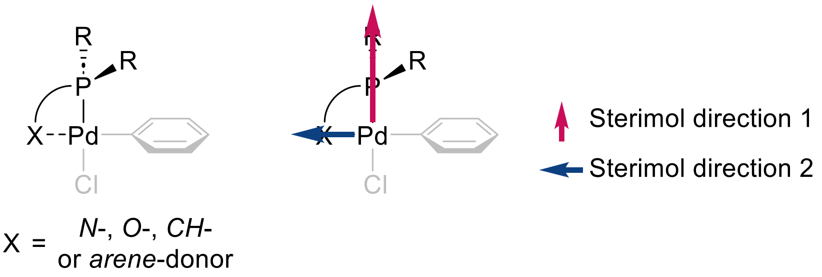

Figure S14: Definition of the three Sterimol directions.

Buried volume*:[44] The buried volume describes the space occupied by a ligand in the first coordination sphere around the metal center. It was developed with a radius of 3.5 Å for the first coordination sphere, which also is the radius used here. The center of the sphere is the palladium atom and any atoms not belonging to the phosphine ligand again were excluded. The orientation of the complex always was chosen in a way that the Ph-substituent shows in the eastern direction and the total volume of the north-western quadrant is larger than that of the south-western quadrant (Figure S15). Next to the buried volumes of cardinal hemispheres, the buried volumes of the *near* and *far* hemisphere are measured. Next to the buried volume the total volume always was obtained with the same definitions.

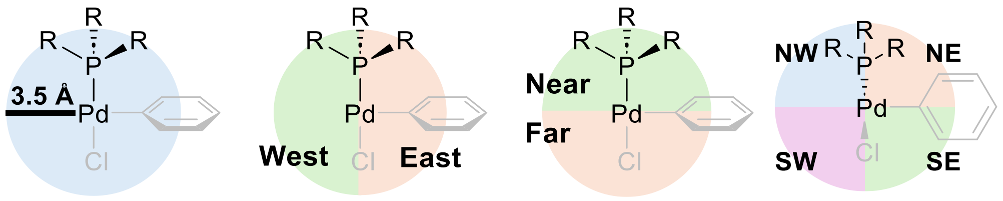

Figure S15: Schematic representation of the buried volume property with definitions of the hemispheres and quadrants.

Solid angle*: The solid angle of the phosphine ligand with the palladium atom as center of the sphere was calculated.

Solvent accessible surface area*:[45] The solvent accessible surface area was obtained for the full complex and for the palladium-center only.

Pyramidalization*:[46] The P-value of the phosphor-donor is obtained as well as for the ylidic carbon (YPhos only) and the phosphonium atom (YPhos only).

Dispersion descriptor P_{int}*:[47] The dispersion descriptor P_{int} and the interaction area A_{int} as well as the product of both P_{int} x A_{int} was obtained for the phosphine ligand with the palladium-center. The Cl- and Ph-ligands were excluded.

Bond lengths: The bond lengths of the Pd–P, Pd–Cl, Pd–C_{Ph} and Pd–X bonds were obtained (Figure S16).

Bond angles: The angles between the C_{Ph}–Pd–Cl atoms and P–Pd–X atoms were obtained (Figure S16).

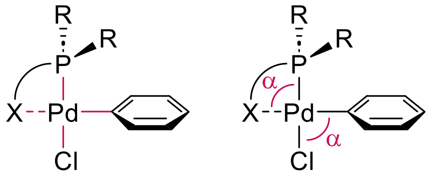

Figure S16: Measured bond lengths and angles for general phosphine ligands and YPhos ligands.

Molecular, electronic properties:

HOMO-LUMO-Gap: The energy difference between the highest occupied molecular orbital (HOMO) and the lowest unoccupied molecular orbital (LUMO) based on the Kohn-Sham orbitals obtained from DFT. η = E(LUMO) – E(HOMO)
Molecular electronegativity/chemical potential: The average energy of the highest occupied molecular orbital (HOMO) and the lowest unoccupied molecular orbital (LUMO) based on the Kohn-Sham orbitals obtained from DFT. $\mu = (E(\text{LUMO})+E(\text{HOMO})) / 2$

Global electrophilicity index: Metric of the lewis acidity of the complex. $\omega = \mu^2 / 2\eta$

Global nucleophilicity index: Metric of the lewis basicity of the complex. $N = 1 / \omega$

Dipole moment: Dipolemoment of the molecule

Solvation energy: Energy difference of the electronic energies of the gas phase and the solvation phase single points. $E_{solv} = SCF_{solv} - SCF_{gas}$

Oxidation energy: Energy difference of the electronic energies of the neutral molecule and the radical cation (rc). $E_{ox} = SCF_{rc} - SCF_{neutral}$

Reduction energy: Energy difference of the electronic energies of the neutral molecule and the radical anion (ra). $E_{red} = SCF_{ra} - SCF_{neutral}$

NBO partial charges: NBO derived partial charges were obtained for the following atoms: Pd, Cl, C_{Ph}, P, X, (C_{ylid}), (P_{onium}), ($C_{backbone}$)

Condensed Fukui functions: The condensed Fukui functions for nucleophilic and electrophilic attack were calculated based on the NBO derived partial charges for the following atoms: Pd, Cl, C_{Ph}, P, X, (C_{ylid}), (P_{onium}), ($C_{backbone}$).

Nucleophilic attack $\boldsymbol{f}^{+} = q(\text{NBO})_{neutral} - q(\text{NBO})_{ra}$

Electrophilic attack $\boldsymbol{f}^{-} = q(\text{NBO})_{rc} - q(\text{NBO})_{neutral}$

Additional properties

Nuclear electrostatic potential: The molecular electrostatic potential (MEP) at the nuclei position is calculated with the orca_vpot module of ORCA 5.0.4 based on the gas-phase single points for the following atoms: Pd, Cl, CPh, P.

Principal interacting orbitals (PIO):[48] The principal interacting orbitals (PIO) analysis builds upon the NBO analysis and was performed using the respective python software package.[49] To quantify the strength of interaction between two fragments, these two fragments have to be defined by their atom indices. The [Pd]-L interaction for example is represented by two fragments, one containing the full phosphine ligand and the other fragment containing the remaining [PdClPh] complex (Figure S17). The resulting interactions between these two fragments are sorted by their relative strength with the strongest interaction being the Pd–P σ-bond, the second strongest being the Pd–P π-back bonding or Pd–X bond, and so on. Next to the indices of every single interaction (PIO-derived bond indices, PBI), a total interaction between both fragments is given, which represents the sum of all individual indices (PBItot).

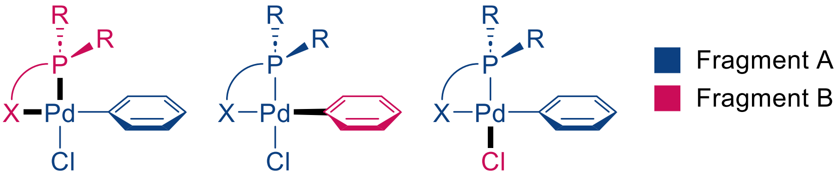

Figure S17: Definition of fragments for PIO analyses to obtain bond strengths.

Condensation of properties to descriptors

After obtaining all properties for every conformer of a ligand system, these properties were condensed to the ligand descriptors. For example, the buried volume Vbur values of all conformers are condensed to the following descriptors:
Vbur (min): minimum value over all conformers
Vbur (max): maximum value over all conformers
ΔVbur: difference between the maximum and minimum value over all conformers
Vbur (boltz): Boltzmann-weighted average over all considered conformers. Default descriptor if not mentioned otherwise
Every property therefore yields four descriptors that are assigned to the ligand. For the min-, max- and Δ- descriptors, all conformers with a relative energy up to 6.0 kcal/mol and for the Boltzmann-weighted average only those conformers up to 3.0 kcal/mol are considered, using final free energies at the PBE0-D3BJ/def2-TZVP/SMD(THF)//r²SCAN-3c level of theory.

All computed descriptors for the evaluated ligands are available in the attached descriptors_combined.csv file.

Ligand Structures

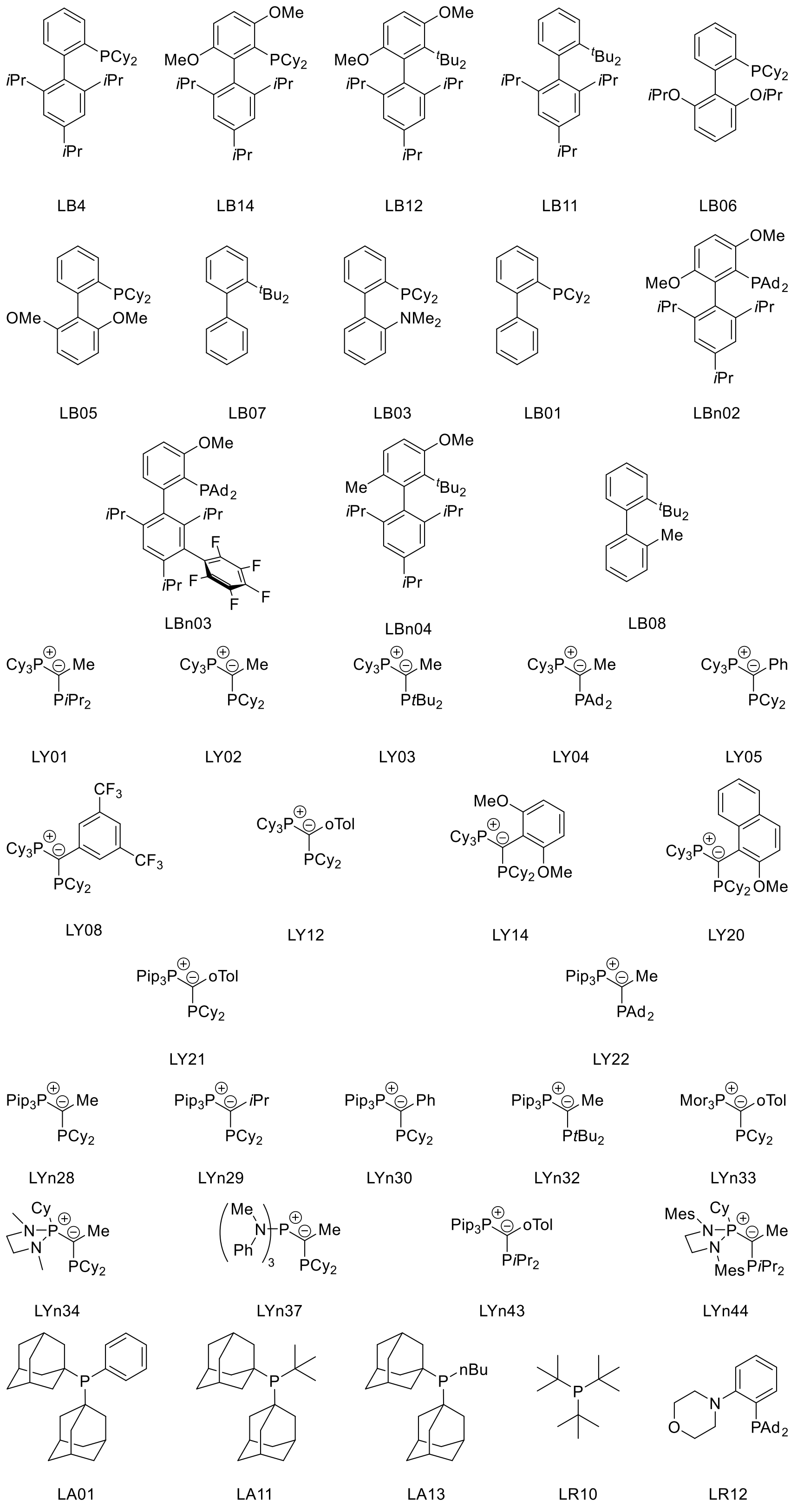

Figure S18: Ligand structures and indices.

Statistical Modeling:

General Considerations:

The statistical modeling was performed using Python 3.10.6. Descriptor preprocessing, model building and evaluation were implemented with scikit-learn (0.19.2), while additional data handling and visualization was performed using NumPy, Pandas and Matplotlib. A fixed random seed (42) was used throughout to ensure reproducibility. All ligand descriptors were standardized using Z-score scaling via scikit-learn's StandadScaler. The dataset was divided into training and test sets in an 80:20 ratio according to Kennard-Stone algorithm, ensuring a uniform spread across descriptor space. Model robustness was assessed using the coefficient of determination R^2, mean absolute error (MAE), leave one out cross validation (Q^2) and repeated K-fold cross validation with 5 folds and 200 repetitions. To identify predictive descriptor combinations, we implemented a custom forward stepwise feature selection algorithm. Starting from all possible one or two variable models, features were iteratively added to form up to 4 term models, provided that the added features did not exceed a pairwise collinearity threshold of $R^2 > 0.5$. At each step, the 100 best-performing candidates based on cross-validated Q^2 were retained and extended. In addition to forward construction, a one-step-back pruning strategy was applied to test whether removing individual features improved performance, helping to avoid local minima. Candidate models were filtered post hoc using following robustness criteria: 1) test set R^2 had to lie within ± 0.15 of the training set R^2. 2) Q^2 had to be within 0.2 of the training R^2. 3) test MAE could not exceed five times the training MAE. These thresholds were chosen to ensure reliable generalization. Code for data preprocessing, model training and evaluation as well as visualization is provided within the attached jupyter notebook and in the end of the ESI.

Data Processing:

From the raw yields, chemo- and diastereoselectivity were calculated as follows:

$$S = \frac{Anti + Syn}{Conversion - Anti - Syn}$$

$$dr = \frac{Anti}{Syn}$$

Using these selectivity values, the corresponding free energy differences were determined according to:

$$\Delta\Delta G = -RT\ ln(Selectivity)$$

Where T = 298.15 K and R = 0.00198588 kcal mol^{-1} K^{-1}

With an estimated error of ±2 in the raw yields, the respective errors for chemo- and diastereoselectivity were determined using standard error propagation formulas. For a function $f(x1, x2, \ldots)$, the propagated error is given by:

$$\sigma_f = \sqrt{\left(\frac{\partial f}{\partial x1} * \sigma_{x1}\right)^2 + \left(\frac{\partial f}{\partial x2} * \sigma_{x2}\right)^2 + \cdots}$$

For chemoselectivity:

$$Numerator = (Anti + Syn),\ \ Denominator = (Conversion - Anti - Syn)$$

$$\sigma_S = S * \sqrt{\left(\frac{\sqrt{\sigma_{Anti}^2 + \sigma_{Syn}^2}}{Anti + Syn}\right)^2 + \left(\frac{\sqrt{\sigma_{Conversion}^2 + \sigma_{Anti}^2 + \sigma_{Syn}^2}}{Conversion - Anti - Syn}\right)^2}$$

For diastereoselectivity:

$$\sigma_{dr} = dr * \sqrt{\left(\frac{\sigma_{Anti}}{Anti}\right)^2 + \left(\frac{\sigma_{Syn}}{Syn}\right)^2}$$

Error in Free Energy:

$$\sigma_{\Delta\Delta G} = RT * \frac{\sigma_{Selectivity}}{Selectivity}$$

Modeling Results for Diastereoselectivity:

The selectivity data ranges from -1.62 kcal/mol to 1.5 kcal/mol (range = 3.13 kcal/mol). Datapoints with an error greater than 10% on the range were removed. The remaining datapoints were split in an 80/20 ratio into training and test set according to the Kennard Stone algorithm to maximize feature diversity in the training set.

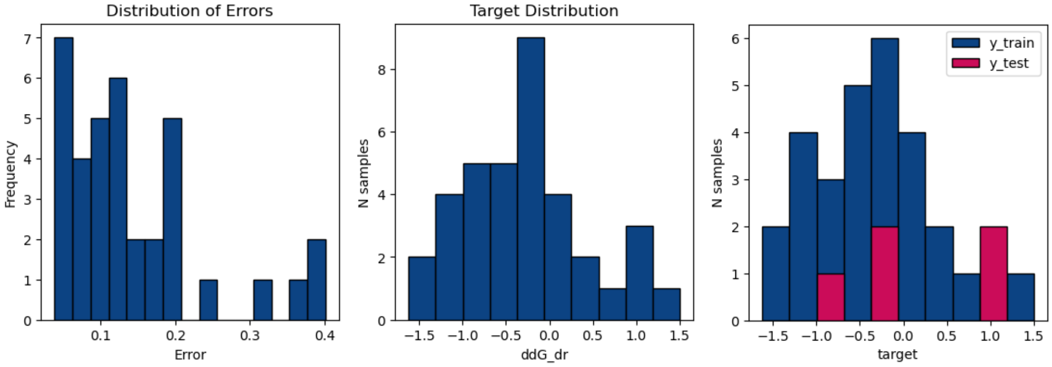

Figure S19: Distribution of errors, targets and training and testing sets.

The resulting ligand splits are as follows:

Training set ligands:
['LB04', 'LB14', 'LB12', 'LB11', 'LB06', 'LY02', 'LY01', 'LY03', 'LY05', 'LY12', 'LY18', 'LY13', 'LY14', 'LY08', 'LYn34', 'LYn37', 'LYn30', 'LY21', 'LYn43', 'LYn29', 'LYn28', 'LYn32', 'LY22', 'LB08', 'LY04', 'LA11','LYn33', 'LB03', 'LBn02']

Test set ligands:
['LB07', 'LYn44', 'LB05', 'LY20', 'LA01']

External Validation ligand:
['LBn04'] (RockPhos)

This achieves near zero median y values for TS and VS:
y_mean training set: -0.306
y_mean test set: 0.151

Final Model Output:
The following model was obtained from sequential feature selection:

$$\Delta\Delta G = -0.3056 - 0.4621 * d(Pd - P)_{boltz} - 0.1486 * qvbur_{boltz}^{\Delta NENW} + 0.4598 * nuesp_{boltz}^{sec} - 0.2413 * B1_{delta}^{sec}$$

List of parameters named in the csv file:
-0.3056 +
-0.4621 * d_Pd_P_boltz
-0.1486 * qvbur_full_delta_NENW_boltz
0.4598 * nuesp_X_boltz
-0.2413 * sterimol_B1_X_delta

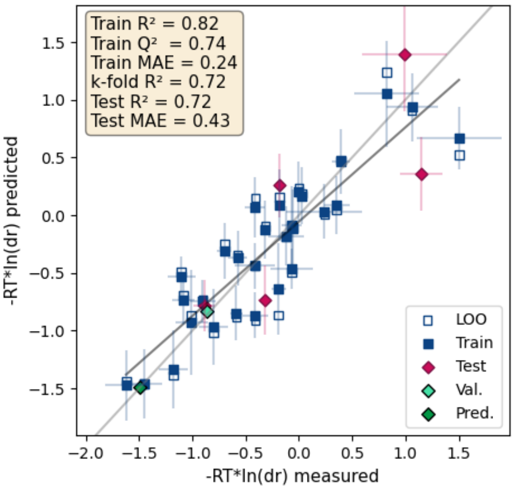

Training R^2 = 0.817
Training Q^2 = 0.739
Training MAE = 0.240

Training k-fold R^2 = 0.726 (+/- 0.001)

Test R^2 = 0.720
Test MAE = 0.431

Training/Test: 29/5

External Validation (RockPhos):
Predicted dr: 4.1
Experimental dr: 4.3

OLS summary:
All four descriptors are statistically significant ($p < 0.05$).

OLS Regression Results

Dep. Variable:	y	R-squared	0.817
Model:	OLS	Adj. R-squared	0.786
Method:	Least Squares	F-statistic	26.73
Date:	Tue, 10 Jun 2025	Prob (F-statistic	1.55e-08
Time:	15:35:03	Log-Likelihood	-6.5773
No. Observations:	29	AIC	23.15
Df Residuals:	24	BIC	29.99
Df Model:	4		

	coef	std err	t	P>|t|	[0.025	0.975]
Const	-0.3056	0.062	-4.931	0.000	-0.433	-0.178
0	-0.4621	0.063	-7.388	0.000	-0.591	-0.333
1	-0.1486	0.070	-2.129	0.044	-0.293	-0.005
2	0.4598	0.074	6.175	0.000	0.306	0.613
3	-0.2413	0.068	-3.551	0.002	-0.382	-0.101

Omnibus:	2.484	Durbin-Watson:	1.388
Prob(Omnibus):	0.289	Jarque-Bera (JB):	1.408
Skew:	0.517	Prob(JB):	0.495
Kurtosis:	3.308	Cond. No.	1.89

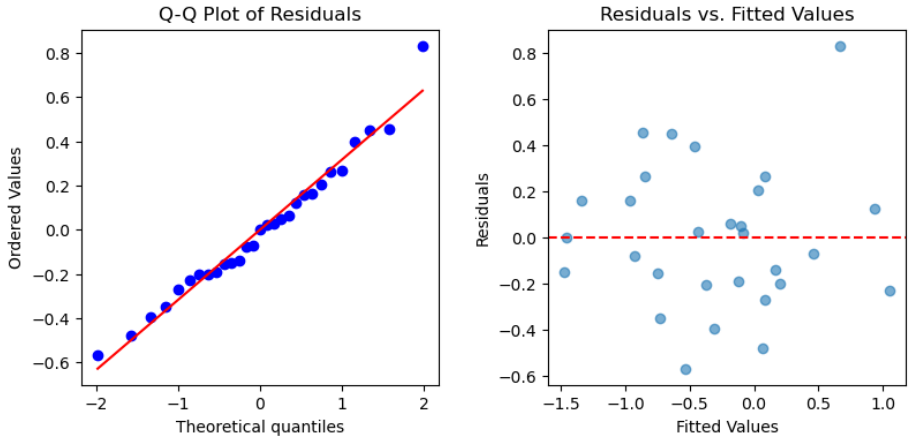

Figure S20: Q-Q Plot of residuals and plot of residuals vs fitted values.

Residuals show normality and hosmoscedasticity:
Shapiro-Wilk Test Statistic: 0.978, p-value: 0.772
Breusch-Pagan Test Statistic: 2.839, p-value: 0.585

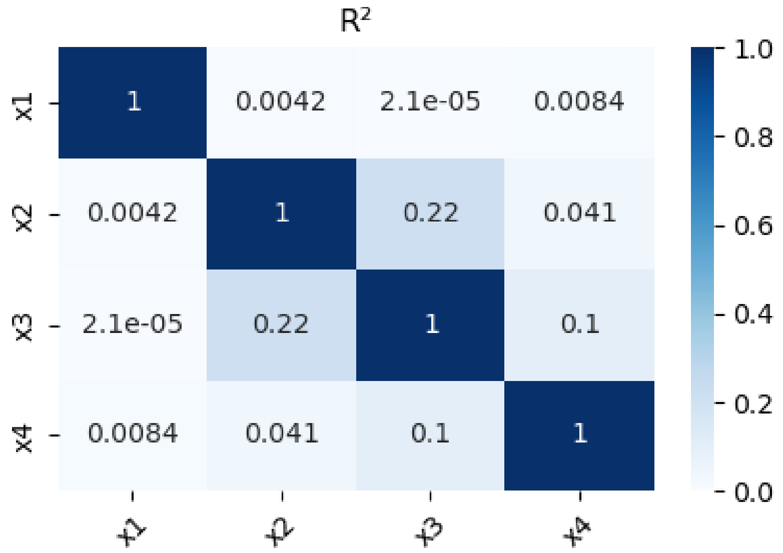

Figure S21: Feature correlation matrix.

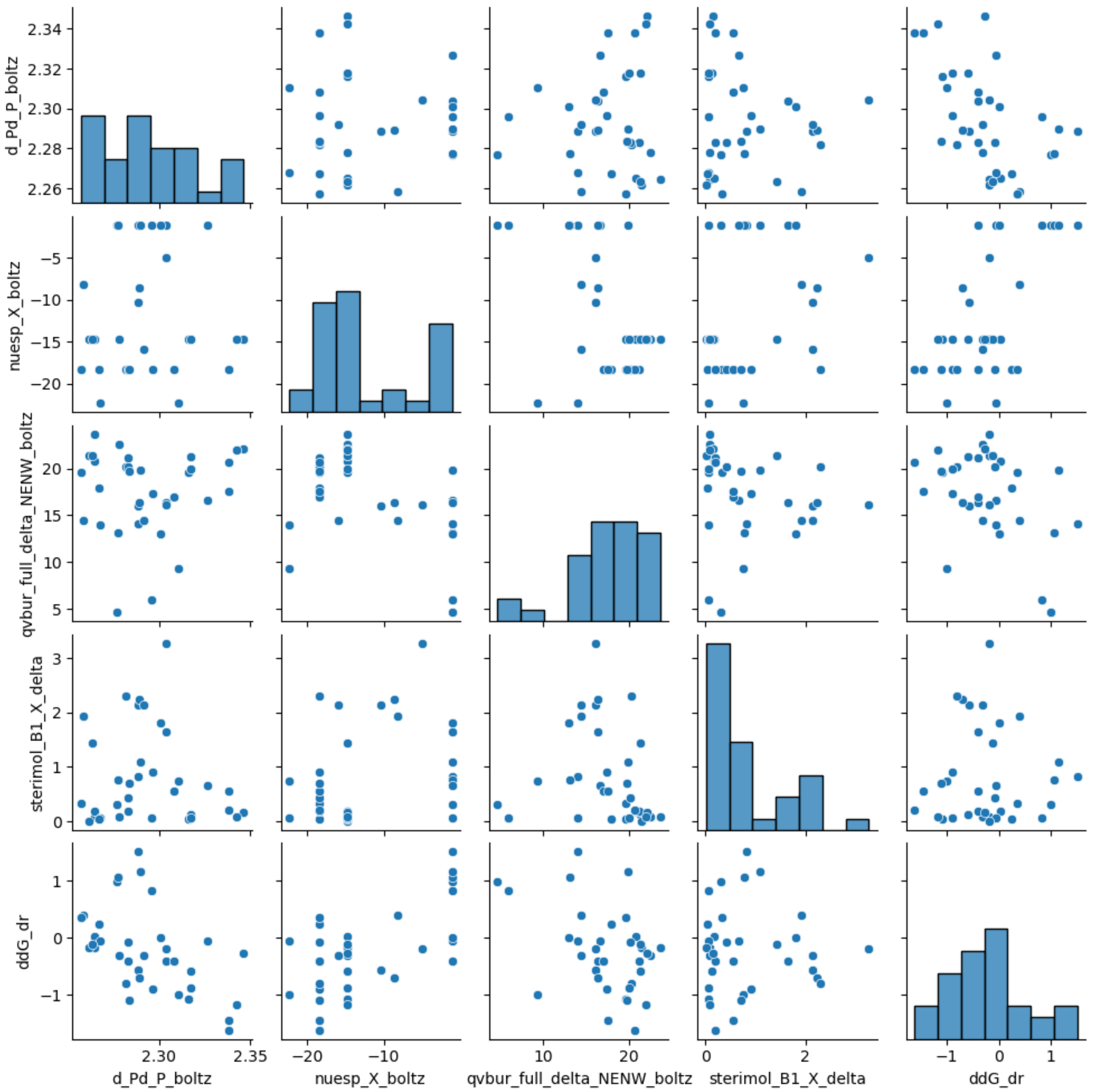

Figure S22: Feature pair plot.

In [ ]:
```
import numpy as np
import matplotlib.pyplot as plt
import seaborn as sns
import pandas as pd

from sklearn import metrics
from sklearn.preprocessing import StandardScaler
from sklearn.linear_model import LinearRegression
from sklearn.model_selection import RepeatedKFold,LeaveOneOut

import statsmodels.api as sm

import warnings
warnings.filterwarnings("ignore")

randomstate = 42
temp = 298.15
R = 0.00198588

colors = {"CP_darkblue": '#0c4383',
"CP_lightblue": "#22a0b6",
"CP_türkis": "#46E0A6",
"CP_darkred": '#7b1346',
"CP_lightred": '#cb0c59',
"CP_orange": "#ed8554",
"CP_green":"#029645",
"CP_yellow":"#c8b029"}
```

In [ ]:
```
# Metric functions

def r2_val(y_test,y_pred_test,y_train):
    """Calculates the external R2 pred as described:
    https://
pdfs.semanticscholar.org/4eb2/5ff5a87f2fd6789c5b9954eddddfd1c59da
    y_resid = y_pred_test - y_test
    SS_resid = np.sum(y_resid**2)
    y_var = y_test - np.mean(y_train)
    SS_total = np.sum(y_var**2)
    r2_validation = 1-SS_resid/SS_total
    return(r2_validation)

### LeaveOneOut



def get_q2(X,y,model=LinearRegression()):

    loo = LeaveOneOut()
    ytests = []
    ypreds = []
    for train_idx, test_idx in loo.split(X):
        X_train, X_test = X[train_idx], X[test_idx]
        y_train, y_test = y[train_idx], y[test_idx]

        model.fit(X_train,y_train)
        y_pred = model.predict(X_test)

        ytests += list(y_test)
        ypreds += list(y_pred)

    rr = metrics.r2_score(ytests, ypreds)
    return(rr,ypreds)



def repeated_k_fold(X_train,y_train,reg = LinearRegression(),
k=5, n=100):
    """Reapeated k-fold cross-validation.
    For each of n repeats, the (training)data is split into k
```

```
folds.
    For each fold, this part of the data is predicted using the
rest.
    Once this is done for all k folds, the coefficient of
determination (R^2) of the predictions of all folds combined (=
the complete data set) is evaluated
    This is repeated n times and all n R^2 are returned for
averaging/further analysis
    """

    rkf = RepeatedKFold(n_splits=k, n_repeats=n)
    r2_scores = []
    y_validations,y_predictions = np.zeros((np.shape(X_train)
[0],n)),np.zeros((np.shape(X_train)[0],n))
    foldcount = 0
    for i,foldsplit in enumerate(rkf.split(X_train)):
        fold, rep = i%k, int(i/k) # Which of k folds. Which of n
repeats
        model =
reg.fit(X_train[foldsplit[0]],y_train[foldsplit[0]]) #
foldsplit[0]: k-1 training folds
        y_validations[foldcount:foldcount+len(foldsplit[1]),rep]
= y_train[foldsplit[1]] # foldsplit[1]: validation fold
        y_predictions[foldcount:foldcount+len(foldsplit[1]),rep]
= model.predict(X_train[foldsplit[1]])
        foldcount += len(foldsplit[1])
        if fold+1==k:
            foldcount = 0
    r2_scores =
np.asarray([metrics.r2_score(y_validations[:,rep],y_predictions[:
for rep in range(n)])
    return(r2_scores)



def kennardstonealgorithm( X, k ):
    """
    @author: Hiromasa Kaneko
    https://github.com/hkaneko1985/kennardstonealgorithm
    --- input ---
    X : dataset of X-variables (samples x variables)
```

```
    k : number of samples to be selected

    --- output ---
    selectedsamplenumbers : selected sample numbers (training
data)
    remainingsamplenumbers : remaining sample numbers (test
data)
    """
    X = np.array( X )
    originalX = X
    distancetoaverage = ( (X - np.tile(X.mean(axis=0),
(X.shape[0], 1) ) )**2 ).sum(axis=1)
    maxdistancesamplenumber = np.where( distancetoaverage ==
np.max(distancetoaverage) )
    maxdistancesamplenumber = maxdistancesamplenumber[0][0]
    selectedsamplenumbers = list()
    selectedsamplenumbers.append(maxdistancesamplenumber)
    remainingsamplenumbers = np.arange( 0, X.shape[0], 1)
    X = np.delete( X, selectedsamplenumbers, 0)
    remainingsamplenumbers = np.delete( remainingsamplenumbers,
selectedsamplenumbers, 0)
    for iteration in range(1, k):
        selectedsamples = originalX[selectedsamplenumbers,:]
        mindistancetoselectedsamples = list()
        for mindistancecalculationnumber in range( 0,
X.shape[0]):
            distancetoselectedsamples = ( (selectedsamples -
np.tile(X[mindistancecalculationnumber,:],
(selectedsamples.shape[0], 1)) )**2 ).sum(axis=1)
            mindistancetoselectedsamples.append(
np.min(distancetoselectedsamples) )
        maxdistancesamplenumber = np.where(
mindistancetoselectedsamples ==
np.max(mindistancetoselectedsamples) )
        maxdistancesamplenumber = maxdistancesamplenumber[0][0]

selectedsamplenumbers.append(remainingsamplenumbers[maxdistancesa
        X = np.delete( X, maxdistancesamplenumber, 0)
        remainingsamplenumbers = np.delete(
remainingsamplenumbers, maxdistancesamplenumber, 0)
```

```
    return(selectedsamplenumbers, remainingsamplenumbers)
```

Selectivity and Error Propagation Formulas

The **ChemoSelectivity** is calculated as:

$$\text{ChemoSelectivity} = \frac{\text{anti} + \text{syn}}{\text{conversion} - \text{anti} - \text{syn}}$$

The error in **ChemoSelectivity** is calculated as:

$$\sigma_{\text{ChemoSelectivity}} = \text{ChemoSelectivity} \cdot \sqrt{\left(\frac{\sigma_{\text{numerator}}}{\text{numerator}}\right)^2 + \left(\frac{\sigma_{\text{denominator}}}{\text{denominator}}\right)^2}$$

where:

$$\sigma_{\text{numerator}} = \sqrt{\sigma_{\text{anti}}^2 + \sigma_{\text{syn}}^2}, \quad \sigma_{\text{denominator}} = \sqrt{\sigma_{\text{conversion}}^2 + \sigma_{\text{anti}}^2 + \sigma_{\text{syn}}^2}$$

The **DiastereoSelectivity** is given by:

$$\text{DiastereoSelectivity} = \frac{\text{anti}}{\text{syn}}$$

The error propagation for **DiastereoSelectivity** is:

$$\sigma_{\text{DiastereoSelectivity}} = \text{DiastereoSelectivity} \cdot \sqrt{\left(\frac{\sigma_{\text{anti}}}{\text{anti}}\right)^2 + \left(\frac{\sigma_{\text{syn}}}{\text{syn}}\right)^2}$$

For the (Δ Δ G), we use:

$$\Delta\,\Delta\,\text{G} = -RT \cdot \ln(\text{Selectivity})$$

The propagated error in (Δ Δ G) is:

$$\sigma_{\Delta\,\Delta\,\text{G}} = RT \cdot \frac{\sigma_{\text{Selectivity}}}{\text{Selectivity}}$$

In [3]:
```
# Load Datasets
df_X1 =
pd.read_csv("bi_gen_red_filt.csv").set_index("Lig_ID",drop=True)
df_X2 =
pd.read_csv("bi_bi_red_filt.csv").set_index("Lig_ID",drop=True)
df_X = pd.merge(df_X1, df_X2,how='inner',
on='Lig_ID').drop(['V_min_boltz','V_min_r_boltz'],axis = 1)
df_ext = df_X.loc["LYn50"] # Not synthesizeable MoradYPhos
Ligand
df_val = df_X.loc["LBn04"] # External Validation (RockPhos)
df_X = df_X.drop(["LYn50","LBn04"],axis = 0)
X = np.asarray(df_X)

df_y = pd.read_csv("targets.csv").set_index("Lig_ID",drop=True)
df_y = df_y[df_y.index.notnull()]
df_y["conversion"] = 100 - df_y["arcl"]

# Assuming all GC errors in raw values are +- 2
error_value = 2

sigma_num_chemo = np.sqrt(error_value**2 + error_value**2)  #
error in (product_anti + product_syn)
sigma_den_chemo = np.sqrt(error_value**2 + error_value**2 +
error_value**2)  # error in (conversion - product_anti -
product_syn)
num_chemo = df_y["anti"] + df_y["syn"]
den_chemo = df_y["conversion"] - df_y["anti"] - df_y["syn"]

df_y["ChemoSelectivity"] = (df_y["anti"] + df_y["syn"]) /
(df_y["conversion"] - df_y["anti"] - df_y["syn"])
df_y["ChemoSelectivity_error"] = df_y["ChemoSelectivity"] *
np.sqrt((sigma_num_chemo / num_chemo)**2 + (sigma_den_chemo /
den_chemo)**2)

df_y["DiastereoSelectivity"] = df_y["anti"] / df_y["syn"]
df_y["DiastereoSelectivity_error"] =
df_y["DiastereoSelectivity"] * np.sqrt((error_value /
df_y["anti"])**2 + (error_value / df_y["syn"])**2)

df_y["ddG_chemo"] = -1 * temp * R *
np.log(df_y["ChemoSelectivity"])
```

```
[illegible]
df_y["ddG_chemo_error"] = R * temp *
(df_y["ChemoSelectivity_error"] / df_y["ChemoSelectivity"])

df_y["ddG_dr"] = -1 * temp * R *
np.log(df_y["DiastereoSelectivity"])
df_y["ddG_dr_error"] = R * temp *
(df_y["DiastereoSelectivity_error"] /
df_y["DiastereoSelectivity"])
```

In [4]:

```
hues = {
    "NYPhos":colors["CP_darkblue"],
    "YPhos":colors["CP_lightred"],
    "Buchwald":colors["CP_green"],
    "Trialkyl":colors["CP_lightblue"]}

markers = ['o', 's', '^', 'v']

# Plor dr vs chemoselectivity
fig,ax = plt.subplots(figsize=(4, 3.6))
sns.scatterplot(data=df_y.drop(["LR12"],axis=0), x="ddG_chemo",
y="ddG_dr", s=70, style="Type", hue="Type",
                palette=hues, markers=markers).set(xlabel = "-
RT*ln(S)",ylabel = "-RT*ln(dr)")

plt.legend()
plt.tight_layout()
plt.show()
```

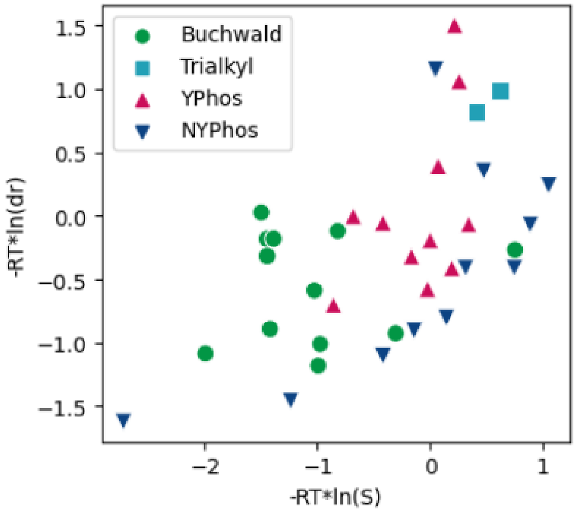

In [5]:
```
df_y.head()
```

Out[5]:

Lig_ID	Ligand	Type	anti	syn	dehalo	arom	arcl	conversion	ChemoSelectivity	Ch
LB04	XPhos	Buchwald	53	39	0	4	0	100	11.500000	
LB14	BrettPhos	Buchwald	58	34	0	3	0	100	11.500000	
LB12	tBu-BrettPhos	Buchwald	44	8	9	0	38	62	5.200000	
LB11	tBu-XPhos	Buchwald	62	23	4	0	0	100	5.666667	
LB06	RuPhos	Buchwald	43	45	6	0	5	95	12.571429	

Diastereoselectivity

In [6]:
```
# Filter
df_y_dr = df_y[~df_y['ddG_dr'].isin([np.nan, np.inf, -np.inf])]

print(f'ddG ranges from {df_y_dr["ddG_dr"].min():.3} to
{df_y_dr["ddG_dr"].max():.3} kcal/mol')
print(f'Range =
{(abs(df_y_dr["ddG_dr"].min())+abs(df_y_dr["ddG_dr"].max())):.3}
kcal/mol')
print(f'ddG Errors range from {df_y_dr["ddG_dr_error"].min():.3}
to {df_y_dr["ddG_dr_error"].max():.3} kcal/mol')
print(f'Average Error = {df_y_dr["ddG_dr_error"].mean():.3}
kcal/mol')
```

```
ddG ranges from -1.62 to 1.5 kcal/mol
Range = 3.13 kcal/mol
ddG Errors range from 0.0377 to 0.402 kcal/mol
Average Error = 0.143 kcal/mol
```

In [7]:
```
# Plotting the error distribution
plt.figure(figsize=(4, 4))
plt.hist(df_y_dr['ddG_dr_error'],
bins=15,color=colors["CP_darkblue"], edgecolor='black')
plt.xlabel('Error')
plt.ylabel('Frequency')
plt.title('Distribution of Errors')
plt.show()
```

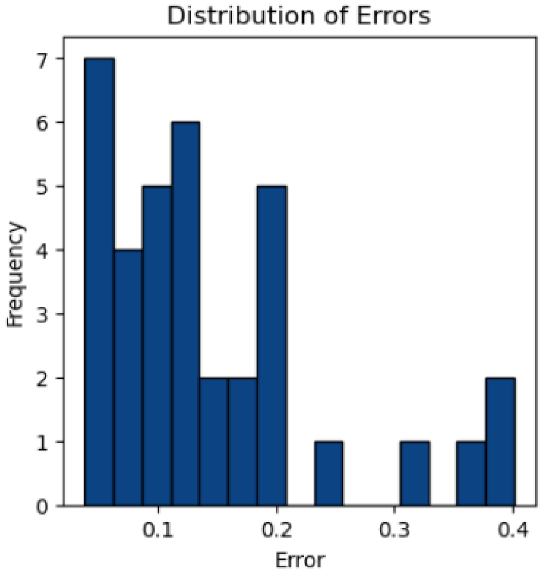

In [8]:
```
y_target = "ddG_dr"
y_target_errors = "ddG_dr_error"

# prepare targets
y_exp = pd.to_numeric(df_y_dr[y_target].dropna(),
errors='coerce')
y_errors = pd.to_numeric(df_y_dr[y_target_errors].dropna(),
errors='coerce')

y = np.asarray([float(i) for i in
np.asarray(y_exp).reshape(-1)])
y_err = np.asarray([float(i) for i in
np.asarray(y_errors).reshape(-1)])
y_val = df_y_dr[y_target].loc["LBn04"]

df_y_dr.drop(["LBn04"],axis=0,inplace=True)

df_X = df_X.loc[df_y_dr[y_target].dropna().index,:]
df_X = df_X.drop([i for i in df_X.columns if
(len(df_X[i].unique()) == 1 or len(df_X[i].unique()) == 0)],
axis = "columns")

X = np.asarray(df_X)
X_ext = np.asarray(df_ext).reshape(1, -1)
X_val = np.asarray(df_val).reshape(1, -1)

df_X_y = pd.concat([df_X,df_y_dr],axis=1)

fig,ax = plt.subplots(figsize=(4,4))
plt.hist(y_exp, bins=10, color=colors["CP_darkblue"],
edgecolor='black')
plt.xlabel(y_target)
plt.ylabel("N samples")
plt.title("Target Distribution")
plt.show()
```

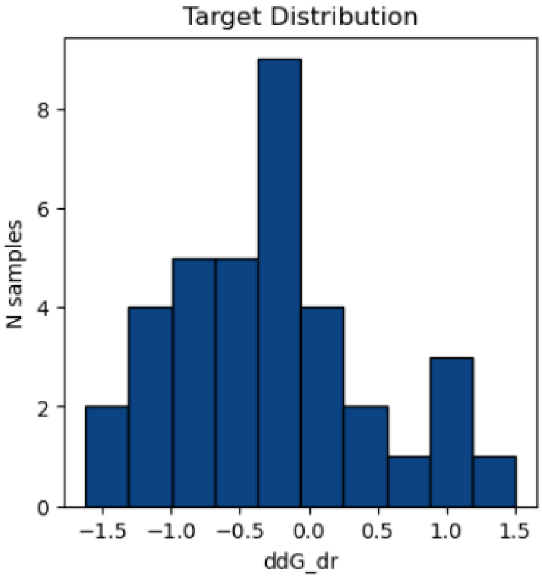
Target Distribution
N samples
ddG_dr
0
2
4
6
8
−1.5
−1.0
−0.5
0.0
0.5
1.0
1.5

In [ ]:
```
# Split Data according to KS Algorithm
X_sel,y_sel,labels_sel = X.astype(float),y,df_y_dr.index
rs = 42
test_ratio = 0.8

VS,TS = kennardstonealgorithm(X_sel,int((1-
test_ratio)*np.shape(X_sel)[0]))

X_train, y_train, y_train_err = X[TS], y[TS], y_err[TS]
X_test, y_test, y_test_err = X[VS], y[VS], y_err[VS]

print("TS: {}".format(([df_X.index[i] for i in TS])))
print("VS: {}".format(([df_X.index[i] for i in VS])))
print("y_mean TS: {:.3f}".format(np.mean(y_train)))
print("y_mean VS: {:.3f}".format(np.mean(y_test)))
print("Shape X_train: {}".format(X_train.shape))
print("Shape X_test:  {}".format(X_test.shape))
fig,ax = plt.subplots(figsize=(4, 4))
hist,bins = np.histogram(y_sel,bins=10)
plt.hist(y_train, bins, alpha=1,
label='y_train',color=colors["CP_darkblue"], edgecolor='black')
plt.hist(y_test, bins, alpha=1, label='y_test',
color=colors["CP_lightred"], edgecolor='black')
plt.legend(loc='best')
plt.xlabel("target")
plt.ylabel("N samples")
plt.show()

# Scale Data
scaler = StandardScaler()
X_train_sc = scaler.fit_transform(X_train)
X_test_sc = scaler.transform(X_test)
X_ext_sc = scaler.transform(X_ext)
X_val_sc = scaler.transform(X_val)
```

```
TS: ['LB04', 'LB14', 'LB12', 'LB11', 'LB06', 'LY02', 'LY01', 'LY03', 'LY0
5', 'LY12', 'LY18', 'LY13', 'LY14', 'LY08', 'LYn34', 'LYn37', 'LYn30', 'L
Y21', 'LYn43', 'LYn29', 'LYn28', 'LYn32', 'LY22', 'LB08', 'LY04', 'LA11',
'LYn33', 'LB03', 'LBn02']
VS: ['LB07', 'LYn44', 'LB05', 'LY20', 'LA01']
y_mean TS: -0.306
y_mean VS: 0.151
Shape X_train: (29, 194)
Shape X_test:  (5, 194)
```

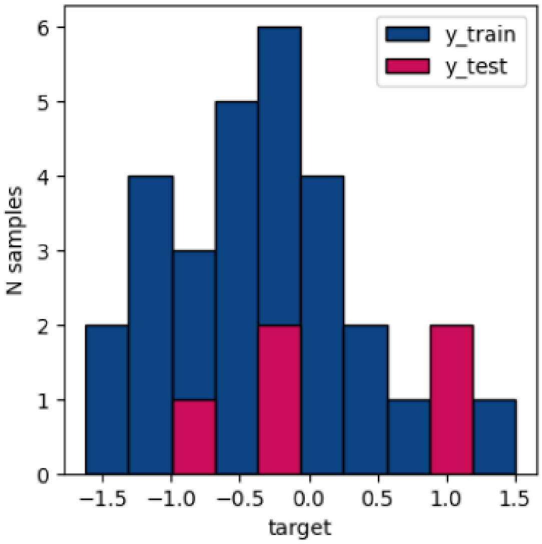

Model

In [10]:
```
features_names =
["d_Pd_P_boltz","nuesp_X_boltz","qvbur_full_delta_NENW_boltz","st
features_py = sorted([list(df_X.columns).index(i) for i in
features_names])
features_py = sorted(features_py)
X_train_sel = X_train_sc[:,features_py]
X_test_sel = X_test_sc[:,features_py]
X_ext_sel = X_ext_sc[:,features_py]
X_val_sel = X_val_sc[:,features_py]

lr = LinearRegression().fit(X_train_sel, y_train)

y_pred_train = lr.predict(X_train_sel)
y_pred_test =  lr.predict(X_test_sel)
y_pred_ext = lr.predict(X_ext_sel)
y_pred_val = lr.predict(X_val_sel)
q2,loo_train = get_q2(X_train_sel,y_train)
kfoldscores = repeated_k_fold(X_train_sel,y_train,k=5,n=200)

print("\nParameters:\n{:10.4f} + \n".format(lr.intercept_) +
"\n".join(["{:10.4f} *
{}".format(lr.coef_[i],df_X.columns[sorted(features_py)[i]]) for
i in range(len(features_py))]))
print(f"\nTraining R2  = {lr.score(X_train_sel,
y_train):.3f}\nTraining Q2  = {q2:.3f}")
print(f"Training MAE =
{metrics.mean_absolute_error(y_train,y_pred_train):.3f}")
print("Training K-fold R2 = {:.3f} (+/-
{:.3f})".format(kfoldscores.mean(), kfoldscores.std() ** 2))
print(f"\nTest R2      =
{r2_val(y_test,y_pred_test,y_train):.3f}\nTest MAE     =
{metrics.mean_absolute_error(y_test,y_pred_test):.3f}")
print(f"\nTraining/Test: {y_train.shape[0]}/{y_test.shape[0]}")

model = sm.OLS(y_train,
sm.add_constant(pd.DataFrame(X_train_sel))).fit()
print(model.summary())
```

```
Parameters:
   -0.3056 +
   -0.4621 * d_Pd_P_boltz
   -0.1486 * qvbur_full_delta_NENW_boltz
    0.4598 * nuesp_X_boltz
   -0.2413 * sterimol_B1_X_delta

Training R2  = 0.817
Training Q2  = 0.739
Training MAE = 0.240
Training K-fold R2 = 0.723 (+/- 0.001)

Test R2      = 0.720
Test MAE     = 0.431

Training/Test: 29/5
                            OLS Regression Results
==========================================================================
Dep. Variable:                      y   R-squared:
Model:                            OLS   Adj. R-squared:
Method:                 Least Squares   F-statistic:
Date:                Tue, 10 Jun 2025   Prob (F-statistic):           1.5
Time:                        17:34:26   Log-Likelihood:               -6
No. Observations:                  29   AIC:
Df Residuals:                      24   BIC:
Df Model:                           4
Covariance Type:            nonrobust
==========================================================================
                 coef    std err          t      P>|t|      [0.025      0
--------------------------------------------------------------------------
const         -0.3056      0.062     -4.931      0.000     -0.433      -
0             -0.4621      0.063     -7.388      0.000     -0.591      -
1             -0.1486      0.070     -2.129      0.044     -0.293      -
2              0.4598      0.074      6.175      0.000      0.306
3             -0.2413      0.068     -3.551      0.002     -0.382      -
==========================================================================
Omnibus:                        2.484   Durbin-Watson:
Prob(Omnibus):                  0.289   Jarque-Bera (JB):
Skew:                           0.517   Prob(JB):
Kurtosis:                       3.308   Cond. No.
==========================================================================

Notes:
[1] Standard Errors assume that the covariance matrix of the errors is co
ly specified.
```

Model exploration

In [11]:
```
import scipy.stats as stats
import matplotlib.pyplot as plt

# Normal distribution of residuals
residuals = y_train - y_pred_train
fig,ax = plt.subplots(figsize=(4, 4))
stats.probplot(residuals, dist="norm", plot=ax)
plt.title("Q-Q Plot of Residuals")
plt.show()
```

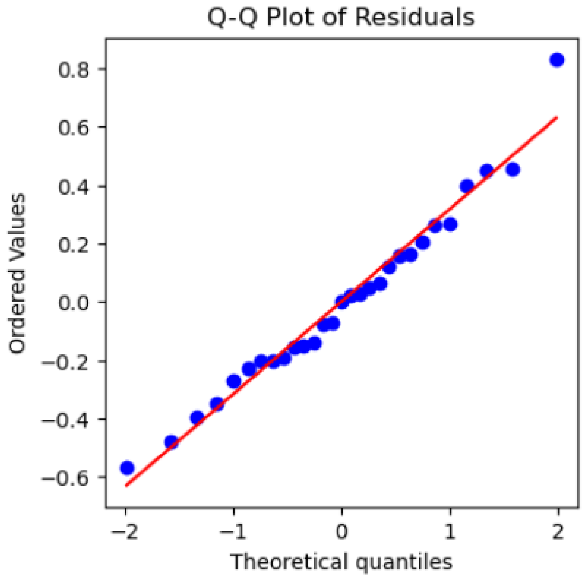

In [12]:
```
# Shapiro-Wilk Test
stat, p = stats.shapiro(residuals)
print(f"Shapiro-Wilk Test Statistic: {stat:.3f}, p-value:
{p:.3f}")
```

```
Shapiro-Wilk Test Statistic: 0.978, p-value: 0.772
```

In [13]:
```
import statsmodels.stats.diagnostic as smd

# Residuals vs. Fitted Values Plot
fig,ax = plt.subplots(figsize=(4, 4))
plt.scatter(y_pred_train, residuals, alpha=0.6)
plt.axhline(0, color="red", linestyle="--")
plt.xlabel("Fitted Values")
plt.ylabel("Residuals")
plt.title("Residuals vs. Fitted Values")
plt.show()
```

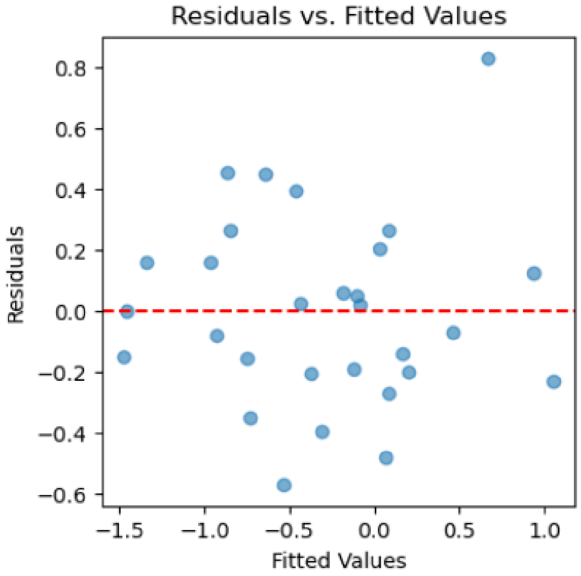

In [14]:
```
# Breusch-Pagan Test
bp_test = smd.het_breuschpagan(residuals,
sm.add_constant(X_train_sel))
print(f"Breusch-Pagan Test Statistic: {bp_test[0]:.3f}, p-value:
{bp_test[1]:.3f}")
```

```
Breusch-Pagan Test Statistic: 2.839, p-value: 0.585
```

In [15]:
```
# correlation matrix of features
new_df = pd.DataFrame()
for i in features_py:
    feature = df_X.columns[i]
    new_df[f"{feature}"] = X_sel[:,i]
new_df.columns = ["x1","x2","x3","x4"]
plt.figure(figsize=(5,3))
sns.heatmap(new_df.corr()**2, annot=True, cmap = "Blues", vmin =
0, vmax = 1)
plt.xticks(rotation=45)
plt.title("R²")
plt.show()
```

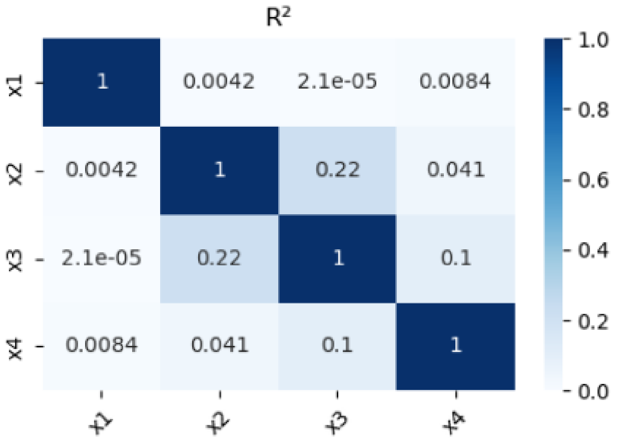

In [16]:
```
plt.figure(figsize=(1,1))
sns.pairplot(data = df_X_y[features_names+[y_target]], size=2)
plt.show()
```

```
<Figure size 100x100 with 0 Axes>
```

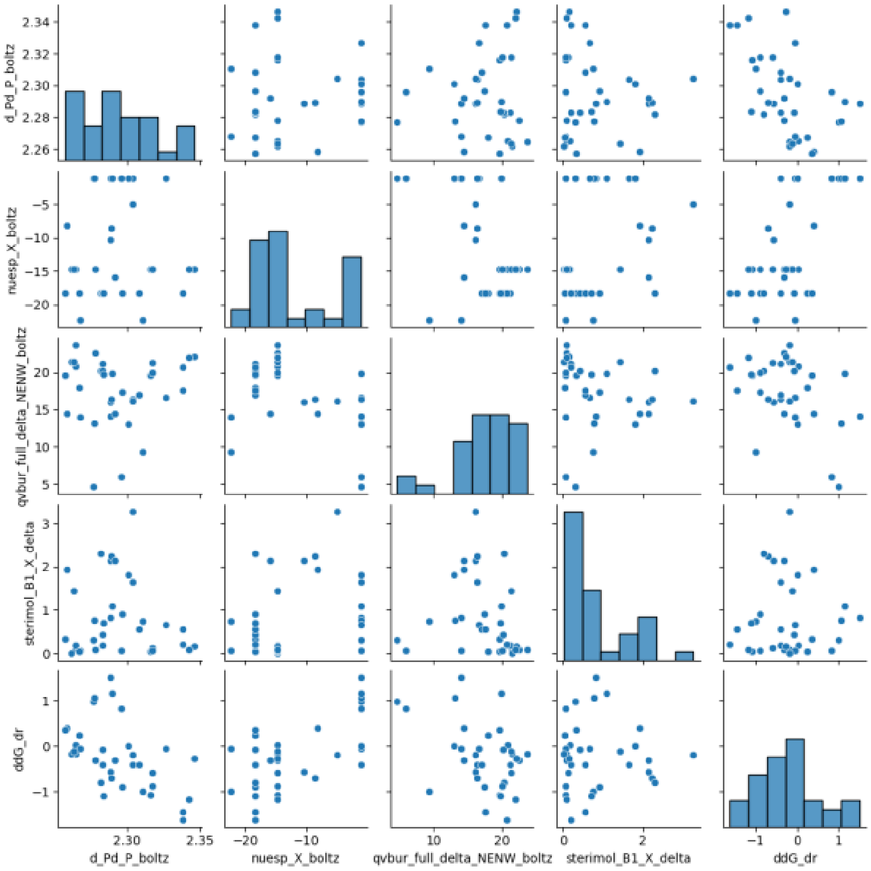

In [17]:

```
# prediction error
# half-width of confidence intervals of predictions using sm
prediction_summary =
model.get_prediction(sm.add_constant(X_test_sel))
confidence_intervals_test = prediction_summary.conf_int()
vertical_errors_test = (confidence_intervals_test[:, 1] -
confidence_intervals_test[:, 0]) / 2

prediction_summary =
model.get_prediction(sm.add_constant(X_train_sel))
confidence_intervals_train = prediction_summary.conf_int()
vertical_errors_train = (confidence_intervals_train[:, 1] -
confidence_intervals_train[:, 0]) / 2
```

In [18]:
```
# final plot
y_orig_min = np.min(np.hstack((y_train,y_test)))
y_pred_min = np.min(np.hstack((y_pred_train,y_pred_test)))
y_orig_max = np.max(np.hstack((y_train,y_test)))
y_pred_max = np.max(np.hstack((y_pred_train,y_pred_test)))
delta_x = 0.15 * (y_orig_max-y_orig_min)
delta_y = 0.15 * (y_pred_max-y_pred_min)

yy_fit = np.polyfit(y_train,y_pred_train,deg=1)
yy_fit_line = yy_fit[1]+yy_fit[0]*y_train

plt.figure(figsize=(5,5))
plt.plot(np.linspace(y_orig_min-delta_x,y_orig_max+delta_x),
         np.linspace(y_orig_min-
delta_x,y_orig_max+delta_x),color="grey",alpha=0.5)
plt.plot(sorted(y_train),sorted(yy_fit_line),color="black",alpha=

plt.xlim([y_orig_min-delta_x,y_orig_max+delta_x])
plt.ylim([y_pred_min-delta_y,y_pred_max+delta_y])

plt.errorbar(y_train, y_pred_train, xerr= y_train_err,
fmt="none",color=colors["CP_darkblue"], capsize=0,
linestyle="none", alpha = 0.25)
plt.errorbar(y_test, y_pred_test, xerr= y_test_err,
fmt="none",color=colors["CP_lightred"], capsize=0,
linestyle="none", alpha = 0.25)

plt.errorbar(y_test, y_pred_test, yerr=vertical_errors_test,
fmt="none", ecolor=colors["CP_lightred"], alpha=0.25, capsize=0)
plt.errorbar(y_train, y_pred_train, yerr=vertical_errors_train,
fmt="none", ecolor=colors["CP_darkblue"], alpha=0.25, capsize=0)


plt.scatter(y_train,loo_train,label="LOO",color=colors["CP_darkbl
plt.scatter(y_train,y_pred_train,label="Train",color=colors["CP_d
plt.scatter(y_test,y_pred_test,label="Test",color=colors["CP_ligh
= colors["CP_darkred"],marker="D",s=30)

plt.scatter(y_val,
y_pred_val,label="Val.",color=colors["CP_türkis"],edgecolors="bla
marker="D", s =35)
```

```
plt.scatter(y_pred_ext,y_pred_ext,label="Pred.", color=
colors["CP_green"],edgecolors="black", marker="D", s =35)


textstr = '\n'.join((
    f"Train R\u00b2 = {lr.score(X_train_sel, y_train):.2f}",
    f"Train Q\u00b2  = {q2:.2f}",
    f"Train MAE =
{metrics.mean_absolute_error(y_train,y_pred_train):.2f}",
    f"k-fold R\u00b2 = {round(kfoldscores.mean(),2)}",#\u00B1
{round(kfold_all.std()**2,3)}"
    f"Test R\u00b2 = {r2_val(y_test,y_pred_test,y_train):.2f}",
    f"Test MAE =
{metrics.mean_absolute_error(y_test,y_pred_test):.2f}"
    ))

props = dict(boxstyle='round', facecolor='wheat', alpha=0.5)
plt.text(0.12, 1.25, textstr, transform=ax.transAxes,
fontsize=11,
        verticalalignment='top', bbox=props)

label = "-RT*ln(dr)"
plt.legend(loc='lower right')
plt.xlabel(label+" measured",fontsize=11)
plt.ylabel(label+" predicted",fontsize=11)
plt.show()
```

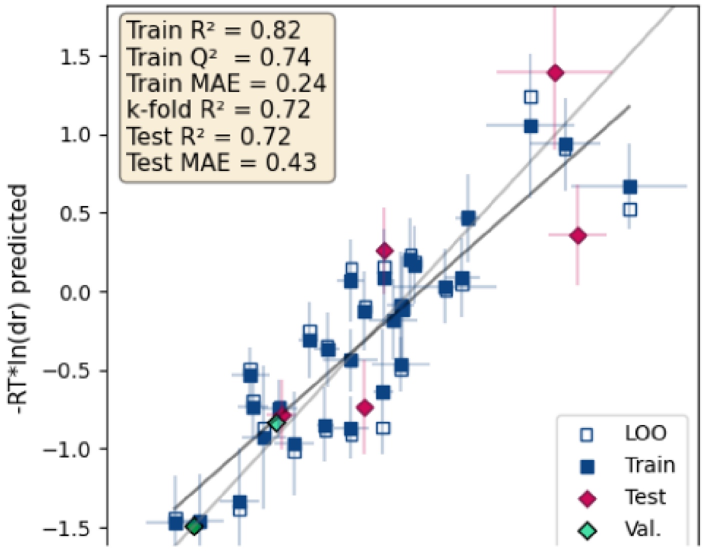

In [19]:
```
# dr Prediction of MoradYPhos Ligand
pred_dr = np.exp(-1*y_pred_ext[0]/(temp*R))
print(pred_dr)
```

```
12.402109017926
```

14. References

1 W. L. F. Armarego, W. L. F. Armarego and D. D. Perrin, *Purification of laboratory chemicals*, Butterworth-Heinemann, Oxford, 4. ed., reprint., 2002.
2 T. Scherpf, C. Schwarz, L. T. Scharf, J.-A. Zur, A. Helbig and V. H. Gessner, *Angew. Chem. Int. Ed.*, 2018, **57**, 12859–12864.
3 S. Lapointe, A. Sarbajna and V. H. Gessner, *Acc. Chem. Res.*, 2022, **55**, 770–782.
4 J. Löffler, N. Kaiser, D. Knyszek, F. Krischer, M. Jörges, K.-S. Feichtner and V. H. Gessner, *Angew. Chem. Int. Ed.*, 2024, e202408947.
5 E. P. Jones, P. Jones and A. G. M. Barrett, *Org. Lett.*, 2011, **13**, 1012–1015.
6 E. Yamamoto, K. Izumi, Y. Horita and H. Ito, *J. Am. Chem. Soc.*, 2012, **134**, 19997–20000.
7 C. W. Jefford, S. Mahajan, J. Waslyn and B. Waegell, *J. Am. Chem. Soc.*, 1965, **87**, 2183–2190.
8 J. J. Piwinski, J. K. Wong, T. M. Chan, M. J. Green and A. K. Ganguly, *J. Org. Chem.*, 1990, **55**, 3341–3350.
9 A. W. Schuppe, J. L. Knippel, G. M. Borrajo-Calleja and S. L. Buchwald, *J. Am. Chem. Soc.*, 2021, **143**, 5330–5335.
10 R. Devant and M. Braun, *Chem. Ber.*, 1986, **119**, 2191–2207.
11 U. Schöllkopf, S. Grüttner, R. Anderskewitz, E. Egert and M. Dyrbusch, *Angew. Chem. Int. Ed.*, 1987, **26**, 683–684.
12 G. Shang, Q. Yang and X. Zhang, *Angew. Chem.*, 2006, **118**, 6508–6510.
13 P. N. Basa, S. Antala, R. E. Dempski and S. C. Burdette, *Angew. Chem. Int. Ed.*, 2015, **54**, 13027–13031.
14 O. V. Dolomanov, L. J. Bourhis, R. J. Gildea, J. A. K. Howard and H. Puschmann, *J. Appl. Crystallogr.*, 2009, **42**, 339–341.
15 G. M. Sheldrick, *Acta Crystallogr. Sect. Found. Adv.*, 2015, **71**, 3–8.
16 G. M. Sheldrick, *Acta Crystallogr. Sect. C Struct. Chem.*, 2015, **71**, 3–8.
17 (a) P. Hohenberg and W. Kohn, *Phys. Rev.* 1964, **136**, B864; (b) W. Kohn and L. J. Sham, *Phys. Rev.* 1965, **140**, A1133.
18 (a) F. Neese, F. Wennmohs, U. Becker and C. Riplinger, *J Chem Phys* 2020, **152**, 224108; (b) F. Neese, *Wiley Interdiscip. Rev. Comput. Mol. Sci.* 2022, **12**, e1606.
19 a) P. Pracht, F. Bohle, S. Grimme, Phys. Chem. Chem. Phys. 2020, 22, 7169–7192. b) S. Grimme, J. Chem. Theory Comput. 2019, 155, 2847–2862.
20 C. Bannwarth, S. Ehlert and S. Grimme., J. Chem. Theory Comput. 2019, 15, 1652–1671.
21 (a) C. Adamo and V. Barone, *J. Chem. Phys.* 1999, **110**, 6158; (b) F. Weigend and R. Ahlrichs, *Phys. Chem. Chem. Phys.* 2005, **7**, 3297.
22 (a) S. Grimme, J. Antony, S. Ehrlich and H. Krieg, *J. Chem. Phys.* 2010, **132**, 154104; (b) S. Grimme, S. Ehrlich, L. Goerigk, *J. Comput. Chem.* 2011, **32**, 1456; (c) D. G. A. Smith, L. A. Burns, K. Patkowski, C. D. Sherrill, *J. Phys. Chem. Lett.* 2016, **7**, 2197.
23 V. Barone and M. Cossi, *J. Phys. Chem. A* 1998, **102**, 1995.
24 (a) P. J. Hay and W. R. Wadt, *J. Chem. Phys.* 1985, **82**, 270; (b) P. J. Hay and W. R. Wadt, *J. Chem. Phys.* 1985, **82**, 284; (c) P. J. Hay and W. R. Wadt, *J. Chem. Phys.* 1985, **82**, 299.
25 T. Gensch, G. dos Passos Gomes, P. Friederich, E. Peters, T. Gaudin, R. Pollice, K. Jorner, A. Nigam, M. Lindner-D'Addario, M. S. Sigman and A. Aspuru-Guzik, *J. Am. Chem. Soc.* 2022, **144**, 1205.
26 C. Bannwarth, S. Ehlert and S. Grimme., *J. Chem. Theory Comput.* 2019, **15**, 1652.
27 S. Ehlert, M. Stahn, S. Spicher and S. Grimme *J. Chem. Theory Comput.* 2021, **17**, 4250.
28 C. Bannwarth, E. Caldeweyher, S. Ehlert, A. Hansen, P. Pracht, J. Seibert, S. Spicher and S. Grimme *WIREs Comput. Mol. Sci.* 2020, **11**, e01493.
29 (a) P. Pracht, F. Bohle and S. Grimme, *Phys. Chem. Chem. Phys.* 2020, **22**, 7169; (b) S. Grimme, *J. Chem. Theory Comput.* 2019, **155**, 2847.
30 S. Grimme, F. Bohle, A. Hansen, P. Pracht, S. Spicher and M. Stahn, *J. Phys. Chem. A* 2021, **19**, 4039.
31 S. Grimme, *J. Comput. Chem.* 2006, **27**, 1787.
32 (a) F. Weigend and R. Ahlrichs, *Phys. Chem. Chem. Phys.* 2005, **7**, 3297; (b) F. Weigend, *Phys. Chem. Chem. Phys.* 2006, **8**, 1057.
33 (a) S. Spicher and S. Grimme, *J. Chem. Theory Comput.* 2021, **17**, 1701; b) H. Kruse and S. Grimme *J. Chem. Phys.* 2012, **136**, 154101.
34 (a) F. Neese, F. Wennmohs, U. Becker and C. Riplinger *J. Chem. Phys.* 2020, **152**, 224108. b) F. Neese, *WIREs Comput. Mol. Sci.* 2022, **12**, e1606.
35 (a) J. W. Furness, A. D. Kaplan, J. Ning, J. P. Perdew, and J. Sun, *J. Phys. Chem. Lett.* 2020, **11**, 8208; (b) J. W. Furness, A. D. Kaplan, J. Ning, J. P. Perdew, and J. Sun, *J. Phys. Chem. Lett.* 2020, **11**, 9248.
36 a) E. Caldeweyher, C. Bannwarth and S. Grimme, *J. Chem. Phys.* 2017, **147**, 034112; (b) E. Caldeweyher, S. Ehlert, A. Hansen, H. Neugebauer, S. Spicher, C. Bannwarth and S. Grimme, *J. Chem. Phys.* 2019, **150**, 154122.
37 S. Grimme, A. Hansen, S. Ehlert and J.-M. Mewes, *J. Chem. Phys.* 2021, **154**, 064103.
38 (a) C. Adamo and V. Barone, *J. Chem. Phys.* 1999, **110***,* 6158; (b) M. Ernzerhof and G. E. Scuseria *The Journal of Chemical Physics* 1999, **110**, 5029.
39 (a) S. Grimme, S. Ehrlich and L. Goerigk, *J Comput Chem.* 2011, **32**, 1456; (b) S. Grimme, J. Antony, S. Ehrlich and H. Krieg, *J. Chem. Phys.* 2010, **132**, 154104.
40 A. V. Marenich, C. J. Cramer and D. G. Truhlar, *J. Phys. Chem. B* 2009, **113**, 6378.
41 NBO 7.0. E. D. Glendening, J. K. Badenhoop, A. E. Reed, J. E. Carpenter, J. A. Bohmann, C. M. Morales, P. Karafiloglou, C. R. Landis and F. Weinhold, Theoretical Chemistry Institute, University of Wisconsin, Madison, WI (2018).
42 K. Jorner, https://github.com/digital-chemistry-laboratory/morfeus
43 A. Verloop, W. Hoogenstraaten, J. Tipker, In *Drug Design*; Ed. E. J. Ariëns, *Medicinal Chemistry: A Series of Monographs*, Academic Press: Amsterdam **1976**, *11*, 165–207.
44 A. C. Hillier, W. J. Sommer, B. S. Yong, J. L. Petersen, L. Cavallo and S. P. Nolan, *Organometallics* 2003, **22**, 4322.
45 (a) A. Shrake and J. A. Rupley, *Journal of Molecular Biology* 1973, **79**, 351; (b) P. L. Eisenhaber, P. Argos, C. Sander and M. Scharf, *Journal of Computational Chemistry* 1995, **16**, 273.
46 T. P. Radhakrishnan and I. Agranat, *Structural Chemistry* 1991, **2**, 107.
47 R. Pollice and P. Chen, *Angew. Chem. Int. Ed.* 2019, **58**, 9758.
48 (a) J.-X. Zhang, F. K. Sheong and Z. Lin, *Chem. Eur. J.* 2018, **24**, 9639; (b) J.-X. Zhang, F. K. Sheong and Z. Lin, *WIREs Comput. Mol. Sci.* 2020, **10**, e1469.
49 J.-X. Zhang, https://github.com/jxzhangcc/PIO
